# Glycemic Control of Diabetes Mellitus Patients in Referral Hospitals of Amhara Region, Ethiopia: A Cross-Sectional Study

**DOI:** 10.1155/2021/6691819

**Published:** 2021-01-16

**Authors:** Berhanu Elfu Feleke, Teferi Elfu Feleke, Melkamu Beyene Kassahun, Wondemu Gebrekirose Adane, Netsanet Fentahun, Abel Girma, Alamirew Alebachew, Eyaya Misgan, Hanna Demelash Desyibelew, Mulat Tirfie Bayih, Omer Seid

**Affiliations:** ^1^Department of Epidemiology and Biostatistics, University of Bahir Dar, Bahir Dar, Ethiopia; ^2^Department of Pediatrics and Child Health Wolkite University, Butajira General hospital, Ethiopia; ^3^Amhara National Regional State Health Bureau, Ethiopia; ^4^Global Tuberculosis Program, Addis Ababa, Ethiopia; ^5^Department of Nutrition and Dietetics, Bahir Dar University, Bahir Dar, Ethiopia; ^6^Department of Internal Medicine, Bahir Dar University, Bahir Dar, Ethiopia; ^7^Department of Pediatrics, Bahir Dar University, Bahir Dar, Ethiopia; ^8^Department of Gynecology and Obstetrics, Bahir Dar University, Bahir Dar, Ethiopia

## Abstract

**Background:**

Glycemic control is the level of glucose in diabetes patient. Evidence regarding glycemic control is scarce in resource-limited settings, and this study was conducted to generate information regarding the prevalence and predictors of glycemic control among diabetes mellitus patients attending their care from the referral hospitals of the Amhara region, Ethiopia.

**Methods:**

A cross-sectional study design was implemented. A simple random sampling technique was used. Data were collected from March 2018 to January 2020. The data were collected using interviews, chart review, and blood samples. Hemoglobin A1c was measured using high-performance liquid chromatography. Data were entered into Epi-info software and analyzed by SPSS software. Descriptive statistics were used to estimate the prevalence of glycemic control; linear regression was used to identify the predictors of HbA1c.

**Results:**

A total of 2554 diabetes patients were included giving for the response rate of 95.83%. The mean age of the study participants was 54.08 years [SD (standard deviation) ± 8.38 years]. The mean HbA1c of the study participants was 7.31% [SD ± 0.94%]. Glycemic control was poor in 55.32% [95% CI: 53.4%-57.25%] of diabetes patients. The glycemic control of diabetes patients was determined by BMI (*β* 0.1; [95% CI: 0.09-0.1]), type 2 diabetes (*β* -0.14; [95% CI: -0.11-0.16]), age (*β* 0.22; [95% CI: 0.02-0.024]), duration of the disease (*β* 0.04; [95% CI: 0.037-0.042]), the presence of hypertension (*β* 0.12; [95% CI:0.09–0.16]), regular physical exercise (*β* -0.06; [95% CI: -0.03-0.09]), medication adherence (*β* -0.16; [95% CI: -0.14-0.18]), and male (*β* 0.34; [95% CI: 0.31-.037]).

**Conclusion:**

The glycemic control of diabetes patients was poor, and it needs the attention of decision-makers.

## 1. Background

Diabetes mellitus defined as metabolic disorders characterized by the high blood glucose level, and the two major classifications of diabetes are type 1 diabetes and type 2 diabetes; type 2 diabetes accounts for the 90% of the total [1]. Diabetes affects 463 million people globally and expected to reach 700 million for the year 2045 [2]. Unfortunately, 50% of world diabetes cases were undiagnosed [3]. The burden of diabetes was rising especially in middle- and low-income countries; in 2045, the prevalence of diabetes expected to shift from 4.7% to 5.2% [3].

Type 1 DM is characterized by absolute insulin deficiency because of the destruction of islet beta cells in the pancreas occurring in the childhood period, while type 2 DM characterized by peripheral insulin resistance affecting the adults [4]. The exact etiology of type 1 DM was not known, but the combination of genetic and environmental factors plays a great role in its pathology. Type 2 diabetes primarily occurs as a result of obesity and lack of exercise [5]. The diagnosis of type 1 DM and type 2 DM was based on the patient characteristics mainly the age. Type 1 DM was managed by administering the insulin injection and life style modifications like adhering to the healthy foods, carbohydrate, proteins and fat counting, regular physical exercise, and frequent blood sugar monitoring [6]. Type 2 DM was managed by administering oral hypoglycemic agent and adhering to the life style modifications. Later, type 2 DM patient may demand insulin injection [7].

Glycemic control is a phrase given for the level of blood sugar in diabetes patient, and good glycemic control avoids the severity of complications and increases cognitive functioning [8]. Glycemic control can be evaluated by measuring the hemoglobin A1c (HbA1c) which notifies the average blood glucose level in the past 2-3 months [9].

Evidence on the current status of glycemic control in resource-limited settings was not updated; the available evidence on the glycemic control of DM patient was based on the fasting blood sugar level, and the available predictors were limited to the self-reporting predictors like residence, age, and sex. Different proportions of glycemic control were reported from resource-limited setting; in Jimma, Ethiopia, the proportion of good glycemic control ranges from 18.3% to 29.1% [10, 11]; in Gondar, it was 35.3% [12]; in the central Ethiopia, proportion of good glycemic control was 50% [13]; in Mekelle, the proportion of good glycemic control was 51.3% [14]; in the Southwest part of the country, 40.8% of DM patient had good glycemic control [15], and in the Northeast of Ethiopia, the proportion of good glycemic control was 29.2% [16]. The effects of BMI, duration of diabetes mellitus, hypertension, regular physical exercise, and total cholesterol level on the HbA1c were not properly assed in resource-limited setting. This study was conducted to generate information regarding the prevalence and determinant factors of glycemic control among diabetes mellitus patients.

## 2. Methods

A cross-sectional study design was implemented among diabetes mellitus patients attending the chronic clinics of the 5 referral hospitals in the Amhara regional state.

The study was conducted in Felege-Hiwot Referral Hospital, Gondar University hospital, Dessie Referral Hospital, Debre Berhan Referral Hospital, and Debre-Markos Referral Hospital. The data were collected using interviews, measuring anthropometric indicators, chart review, and collecting the blood samples. Data were collected from March 2018 to January 2020. The patient interview was conducted by clinical nurses. The target population for the study was all diabetes mellitus patients attending their chronic care, and diabetes mellitus patients unwilling to participate were excluded from the study.

The history of hypertension was crosschecked from the patient's medical chart. The weight and height of each study participants were measured by clinical nurses. The digital weight scale was used to measure the weight of each study participant, and weight was measured to the nearest 0.1 kilograms. The height was measured using the vertical measuring rod to the nearest 0.1 cm. The participant's shoes, hair clips, and braids were removed during the measurement, and participants were positioned feet together, feet flat on the ground, heels touching the backplate of the measuring instrument, legs straight, buttocks against the backboard, scapula against the backboard, and arms were loosely at their side [17, 18].

The postprandial glucose level was measured after 2 hours of the patient meal using American Diabetes Association Standards [19], and its normal reference range was less than 140 mg/dl for adults under the age of 50 years, 150 mg/dl for adults 50-60 years, and 160 mg/dl for elderly patients greater than 60 years [20]. Regular physical activity was measured using the 7 International Physical Activity Questionnaire (IPAQ) [21]. Medication adherence was measured using the 10-Item Medication Adherence Scale (MARS) [22]. HbA1c was measured using high-performance liquid chromatography, and its levels greater than 7% indicate bad glycemic control [23]. The total cholesterol level was measured using Hitachi 704 Analyzer (the normal reference range was less than 200 mg/dl) [24].

The sample size was calculated using Epi-info software version 7 with the assumption of 95% CI, 85% power, ratio of type 1 DM to type 2 DM 1 : 2, and 10% non-response rate level which gives 2657 diabetes patients (885 type 1 DM and 1772 type 2 DM). A simple random sampling technique was used by using their medical record number as a sampling frame.

The training was given for data collectors and supervisors, and the standard operating procedures were adhered during the blood sample collection and analysis. The data were entered using Epi-info software and transferred to SPSS version 25 for the analysis. Descriptive statistics were used to describe the profile of study participants and estimate the prevalence of glycemic control. Linear regression was used to identify the determinants of HbA1c levels of diabetes patients. Two independent sample *t*-tests were used to see the effects of hypertension, types of diabetes mellitus, sex, and regular physical exercise on the HbA1c levels.

Ethical clearance was obtained from the Bahir Dar University College of Medicine and health sciences ethical review board with an ethical approval number of CMHS/IRB/52/2018. Permission was obtained from the respective authorities of each hospital. Written informed consent was obtained from each study participant. The confidentiality of the data was kept at all steps. Study participants the right to withdraw from the study at any steps were respected. Study participants with abnormal laboratory findings were linked to curative care.

## 3. Results

A total of 2554 diabetes patients were included with the response rate of 96%, 41 study participants were excluded due to their consent, 23 diabetes patients were excluded due to poor laboratory samples, and 47 study participants were excluded due to missing necessary variables during data collection. The mean age of the study participants was 54.08 years [SD (standard deviation) ± 8.38 years]. Male constitutes 61.2% of the study participants, and 68.3% of the diabetes patients were type 2 diabetes cases (Tables 1 and 2).

The mean HbA1c of the study participants was 7.31% [SD ± 0.94%]. Glycemic control was poor in 55.32% [95% CI: 53.4%-57.25%] of diabetes patients. After adjusting for sex, type of diabetes mellitus, total cholesterol, body mass, index, duration of diabetes, regular physical exercise, and medication adherence, the glycemic control of diabetes patients was determined by BMI, type of diabetes, age, duration of the disease, the presence of hypertension, regular physical exercise, medication adherence, and sex (Table 3).

## 4. Interpretation of Table 3

One unit increase in the patient BMI increases the patient HbA1c by 0.1% (*β* 0.1; [95% CI: 0.09-0.1]). Per a year increase in the age of DM patient, the HbA1c of the patient increases by 0.02% [*β* 0.22; [95% CI: 0.02-0.024]). Per a year increase in the duration of the disease, the patient HbA1c increases by 0.04% (*β* 0.04; [95% CI: 0.037-0.042]). The mean HbA1c of diabetes patients in the presence of hypertension comorbidity was 8.16%, and the mean HbA1c of diabetes patients in the absence of hypertension was 7.17% (*β* 0.12; [95% CI: 0.09–0.16]). The mean HbA1c of DM patients in the absence of regular physical exercise was 7.68%, and in the presence of regular physical exercise, it was 6.59% (*β* -0.06; [95% CI: -0.03-0.09]). One mg/dl increase in the total cholesterol level of the patient increases the HbA1c by 0.007% (*β* .007; [95% CI: .006-.008]). One scale increase in the medication adherence scale decreases the HbA1c by 0.16%(*β* -0.16; [95% CI:-0.14-0.18]). The mean HbA1c of female DM patients was 6.73%, and the mean HbA1c of male patients was 7.69% (*β* 0.34; [95% CI: 0.31-.037]).

## 5. Discussion

The mean HbA1c of the study participants was 7.31% [SD ± 0.94%]. Glycemic control was poor in 55.32% [95% CI: 53.4%-57.25%] of diabetes patients. This finding was lower when compared to the research output from Egypt [25], and this finding agrees with the finding from Bangladesh [26], India [27], Ethiopia [28]. This finding alerts that more than half of diabetes mellitus patients were not properly regulating their HbA1c, which calls the attention of decision-makers to give priority to handle the unwanted consequences of diabetes mellitus in the resource-limited settings.

The mean HbA1c for type 1 DM patients was 7.8%, and the mean HbA1c for type 2 DM patients was 7.07%. This finding agrees with the finding from Gondar, Ethiopia [29]. This indicates that as the duration of DM increases, patients usually become negligent on their glycemic control, and their medication adherence scale drops as the duration of DM increases (the mode duration of DM in type 1 patients was 24 years, while the mode for type 2 DM was 2 years).

Per one unit increase in the patient BMI, the HbA1c of the patient increases by 0.1%. This finding agrees with the finding from India [27]. This is due to the effect of weight gain in poor glycemic control patients [30]. The prevalence of overweight and obesity was increasing dramatically in resource-limited settings [31, 32], and it makes the glycemic control difficult and calls the attention of decision-makers.

Per a year increase in the age of DM patient, the HbA1c increases by 0.02%. This finding agrees with the finding from India [27]. This is due to the occurrence of diabetes-related complications within the higher ages [33]. This implies that, the older the age not only increases the risk of chronic illness, the management of the illnesses also becomes difficult.

Per a year increase in the duration of the disease, the HbA1c increases by 0.04%. This finding agrees with the finding from India [34] and Yemen [35]. This is due to the appearance of chronic diabetes complications and numerous episodes of acute complications with longer diabetes duration [36, 37]. This implies that the diabetes mellitus intervention should work with other chronic illness activities for the better outcomes.

The mean HbA1c of diabetes patients in the presence of hypertension comorbidity was 8.16%, and the mean HbA1c of diabetes patients in the absence of hypertension was 7.17%. This finding agrees with the finding from Egypt [25] and China [38]. This is because the additional antihypertensive pill burden and complication inhibit the utilization of peripheral glucose which finally increases the glycated hemoglobin level [39]. According to the World Health Organization report, more than 1.13 billion people were hypertensive, and unfortunately, two thirds of world hypertensive patients were living in resource-limited settings that alert attention in the area for good glycemic control [40].

The mean HbA1c of DM patients in the absence of regular physical exercise was 7.68%, and in the presence of regular physical exercise, it was 6.59%. This finding agrees with the finding from Yemen [35] and Sri Lanka [41]. This is due to the effect of regular physical exercise on insulin production and metabolism of the available glucose [42, 43]. Physical inactivity was prevalent in diabetes mellitus patients; in this study, 33.6% [95% CI: 31.76%-35.43%] that signifies one of the priority area should focus on it.

One mg/dl increase in the total cholesterol level of the patient increases the HbA1c by 0.007%. This finding agrees with the Asians research results [44]. This is due to the poor dietary habits of patients with a high total cholesterol level [45]. The mean TCL of DM patients in the study area was 230 mg/dl, which signifies that the patients are having the bad cholesterol level above the reference range.

One unit increase in the medication adherence scale decreases the HbA1c by 0.16%. This finding was in line with the previously conducted research from Ethiopia [28] and Sri Lanka [41]. This is due to inadequate diabetes intervention in poorly adhered patients [46]. Results from this research work indicate that significant proportion of diabetes mellitus patients were not adhered to their drugs, and this will facilitate to the inefficient effects of the drugs.

The mean HbA1c of female DM patients was 6.73%, and the mean HbA1c of male patients was 7.69%. This finding disagrees with the finding from Bangladesh [26]. This is due to the unhealthy lifestyle of male, because in the study area, the problematic alcohol intake and cigarette smoking are high in male gender which finally increase the glycated hemoglobin [47].

The main limitation of this research work was a failure to generate information regarding the short-term and long-term complications of poor glycemic control.

## 6. Conclusion

Glycemic control was poor among diabetes patients. Duration of diabetes, hypertension, sex, TCL, medication adherence, age, BMI, and types of diabetes were the predictors of glycemic control. Behavioral factors (Medication Adherence Scale, obesity, regular physical exercise) and the consequences of old age (high duration of DM, higher age, and hypertension) were predominantly hindering the glycemic control of DM patients. Diabetes patients should be monitored for their dietary practice and regular physical exercise to decrease their TCL. Physicians should critically follow the adherence of the patients for their medications.

## Figures and Tables

**Table 1:** Profile of the study participants (*n* = 2554).

Variables	Type 1 DM		Type 2 DM	
	Mean	SD	Mean	SD
Age	56.62	9.15	52.91	7.73
Body mass index	26	2.9	23.85	2.67
Medication adherence scale	6.58	1.31	7.44	1.48
Total cholesterol level in mg/dl	239.47	24.73	225	27.77
Duration of the disease in years	11.46	6.43	11.12	5.54

**Table 2:** Categorical variables information for the study participants (*n* = 2554).

Serial Number	Variables		Type 1 DM		Type 2 DM	
			Frequency	Percentage	Frequency	Percentage
1	Regular physical exercise	Present	160	18.6	698	81.4
		Absent	649	38.3	1047	61.7
2	Hypertension	Present	154	42	213	58
		Absent	655	29.9	1532	70.1
3	Sex	Male	601	38.5	961	61.5
		Female	208	21	784	79

**Table 3:** Linear regression outputs for determinants of glycemic control among diabetes patients (*n* = 2554).

Variables	*Β* coefficient	Std. error	t	*P* value	95.0% confidence interval for *B*	
					Lower bound	Upper bound
Body mass index	.101	.003	29.669	<0.01	.095	.108
Types of diabetes	-.136	.013	-10.344	<0.01	-.161	-.110
Age	.022	.001	24.344	<0.01	.020	.024
Duration of diabetes mellitus	.039	.001	30.652	<0.01	.037	.042
Hypertension	.120	.018	6.746	<0.01	.085	.155
Regular physical exercise	-.056	.016	-3.598	<0.01	-.087	-.026
Total cholesterol level in mg/dl	.007	.001	12.608	<0.01	.006	.008
Medication adherence rating scale	-.158	.010	-15.216	<0.01	-.179	-.138
Sex	.342	.016	21.893	<0.01	.312	.373

## Data Availability

The underlying data of the research are attached in the supplementary file and can be accessed without restriction.

## Abbreviations

*μ*g/dl: Microgram per deciliter
AOR: Adjusted odds ratio
B: Beta coefficient
CI: Confidence interval
COR: Crude odds ratio
DM: Diabetes mellitus
ELISA: Enzyme-linked immune sorbent assay
g/dl: Gram per deciliter
HbA1c: Hemoglobin A one c
IPAQ: International Physical Activity Questionnaire
IQR: Interquartile range
MARS: Medication Adherence Scale
mg/dl: Milligram per deciliter
ng/dl: Nanogram per deciliter
SD: Standard deviation
WHO: World Health Organization.

## Supplementary Materials

Supplementary Materials The supplementary material contains the raw data of the research.
