# Supplementary material for: Glycemic Control of Diabetes Mellitus Patients in Referral Hospitals of Amhara Region, Ethiopia: A Cross-Sectional Study
Source: Biomed Res Int. 2021 Jan 16;2021:6691819. doi: 10.1155/2021/6691819 (PMC7834781; doi:10.1155/2021/6691819)
Supplement: Supplementary Materials — The supplementary material contains the raw data of the research. [file 6691819.f1.pdf]

| ID | FBG | BMI | TYPEOFDI | PBGL | AGE | HGA1C | EXERCISE |
|---|---|---|---|---|---|---|---|
| 1549 | 98 | 25 | 1 | 200 | 51 | 5.925 | 1 |
| 1411 | 98 | 25 | 1 | 200 | 42 | 5.925 | 1 |
| 1542 | 98 | 25 | 1 | 200 | 51 | 5.98 | 1 |
| 1410 | 98 | 25 | 1 | 200 | 42 | 5.98 | 1 |
| 1541 | 98 | 25 | 1 | 200 | 51 | 6.035 | 1 |
| 1406 | 98 | 25 | 1 | 200 | 42 | 6.035 | 1 |
| 1540 | 98 | 25 | 1 | 200 | 51 | 6.09 | 1 |
| 1405 | 98 | 25 | 1 | 200 | 42 | 6.09 | 1 |
| 1539 | 98 | 25 | 1 | 200 | 50 | 6.145 | 1 |
| 1400 | 98 | 25 | 1 | 200 | 42 | 6.145 | 1 |
| 1538 | 98 | 25 | 1 | 200 | 51 | 6.2 | 1 |
| 1399 | 98 | 25 | 1 | 200 | 42 | 6.2 | 1 |
| 1186 | 97 | 24 | 1 | 190 | 49 | 6.24 | 1 |
| 1512 | 97 | 24 | 1 | 190 | 50 | 6.25 | 1 |
| 1537 | 98 | 25 | 1 | 200 | 50 | 6.255 | 1 |
| 1391 | 98 | 25 | 1 | 200 | 42 | 6.255 | 1 |
| 1509 | 97 | 24 | 1 | 190 | 50 | 6.26 | 1 |
| 1508 | 97 | 24 | 1 | 190 | 50 | 6.27 | 1 |
| 1507 | 97 | 24 | 1 | 190 | 50 | 6.28 | 1 |
| 1505 | 97 | 24 | 1 | 190 | 50 | 6.29 | 1 |
| 1499 | 97 | 24 | 1 | 190 | 49 | 6.3 | 1 |
| 1766 | 91 | 20 | 1 | 140 | 49 | 6.302 | 1 |
| 1761 | 91 | 20 | 1 | 140 | 49 | 6.303 | 1 |
| 1760 | 91 | 20 | 1 | 140 | 49 | 6.304 | 1 |
| 1735 | 91 | 20 | 1 | 140 | 49 | 6.305 | 1 |
| 1734 | 91 | 20 | 1 | 140 | 49 | 6.306 | 1 |
| 1706 | 91 | 20 | 1 | 140 | 49 | 6.307 | 1 |
| 1701 | 91 | 20 | 1 | 140 | 49 | 6.308 | 1 |
| 1700 | 91 | 20 | 1 | 140 | 49 | 6.309 | 1 |
| 1195 | 91 | 20 | 1 | 140 | 49 | 6.31 | 1 |
| 1498 | 97 | 24 | 1 | 190 | 49 | 6.31 | 1 |
| 1533 | 98 | 25 | 1 | 200 | 49 | 6.31 | 1 |
| 1389 | 98 | 25 | 1 | 200 | 42 | 6.31 | 1 |
| 1211 | 90 | 20 | 1 | 140 | 49 | 6.311 | 1 |
| 1208 | 90 | 20 | 1 | 140 | 49 | 6.312 | 1 |
| 1202 | 90 | 20 | 1 | 140 | 49 | 6.313 | 1 |
| 1183 | 90 | 20 | 1 | 140 | 49 | 6.314 | 1 |
| 1182 | 90 | 20 | 1 | 140 | 49 | 6.315 | 1 |
| 1179 | 90 | 20 | 1 | 140 | 49 | 6.316 | 1 |
| 3501 | 91 | 20 | 1 | 140 | 49 | 6.317 | 1 |
| 3524 | 91 | 20 | 1 | 140 | 49 | 6.318 | 1 |
| 3500 | 91 | 20 | 1 | 140 | 49 | 6.319 | 1 |
| 1497 | 97 | 24 | 1 | 190 | 49 | 6.32 | 1 |

| | | | | | | | |
|---|---|---|---|---|---|---|---|
| 1495 | 97 | 24 | 1 | 190 | 49 | 6.33 | 1 |
| 1494 | 97 | 24 | 1 | 190 | 49 | 6.34 | 1 |
| 1493 | 97 | 24 | 1 | 190 | 49 | 6.35 | 1 |
| 1492 | 97 | 24 | 1 | 190 | 49 | 6.36 | 1 |
| 1530 | 98 | 25 | 1 | 200 | 43 | 6.365 | 1 |
| 1744 | 79 | 26 | 1 | 200 | 43 | 6.365 | 1 |
| 1491 | 97 | 24 | 1 | 190 | 43 | 6.37 | 1 |
| 1488 | 97 | 24 | 1 | 190 | 43 | 6.38 | 1 |
| 1238 | 97 | 24 | 1 | 190 | 49 | 6.39 | 1 |
| 1058 | 96 | 24 | 1 | 190 | 51 | 6.4 | 1 |
| 1057 | 96 | 24 | 1 | 190 | 51 | 6.41 | 1 |
| 1056 | 96 | 24 | 1 | 190 | 51 | 6.42 | 1 |
| 1517 | 98 | 25 | 1 | 200 | 43 | 6.42 | 1 |
| 1739 | 79 | 26 | 1 | 200 | 43 | 6.42 | 1 |
| 1051 | 96 | 24 | 1 | 190 | 51 | 6.43 | 1 |
| 1780 | 93 | 21 | 1 | 145 | 43 | 6.43 | 1 |
| 1779 | 93 | 21 | 1 | 145 | 43 | 6.431 | 1 |
| 1764 | 93 | 21 | 1 | 145 | 43 | 6.432 | 1 |
| 1671 | 93 | 21 | 1 | 145 | 43 | 6.433 | 1 |
| 1635 | 93 | 21 | 1 | 145 | 43 | 6.434 | 1 |
| 1610 | 93 | 21 | 1 | 145 | 43 | 6.435 | 1 |
| 1609 | 93 | 21 | 1 | 145 | 43 | 6.436 | 1 |
| 1608 | 93 | 21 | 1 | 145 | 43 | 6.437 | 1 |
| 1592 | 93 | 21 | 1 | 145 | 43 | 6.438 | 1 |
| 1585 | 93 | 21 | 1 | 145 | 43 | 6.439 | 1 |
| 1050 | 96 | 24 | 1 | 190 | 51 | 6.44 | 1 |
| 1583 | 93 | 21 | 1 | 145 | 43 | 6.44 | 1 |
| 1578 | 93 | 21 | 1 | 145 | 43 | 6.441 | 1 |
| 1575 | 93 | 21 | 1 | 145 | 43 | 6.442 | 1 |
| 1574 | 93 | 21 | 1 | 145 | 43 | 6.443 | 1 |
| 1034 | 92 | 21 | 1 | 145 | 51 | 6.444 | 1 |
| 1800 | 92 | 21 | 1 | 145 | 43 | 6.445 | 1 |
| 1799 | 92 | 21 | 1 | 145 | 43 | 6.446 | 1 |
| 1732 | 92 | 21 | 1 | 145 | 43 | 6.447 | 1 |
| 1731 | 92 | 21 | 1 | 145 | 43 | 6.448 | 0 |
| 1730 | 92 | 21 | 1 | 145 | 43 | 6.449 | 0 |
| 1041 | 96 | 24 | 1 | 190 | 51 | 6.45 | 0 |
| 1363 | 92 | 21 | 1 | 145 | 43 | 6.45 | 0 |
| 1347 | 92 | 21 | 1 | 145 | 43 | 6.451 | 0 |
| 1328 | 92 | 21 | 1 | 145 | 43 | 6.452 | 0 |
| 1267 | 92 | 21 | 1 | 145 | 43 | 6.453 | 0 |
| 1261 | 92 | 21 | 1 | 145 | 43 | 6.454 | 0 |
| 1191 | 92 | 21 | 1 | 145 | 49 | 6.455 | 0 |
| 1176 | 92 | 21 | 1 | 145 | 49 | 6.456 | 0 |

| | | | | | | | |
|---|---|---|---|---|---|---|---|
| 1166 | 92 | 21 | 1 | 145 | 49 | 6.457 | 0 |
| 1138 | 92 | 21 | 1 | 145 | 50 | 6.458 | 0 |
| 1137 | 92 | 21 | 1 | 145 | 50 | 6.459 | 0 |
| 1040 | 96 | 24 | 1 | 190 | 51 | 6.46 | 1 |
| 1135 | 92 | 21 | 1 | 145 | 50 | 6.46 | 0 |
| 1134 | 92 | 21 | 1 | 145 | 50 | 6.461 | 1 |
| 3495 | 92 | 21 | 1 | 145 | 42 | 6.462 | 0 |
| 3541 | 92 | 21 | 1 | 145 | 42 | 6.463 | 0 |
| 1039 | 96 | 24 | 1 | 190 | 51 | 6.47 | 0 |
| 1272 | 105 | 26 | 1 | 200 | 43 | 6.475 | 0 |
| 1516 | 98 | 25 | 1 | 200 | 42 | 6.475 | 0 |
| 1038 | 96 | 24 | 1 | 190 | 51 | 6.48 | 1 |
| 1033 | 96 | 24 | 1 | 190 | 51 | 6.49 | 0 |
| 1474 | 96 | 24 | 1 | 190 | 42 | 6.5 | 1 |
| 1473 | 96 | 24 | 1 | 190 | 42 | 6.51 | 1 |
| 1443 | 96 | 24 | 1 | 190 | 42 | 6.52 | 1 |
| 1388 | 96 | 24 | 1 | 190 | 42 | 6.53 | 0 |
| 1515 | 98 | 25 | 1 | 200 | 42 | 6.53 | 0 |
| 1408 | 104 | 26 | 1 | 200 | 42 | 6.53 | 1 |
| 1326 | 96 | 24 | 1 | 190 | 43 | 6.54 | 0 |
| 1325 | 96 | 24 | 1 | 190 | 43 | 6.55 | 0 |
| 1694 | 94 | 22 | 1 | 155 | 60 | 6.556 | 1 |
| 1321 | 96 | 24 | 1 | 190 | 43 | 6.56 | 0 |
| 1316 | 96 | 24 | 1 | 190 | 43 | 6.57 | 1 |
| 1273 | 96 | 24 | 1 | 190 | 43 | 6.58 | 1 |
| 1514 | 98 | 25 | 1 | 200 | 52 | 6.585 | 0 |
| 1407 | 104 | 26 | 1 | 200 | 42 | 6.585 | 0 |
| 1225 | 96 | 24 | 1 | 190 | 49 | 6.59 | 0 |
| 1193 | 96 | 24 | 1 | 190 | 49 | 6.6 | 0 |
| 1187 | 96 | 24 | 1 | 190 | 49 | 6.61 | 0 |
| 1172 | 96 | 24 | 1 | 190 | 49 | 6.62 | 1 |
| 1114 | 96 | 24 | 1 | 190 | 50 | 6.63 | 0 |
| 1524 | 93 | 22 | 1 | 170 | 64 | 6.635 | 0 |
| 1519 | 93 | 22 | 1 | 170 | 64 | 6.636 | 0 |
| 1518 | 93 | 22 | 1 | 170 | 64 | 6.637 | 0 |
| 1511 | 93 | 22 | 1 | 170 | 64 | 6.638 | 0 |
| 1510 | 93 | 22 | 1 | 170 | 64 | 6.639 | 0 |
| 1504 | 93 | 22 | 1 | 170 | 64 | 6.64 | 0 |
| 1513 | 98 | 25 | 1 | 200 | 52 | 6.64 | 0 |
| 1064 | 96 | 24 | 1 | 190 | 51 | 6.64 | 0 |
| 1397 | 104 | 26 | 1 | 200 | 42 | 6.64 | 0 |
| 1503 | 93 | 22 | 1 | 170 | 64 | 6.641 | 0 |
| 1502 | 93 | 22 | 1 | 170 | 64 | 6.642 | 0 |
| 1500 | 93 | 22 | 1 | 170 | 64 | 6.643 | 0 |

| | | | | | | | |
|---|---|---|---|---|---|---|---|
| 1484 | 93 | 22 | 1 | 170 | 64 | 6.644 | 0 |
| 1476 | 93 | 22 | 1 | 170 | 42 | 6.645 | 0 |
| 1469 | 93 | 22 | 1 | 170 | 42 | 6.646 | 0 |
| 1468 | 93 | 22 | 1 | 170 | 42 | 6.647 | 0 |
| 1467 | 93 | 22 | 1 | 170 | 42 | 6.648 | 0 |
| 1442 | 93 | 22 | 1 | 170 | 42 | 6.649 | 1 |
| 1047 | 96 | 24 | 1 | 190 | 51 | 6.65 | 1 |
| 1422 | 93 | 22 | 1 | 170 | 42 | 6.65 | 1 |
| 1385 | 96 | 24 | 1 | 190 | 42 | 6.66 | 0 |
| 1103 | 96 | 24 | 1 | 190 | 50 | 6.67 | 0 |
| 1102 | 96 | 24 | 1 | 190 | 50 | 6.68 | 0 |
| 1101 | 96 | 24 | 1 | 190 | 50 | 6.69 | 0 |
| 1218 | 98 | 25 | 1 | 200 | 49 | 6.695 | 1 |
| 1396 | 104 | 26 | 1 | 200 | 42 | 6.695 | 1 |
| 1099 | 96 | 24 | 1 | 190 | 50 | 6.7 | 0 |
| 1097 | 96 | 24 | 1 | 190 | 50 | 6.71 | 0 |
| 1096 | 96 | 24 | 1 | 190 | 50 | 6.72 | 0 |
| 1095 | 96 | 24 | 1 | 190 | 50 | 6.73 | 0 |
| 1094 | 96 | 24 | 1 | 190 | 50 | 6.74 | 1 |
| 1093 | 96 | 24 | 1 | 190 | 50 | 6.75 | 1 |
| 1428 | 98 | 25 | 1 | 200 | 42 | 6.75 | 0 |
| 1395 | 104 | 26 | 1 | 200 | 42 | 6.75 | 0 |
| 1269 | 93 | 22 | 1 | 175 | 43 | 6.752 | 0 |
| 1268 | 93 | 22 | 1 | 175 | 43 | 6.753 | 0 |
| 1266 | 93 | 22 | 1 | 175 | 43 | 6.754 | 0 |
| 1197 | 93 | 22 | 1 | 175 | 49 | 6.755 | 0 |
| 1711 | 93 | 22 | 1 | 175 | 63 | 6.756 | 1 |
| 1570 | 93 | 22 | 1 | 175 | 63 | 6.757 | 0 |
| 1569 | 93 | 22 | 1 | 175 | 63 | 6.758 | 1 |
| 1563 | 93 | 22 | 1 | 175 | 63 | 6.759 | 1 |
| 1561 | 93 | 22 | 1 | 175 | 63 | 6.76 | 1 |
| 1092 | 96 | 24 | 1 | 190 | 50 | 6.76 | 0 |
| 1560 | 93 | 22 | 1 | 175 | 63 | 6.761 | 0 |
| 1559 | 93 | 22 | 1 | 175 | 63 | 6.762 | 1 |
| 1558 | 93 | 22 | 1 | 175 | 63 | 6.763 | 1 |
| 1555 | 93 | 22 | 1 | 175 | 63 | 6.764 | 1 |
| 1550 | 93 | 22 | 1 | 175 | 63 | 6.765 | 1 |
| 1548 | 93 | 22 | 1 | 175 | 63 | 6.766 | 1 |
| 1545 | 93 | 22 | 1 | 175 | 63 | 6.767 | 0 |
| 1544 | 93 | 22 | 1 | 175 | 63 | 6.768 | 1 |
| 1543 | 93 | 22 | 1 | 175 | 63 | 6.769 | 1 |
| 1534 | 93 | 22 | 1 | 175 | 63 | 6.77 | 1 |
| 1091 | 96 | 24 | 1 | 190 | 50 | 6.77 | 0 |
| 1527 | 93 | 22 | 1 | 175 | 63 | 6.771 | 0 |

| | | | | | | | |
|---|---|---|---|---|---|---|---|
| 1526 | 93 | 22 | 1 | 175 | 63 | 6.772 | 1 |
| 1710 | 93 | 22 | 1 | 175 | 62 | 6.773 | 1 |
| 1145 | 96 | 23 | 1 | 175 | 50 | 6.774 | 1 |
| 1141 | 96 | 23 | 1 | 175 | 50 | 6.775 | 1 |
| 1139 | 96 | 23 | 1 | 175 | 50 | 6.776 | 0 |
| 1131 | 96 | 23 | 1 | 175 | 50 | 6.777 | 0 |
| 1090 | 96 | 24 | 1 | 190 | 50 | 6.78 | 0 |
| 1089 | 96 | 24 | 1 | 190 | 50 | 6.79 | 0 |
| 1088 | 96 | 24 | 1 | 190 | 50 | 6.8 | 0 |
| 1427 | 98 | 25 | 1 | 200 | 42 | 6.805 | 0 |
| 1394 | 104 | 26 | 1 | 200 | 42 | 6.805 | 1 |
| 1086 | 96 | 24 | 1 | 190 | 50 | 6.81 | 0 |
| 1079 | 96 | 24 | 1 | 190 | 51 | 6.82 | 1 |
| 1593 | 96 | 24 | 1 | 190 | 56 | 6.83 | 1 |
| 1571 | 96 | 24 | 1 | 190 | 56 | 6.84 | 0 |
| 1564 | 96 | 24 | 1 | 190 | 56 | 6.85 | 0 |
| 1562 | 96 | 24 | 1 | 190 | 56 | 6.86 | 0 |
| 1420 | 98 | 25 | 1 | 200 | 42 | 6.86 | 0 |
| 1393 | 104 | 26 | 1 | 200 | 42 | 6.86 | 0 |
| 1556 | 96 | 24 | 1 | 190 | 56 | 6.87 | 0 |
| 1546 | 96 | 24 | 1 | 190 | 56 | 6.88 | 0 |
| 1536 | 96 | 24 | 1 | 190 | 56 | 6.89 | 0 |
| 1535 | 96 | 24 | 1 | 190 | 56 | 6.9 | 0 |
| 1418 | 98 | 25 | 1 | 200 | 42 | 6.9 | 0 |
| 1392 | 104 | 26 | 1 | 200 | 42 | 6.9 | 0 |
| 1130 | 96 | 23 | 1 | 180 | 50 | 6.903 | 0 |
| 1126 | 96 | 23 | 1 | 180 | 50 | 6.904 | 1 |
| 1119 | 96 | 23 | 1 | 180 | 50 | 6.905 | 0 |
| 1117 | 96 | 23 | 1 | 180 | 50 | 6.906 | 1 |
| 1116 | 96 | 23 | 1 | 180 | 50 | 6.907 | 0 |
| 1110 | 96 | 23 | 1 | 180 | 50 | 6.908 | 0 |
| 1109 | 96 | 23 | 1 | 180 | 50 | 6.909 | 0 |
| 1529 | 96 | 24 | 1 | 190 | 56 | 6.91 | 0 |
| 1107 | 96 | 23 | 1 | 180 | 50 | 6.91 | 0 |
| 1105 | 96 | 23 | 1 | 180 | 50 | 6.911 | 0 |
| 1104 | 96 | 23 | 1 | 180 | 50 | 6.912 | 0 |
| 1693 | 95 | 23 | 1 | 180 | 59 | 6.913 | 0 |
| 1645 | 95 | 23 | 1 | 180 | 58 | 6.914 | 0 |
| 1032 | 95 | 23 | 1 | 180 | 51 | 6.915 | 0 |
| 1030 | 95 | 23 | 1 | 180 | 51 | 6.916 | 0 |
| 1018 | 95 | 23 | 1 | 180 | 51 | 6.917 | 1 |
| 1017 | 95 | 23 | 1 | 180 | 51 | 6.918 | 0 |
| 1016 | 95 | 23 | 1 | 180 | 51 | 6.919 | 0 |
| 1522 | 96 | 24 | 1 | 190 | 56 | 6.92 | 0 |

| | | | | | | | |
|---|---|---|---|---|---|---|---|
| 1013 | 95 | 23 | 1 | 180 | 51 | 6.92 | 0 |
| 1012 | 95 | 23 | 1 | 180 | 51 | 6.921 | 0 |
| 1010 | 95 | 23 | 1 | 180 | 51 | 6.922 | 0 |
| 1521 | 96 | 24 | 1 | 190 | 56 | 6.93 | 0 |
| 1520 | 96 | 24 | 1 | 190 | 56 | 6.94 | 0 |
| 1490 | 96 | 24 | 1 | 190 | 56 | 6.95 | 1 |
| 1485 | 96 | 24 | 1 | 190 | 56 | 6.96 | 0 |
| 1478 | 96 | 24 | 1 | 190 | 42 | 6.97 | 0 |
| 1477 | 96 | 24 | 1 | 190 | 42 | 6.98 | 1 |
| 1383 | 96 | 24 | 1 | 190 | 42 | 6.99 | 1 |
| 1133 | 100 | 25 | 1 | 190 | 50 | 7 | 0 |
| 1417 | 98 | 25 | 1 | 200 | 42 | 7 | 1 |
| 1390 | 104 | 26 | 1 | 200 | 42 | 7 | 0 |
| 1132 | 100 | 25 | 1 | 190 | 50 | 7.01 | 1 |
| 1416 | 98 | 25 | 1 | 200 | 42 | 7.01 | 1 |
| 1381 | 104 | 26 | 1 | 200 | 42 | 7.01 | 1 |
| 1111 | 100 | 25 | 1 | 190 | 50 | 7.02 | 1 |
| 1069 | 100 | 25 | 1 | 190 | 51 | 7.03 | 0 |
| 1028 | 100 | 25 | 1 | 190 | 51 | 7.04 | 0 |
| 1024 | 100 | 25 | 1 | 190 | 51 | 7.05 | 0 |
| 1035 | 95 | 23 | 1 | 185 | 51 | 7.053 | 1 |
| 1026 | 95 | 23 | 1 | 185 | 51 | 7.054 | 0 |
| 1025 | 95 | 23 | 1 | 185 | 51 | 7.055 | 1 |
| 1633 | 95 | 23 | 1 | 185 | 57 | 7.056 | 0 |
| 1078 | 95 | 23 | 1 | 185 | 51 | 7.057 | 1 |
| 1071 | 95 | 23 | 1 | 185 | 51 | 7.058 | 0 |
| 1062 | 95 | 23 | 1 | 185 | 51 | 7.059 | 1 |
| 1061 | 95 | 23 | 1 | 185 | 51 | 7.06 | 1 |
| 1023 | 100 | 25 | 1 | 190 | 51 | 7.06 | 1 |
| 1060 | 95 | 23 | 1 | 185 | 51 | 7.061 | 1 |
| 1059 | 95 | 23 | 1 | 185 | 51 | 7.062 | 1 |
| 1702 | 94 | 23 | 1 | 185 | 61 | 7.063 | 1 |
| 1597 | 98 | 24 | 1 | 185 | 51 | 7.064 | 1 |
| 1596 | 98 | 24 | 1 | 185 | 51 | 7.065 | 1 |
| 1586 | 98 | 24 | 1 | 185 | 51 | 7.066 | 1 |
| 1581 | 98 | 24 | 1 | 185 | 51 | 7.067 | 1 |
| 1580 | 98 | 24 | 1 | 185 | 51 | 7.068 | 0 |
| 1579 | 98 | 24 | 1 | 185 | 51 | 7.069 | 1 |
| 1577 | 98 | 24 | 1 | 185 | 51 | 7.07 | 1 |
| 1699 | 100 | 25 | 1 | 190 | 41 | 7.07 | 0 |
| 1576 | 98 | 24 | 1 | 185 | 51 | 7.071 | 1 |
| 1572 | 98 | 24 | 1 | 185 | 51 | 7.072 | 0 |
| 1565 | 98 | 24 | 1 | 185 | 51 | 7.073 | 0 |
| 1557 | 98 | 24 | 1 | 185 | 51 | 7.074 | 0 |

| | | | | | | | |
|---|---|---|---|---|---|---|---|
| 1554 | 98 | 24 | 1 | 185 | 51 | 7.075 | 0 |
| 1553 | 98 | 24 | 1 | 185 | 51 | 7.076 | 0 |
| 1552 | 98 | 24 | 1 | 185 | 51 | 7.077 | 0 |
| 1217 | 98 | 24 | 1 | 185 | 49 | 7.078 | 0 |
| 1456 | 98 | 24 | 1 | 185 | 42 | 7.079 | 0 |
| 1455 | 98 | 24 | 1 | 185 | 42 | 7.08 | 0 |
| 1698 | 100 | 25 | 1 | 190 | 41 | 7.08 | 0 |
| 1453 | 98 | 24 | 1 | 185 | 42 | 7.081 | 0 |
| 1451 | 98 | 24 | 1 | 185 | 42 | 7.082 | 0 |
| 1449 | 98 | 24 | 1 | 185 | 42 | 7.083 | 0 |
| 1448 | 98 | 24 | 1 | 185 | 42 | 7.084 | 0 |
| 1447 | 98 | 24 | 1 | 185 | 42 | 7.085 | 0 |
| 1445 | 98 | 24 | 1 | 185 | 42 | 7.086 | 0 |
| 1440 | 98 | 24 | 1 | 185 | 42 | 7.087 | 1 |
| 1439 | 98 | 24 | 1 | 185 | 42 | 7.088 | 0 |
| 1432 | 98 | 24 | 1 | 185 | 42 | 7.089 | 0 |
| 1431 | 98 | 24 | 1 | 185 | 42 | 7.09 | 0 |
| 1682 | 100 | 25 | 1 | 190 | 41 | 7.09 | 0 |
| 1430 | 98 | 24 | 1 | 185 | 42 | 7.091 | 0 |
| 1429 | 98 | 24 | 1 | 185 | 42 | 7.092 | 0 |
| 1672 | 100 | 25 | 1 | 190 | 41 | 7.1 | 0 |
| 1765 | 99 | 25 | 1 | 190 | 50 | 7.11 | 0 |
| 1743 | 99 | 25 | 1 | 190 | 50 | 7.12 | 1 |
| 1613 | 99 | 25 | 1 | 190 | 50 | 7.13 | 1 |
| 1606 | 99 | 25 | 1 | 190 | 50 | 7.14 | 1 |
| 1605 | 99 | 25 | 1 | 190 | 50 | 7.15 | 0 |
| 1604 | 99 | 25 | 1 | 190 | 50 | 7.16 | 0 |
| 1603 | 99 | 25 | 1 | 190 | 50 | 7.17 | 0 |
| 1602 | 99 | 25 | 1 | 190 | 50 | 7.18 | 0 |
| 1601 | 99 | 25 | 1 | 190 | 50 | 7.19 | 0 |
| 1600 | 99 | 25 | 1 | 190 | 50 | 7.2 | 1 |
| 1599 | 99 | 25 | 1 | 190 | 50 | 7.21 | 1 |
| 1598 | 99 | 25 | 1 | 190 | 50 | 7.22 | 1 |
| 1203 | 99 | 25 | 1 | 190 | 49 | 7.23 | 0 |
| 1525 | 99 | 25 | 1 | 190 | 49 | 7.24 | 0 |
| 1501 | 99 | 25 | 1 | 190 | 49 | 7.26 | 0 |
| 1489 | 99 | 25 | 1 | 190 | 49 | 7.27 | 0 |
| 1472 | 99 | 25 | 1 | 190 | 42 | 7.28 | 0 |
| 1464 | 99 | 25 | 1 | 190 | 42 | 7.3 | 0 |
| 1463 | 99 | 25 | 1 | 190 | 42 | 7.31 | 0 |
| 1462 | 99 | 25 | 1 | 190 | 42 | 7.32 | 0 |
| 1646 | 103 | 26 | 1 | 200 | 42 | 7.32365 | 0 |
| 1461 | 99 | 25 | 1 | 190 | 42 | 7.33 | 0 |
| 1649 | 103 | 26 | 1 | 200 | 42 | 7.33155 | 0 |

| | | | | | | | |
|---|---|---|---|---|---|---|---|
| 1650 | 103 | 26 | 1 | 200 | 42 | 7.3355 | 0 |
| 1651 | 103 | 26 | 1 | 200 | 42 | 7.33945 | 0 |
| 1460 | 99 | 25 | 1 | 190 | 42 | 7.34 | 0 |
| 1654 | 103 | 26 | 1 | 200 | 42 | 7.3434 | 0 |
| 1661 | 103 | 26 | 1 | 200 | 42 | 7.34735 | 0 |
| 1666 | 103 | 26 | 1 | 200 | 42 | 7.3592 | 0 |
| 1458 | 99 | 25 | 1 | 190 | 42 | 7.36 | 0 |
| 1667 | 103 | 26 | 1 | 200 | 42 | 7.36315 | 0 |
| 1669 | 103 | 26 | 1 | 200 | 42 | 7.3671 | 0 |
| 1457 | 99 | 25 | 1 | 190 | 42 | 7.37 | 0 |
| 1673 | 103 | 26 | 1 | 200 | 42 | 7.37105 | 0 |
| 1675 | 103 | 26 | 1 | 200 | 42 | 7.37895 | 0 |
| 1797 | 99 | 25 | 1 | 190 | 43 | 7.38 | 0 |
| 1676 | 103 | 26 | 1 | 200 | 42 | 7.3829 | 0 |
| 1678 | 103 | 26 | 1 | 200 | 42 | 7.38685 | 0 |
| 1679 | 103 | 26 | 1 | 200 | 42 | 7.3908 | 0 |
| 1681 | 103 | 26 | 1 | 200 | 42 | 7.3987 | 0 |
| 1664 | 99 | 25 | 1 | 190 | 43 | 7.4 | 0 |
| 1683 | 103 | 26 | 1 | 200 | 42 | 7.40265 | 0 |
| 1042 | 99 | 25 | 1 | 190 | 51 | 7.42 | 0 |
| 1716 | 103 | 26 | 1 | 200 | 42 | 7.42635 | 0 |
| 1638 | 99 | 25 | 1 | 190 | 43 | 7.43 | 0 |
| 1719 | 103 | 26 | 1 | 200 | 42 | 7.43425 | 0 |
| 1720 | 103 | 26 | 1 | 200 | 42 | 7.4382 | 0 |
| 1634 | 99 | 25 | 1 | 190 | 43 | 7.45 | 0 |
| 1724 | 103 | 26 | 1 | 200 | 42 | 7.45005 | 0 |
| 1021 | 104 | 26 | 1 | 200 | 51 | 7.46585 | 0 |
| 1067 | 104 | 26 | 1 | 200 | 51 | 7.47375 | 0 |
| 1083 | 104 | 26 | 1 | 200 | 50 | 7.4777 | 0 |
| 1591 | 99 | 25 | 1 | 190 | 43 | 7.48 | 0 |
| 1115 | 104 | 26 | 1 | 200 | 50 | 7.48165 | 0 |
| 1122 | 104 | 26 | 1 | 200 | 50 | 7.4856 | 0 |
| 1128 | 104 | 26 | 1 | 200 | 50 | 7.48955 | 0 |
| 1590 | 99 | 25 | 1 | 190 | 43 | 7.49 | 0 |
| 1796 | 97 | 24 | 1 | 190 | 59 | 7.494 | 0 |
| 1789 | 97 | 24 | 1 | 190 | 59 | 7.496 | 0 |
| 1769 | 97 | 24 | 1 | 190 | 59 | 7.497 | 0 |
| 1206 | 104 | 26 | 1 | 200 | 59 | 7.49745 | 0 |
| 1612 | 97 | 24 | 1 | 190 | 59 | 7.498 | 0 |
| 1611 | 97 | 24 | 1 | 190 | 59 | 7.499 | 0 |
| 1595 | 97 | 24 | 1 | 190 | 59 | 7.5 | 0 |
| 1589 | 99 | 25 | 1 | 190 | 59 | 7.5 | 0 |
| 1594 | 97 | 24 | 1 | 190 | 59 | 7.501 | 0 |
| 1207 | 104 | 26 | 1 | 200 | 59 | 7.5014 | 0 |

| | | | | | | | |
|---|---|---|---|---|---|---|---|
| 1136 | 97 | 24 | 1 | 190 | 59 | 7.504 | 0 |
| 1072 | 97 | 24 | 1 | 190 | 59 | 7.505 | 0 |
| 1252 | 104 | 26 | 1 | 200 | 59 | 7.50535 | 0 |
| 1070 | 97 | 24 | 1 | 190 | 59 | 7.506 | 0 |
| 1066 | 97 | 24 | 1 | 190 | 59 | 7.507 | 0 |
| 1063 | 97 | 24 | 1 | 190 | 59 | 7.508 | 0 |
| 1029 | 97 | 24 | 1 | 190 | 59 | 7.509 | 0 |
| 1270 | 104 | 26 | 1 | 200 | 59 | 7.5093 | 0 |
| 1027 | 97 | 24 | 1 | 190 | 59 | 7.51 | 0 |
| 1588 | 99 | 25 | 1 | 190 | 59 | 7.51 | 1 |
| 1022 | 97 | 24 | 1 | 190 | 59 | 7.511 | 0 |
| 1020 | 97 | 24 | 1 | 190 | 59 | 7.512 | 0 |
| 1185 | 97 | 24 | 1 | 190 | 59 | 7.514 | 0 |
| 1170 | 97 | 24 | 1 | 190 | 60 | 7.518 | 0 |
| 1475 | 97 | 24 | 1 | 190 | 60 | 7.535 | 0 |
| 1471 | 97 | 24 | 1 | 190 | 60 | 7.536 | 0 |
| 1470 | 97 | 24 | 1 | 190 | 60 | 7.537 | 1 |
| 1173 | 97 | 24 | 1 | 190 | 60 | 7.539 | 0 |
| 1582 | 99 | 25 | 1 | 190 | 60 | 7.54 | 0 |
| 1167 | 97 | 24 | 1 | 190 | 60 | 7.54 | 0 |
| 1143 | 97 | 24 | 1 | 190 | 60 | 7.541 | 0 |
| 1276 | 97 | 24 | 1 | 190 | 60 | 7.542 | 0 |
| 1235 | 97 | 24 | 1 | 190 | 60 | 7.548 | 0 |
| 1234 | 97 | 24 | 1 | 190 | 60 | 7.549 | 0 |
| 1573 | 99 | 25 | 1 | 190 | 60 | 7.55 | 0 |
| 1233 | 97 | 24 | 1 | 190 | 60 | 7.55 | 0 |
| 1220 | 97 | 24 | 1 | 190 | 60 | 7.551 | 0 |
| 1198 | 97 | 24 | 1 | 190 | 60 | 7.552 | 0 |
| 1192 | 97 | 24 | 1 | 190 | 60 | 7.553 | 0 |
| 1189 | 97 | 24 | 1 | 190 | 60 | 7.554 | 1 |
| 1188 | 97 | 24 | 1 | 190 | 60 | 7.555 | 0 |
| 1568 | 99 | 25 | 1 | 190 | 60 | 7.56 | 1 |
| 1567 | 99 | 25 | 1 | 190 | 60 | 7.57 | 0 |
| 1566 | 99 | 25 | 1 | 190 | 60 | 7.58 | 0 |
| 1551 | 99 | 25 | 1 | 190 | 60 | 7.59 | 0 |
| 1528 | 99 | 25 | 1 | 190 | 60 | 7.62 | 0 |
| 1382 | 98 | 25 | 1 | 190 | 60 | 7.63 | 0 |
| 1376 | 98 | 25 | 1 | 190 | 60 | 7.64 | 0 |
| 1324 | 98 | 25 | 1 | 190 | 60 | 7.65 | 1 |
| 1323 | 98 | 25 | 1 | 190 | 60 | 7.66 | 0 |
| 1322 | 98 | 25 | 1 | 190 | 60 | 7.67 | 0 |
| 1318 | 98 | 25 | 1 | 190 | 60 | 7.7 | 0 |
| 1317 | 98 | 25 | 1 | 190 | 60 | 7.71 | 0 |
| 1315 | 98 | 25 | 1 | 190 | 60 | 7.72 | 0 |

| | | | | | | | |
|---|---|---|---|---|---|---|---|
| 1271 | 98 | 25 | 1 | 190 | 60 | 7.74 | 0 |
| 1264 | 98 | 25 | 1 | 190 | 60 | 7.75 | 0 |
| 1773 | 89 | 29 | 1 | 205 | 61 | 8.9517 | 0 |
| 1737 | 89 | 29 | 1 | 205 | 61 | 8.952 | 0 |
| 1736 | 89 | 29 | 1 | 205 | 61 | 8.9521 | 0 |
| 1728 | 89 | 29 | 1 | 205 | 61 | 8.9522 | 0 |
| 1715 | 89 | 29 | 1 | 205 | 61 | 8.9523 | 0 |
| 1697 | 89 | 29 | 1 | 205 | 61 | 8.9528 | 0 |
| 1653 | 89 | 29 | 1 | 205 | 61 | 8.9529 | 0 |
| 1636 | 89 | 29 | 1 | 205 | 61 | 8.9531 | 0 |
| 1632 | 89 | 29 | 1 | 205 | 61 | 8.9532 | 0 |
| 1631 | 89 | 29 | 1 | 205 | 61 | 8.9533 | 0 |
| 1626 | 89 | 29 | 1 | 205 | 61 | 8.9534 | 0 |
| 1621 | 89 | 29 | 1 | 205 | 61 | 8.9535 | 0 |
| 1620 | 89 | 29 | 1 | 205 | 61 | 8.9536 | 0 |
| 1618 | 89 | 29 | 1 | 205 | 61 | 8.9537 | 0 |
| 1532 | 89 | 29 | 1 | 205 | 61 | 8.9538 | 0 |
| 1496 | 89 | 29 | 1 | 205 | 61 | 8.954 | 0 |
| 1466 | 89 | 29 | 1 | 205 | 61 | 8.9541 | 0 |
| 1454 | 89 | 29 | 1 | 205 | 61 | 8.9542 | 0 |
| 1437 | 89 | 29 | 1 | 205 | 61 | 8.9546 | 0 |
| 1435 | 89 | 29 | 1 | 205 | 62 | 8.9548 | 0 |
| 1433 | 89 | 29 | 1 | 205 | 62 | 8.9549 | 0 |
| 1413 | 89 | 29 | 1 | 205 | 62 | 8.955 | 0 |
| 1350 | 88 | 29 | 1 | 205 | 62 | 8.9553 | 0 |
| 1242 | 88 | 29 | 1 | 205 | 62 | 8.9554 | 0 |
| 1230 | 88 | 29 | 1 | 205 | 62 | 8.9555 | 0 |
| 1223 | 88 | 29 | 1 | 205 | 62 | 8.9556 | 0 |
| 1005 | 88 | 29 | 1 | 205 | 62 | 8.9557 | 0 |
| 1808 | 88 | 29 | 1 | 205 | 62 | 8.9558 | 0 |
| 1767 | 88 | 29 | 1 | 205 | 62 | 8.9559 | 0 |
| 1750 | 88 | 29 | 1 | 205 | 62 | 8.956 | 0 |
| 1712 | 88 | 29 | 1 | 205 | 62 | 8.9561 | 0 |
| 1670 | 88 | 29 | 1 | 205 | 62 | 8.9562 | 0 |
| 1658 | 88 | 29 | 1 | 205 | 62 | 8.9563 | 0 |
| 1656 | 88 | 29 | 1 | 205 | 62 | 8.9564 | 0 |
| 1426 | 88 | 29 | 1 | 205 | 62 | 8.9565 | 0 |
| 1424 | 88 | 29 | 1 | 205 | 62 | 8.9567 | 0 |
| 1404 | 88 | 29 | 1 | 205 | 62 | 8.9568 | 0 |
| 1344 | 87 | 29 | 1 | 205 | 62 | 8.9569 | 0 |
| 1311 | 87 | 29 | 1 | 205 | 62 | 8.957 | 0 |
| 1310 | 87 | 29 | 1 | 205 | 62 | 8.9571 | 0 |
| 1305 | 87 | 29 | 1 | 205 | 62 | 8.9572 | 0 |
| 1304 | 87 | 29 | 1 | 205 | 62 | 8.9573 | 0 |

| | | | | | | | |
|---|---|---|---|---|---|---|---|
| 1277 | 87 | 29 | 1 | 205 | 62 | 8.9574 | 0 |
| 1241 | 87 | 29 | 1 | 205 | 62 | 8.9575 | 0 |
| 1240 | 87 | 29 | 1 | 205 | 62 | 8.9576 | 0 |
| 1204 | 87 | 29 | 1 | 205 | 62 | 8.9577 | 0 |
| 1200 | 87 | 29 | 1 | 205 | 62 | 8.9578 | 0 |
| 1174 | 87 | 29 | 1 | 205 | 62 | 8.9579 | 0 |
| 1146 | 87 | 29 | 1 | 205 | 62 | 8.958 | 0 |
| 1004 | 87 | 29 | 1 | 205 | 62 | 8.9581 | 0 |
| 1624 | 87 | 29 | 1 | 205 | 62 | 8.9586 | 0 |
| 1619 | 87 | 29 | 1 | 205 | 62 | 8.9587 | 0 |
| 1423 | 87 | 29 | 1 | 205 | 62 | 8.9588 | 0 |
| 1368 | 87 | 29 | 1 | 205 | 62 | 8.9589 | 0 |
| 1359 | 87 | 29 | 1 | 205 | 62 | 8.959 | 0 |
| 1665 | 86 | 29 | 1 | 205 | 62 | 8.9591 | 0 |
| 1660 | 86 | 29 | 1 | 205 | 62 | 8.9592 | 0 |
| 1659 | 86 | 29 | 1 | 205 | 62 | 8.9593 | 0 |
| 1648 | 86 | 29 | 1 | 205 | 62 | 8.9594 | 0 |
| 1124 | 90 | 30 | 1 | 205 | 62 | 8.9597 | 0 |
| 1123 | 90 | 30 | 1 | 205 | 62 | 8.9598 | 0 |
| 1121 | 90 | 30 | 1 | 205 | 62 | 8.9599 | 0 |
| 1118 | 90 | 30 | 1 | 205 | 62 | 8.96 | 0 |
| 1085 | 90 | 30 | 1 | 205 | 62 | 8.9601 | 0 |
| 1080 | 90 | 30 | 1 | 205 | 62 | 8.9602 | 0 |
| 1077 | 90 | 30 | 1 | 205 | 62 | 8.9603 | 0 |
| 1076 | 90 | 30 | 1 | 205 | 62 | 8.9604 | 0 |
| 1074 | 90 | 30 | 1 | 205 | 62 | 8.9605 | 0 |
| 1055 | 90 | 30 | 1 | 205 | 62 | 8.9606 | 0 |
| 1031 | 90 | 30 | 1 | 205 | 62 | 8.9607 | 0 |
| 1007 | 90 | 30 | 1 | 205 | 62 | 8.9608 | 0 |
| 1006 | 90 | 30 | 1 | 205 | 62 | 8.9609 | 0 |
| 1372 | 89 | 30 | 1 | 205 | 62 | 8.961 | 0 |
| 1367 | 89 | 30 | 1 | 205 | 62 | 8.9611 | 0 |
| 1346 | 89 | 30 | 1 | 205 | 62 | 8.9614 | 0 |
| 1335 | 89 | 30 | 1 | 205 | 62 | 8.9615 | 0 |
| 1333 | 89 | 30 | 1 | 205 | 62 | 8.9617 | 0 |
| 1332 | 89 | 30 | 1 | 205 | 62 | 8.9618 | 0 |
| 1331 | 89 | 30 | 1 | 205 | 62 | 8.9619 | 0 |
| 1330 | 89 | 30 | 1 | 205 | 62 | 8.962 | 0 |
| 1306 | 89 | 30 | 1 | 205 | 62 | 8.9621 | 0 |
| 1296 | 89 | 30 | 1 | 205 | 62 | 8.9623 | 0 |
| 1278 | 89 | 30 | 1 | 205 | 62 | 8.9625 | 0 |
| 1243 | 89 | 30 | 1 | 205 | 62 | 8.9626 | 0 |
| 1239 | 89 | 30 | 1 | 205 | 62 | 8.9627 | 0 |
| 1232 | 89 | 30 | 1 | 205 | 62 | 8.9628 | 0 |

| | | | | | | | |
|---|---|---|---|---|---|---|---|
| 1231 | 89 | 30 | 1 | 205 | 62 | 8.9629 | 0 |
| 1219 | 89 | 30 | 1 | 205 | 62 | 8.963 | 0 |
| 1205 | 89 | 30 | 1 | 205 | 62 | 8.9631 | 0 |
| 1181 | 89 | 30 | 1 | 205 | 62 | 8.9632 | 0 |
| 1178 | 89 | 30 | 1 | 205 | 62 | 8.9633 | 0 |
| 1177 | 89 | 30 | 1 | 205 | 62 | 8.9634 | 0 |
| 1084 | 89 | 30 | 1 | 205 | 62 | 8.9635 | 0 |
| 1073 | 89 | 30 | 1 | 205 | 62 | 8.9636 | 0 |
| 1001 | 89 | 30 | 1 | 205 | 62 | 8.9638 | 0 |
| 1812 | 89 | 30 | 1 | 205 | 62 | 8.9639 | 0 |
| 1809 | 89 | 30 | 1 | 205 | 62 | 8.964 | 0 |
| 1762 | 89 | 30 | 1 | 205 | 62 | 8.9641 | 0 |
| 1370 | 89 | 30 | 1 | 205 | 62 | 8.9642 | 0 |
| 1369 | 89 | 30 | 1 | 205 | 62 | 8.9643 | 0 |
| 1361 | 89 | 30 | 1 | 205 | 63 | 8.9644 | 0 |
| 1253 | 89 | 30 | 1 | 205 | 63 | 8.9645 | 0 |
| 1201 | 89 | 30 | 1 | 205 | 63 | 8.9646 | 0 |
| 1152 | 89 | 30 | 1 | 205 | 63 | 8.9647 | 0 |
| 1129 | 89 | 30 | 1 | 205 | 63 | 8.9648 | 0 |
| 1120 | 89 | 30 | 1 | 205 | 63 | 8.9649 | 0 |
| 1075 | 89 | 30 | 1 | 205 | 63 | 8.965 | 0 |
| 1009 | 89 | 30 | 1 | 205 | 63 | 8.9651 | 0 |
| 1813 | 89 | 30 | 1 | 205 | 63 | 8.9652 | 0 |
| 1733 | 89 | 30 | 1 | 205 | 63 | 8.9654 | 0 |
| 1687 | 89 | 30 | 1 | 205 | 63 | 8.9655 | 0 |
| 1657 | 89 | 30 | 1 | 205 | 63 | 8.9656 | 0 |
| 1616 | 89 | 30 | 1 | 205 | 63 | 8.9658 | 0 |
| 1353 | 89 | 30 | 1 | 205 | 63 | 8.966 | 0 |
| 1351 | 89 | 30 | 1 | 205 | 63 | 8.9661 | 0 |
| 1340 | 89 | 30 | 1 | 205 | 63 | 8.9662 | 0 |
| 1312 | 89 | 30 | 1 | 205 | 63 | 8.9663 | 0 |
| 1301 | 89 | 30 | 1 | 205 | 63 | 8.9664 | 0 |
| 1300 | 89 | 30 | 1 | 205 | 63 | 8.9665 | 0 |
| 1289 | 89 | 30 | 1 | 205 | 63 | 8.9666 | 0 |
| 1287 | 89 | 30 | 1 | 205 | 63 | 8.9668 | 0 |
| 1275 | 89 | 30 | 1 | 205 | 63 | 8.967 | 0 |
| 1246 | 89 | 30 | 1 | 205 | 63 | 8.9671 | 0 |
| 1629 | 86 | 30 | 1 | 205 | 63 | 8.9674 | 0 |
| 1628 | 86 | 30 | 1 | 205 | 63 | 8.9675 | 0 |
| 1627 | 86 | 30 | 1 | 205 | 63 | 8.9676 | 0 |
| 1625 | 86 | 30 | 1 | 205 | 63 | 8.9677 | 0 |
| 1801 | 91 | 31 | 1 | 205 | 63 | 8.9679 | 0 |
| 1402 | 90 | 31 | 1 | 205 | 63 | 8.968 | 0 |
| 1398 | 90 | 31 | 1 | 205 | 63 | 8.9681 | 0 |

| | | | | | | | |
|---|---|---|---|---|---|---|---|
| 1384 | 90 | 31 | 1 | 205 | 63 | 8.9682 | 0 |
| 1378 | 90 | 31 | 1 | 205 | 63 | 8.9683 | 0 |
| 1374 | 90 | 31 | 1 | 205 | 63 | 8.9684 | 1 |
| 1357 | 90 | 31 | 1 | 205 | 63 | 8.9685 | 0 |
| 1355 | 90 | 31 | 1 | 205 | 63 | 8.9686 | 0 |
| 1354 | 90 | 31 | 1 | 205 | 63 | 8.9687 | 0 |
| 1352 | 90 | 31 | 1 | 205 | 63 | 8.9688 | 0 |
| 1348 | 90 | 31 | 1 | 205 | 63 | 8.9689 | 1 |
| 1345 | 90 | 31 | 1 | 205 | 63 | 8.969 | 0 |
| 1339 | 90 | 31 | 1 | 205 | 63 | 8.9693 | 1 |
| 1338 | 90 | 31 | 1 | 205 | 63 | 8.9694 | 1 |
| 1337 | 90 | 31 | 1 | 205 | 63 | 8.9695 | 0 |
| 1336 | 90 | 31 | 1 | 205 | 63 | 8.9696 | 0 |
| 1314 | 90 | 31 | 1 | 205 | 63 | 8.9698 | 0 |
| 1309 | 90 | 31 | 1 | 205 | 63 | 8.9699 | 0 |
| 1308 | 90 | 31 | 1 | 205 | 63 | 8.97 | 0 |
| 1307 | 90 | 31 | 1 | 205 | 63 | 8.9701 | 0 |
| 1303 | 90 | 31 | 1 | 205 | 63 | 8.9702 | 0 |
| 1299 | 90 | 31 | 1 | 205 | 63 | 8.9703 | 0 |
| 1294 | 90 | 31 | 1 | 205 | 63 | 8.9706 | 0 |
| 1283 | 90 | 31 | 1 | 205 | 63 | 8.9709 | 0 |
| 1281 | 90 | 31 | 1 | 205 | 63 | 8.971 | 0 |
| 1280 | 90 | 31 | 1 | 205 | 63 | 8.9711 | 0 |
| 1256 | 90 | 31 | 1 | 205 | 63 | 8.9712 | 0 |
| 1254 | 90 | 31 | 1 | 205 | 63 | 8.9714 | 0 |
| 1251 | 90 | 31 | 1 | 205 | 63 | 8.9715 | 0 |
| 1250 | 90 | 31 | 1 | 205 | 63 | 8.9716 | 0 |
| 1249 | 90 | 31 | 1 | 205 | 63 | 8.9717 | 0 |
| 1248 | 90 | 31 | 1 | 205 | 63 | 8.9718 | 0 |
| 1224 | 90 | 31 | 1 | 205 | 63 | 8.9719 | 0 |
| 1222 | 90 | 31 | 1 | 205 | 63 | 8.972 | 0 |
| 1221 | 90 | 31 | 1 | 205 | 63 | 8.9721 | 0 |
| 1216 | 90 | 31 | 1 | 205 | 63 | 8.9722 | 0 |
| 1215 | 90 | 31 | 1 | 205 | 63 | 8.9723 | 0 |
| 1644 | 103 | 26 | 1 | 210 | 65 | 8.9878 | 0 |
| 1640 | 103 | 26 | 1 | 210 | 65 | 8.988 | 0 |
| 1803 | 102 | 26 | 1 | 210 | 65 | 8.9882 | 0 |
| 1802 | 102 | 26 | 1 | 210 | 65 | 8.9883 | 0 |
| 1783 | 102 | 26 | 1 | 210 | 65 | 8.9884 | 0 |
| 1782 | 102 | 26 | 1 | 210 | 65 | 8.9885 | 0 |
| 1771 | 102 | 26 | 1 | 210 | 65 | 8.9886 | 0 |
| 1758 | 102 | 26 | 1 | 210 | 65 | 8.9888 | 0 |
| 1742 | 102 | 26 | 1 | 210 | 65 | 8.9889 | 0 |
| 1723 | 102 | 26 | 1 | 210 | 65 | 8.989 | 0 |

| | | | | | | | |
|---|---|---|---|---|---|---|---|
| 1717 | 102 | 26 | 1 | 210 | 65 | 8.9891 | 0 |
| 1677 | 102 | 26 | 1 | 210 | 65 | 8.9894 | 0 |
| 1668 | 102 | 26 | 1 | 210 | 65 | 8.9895 | 0 |
| 1655 | 102 | 26 | 1 | 210 | 65 | 8.9896 | 0 |
| 1652 | 102 | 26 | 1 | 210 | 65 | 8.9897 | 0 |
| 1643 | 102 | 26 | 1 | 210 | 65 | 8.9898 | 0 |
| 1623 | 102 | 26 | 1 | 210 | 65 | 8.9899 | 0 |
| 1615 | 102 | 26 | 1 | 210 | 65 | 8.99 | 0 |
| 1614 | 102 | 26 | 1 | 210 | 65 | 8.9901 | 0 |
| 1444 | 102 | 26 | 1 | 210 | 65 | 8.9902 | 0 |
| 1414 | 102 | 26 | 1 | 210 | 65 | 8.9903 | 0 |
| 1412 | 102 | 26 | 1 | 210 | 65 | 8.9904 | 0 |
| 1387 | 102 | 26 | 1 | 210 | 65 | 8.9905 | 0 |
| 1379 | 102 | 26 | 1 | 210 | 65 | 8.9906 | 0 |
| 1366 | 102 | 26 | 1 | 210 | 65 | 8.9908 | 0 |
| 1285 | 102 | 26 | 1 | 210 | 66 | 8.9912 | 0 |
| 1793 | 80 | 27 | 1 | 210 | 67 | 8.9933 | 0 |
| 1792 | 80 | 27 | 1 | 210 | 67 | 8.9934 | 0 |
| 1790 | 80 | 27 | 1 | 210 | 67 | 8.9935 | 0 |
| 1787 | 80 | 27 | 1 | 210 | 67 | 8.9937 | 0 |
| 1786 | 80 | 27 | 1 | 210 | 67 | 8.9938 | 0 |
| 1785 | 80 | 27 | 1 | 210 | 67 | 8.9939 | 0 |
| 1777 | 80 | 27 | 1 | 210 | 67 | 8.9941 | 0 |
| 1776 | 80 | 27 | 1 | 210 | 67 | 8.9942 | 0 |
| 1757 | 79 | 27 | 1 | 210 | 68 | 8.9948 | 0 |
| 1755 | 79 | 27 | 1 | 210 | 68 | 8.9949 | 0 |
| 1754 | 79 | 27 | 1 | 210 | 68 | 8.995 | 0 |
| 1753 | 79 | 27 | 1 | 210 | 68 | 8.9951 | 0 |
| 1752 | 79 | 27 | 1 | 210 | 68 | 8.9952 | 0 |
| 1751 | 79 | 27 | 1 | 210 | 68 | 8.9953 | 0 |
| 1749 | 79 | 27 | 1 | 210 | 68 | 8.9954 | 0 |
| 1748 | 79 | 27 | 1 | 210 | 68 | 8.9955 | 0 |
| 1747 | 79 | 27 | 1 | 210 | 68 | 8.9956 | 0 |
| 1746 | 79 | 27 | 1 | 210 | 68 | 8.9957 | 0 |
| 1745 | 79 | 27 | 1 | 210 | 68 | 8.9958 | 0 |
| 1805 | 86 | 28 | 1 | 210 | 68 | 8.9959 | 0 |
| 1804 | 86 | 28 | 1 | 210 | 69 | 8.996 | 0 |
| 1727 | 86 | 28 | 1 | 210 | 69 | 8.9961 | 0 |
| 1726 | 86 | 28 | 1 | 210 | 69 | 8.9962 | 0 |
| 1714 | 86 | 28 | 1 | 210 | 69 | 8.9963 | 0 |
| 1695 | 86 | 28 | 1 | 210 | 69 | 8.9964 | 0 |
| 1349 | 86 | 28 | 1 | 210 | 69 | 8.9969 | 0 |
| 1781 | 83 | 28 | 1 | 210 | 69 | 8.997 | 0 |
| 1741 | 83 | 28 | 1 | 210 | 69 | 8.9971 | 0 |

| | | | | | | | |
|---|---|---|---|---|---|---|---|
| 1725 | 83 | 28 | 1 | 210 | 69 | 8.9972 | 0 |
| 1686 | 83 | 28 | 1 | 210 | 69 | 8.9973 | 0 |
| 1401 | 83 | 28 | 1 | 210 | 69 | 8.9974 | 0 |
| 1386 | 83 | 28 | 1 | 210 | 69 | 8.9975 | 0 |
| 1377 | 83 | 28 | 1 | 210 | 70 | 8.9976 | 0 |
| 1375 | 83 | 28 | 1 | 210 | 70 | 8.9977 | 0 |
| 1265 | 83 | 28 | 1 | 210 | 70 | 8.9978 | 0 |
| 1228 | 83 | 28 | 1 | 210 | 70 | 8.9979 | 0 |
| 1227 | 83 | 28 | 1 | 210 | 70 | 8.998 | 0 |
| 1194 | 83 | 28 | 1 | 210 | 70 | 8.9981 | 0 |
| 1155 | 83 | 28 | 1 | 210 | 70 | 8.9982 | 0 |
| 1108 | 83 | 28 | 1 | 210 | 70 | 8.9985 | 0 |
| 1106 | 83 | 28 | 1 | 210 | 70 | 8.9986 | 0 |
| 1081 | 83 | 28 | 1 | 210 | 70 | 8.9987 | 0 |
| 1065 | 82 | 28 | 1 | 210 | 70 | 8.9988 | 0 |
| 1049 | 82 | 28 | 1 | 210 | 70 | 8.9989 | 0 |
| 1244 | 89 | 29 | 1 | 210 | 70 | 8.999 | 0 |
| 1213 | 89 | 29 | 1 | 210 | 71 | 8.9992 | 0 |
| 1210 | 89 | 29 | 1 | 210 | 71 | 8.9994 | 0 |
| 1209 | 89 | 29 | 1 | 210 | 71 | 8.9995 | 0 |
| 1127 | 89 | 29 | 1 | 210 | 71 | 8.9997 | 0 |
| 1784 | 89 | 29 | 1 | 210 | 71 | 8.9999 | 0 |
| 1703 | 89 | 29 | 1 | 210 | 71 | 9 | 0 |
| 1691 | 89 | 29 | 1 | 210 | 71 | 9.0002 | 0 |
| 1421 | 89 | 29 | 1 | 210 | 71 | 9.0003 | 0 |
| 1419 | 89 | 29 | 1 | 210 | 71 | 9.0004 | 0 |
| 1341 | 89 | 29 | 1 | 210 | 71 | 9.0006 | 0 |
| 1313 | 89 | 29 | 1 | 210 | 71 | 9.0007 | 0 |
| 1292 | 89 | 29 | 1 | 210 | 72 | 9.0008 | 0 |
| 1279 | 89 | 29 | 1 | 210 | 72 | 9.0009 | 0 |
| 1247 | 89 | 29 | 1 | 210 | 73 | 9.001 | 0 |
| 1113 | 89 | 29 | 1 | 210 | 73 | 9.0011 | 0 |
| 1112 | 89 | 29 | 1 | 210 | 74 | 9.0012 | 0 |
| 1068 | 89 | 29 | 1 | 210 | 74 | 9.0013 | 0 |
| 1054 | 89 | 29 | 1 | 210 | 75 | 9.0014 | 0 |
| 1053 | 89 | 29 | 1 | 210 | 75 | 9.0015 | 0 |
| 1052 | 89 | 29 | 1 | 210 | 76 | 9.0016 | 0 |
| 1037 | 89 | 29 | 1 | 210 | 76 | 9.0017 | 0 |
| 1003 | 89 | 29 | 1 | 210 | 77 | 9.0018 | 0 |
| 1002 | 89 | 29 | 1 | 210 | 77 | 9.0019 | 0 |
| 2679 | 98 | 25 | 2 | 200 | 51 | 6.07 | 1 |
| 1904 | 92 | 20 | 2 | 120 | 51 | 6.071 | 1 |
| 1903 | 92 | 20 | 2 | 120 | 51 | 6.072 | 1 |
| 3110 | 91 | 20 | 2 | 120 | 51 | 6.073 | 1 |

| | | | | | | | |
|---|---|---|---|---|---|---|---|
| 3109 | 91 | 20 | 2 | 120 | 51 | 6.074 | 1 |
| 3108 | 91 | 20 | 2 | 120 | 51 | 6.075 | 1 |
| 3107 | 91 | 20 | 2 | 120 | 51 | 6.076 | 1 |
| 3106 | 91 | 20 | 2 | 120 | 51 | 6.077 | 1 |
| 3105 | 91 | 20 | 2 | 120 | 51 | 6.078 | 1 |
| 3104 | 91 | 20 | 2 | 120 | 51 | 6.079 | 1 |
| 3103 | 91 | 20 | 2 | 120 | 51 | 6.08 | 1 |
| 2990 | 105 | 26 | 2 | 200 | 51 | 6.08 | 1 |
| 3102 | 91 | 20 | 2 | 120 | 51 | 6.081 | 1 |
| 3101 | 91 | 20 | 2 | 120 | 51 | 6.082 | 1 |
| 3100 | 91 | 20 | 2 | 120 | 51 | 6.083 | 1 |
| 3099 | 91 | 20 | 2 | 120 | 51 | 6.084 | 1 |
| 3098 | 91 | 20 | 2 | 120 | 51 | 6.085 | 1 |
| 2883 | 91 | 20 | 2 | 125 | 51 | 6.086 | 1 |
| 2882 | 91 | 20 | 2 | 125 | 51 | 6.087 | 1 |
| 2881 | 91 | 20 | 2 | 125 | 51 | 6.088 | 1 |
| 2880 | 91 | 20 | 2 | 125 | 51 | 6.089 | 1 |
| 2879 | 91 | 20 | 2 | 125 | 51 | 6.09 | 1 |
| 2989 | 105 | 26 | 2 | 200 | 51 | 6.09 | 1 |
| 2878 | 91 | 20 | 2 | 125 | 51 | 6.091 | 1 |
| 2877 | 91 | 20 | 2 | 125 | 51 | 6.092 | 1 |
| 2876 | 91 | 20 | 2 | 125 | 51 | 6.093 | 1 |
| 2875 | 91 | 20 | 2 | 125 | 51 | 6.094 | 1 |
| 2874 | 91 | 20 | 2 | 125 | 51 | 6.095 | 1 |
| 2873 | 91 | 20 | 2 | 125 | 51 | 6.096 | 1 |
| 2872 | 91 | 20 | 2 | 125 | 51 | 6.097 | 1 |
| 2871 | 91 | 20 | 2 | 125 | 51 | 6.098 | 1 |
| 2870 | 91 | 20 | 2 | 125 | 51 | 6.099 | 1 |
| 2869 | 91 | 20 | 2 | 125 | 51 | 6.1 | 1 |
| 2201 | 105 | 26 | 2 | 200 | 51 | 6.1 | 1 |
| 2868 | 91 | 20 | 2 | 125 | 51 | 6.101 | 1 |
| 2867 | 91 | 20 | 2 | 125 | 51 | 6.102 | 1 |
| 2866 | 91 | 20 | 2 | 125 | 51 | 6.103 | 1 |
| 2865 | 91 | 20 | 2 | 125 | 51 | 6.104 | 1 |
| 2774 | 91 | 20 | 2 | 125 | 51 | 6.105 | 1 |
| 2773 | 91 | 20 | 2 | 125 | 51 | 6.106 | 1 |
| 2689 | 91 | 20 | 2 | 125 | 51 | 6.107 | 1 |
| 2688 | 91 | 20 | 2 | 125 | 51 | 6.108 | 1 |
| 2663 | 91 | 20 | 2 | 125 | 51 | 6.109 | 1 |
| 2662 | 91 | 20 | 2 | 125 | 51 | 6.11 | 1 |
| 2200 | 105 | 26 | 2 | 200 | 51 | 6.11 | 1 |
| 2661 | 91 | 20 | 2 | 130 | 51 | 6.111 | 1 |
| 2660 | 91 | 20 | 2 | 130 | 51 | 6.112 | 1 |
| 2637 | 91 | 20 | 2 | 130 | 51 | 6.113 | 1 |

| | | | | | | | |
|---|---|---|---|---|---|---|---|
| 2636 | 91 | 20 | 2 | 130 | 51 | 6.114 | 1 |
| 2635 | 91 | 20 | 2 | 130 | 51 | 6.115 | 1 |
| 2634 | 91 | 20 | 2 | 130 | 51 | 6.116 | 1 |
| 2633 | 91 | 20 | 2 | 130 | 51 | 6.117 | 1 |
| 2632 | 91 | 20 | 2 | 130 | 51 | 6.118 | 1 |
| 2602 | 91 | 20 | 2 | 130 | 51 | 6.119 | 1 |
| 2601 | 91 | 20 | 2 | 130 | 51 | 6.12 | 1 |
| 1893 | 105 | 26 | 2 | 200 | 51 | 6.12 | 1 |
| 2600 | 91 | 20 | 2 | 130 | 51 | 6.121 | 1 |
| 2599 | 91 | 20 | 2 | 130 | 51 | 6.122 | 1 |
| 2598 | 91 | 20 | 2 | 130 | 51 | 6.123 | 1 |
| 2597 | 91 | 20 | 2 | 130 | 51 | 6.124 | 1 |
| 2494 | 91 | 20 | 2 | 130 | 51 | 6.125 | 1 |
| 2459 | 91 | 20 | 2 | 130 | 51 | 6.126 | 1 |
| 2458 | 91 | 20 | 2 | 130 | 51 | 6.127 | 1 |
| 2457 | 91 | 20 | 2 | 130 | 51 | 6.128 | 1 |
| 2456 | 91 | 20 | 2 | 130 | 51 | 6.129 | 1 |
| 2455 | 91 | 20 | 2 | 130 | 51 | 6.13 | 1 |
| 3255 | 105 | 26 | 2 | 200 | 51 | 6.13 | 1 |
| 2454 | 91 | 20 | 2 | 130 | 51 | 6.131 | 1 |
| 2453 | 91 | 20 | 2 | 130 | 51 | 6.132 | 1 |
| 2448 | 91 | 20 | 2 | 130 | 51 | 6.133 | 1 |
| 2447 | 91 | 20 | 2 | 130 | 51 | 6.134 | 1 |
| 2446 | 91 | 20 | 2 | 130 | 51 | 6.135 | 1 |
| 2445 | 91 | 20 | 2 | 135 | 50 | 6.136 | 1 |
| 2444 | 91 | 20 | 2 | 135 | 50 | 6.137 | 1 |
| 2443 | 91 | 20 | 2 | 135 | 50 | 6.138 | 1 |
| 2442 | 91 | 20 | 2 | 135 | 50 | 6.139 | 1 |
| 2441 | 91 | 20 | 2 | 135 | 50 | 6.14 | 1 |
| 3254 | 105 | 26 | 2 | 200 | 50 | 6.14 | 1 |
| 2440 | 91 | 20 | 2 | 135 | 50 | 6.141 | 1 |
| 2439 | 91 | 20 | 2 | 135 | 50 | 6.142 | 1 |
| 2438 | 91 | 20 | 2 | 135 | 50 | 6.143 | 1 |
| 2437 | 91 | 20 | 2 | 135 | 50 | 6.144 | 1 |
| 2436 | 91 | 20 | 2 | 135 | 50 | 6.145 | 1 |
| 2435 | 91 | 20 | 2 | 135 | 50 | 6.146 | 1 |
| 2434 | 91 | 20 | 2 | 135 | 50 | 6.147 | 1 |
| 2433 | 91 | 20 | 2 | 135 | 50 | 6.148 | 1 |
| 2432 | 91 | 20 | 2 | 135 | 50 | 6.149 | 1 |
| 2431 | 91 | 20 | 2 | 135 | 50 | 6.15 | 1 |
| 3253 | 105 | 26 | 2 | 200 | 50 | 6.15 | 1 |
| 2409 | 91 | 20 | 2 | 135 | 50 | 6.151 | 1 |
| 2408 | 91 | 20 | 2 | 135 | 50 | 6.152 | 1 |
| 2407 | 91 | 20 | 2 | 135 | 50 | 6.153 | 1 |

| | | | | | | | |
|---|---|---|---|---|---|---|---|
| 2406 | 91 | 20 | 2 | 135 | 50 | 6.154 | 1 |
| 2405 | 91 | 20 | 2 | 135 | 50 | 6.155 | 1 |
| 2404 | 91 | 20 | 2 | 135 | 50 | 6.156 | 1 |
| 2403 | 91 | 20 | 2 | 135 | 50 | 6.157 | 1 |
| 2402 | 91 | 20 | 2 | 135 | 51 | 6.158 | 1 |
| 2401 | 91 | 20 | 2 | 135 | 51 | 6.159 | 1 |
| 2400 | 91 | 20 | 2 | 135 | 51 | 6.16 | 1 |
| 3252 | 105 | 26 | 2 | 200 | 51 | 6.16 | 1 |
| 2399 | 91 | 20 | 2 | 135 | 51 | 6.161 | 1 |
| 2398 | 91 | 20 | 2 | 135 | 51 | 6.162 | 1 |
| 2397 | 91 | 20 | 2 | 135 | 51 | 6.163 | 1 |
| 2396 | 91 | 20 | 2 | 135 | 51 | 6.164 | 1 |
| 2346 | 91 | 20 | 2 | 135 | 51 | 6.165 | 1 |
| 2325 | 91 | 20 | 2 | 135 | 51 | 6.166 | 1 |
| 2324 | 91 | 20 | 2 | 135 | 51 | 6.167 | 1 |
| 2323 | 91 | 20 | 2 | 135 | 51 | 6.168 | 1 |
| 2322 | 91 | 20 | 2 | 135 | 51 | 6.169 | 1 |
| 2321 | 91 | 20 | 2 | 135 | 51 | 6.17 | 1 |
| 3251 | 105 | 26 | 2 | 200 | 51 | 6.17 | 1 |
| 2314 | 91 | 20 | 2 | 135 | 51 | 6.171 | 1 |
| 2313 | 91 | 20 | 2 | 135 | 51 | 6.172 | 1 |
| 2312 | 91 | 20 | 2 | 135 | 51 | 6.173 | 1 |
| 3097 | 91 | 20 | 2 | 135 | 51 | 6.174 | 1 |
| 3096 | 91 | 20 | 2 | 135 | 51 | 6.175 | 1 |
| 3095 | 91 | 20 | 2 | 135 | 51 | 6.176 | 1 |
| 3094 | 91 | 20 | 2 | 135 | 51 | 6.177 | 1 |
| 3093 | 91 | 20 | 2 | 135 | 51 | 6.178 | 1 |
| 3092 | 91 | 20 | 2 | 135 | 51 | 6.179 | 1 |
| 3091 | 91 | 20 | 2 | 135 | 51 | 6.18 | 1 |
| 3250 | 105 | 26 | 2 | 200 | 51 | 6.18 | 1 |
| 2886 | 91 | 20 | 2 | 135 | 51 | 6.181 | 1 |
| 2885 | 91 | 20 | 2 | 135 | 51 | 6.182 | 1 |
| 2884 | 91 | 20 | 2 | 135 | 51 | 6.183 | 1 |
| 1923 | 86 | 19 | 2 | 135 | 51 | 6.184 | 1 |
| 2244 | 82 | 19 | 2 | 135 | 51 | 6.185 | 1 |
| 2243 | 82 | 19 | 2 | 135 | 51 | 6.186 | 1 |
| 2021 | 92 | 20 | 2 | 135 | 51 | 6.187 | 1 |
| 2054 | 92 | 20 | 2 | 135 | 51 | 6.188 | 1 |
| 2231 | 92 | 20 | 2 | 135 | 51 | 6.189 | 1 |
| 2278 | 92 | 20 | 2 | 135 | 51 | 6.19 | 1 |
| 3249 | 105 | 26 | 2 | 200 | 51 | 6.19 | 1 |
| 2471 | 92 | 20 | 2 | 135 | 51 | 6.191 | 1 |
| 2566 | 92 | 20 | 2 | 135 | 51 | 6.192 | 1 |
| 2676 | 92 | 20 | 2 | 135 | 51 | 6.193 | 1 |

| | | | | | | | |
|---|---|---|---|---|---|---|---|
| 1434 | 86 | 19 | 2 | 135 | 42 | 6.194 | 1 |
| 1048 | 82 | 19 | 2 | 135 | 51 | 6.195 | 1 |
| 1045 | 82 | 19 | 2 | 135 | 51 | 6.196 | 1 |
| 1044 | 82 | 19 | 2 | 135 | 51 | 6.197 | 1 |
| 1043 | 82 | 19 | 2 | 135 | 51 | 6.198 | 1 |
| 1729 | 92 | 20 | 2 | 135 | 51 | 6.199 | 1 |
| 3517 | 92 | 20 | 2 | 135 | 51 | 6.2 | 1 |
| 3248 | 105 | 26 | 2 | 200 | 51 | 6.2 | 1 |
| 3540 | 92 | 20 | 2 | 135 | 51 | 6.201 | 1 |
| 2311 | 91 | 20 | 2 | 140 | 51 | 6.202 | 1 |
| 2310 | 91 | 20 | 2 | 140 | 51 | 6.203 | 1 |
| 2298 | 91 | 20 | 2 | 140 | 51 | 6.204 | 1 |
| 2297 | 91 | 20 | 2 | 140 | 51 | 6.205 | 1 |
| 2284 | 91 | 20 | 2 | 140 | 51 | 6.206 | 1 |
| 2283 | 91 | 20 | 2 | 140 | 51 | 6.207 | 1 |
| 2239 | 91 | 20 | 2 | 140 | 51 | 6.208 | 1 |
| 2238 | 91 | 20 | 2 | 140 | 51 | 6.209 | 1 |
| 2237 | 91 | 20 | 2 | 140 | 51 | 6.21 | 1 |
| 3247 | 105 | 26 | 2 | 200 | 51 | 6.21 | 1 |
| 2139 | 91 | 20 | 2 | 140 | 51 | 6.211 | 1 |
| 2138 | 91 | 20 | 2 | 140 | 51 | 6.212 | 1 |
| 2137 | 91 | 20 | 2 | 140 | 51 | 6.213 | 1 |
| 1948 | 91 | 20 | 2 | 140 | 51 | 6.214 | 1 |
| 1914 | 91 | 20 | 2 | 140 | 51 | 6.215 | 1 |
| 1913 | 91 | 20 | 2 | 140 | 51 | 6.216 | 1 |
| 1912 | 91 | 20 | 2 | 140 | 51 | 6.217 | 1 |
| 1911 | 91 | 20 | 2 | 140 | 51 | 6.218 | 1 |
| 1867 | 91 | 20 | 2 | 140 | 51 | 6.219 | 1 |
| 1849 | 91 | 20 | 2 | 140 | 51 | 6.22 | 1 |
| 3246 | 105 | 26 | 2 | 200 | 51 | 6.22 | 1 |
| 1836 | 91 | 20 | 2 | 140 | 51 | 6.221 | 1 |
| 1820 | 91 | 20 | 2 | 140 | 51 | 6.222 | 1 |
| 1818 | 91 | 20 | 2 | 140 | 51 | 6.223 | 1 |
| 2856 | 91 | 20 | 2 | 140 | 51 | 6.224 | 1 |
| 3187 | 91 | 20 | 2 | 140 | 51 | 6.225 | 1 |
| 2855 | 91 | 20 | 2 | 140 | 51 | 6.226 | 1 |
| 3186 | 91 | 20 | 2 | 140 | 51 | 6.227 | 1 |
| 2854 | 91 | 20 | 2 | 140 | 51 | 6.228 | 1 |
| 3185 | 91 | 20 | 2 | 140 | 50 | 6.229 | 1 |
| 2853 | 91 | 20 | 2 | 140 | 50 | 6.23 | 1 |
| 3233 | 105 | 26 | 2 | 200 | 50 | 6.23 | 1 |
| 3184 | 91 | 20 | 2 | 140 | 50 | 6.231 | 1 |
| 3183 | 91 | 20 | 2 | 140 | 50 | 6.233 | 1 |
| 2851 | 91 | 20 | 2 | 140 | 50 | 6.234 | 1 |

| | | | | | | | |
|---|---|---|---|---|---|---|---|
| 3182 | 91 | 20 | 2 | 140 | 50 | 6.235 | 1 |
| 2850 | 91 | 20 | 2 | 140 | 50 | 6.236 | 1 |
| 3181 | 91 | 20 | 2 | 140 | 50 | 6.237 | 1 |
| 3210 | 91 | 20 | 2 | 140 | 50 | 6.238 | 1 |
| 3445 | 91 | 20 | 2 | 140 | 50 | 6.239 | 1 |
| 1830 | 91 | 20 | 2 | 140 | 50 | 6.24 | 1 |
| 3232 | 105 | 26 | 2 | 200 | 50 | 6.24 | 1 |
| 2030 | 91 | 20 | 2 | 140 | 50 | 6.241 | 1 |
| 2062 | 91 | 20 | 2 | 140 | 50 | 6.242 | 1 |
| 2247 | 91 | 20 | 2 | 140 | 50 | 6.243 | 1 |
| 2290 | 91 | 20 | 2 | 140 | 50 | 6.244 | 1 |
| 2498 | 91 | 20 | 2 | 140 | 50 | 6.245 | 1 |
| 2573 | 91 | 20 | 2 | 140 | 50 | 6.246 | 1 |
| 2696 | 91 | 20 | 2 | 140 | 50 | 6.247 | 1 |
| 2748 | 91 | 20 | 2 | 140 | 50 | 6.248 | 1 |
| 2849 | 91 | 20 | 2 | 140 | 50 | 6.249 | 1 |
| 3180 | 91 | 20 | 2 | 140 | 50 | 6.25 | 1 |
| 3231 | 105 | 26 | 2 | 200 | 50 | 6.25 | 1 |
| 3209 | 91 | 20 | 2 | 140 | 50 | 6.251 | 1 |
| 3444 | 91 | 20 | 2 | 140 | 50 | 6.252 | 1 |
| 3224 | 93 | 21 | 2 | 140 | 50 | 6.253 | 1 |
| 3223 | 93 | 21 | 2 | 140 | 50 | 6.254 | 1 |
| 3222 | 93 | 21 | 2 | 140 | 50 | 6.255 | 1 |
| 3221 | 93 | 21 | 2 | 140 | 50 | 6.256 | 1 |
| 3220 | 93 | 21 | 2 | 140 | 50 | 6.257 | 1 |
| 3219 | 93 | 21 | 2 | 140 | 50 | 6.258 | 1 |
| 3218 | 93 | 21 | 2 | 140 | 50 | 6.259 | 1 |
| 3217 | 93 | 21 | 2 | 140 | 50 | 6.26 | 1 |
| 3230 | 105 | 26 | 2 | 200 | 50 | 6.26 | 1 |
| 2083 | 93 | 21 | 2 | 140 | 50 | 6.261 | 1 |
| 2082 | 93 | 21 | 2 | 140 | 50 | 6.262 | 1 |
| 2009 | 93 | 21 | 2 | 140 | 50 | 6.263 | 1 |
| 2006 | 93 | 21 | 2 | 140 | 50 | 6.264 | 1 |
| 2001 | 93 | 21 | 2 | 140 | 50 | 6.265 | 1 |
| 2000 | 93 | 21 | 2 | 140 | 50 | 6.266 | 1 |
| 1999 | 93 | 21 | 2 | 140 | 50 | 6.267 | 1 |
| 1998 | 93 | 21 | 2 | 140 | 50 | 6.268 | 1 |
| 1997 | 93 | 21 | 2 | 140 | 50 | 6.269 | 1 |
| 1996 | 93 | 21 | 2 | 140 | 50 | 6.27 | 1 |
| 3070 | 105 | 26 | 2 | 200 | 50 | 6.27 | 1 |
| 2741 | 92 | 20 | 2 | 140 | 50 | 6.271 | 1 |
| 3077 | 92 | 20 | 2 | 140 | 50 | 6.272 | 1 |
| 3076 | 92 | 20 | 2 | 140 | 50 | 6.273 | 1 |
| 3075 | 92 | 20 | 2 | 140 | 50 | 6.274 | 1 |

| | | | | | | | |
|---|---|---|---|---|---|---|---|
| 2824 | 92 | 20 | 2 | 140 | 50 | 6.275 | 1 |
| 2823 | 92 | 20 | 2 | 140 | 50 | 6.276 | 1 |
| 2822 | 92 | 20 | 2 | 140 | 50 | 6.277 | 1 |
| 2821 | 92 | 20 | 2 | 140 | 50 | 6.278 | 1 |
| 2820 | 92 | 20 | 2 | 140 | 50 | 6.279 | 1 |
| 2819 | 92 | 20 | 2 | 140 | 50 | 6.28 | 1 |
| 3069 | 105 | 26 | 2 | 200 | 50 | 6.28 | 1 |
| 2818 | 92 | 20 | 2 | 140 | 50 | 6.281 | 1 |
| 2817 | 92 | 20 | 2 | 140 | 50 | 6.282 | 1 |
| 2816 | 92 | 20 | 2 | 140 | 50 | 6.283 | 1 |
| 2815 | 92 | 20 | 2 | 140 | 50 | 6.284 | 1 |
| 2814 | 92 | 20 | 2 | 140 | 50 | 6.285 | 1 |
| 2813 | 92 | 20 | 2 | 140 | 50 | 6.286 | 1 |
| 2812 | 92 | 20 | 2 | 140 | 50 | 6.287 | 1 |
| 1955 | 92 | 20 | 2 | 140 | 50 | 6.288 | 1 |
| 1954 | 92 | 20 | 2 | 140 | 50 | 6.289 | 1 |
| 3068 | 105 | 26 | 2 | 200 | 50 | 6.29 | 1 |
| 1952 | 92 | 20 | 2 | 140 | 50 | 6.291 | 1 |
| 1951 | 92 | 20 | 2 | 140 | 50 | 6.292 | 1 |
| 1950 | 92 | 20 | 2 | 140 | 50 | 6.293 | 1 |
| 1949 | 92 | 20 | 2 | 140 | 50 | 6.294 | 1 |
| 1910 | 92 | 20 | 2 | 140 | 50 | 6.295 | 1 |
| 1908 | 92 | 20 | 2 | 140 | 50 | 6.296 | 1 |
| 1906 | 92 | 20 | 2 | 140 | 50 | 6.297 | 1 |
| 1158 | 90 | 20 | 2 | 140 | 50 | 6.299 | 1 |
| 1151 | 90 | 20 | 2 | 140 | 50 | 6.3 | 1 |
| 3067 | 105 | 26 | 2 | 200 | 49 | 6.3 | 1 |
| 1150 | 90 | 20 | 2 | 140 | 50 | 6.301 | 1 |
| 2429 | 105 | 26 | 2 | 200 | 49 | 6.31 | 1 |
| 1995 | 93 | 21 | 2 | 145 | 49 | 6.32 | 1 |
| 2428 | 105 | 26 | 2 | 200 | 49 | 6.32 | 1 |
| 1994 | 93 | 21 | 2 | 145 | 49 | 6.321 | 1 |
| 1993 | 93 | 21 | 2 | 145 | 49 | 6.322 | 1 |
| 1991 | 93 | 21 | 2 | 145 | 49 | 6.323 | 1 |
| 1990 | 93 | 21 | 2 | 145 | 49 | 6.324 | 1 |
| 1989 | 93 | 21 | 2 | 145 | 49 | 6.325 | 1 |
| 1845 | 93 | 21 | 2 | 145 | 49 | 6.326 | 1 |
| 1825 | 93 | 21 | 2 | 145 | 49 | 6.327 | 1 |
| 2246 | 92 | 21 | 2 | 145 | 49 | 6.328 | 1 |
| 2289 | 92 | 21 | 2 | 145 | 49 | 6.329 | 1 |
| 2497 | 92 | 21 | 2 | 145 | 49 | 6.33 | 1 |
| 2427 | 105 | 26 | 2 | 200 | 49 | 6.33 | 1 |
| 2572 | 92 | 21 | 2 | 145 | 49 | 6.331 | 1 |
| 2695 | 92 | 21 | 2 | 145 | 49 | 6.332 | 1 |

| | | | | | | | |
|---|---|---|---|---|---|---|---|
| 2747 | 92 | 21 | 2 | 145 | 49 | 6.333 | 1 |
| 2848 | 92 | 21 | 2 | 145 | 49 | 6.334 | 1 |
| 3179 | 92 | 21 | 2 | 145 | 49 | 6.335 | 1 |
| 3208 | 92 | 21 | 2 | 145 | 49 | 6.336 | 1 |
| 3443 | 92 | 21 | 2 | 145 | 49 | 6.337 | 1 |
| 2026 | 92 | 21 | 2 | 145 | 49 | 6.338 | 1 |
| 2060 | 92 | 21 | 2 | 145 | 49 | 6.339 | 1 |
| 2236 | 92 | 21 | 2 | 145 | 49 | 6.34 | 1 |
| 2359 | 105 | 26 | 2 | 200 | 49 | 6.34 | 1 |
| 2288 | 92 | 21 | 2 | 145 | 49 | 6.341 | 1 |
| 2496 | 92 | 21 | 2 | 145 | 49 | 6.342 | 1 |
| 2571 | 92 | 21 | 2 | 145 | 49 | 6.343 | 1 |
| 2694 | 92 | 21 | 2 | 145 | 49 | 6.344 | 1 |
| 2746 | 92 | 21 | 2 | 145 | 49 | 6.345 | 1 |
| 2847 | 92 | 21 | 2 | 145 | 49 | 6.346 | 1 |
| 3178 | 92 | 21 | 2 | 145 | 49 | 6.347 | 1 |
| 3207 | 92 | 21 | 2 | 145 | 49 | 6.348 | 1 |
| 3442 | 92 | 21 | 2 | 145 | 49 | 6.349 | 1 |
| 2025 | 92 | 21 | 2 | 145 | 49 | 6.35 | 1 |
| 2358 | 105 | 26 | 2 | 200 | 49 | 6.35 | 1 |
| 2059 | 92 | 21 | 2 | 145 | 49 | 6.351 | 1 |
| 2235 | 92 | 21 | 2 | 145 | 49 | 6.352 | 1 |
| 2287 | 92 | 21 | 2 | 145 | 49 | 6.353 | 1 |
| 2495 | 92 | 21 | 2 | 145 | 49 | 6.354 | 1 |
| 2570 | 92 | 21 | 2 | 145 | 49 | 6.355 | 1 |
| 2693 | 92 | 21 | 2 | 145 | 49 | 6.356 | 1 |
| 2745 | 92 | 21 | 2 | 145 | 49 | 6.357 | 1 |
| 2846 | 92 | 21 | 2 | 145 | 49 | 6.358 | 1 |
| 3177 | 92 | 21 | 2 | 145 | 49 | 6.359 | 1 |
| 3206 | 92 | 21 | 2 | 145 | 49 | 6.36 | 1 |
| 2357 | 105 | 26 | 2 | 200 | 49 | 6.36 | 1 |
| 3441 | 92 | 21 | 2 | 145 | 49 | 6.361 | 1 |
| 2024 | 92 | 21 | 2 | 145 | 49 | 6.362 | 1 |
| 2058 | 92 | 21 | 2 | 145 | 49 | 6.363 | 1 |
| 2234 | 92 | 21 | 2 | 145 | 49 | 6.364 | 1 |
| 2281 | 92 | 21 | 2 | 145 | 49 | 6.365 | 1 |
| 2474 | 92 | 21 | 2 | 145 | 43 | 6.366 | 1 |
| 2569 | 92 | 21 | 2 | 145 | 43 | 6.367 | 1 |
| 2692 | 92 | 21 | 2 | 145 | 43 | 6.368 | 1 |
| 2744 | 92 | 21 | 2 | 145 | 43 | 6.369 | 1 |
| 2341 | 105 | 26 | 2 | 200 | 43 | 6.37 | 1 |
| 2845 | 92 | 21 | 2 | 145 | 43 | 6.37 | 1 |
| 3176 | 92 | 21 | 2 | 145 | 43 | 6.371 | 1 |
| 3205 | 92 | 21 | 2 | 145 | 43 | 6.372 | 1 |

| | | | | | | | |
|---|---|---|---|---|---|---|---|
| 3440 | 92 | 21 | 2 | 145 | 43 | 6.373 | 1 |
| 2023 | 92 | 21 | 2 | 145 | 43 | 6.374 | 1 |
| 2056 | 92 | 21 | 2 | 145 | 43 | 6.375 | 1 |
| 2233 | 92 | 21 | 2 | 145 | 43 | 6.376 | 1 |
| 2280 | 92 | 21 | 2 | 145 | 43 | 6.377 | 1 |
| 2473 | 92 | 21 | 2 | 145 | 43 | 6.378 | 1 |
| 2568 | 92 | 21 | 2 | 145 | 43 | 6.379 | 1 |
| 2691 | 92 | 21 | 2 | 145 | 43 | 6.38 | 1 |
| 2340 | 105 | 26 | 2 | 200 | 43 | 6.38 | 1 |
| 2743 | 92 | 21 | 2 | 145 | 43 | 6.381 | 1 |
| 2844 | 92 | 21 | 2 | 145 | 43 | 6.382 | 1 |
| 3175 | 92 | 21 | 2 | 145 | 43 | 6.383 | 1 |
| 3204 | 92 | 21 | 2 | 145 | 43 | 6.384 | 1 |
| 3439 | 92 | 21 | 2 | 145 | 43 | 6.385 | 1 |
| 2022 | 92 | 21 | 2 | 145 | 43 | 6.386 | 1 |
| 2055 | 92 | 21 | 2 | 145 | 43 | 6.387 | 1 |
| 2232 | 92 | 21 | 2 | 145 | 43 | 6.388 | 1 |
| 2279 | 92 | 21 | 2 | 145 | 43 | 6.389 | 1 |
| 2472 | 92 | 21 | 2 | 145 | 43 | 6.39 | 1 |
| 2274 | 105 | 26 | 2 | 200 | 43 | 6.39 | 1 |
| 2567 | 92 | 21 | 2 | 145 | 43 | 6.391 | 1 |
| 2690 | 92 | 21 | 2 | 145 | 43 | 6.392 | 1 |
| 2742 | 92 | 21 | 2 | 145 | 43 | 6.393 | 1 |
| 2811 | 92 | 21 | 2 | 145 | 43 | 6.394 | 1 |
| 2810 | 92 | 21 | 2 | 145 | 43 | 6.395 | 1 |
| 2809 | 92 | 21 | 2 | 145 | 43 | 6.396 | 1 |
| 2757 | 92 | 21 | 2 | 145 | 43 | 6.397 | 1 |
| 2756 | 92 | 21 | 2 | 145 | 43 | 6.398 | 1 |
| 2755 | 92 | 21 | 2 | 145 | 43 | 6.399 | 1 |
| 2754 | 92 | 21 | 2 | 145 | 43 | 6.4 | 1 |
| 2273 | 105 | 26 | 2 | 200 | 43 | 6.4 | 1 |
| 2753 | 92 | 21 | 2 | 145 | 43 | 6.401 | 1 |
| 2752 | 92 | 21 | 2 | 145 | 43 | 6.402 | 1 |
| 2751 | 92 | 21 | 2 | 145 | 43 | 6.403 | 1 |
| 2750 | 92 | 21 | 2 | 145 | 43 | 6.404 | 1 |
| 2749 | 92 | 21 | 2 | 145 | 43 | 6.405 | 1 |
| 1885 | 92 | 21 | 2 | 145 | 43 | 6.406 | 1 |
| 1884 | 92 | 21 | 2 | 145 | 43 | 6.407 | 1 |
| 1883 | 92 | 21 | 2 | 145 | 43 | 6.408 | 1 |
| 1880 | 92 | 21 | 2 | 145 | 43 | 6.409 | 1 |
| 1878 | 92 | 21 | 2 | 145 | 43 | 6.41 | 1 |
| 2272 | 105 | 26 | 2 | 200 | 43 | 6.41 | 1 |
| 1877 | 92 | 21 | 2 | 145 | 43 | 6.411 | 1 |
| 1876 | 92 | 21 | 2 | 145 | 43 | 6.412 | 1 |

| | | | | | | | |
|---|---|---|---|---|---|---|---|
| 1875 | 92 | 21 | 2 | 145 | 43 | 6.413 | 1 |
| 1874 | 92 | 21 | 2 | 145 | 43 | 6.414 | 1 |
| 1873 | 92 | 21 | 2 | 145 | 43 | 6.415 | 1 |
| 1872 | 92 | 21 | 2 | 145 | 43 | 6.416 | 1 |
| 1871 | 92 | 21 | 2 | 145 | 43 | 6.417 | 1 |
| 3499 | 92 | 21 | 2 | 145 | 43 | 6.418 | 1 |
| 3522 | 92 | 21 | 2 | 145 | 43 | 6.419 | 1 |
| 3498 | 92 | 21 | 2 | 145 | 43 | 6.42 | 1 |
| 2263 | 105 | 26 | 2 | 200 | 43 | 6.42 | 1 |
| 3521 | 92 | 21 | 2 | 145 | 43 | 6.421 | 1 |
| 3544 | 92 | 21 | 2 | 145 | 43 | 6.422 | 1 |
| 3497 | 92 | 21 | 2 | 145 | 43 | 6.423 | 1 |
| 3520 | 92 | 21 | 2 | 145 | 43 | 6.424 | 1 |
| 3543 | 92 | 21 | 2 | 145 | 43 | 6.425 | 1 |
| 3496 | 92 | 21 | 2 | 145 | 43 | 6.426 | 1 |
| 3519 | 92 | 21 | 2 | 145 | 43 | 6.427 | 1 |
| 3542 | 92 | 21 | 2 | 145 | 43 | 6.428 | 1 |
| 3518 | 92 | 21 | 2 | 145 | 43 | 6.429 | 1 |
| 2262 | 105 | 26 | 2 | 200 | 43 | 6.43 | 1 |
| 2261 | 105 | 26 | 2 | 200 | 43 | 6.44 | 1 |
| 2212 | 105 | 26 | 2 | 200 | 43 | 6.45 | 0 |
| 2193 | 105 | 26 | 2 | 200 | 43 | 6.46 | 0 |
| 3315 | 92 | 21 | 2 | 150 | 42 | 6.464 | 0 |
| 3314 | 92 | 21 | 2 | 150 | 42 | 6.465 | 0 |
| 3313 | 92 | 21 | 2 | 150 | 42 | 6.466 | 0 |
| 3312 | 92 | 21 | 2 | 150 | 42 | 6.467 | 1 |
| 3311 | 92 | 21 | 2 | 150 | 42 | 6.468 | 1 |
| 3074 | 92 | 21 | 2 | 150 | 42 | 6.469 | 1 |
| 3073 | 92 | 21 | 2 | 150 | 42 | 6.47 | 0 |
| 2192 | 105 | 26 | 2 | 200 | 42 | 6.47 | 0 |
| 3072 | 92 | 21 | 2 | 150 | 42 | 6.471 | 0 |
| 3071 | 92 | 21 | 2 | 150 | 42 | 6.472 | 0 |
| 2728 | 92 | 21 | 2 | 150 | 42 | 6.473 | 0 |
| 2727 | 92 | 21 | 2 | 150 | 42 | 6.474 | 0 |
| 2726 | 92 | 21 | 2 | 150 | 42 | 6.475 | 0 |
| 2843 | 92 | 21 | 2 | 150 | 42 | 6.476 | 0 |
| 3174 | 92 | 21 | 2 | 150 | 42 | 6.477 | 0 |
| 3203 | 92 | 21 | 2 | 150 | 42 | 6.478 | 0 |
| 3438 | 92 | 21 | 2 | 150 | 42 | 6.479 | 1 |
| 2673 | 94 | 22 | 2 | 150 | 42 | 6.48 | 1 |
| 2191 | 105 | 26 | 2 | 200 | 42 | 6.48 | 0 |
| 2738 | 94 | 22 | 2 | 150 | 42 | 6.481 | 0 |
| 3280 | 94 | 22 | 2 | 150 | 42 | 6.482 | 0 |
| 3279 | 94 | 22 | 2 | 150 | 42 | 6.483 | 0 |

| | | | | | | | |
|---|---|---|---|---|---|---|---|
| 3278 | 94 | 22 | 2 | 150 | 42 | 6.484 | 0 |
| 3277 | 94 | 22 | 2 | 150 | 42 | 6.485 | 0 |
| 3276 | 94 | 22 | 2 | 150 | 42 | 6.486 | 1 |
| 3275 | 94 | 22 | 2 | 150 | 42 | 6.487 | 1 |
| 3274 | 94 | 22 | 2 | 150 | 42 | 6.488 | 0 |
| 3273 | 94 | 22 | 2 | 150 | 42 | 6.489 | 1 |
| 3272 | 94 | 22 | 2 | 150 | 42 | 6.49 | 0 |
| 2190 | 105 | 26 | 2 | 200 | 42 | 6.49 | 1 |
| 3271 | 94 | 22 | 2 | 150 | 42 | 6.491 | 0 |
| 3270 | 94 | 22 | 2 | 150 | 42 | 6.492 | 1 |
| 3269 | 94 | 22 | 2 | 150 | 42 | 6.493 | 1 |
| 3268 | 94 | 22 | 2 | 150 | 42 | 6.494 | 1 |
| 3267 | 94 | 22 | 2 | 150 | 42 | 6.495 | 1 |
| 3266 | 94 | 22 | 2 | 150 | 42 | 6.496 | 1 |
| 3265 | 94 | 22 | 2 | 150 | 42 | 6.497 | 1 |
| 2099 | 94 | 22 | 2 | 150 | 42 | 6.498 | 1 |
| 2098 | 94 | 22 | 2 | 150 | 42 | 6.499 | 0 |
| 2097 | 94 | 22 | 2 | 150 | 42 | 6.5 | 0 |
| 2136 | 105 | 26 | 2 | 200 | 42 | 6.5 | 0 |
| 2096 | 94 | 22 | 2 | 150 | 42 | 6.501 | 1 |
| 2095 | 94 | 22 | 2 | 150 | 42 | 6.502 | 1 |
| 2094 | 94 | 22 | 2 | 150 | 42 | 6.503 | 1 |
| 2093 | 94 | 22 | 2 | 150 | 42 | 6.504 | 1 |
| 2092 | 94 | 22 | 2 | 150 | 42 | 6.505 | 0 |
| 2091 | 94 | 22 | 2 | 150 | 42 | 6.506 | 1 |
| 2090 | 94 | 22 | 2 | 150 | 42 | 6.507 | 0 |
| 2089 | 94 | 22 | 2 | 150 | 42 | 6.508 | 1 |
| 2088 | 94 | 22 | 2 | 150 | 42 | 6.509 | 1 |
| 2087 | 94 | 22 | 2 | 150 | 42 | 6.51 | 1 |
| 3245 | 105 | 26 | 2 | 200 | 42 | 6.51 | 1 |
| 2086 | 94 | 22 | 2 | 150 | 42 | 6.511 | 1 |
| 2085 | 94 | 22 | 2 | 150 | 42 | 6.512 | 1 |
| 2084 | 94 | 22 | 2 | 150 | 42 | 6.513 | 1 |
| 2383 | 94 | 22 | 2 | 150 | 42 | 6.514 | 1 |
| 2382 | 94 | 22 | 2 | 150 | 42 | 6.515 | 1 |
| 3494 | 92 | 21 | 2 | 150 | 42 | 6.516 | 1 |
| 2381 | 94 | 22 | 2 | 155 | 42 | 6.517 | 1 |
| 2351 | 94 | 22 | 2 | 155 | 42 | 6.518 | 1 |
| 2350 | 94 | 22 | 2 | 155 | 42 | 6.519 | 1 |
| 2349 | 94 | 22 | 2 | 155 | 42 | 6.52 | 1 |
| 3244 | 105 | 26 | 2 | 200 | 42 | 6.52 | 1 |
| 2348 | 94 | 22 | 2 | 155 | 42 | 6.521 | 1 |
| 2331 | 94 | 22 | 2 | 155 | 42 | 6.522 | 1 |
| 2186 | 94 | 22 | 2 | 155 | 42 | 6.523 | 1 |

| | | | | | | | |
|---|---|---|---|---|---|---|---|
| 2185 | 94 | 22 | 2 | 155 | 42 | 6.524 | 1 |
| 2184 | 94 | 22 | 2 | 155 | 42 | 6.525 | 1 |
| 2142 | 94 | 22 | 2 | 155 | 42 | 6.526 | 1 |
| 1973 | 94 | 22 | 2 | 155 | 42 | 6.527 | 1 |
| 1972 | 94 | 22 | 2 | 155 | 42 | 6.528 | 0 |
| 1971 | 94 | 22 | 2 | 155 | 42 | 6.529 | 0 |
| 1848 | 94 | 22 | 2 | 155 | 42 | 6.53 | 1 |
| 3243 | 105 | 26 | 2 | 200 | 42 | 6.53 | 0 |
| 2546 | 94 | 22 | 2 | 155 | 42 | 6.531 | 1 |
| 2545 | 94 | 22 | 2 | 155 | 42 | 6.532 | 0 |
| 2544 | 94 | 22 | 2 | 155 | 42 | 6.533 | 0 |
| 2543 | 94 | 22 | 2 | 155 | 42 | 6.534 | 0 |
| 2542 | 94 | 22 | 2 | 155 | 42 | 6.535 | 0 |
| 2541 | 94 | 22 | 2 | 155 | 42 | 6.536 | 1 |
| 2540 | 94 | 22 | 2 | 155 | 42 | 6.537 | 0 |
| 2539 | 94 | 22 | 2 | 155 | 42 | 6.538 | 0 |
| 2538 | 94 | 22 | 2 | 155 | 42 | 6.539 | 1 |
| 2537 | 94 | 22 | 2 | 155 | 42 | 6.54 | 0 |
| 3242 | 105 | 26 | 2 | 200 | 42 | 6.54 | 1 |
| 2536 | 94 | 22 | 2 | 155 | 42 | 6.541 | 1 |
| 2535 | 94 | 22 | 2 | 155 | 42 | 6.542 | 0 |
| 2534 | 94 | 22 | 2 | 155 | 42 | 6.543 | 0 |
| 2533 | 94 | 22 | 2 | 155 | 42 | 6.544 | 0 |
| 2532 | 94 | 22 | 2 | 155 | 42 | 6.545 | 1 |
| 2531 | 94 | 22 | 2 | 155 | 42 | 6.546 | 0 |
| 2839 | 94 | 22 | 2 | 155 | 42 | 6.547 | 0 |
| 2861 | 94 | 22 | 2 | 155 | 42 | 6.548 | 1 |
| 3199 | 94 | 22 | 2 | 155 | 42 | 6.549 | 1 |
| 3434 | 94 | 22 | 2 | 155 | 42 | 6.55 | 0 |
| 3241 | 105 | 26 | 2 | 200 | 42 | 6.55 | 1 |
| 2013 | 94 | 22 | 2 | 155 | 60 | 6.551 | 0 |
| 2043 | 94 | 22 | 2 | 155 | 60 | 6.552 | 0 |
| 3490 | 94 | 22 | 2 | 155 | 60 | 6.553 | 0 |
| 3513 | 94 | 22 | 2 | 155 | 60 | 6.554 | 0 |
| 3536 | 94 | 22 | 2 | 155 | 60 | 6.555 | 0 |
| 2227 | 94 | 22 | 2 | 160 | 60 | 6.557 | 0 |
| 2259 | 94 | 22 | 2 | 160 | 60 | 6.558 | 0 |
| 2467 | 94 | 22 | 2 | 160 | 60 | 6.559 | 0 |
| 2510 | 94 | 22 | 2 | 160 | 60 | 6.56 | 0 |
| 3240 | 105 | 26 | 2 | 200 | 42 | 6.56 | 0 |
| 2672 | 94 | 22 | 2 | 160 | 60 | 6.561 | 1 |
| 2737 | 94 | 22 | 2 | 160 | 60 | 6.562 | 1 |
| 3296 | 94 | 22 | 2 | 160 | 60 | 6.563 | 1 |
| 3295 | 94 | 22 | 2 | 160 | 60 | 6.564 | 0 |

| | | | | | | | |
|---|---|---|---|---|---|---|---|
| 3294 | 94 | 22 | 2 | 160 | 60 | 6.565 | 0 |
| 3293 | 94 | 22 | 2 | 160 | 60 | 6.566 | 0 |
| 3292 | 94 | 22 | 2 | 160 | 60 | 6.567 | 1 |
| 3291 | 94 | 22 | 2 | 160 | 60 | 6.568 | 1 |
| 3290 | 94 | 22 | 2 | 160 | 60 | 6.569 | 1 |
| 3239 | 105 | 26 | 2 | 200 | 42 | 6.57 | 1 |
| 3289 | 94 | 22 | 2 | 160 | 60 | 6.57 | 1 |
| 3288 | 94 | 22 | 2 | 160 | 60 | 6.571 | 0 |
| 3287 | 94 | 22 | 2 | 160 | 60 | 6.572 | 1 |
| 3286 | 94 | 22 | 2 | 160 | 60 | 6.573 | 1 |
| 3285 | 94 | 22 | 2 | 160 | 60 | 6.574 | 1 |
| 3284 | 94 | 22 | 2 | 160 | 60 | 6.575 | 1 |
| 3283 | 94 | 22 | 2 | 160 | 60 | 6.576 | 1 |
| 3282 | 94 | 22 | 2 | 160 | 60 | 6.577 | 1 |
| 3281 | 94 | 22 | 2 | 160 | 60 | 6.578 | 1 |
| 2118 | 94 | 22 | 2 | 160 | 60 | 6.579 | 1 |
| 3238 | 105 | 26 | 2 | 200 | 42 | 6.58 | 1 |
| 2117 | 94 | 22 | 2 | 160 | 60 | 6.58 | 1 |
| 2116 | 94 | 22 | 2 | 160 | 60 | 6.581 | 1 |
| 2112 | 94 | 22 | 2 | 160 | 60 | 6.582 | 0 |
| 2111 | 94 | 22 | 2 | 160 | 60 | 6.583 | 0 |
| 2110 | 94 | 22 | 2 | 160 | 60 | 6.584 | 0 |
| 2109 | 94 | 22 | 2 | 160 | 60 | 6.585 | 0 |
| 2108 | 94 | 22 | 2 | 160 | 60 | 6.586 | 0 |
| 2107 | 94 | 22 | 2 | 160 | 60 | 6.587 | 0 |
| 2106 | 94 | 22 | 2 | 160 | 60 | 6.588 | 0 |
| 2105 | 94 | 22 | 2 | 160 | 60 | 6.589 | 0 |
| 2104 | 94 | 22 | 2 | 160 | 60 | 6.59 | 0 |
| 3237 | 105 | 26 | 2 | 200 | 42 | 6.59 | 0 |
| 2103 | 94 | 22 | 2 | 160 | 60 | 6.591 | 0 |
| 2102 | 94 | 22 | 2 | 160 | 60 | 6.592 | 1 |
| 2101 | 94 | 22 | 2 | 160 | 60 | 6.593 | 0 |
| 2100 | 94 | 22 | 2 | 165 | 60 | 6.594 | 0 |
| 2584 | 94 | 22 | 2 | 165 | 60 | 6.595 | 0 |
| 2583 | 94 | 22 | 2 | 165 | 60 | 6.596 | 0 |
| 2523 | 94 | 22 | 2 | 165 | 60 | 6.597 | 0 |
| 2522 | 94 | 22 | 2 | 165 | 60 | 6.598 | 0 |
| 2521 | 94 | 22 | 2 | 165 | 60 | 6.599 | 0 |
| 2520 | 94 | 22 | 2 | 165 | 60 | 6.6 | 0 |
| 3236 | 105 | 26 | 2 | 200 | 42 | 6.6 | 0 |
| 2519 | 94 | 22 | 2 | 165 | 60 | 6.601 | 0 |
| 2518 | 94 | 22 | 2 | 165 | 60 | 6.602 | 0 |
| 2517 | 94 | 22 | 2 | 165 | 60 | 6.603 | 0 |
| 2516 | 94 | 22 | 2 | 165 | 60 | 6.604 | 0 |

| | | | | | | | |
|---|---|---|---|---|---|---|---|
| 2515 | 94 | 22 | 2 | 165 | 60 | 6.605 | 0 |
| 2514 | 94 | 22 | 2 | 165 | 60 | 6.606 | 0 |
| 2513 | 94 | 22 | 2 | 165 | 60 | 6.607 | 0 |
| 2512 | 94 | 22 | 2 | 165 | 60 | 6.608 | 0 |
| 2511 | 94 | 22 | 2 | 165 | 60 | 6.609 | 0 |
| 2475 | 94 | 22 | 2 | 165 | 60 | 6.61 | 0 |
| 3235 | 104 | 26 | 2 | 200 | 42 | 6.61 | 0 |
| 2722 | 94 | 22 | 2 | 165 | 60 | 6.611 | 1 |
| 2713 | 94 | 22 | 2 | 165 | 60 | 6.612 | 0 |
| 2712 | 94 | 22 | 2 | 165 | 60 | 6.613 | 0 |
| 2711 | 94 | 22 | 2 | 165 | 60 | 6.614 | 0 |
| 2710 | 94 | 22 | 2 | 165 | 60 | 6.615 | 0 |
| 2709 | 94 | 22 | 2 | 165 | 60 | 6.616 | 0 |
| 2683 | 94 | 22 | 2 | 165 | 60 | 6.617 | 0 |
| 2682 | 94 | 22 | 2 | 165 | 60 | 6.618 | 0 |
| 2647 | 94 | 22 | 2 | 165 | 60 | 6.619 | 0 |
| 2646 | 94 | 22 | 2 | 165 | 60 | 6.62 | 0 |
| 3234 | 104 | 26 | 2 | 200 | 42 | 6.62 | 0 |
| 2645 | 94 | 22 | 2 | 165 | 60 | 6.621 | 0 |
| 2644 | 94 | 22 | 2 | 165 | 60 | 6.622 | 0 |
| 2643 | 94 | 22 | 2 | 165 | 60 | 6.623 | 0 |
| 2590 | 94 | 22 | 2 | 165 | 60 | 6.624 | 0 |
| 2548 | 94 | 22 | 2 | 170 | 60 | 6.625 | 0 |
| 2547 | 94 | 22 | 2 | 170 | 60 | 6.626 | 0 |
| 2838 | 94 | 22 | 2 | 170 | 59 | 6.627 | 0 |
| 2860 | 94 | 22 | 2 | 170 | 59 | 6.628 | 0 |
| 3198 | 94 | 22 | 2 | 170 | 59 | 6.629 | 0 |
| 1901 | 93 | 22 | 2 | 170 | 64 | 6.63 | 0 |
| 3229 | 104 | 26 | 2 | 200 | 42 | 6.63 | 0 |
| 1892 | 93 | 22 | 2 | 170 | 64 | 6.631 | 1 |
| 1886 | 93 | 22 | 2 | 170 | 64 | 6.632 | 0 |
| 2343 | 93 | 22 | 2 | 170 | 64 | 6.633 | 1 |
| 2342 | 93 | 22 | 2 | 170 | 64 | 6.634 | 0 |
| 3228 | 104 | 26 | 2 | 200 | 42 | 6.64 | 0 |
| 3227 | 104 | 26 | 2 | 200 | 42 | 6.65 | 0 |
| 2315 | 93 | 22 | 2 | 175 | 64 | 6.651 | 0 |
| 2213 | 93 | 22 | 2 | 175 | 64 | 6.652 | 1 |
| 2198 | 93 | 22 | 2 | 175 | 64 | 6.653 | 0 |
| 2197 | 93 | 22 | 2 | 175 | 64 | 6.654 | 0 |
| 2196 | 93 | 22 | 2 | 175 | 64 | 6.655 | 1 |
| 2195 | 93 | 22 | 2 | 175 | 64 | 6.656 | 0 |
| 2194 | 93 | 22 | 2 | 175 | 64 | 6.657 | 0 |
| 2169 | 93 | 22 | 2 | 175 | 64 | 6.658 | 1 |
| 2168 | 93 | 22 | 2 | 175 | 64 | 6.659 | 1 |

| | | | | | | | |
|---|---|---|---|---|---|---|---|
| 2167 | 93 | 22 | 2 | 175 | 64 | 6.66 | 0 |
| 3226 | 104 | 26 | 2 | 200 | 42 | 6.66 | 1 |
| 2842 | 93 | 22 | 2 | 175 | 63 | 6.661 | 0 |
| 2864 | 93 | 22 | 2 | 175 | 63 | 6.662 | 0 |
| 3202 | 93 | 22 | 2 | 175 | 63 | 6.663 | 1 |
| 3437 | 93 | 22 | 2 | 175 | 63 | 6.664 | 0 |
| 2020 | 93 | 22 | 2 | 175 | 63 | 6.665 | 1 |
| 2051 | 93 | 22 | 2 | 175 | 63 | 6.666 | 1 |
| 2230 | 93 | 22 | 2 | 175 | 63 | 6.667 | 1 |
| 2277 | 93 | 22 | 2 | 175 | 63 | 6.668 | 0 |
| 2470 | 93 | 22 | 2 | 175 | 63 | 6.669 | 0 |
| 2565 | 93 | 22 | 2 | 175 | 63 | 6.67 | 0 |
| 3225 | 104 | 26 | 2 | 200 | 42 | 6.67 | 0 |
| 2675 | 93 | 22 | 2 | 175 | 63 | 6.671 | 0 |
| 2740 | 93 | 22 | 2 | 175 | 63 | 6.672 | 0 |
| 3216 | 93 | 22 | 2 | 175 | 63 | 6.673 | 0 |
| 3215 | 93 | 22 | 2 | 175 | 63 | 6.674 | 0 |
| 3214 | 93 | 22 | 2 | 175 | 63 | 6.675 | 0 |
| 3090 | 93 | 22 | 2 | 175 | 63 | 6.676 | 0 |
| 3089 | 93 | 22 | 2 | 175 | 63 | 6.677 | 0 |
| 3088 | 93 | 22 | 2 | 175 | 63 | 6.678 | 0 |
| 3087 | 93 | 22 | 2 | 175 | 63 | 6.679 | 0 |
| 3086 | 93 | 22 | 2 | 175 | 63 | 6.68 | 0 |
| 3048 | 104 | 26 | 2 | 200 | 42 | 6.68 | 0 |
| 3085 | 93 | 22 | 2 | 175 | 63 | 6.681 | 0 |
| 3084 | 93 | 22 | 2 | 175 | 63 | 6.682 | 0 |
| 3083 | 93 | 22 | 2 | 175 | 63 | 6.683 | 0 |
| 3082 | 93 | 22 | 2 | 175 | 63 | 6.684 | 1 |
| 3081 | 93 | 22 | 2 | 175 | 63 | 6.685 | 0 |
| 3080 | 93 | 22 | 2 | 175 | 63 | 6.686 | 1 |
| 3079 | 93 | 22 | 2 | 175 | 63 | 6.687 | 0 |
| 3078 | 93 | 22 | 2 | 175 | 63 | 6.688 | 0 |
| 1988 | 93 | 22 | 2 | 175 | 63 | 6.689 | 0 |
| 1970 | 93 | 22 | 2 | 175 | 63 | 6.69 | 0 |
| 3047 | 104 | 26 | 2 | 200 | 42 | 6.69 | 0 |
| 1969 | 93 | 22 | 2 | 175 | 63 | 6.691 | 0 |
| 1968 | 93 | 22 | 2 | 175 | 63 | 6.692 | 0 |
| 1967 | 93 | 22 | 2 | 175 | 63 | 6.693 | 0 |
| 1966 | 93 | 22 | 2 | 175 | 63 | 6.694 | 1 |
| 1965 | 93 | 22 | 2 | 175 | 63 | 6.695 | 0 |
| 1964 | 93 | 22 | 2 | 175 | 63 | 6.696 | 1 |
| 1963 | 93 | 22 | 2 | 175 | 63 | 6.697 | 0 |
| 1962 | 93 | 22 | 2 | 175 | 63 | 6.698 | 0 |
| 1961 | 93 | 22 | 2 | 175 | 63 | 6.699 | 0 |

| | | | | | | | |
|---|---|---|---|---|---|---|---|
| 1960 | 93 | 22 | 2 | 175 | 63 | 6.7 | 0 |
| 3046 | 104 | 26 | 2 | 200 | 42 | 6.7 | 0 |
| 1959 | 93 | 22 | 2 | 175 | 63 | 6.701 | 0 |
| 1958 | 93 | 22 | 2 | 175 | 63 | 6.702 | 0 |
| 1957 | 93 | 22 | 2 | 175 | 63 | 6.703 | 0 |
| 1956 | 93 | 22 | 2 | 175 | 63 | 6.704 | 0 |
| 2413 | 93 | 22 | 2 | 175 | 63 | 6.705 | 0 |
| 2412 | 93 | 22 | 2 | 175 | 63 | 6.706 | 0 |
| 2392 | 93 | 22 | 2 | 175 | 63 | 6.707 | 0 |
| 2391 | 93 | 22 | 2 | 175 | 63 | 6.708 | 1 |
| 2376 | 93 | 22 | 2 | 175 | 63 | 6.709 | 0 |
| 2368 | 93 | 22 | 2 | 175 | 63 | 6.71 | 0 |
| 3045 | 104 | 26 | 2 | 200 | 42 | 6.71 | 0 |
| 2367 | 93 | 22 | 2 | 175 | 63 | 6.711 | 0 |
| 2366 | 93 | 22 | 2 | 175 | 63 | 6.712 | 0 |
| 2365 | 93 | 22 | 2 | 175 | 63 | 6.713 | 0 |
| 2364 | 93 | 22 | 2 | 175 | 63 | 6.714 | 0 |
| 2363 | 93 | 22 | 2 | 175 | 63 | 6.715 | 0 |
| 2362 | 93 | 22 | 2 | 175 | 63 | 6.716 | 0 |
| 2361 | 93 | 22 | 2 | 175 | 63 | 6.717 | 0 |
| 2360 | 93 | 22 | 2 | 175 | 63 | 6.718 | 0 |
| 2345 | 93 | 22 | 2 | 175 | 63 | 6.719 | 1 |
| 2344 | 93 | 22 | 2 | 175 | 63 | 6.72 | 0 |
| 3044 | 104 | 26 | 2 | 200 | 42 | 6.72 | 0 |
| 2841 | 93 | 22 | 2 | 175 | 62 | 6.721 | 1 |
| 2863 | 93 | 22 | 2 | 175 | 62 | 6.722 | 0 |
| 3201 | 93 | 22 | 2 | 175 | 62 | 6.723 | 0 |
| 3436 | 93 | 22 | 2 | 175 | 62 | 6.724 | 0 |
| 2017 | 93 | 22 | 2 | 175 | 62 | 6.725 | 1 |
| 2050 | 93 | 22 | 2 | 175 | 62 | 6.726 | 1 |
| 2229 | 93 | 22 | 2 | 175 | 62 | 6.727 | 1 |
| 2276 | 93 | 22 | 2 | 175 | 62 | 6.728 | 1 |
| 2469 | 93 | 22 | 2 | 175 | 62 | 6.729 | 1 |
| 2564 | 93 | 22 | 2 | 175 | 62 | 6.73 | 0 |
| 3043 | 104 | 26 | 2 | 200 | 42 | 6.73 | 1 |
| 2674 | 93 | 22 | 2 | 175 | 62 | 6.731 | 1 |
| 2739 | 93 | 22 | 2 | 175 | 62 | 6.732 | 1 |
| 3264 | 93 | 22 | 2 | 175 | 62 | 6.733 | 0 |
| 3263 | 93 | 22 | 2 | 175 | 62 | 6.734 | 0 |
| 3262 | 93 | 22 | 2 | 175 | 62 | 6.735 | 0 |
| 3261 | 93 | 22 | 2 | 175 | 62 | 6.736 | 0 |
| 3260 | 93 | 22 | 2 | 175 | 62 | 6.737 | 1 |
| 3259 | 93 | 22 | 2 | 175 | 62 | 6.738 | 0 |
| 3258 | 93 | 22 | 2 | 175 | 62 | 6.739 | 0 |

| | | | | | | | |
|---|---|---|---|---|---|---|---|
| 3257 | 93 | 22 | 2 | 175 | 62 | 6.74 | 1 |
| 3042 | 104 | 26 | 2 | 200 | 42 | 6.74 | 0 |
| 2254 | 96 | 23 | 2 | 175 | 55 | 6.741 | 0 |
| 2462 | 96 | 23 | 2 | 175 | 55 | 6.742 | 0 |
| 2505 | 96 | 23 | 2 | 175 | 55 | 6.743 | 0 |
| 2580 | 96 | 23 | 2 | 175 | 55 | 6.744 | 0 |
| 2732 | 96 | 23 | 2 | 175 | 55 | 6.745 | 0 |
| 3493 | 93 | 22 | 2 | 175 | 63 | 6.746 | 0 |
| 3516 | 93 | 22 | 2 | 175 | 63 | 6.747 | 0 |
| 3539 | 93 | 22 | 2 | 175 | 63 | 6.748 | 0 |
| 3492 | 93 | 22 | 2 | 175 | 62 | 6.749 | 0 |
| 3515 | 93 | 22 | 2 | 175 | 62 | 6.75 | 0 |
| 3041 | 104 | 26 | 2 | 200 | 42 | 6.75 | 0 |
| 3538 | 93 | 22 | 2 | 175 | 62 | 6.751 | 0 |
| 3040 | 104 | 26 | 2 | 200 | 42 | 6.76 | 0 |
| 3039 | 104 | 26 | 2 | 200 | 42 | 6.77 | 1 |
| 2419 | 96 | 23 | 2 | 180 | 55 | 6.778 | 0 |
| 2411 | 96 | 23 | 2 | 180 | 55 | 6.779 | 1 |
| 2410 | 96 | 23 | 2 | 180 | 55 | 6.78 | 0 |
| 3038 | 104 | 26 | 2 | 200 | 42 | 6.78 | 0 |
| 2330 | 96 | 23 | 2 | 180 | 55 | 6.781 | 0 |
| 2329 | 96 | 23 | 2 | 180 | 55 | 6.782 | 1 |
| 2328 | 96 | 23 | 2 | 180 | 55 | 6.783 | 0 |
| 2318 | 96 | 23 | 2 | 180 | 55 | 6.784 | 0 |
| 2317 | 96 | 23 | 2 | 180 | 55 | 6.785 | 0 |
| 2308 | 96 | 23 | 2 | 180 | 55 | 6.786 | 0 |
| 2307 | 96 | 23 | 2 | 180 | 55 | 6.787 | 0 |
| 2306 | 96 | 23 | 2 | 180 | 55 | 6.788 | 0 |
| 2305 | 96 | 23 | 2 | 180 | 55 | 6.789 | 1 |
| 2304 | 96 | 23 | 2 | 180 | 55 | 6.79 | 0 |
| 3037 | 104 | 26 | 2 | 200 | 42 | 6.79 | 1 |
| 3433 | 95 | 23 | 2 | 180 | 59 | 6.791 | 0 |
| 2012 | 95 | 23 | 2 | 180 | 59 | 6.792 | 0 |
| 2042 | 95 | 23 | 2 | 180 | 59 | 6.793 | 0 |
| 2226 | 95 | 23 | 2 | 180 | 59 | 6.794 | 0 |
| 2258 | 95 | 23 | 2 | 180 | 59 | 6.795 | 1 |
| 2466 | 95 | 23 | 2 | 180 | 59 | 6.796 | 1 |
| 2509 | 95 | 23 | 2 | 180 | 59 | 6.797 | 0 |
| 2642 | 95 | 23 | 2 | 180 | 59 | 6.798 | 1 |
| 2736 | 95 | 23 | 2 | 180 | 59 | 6.799 | 1 |
| 3310 | 95 | 23 | 2 | 180 | 59 | 6.8 | 0 |
| 3036 | 104 | 26 | 2 | 200 | 42 | 6.8 | 0 |
| 3309 | 95 | 23 | 2 | 180 | 59 | 6.801 | 0 |
| 3308 | 95 | 23 | 2 | 180 | 59 | 6.802 | 0 |

| | | | | | | | |
|---|---|---|---|---|---|---|---|
| 3307 | 95 | 23 | 2 | 180 | 59 | 6.803 | 0 |
| 3306 | 95 | 23 | 2 | 180 | 59 | 6.804 | 1 |
| 3305 | 95 | 23 | 2 | 180 | 59 | 6.805 | 1 |
| 3304 | 95 | 23 | 2 | 180 | 59 | 6.806 | 1 |
| 3303 | 95 | 23 | 2 | 180 | 59 | 6.807 | 0 |
| 3302 | 95 | 23 | 2 | 180 | 59 | 6.808 | 1 |
| 3301 | 95 | 23 | 2 | 180 | 59 | 6.809 | 1 |
| 3300 | 95 | 23 | 2 | 180 | 59 | 6.81 | 0 |
| 3371 | 104 | 26 | 2 | 200 | 42 | 6.81 | 0 |
| 3299 | 95 | 23 | 2 | 180 | 59 | 6.811 | 0 |
| 3298 | 95 | 23 | 2 | 180 | 59 | 6.812 | 1 |
| 3297 | 95 | 23 | 2 | 180 | 59 | 6.813 | 0 |
| 2134 | 95 | 23 | 2 | 180 | 59 | 6.814 | 0 |
| 2133 | 95 | 23 | 2 | 180 | 59 | 6.815 | 0 |
| 2132 | 95 | 23 | 2 | 180 | 59 | 6.816 | 0 |
| 2131 | 95 | 23 | 2 | 180 | 59 | 6.817 | 0 |
| 2130 | 95 | 23 | 2 | 180 | 59 | 6.818 | 1 |
| 2129 | 95 | 23 | 2 | 180 | 59 | 6.819 | 0 |
| 2128 | 95 | 23 | 2 | 180 | 59 | 6.82 | 0 |
| 3370 | 104 | 26 | 2 | 200 | 42 | 6.82 | 1 |
| 2127 | 95 | 23 | 2 | 180 | 59 | 6.821 | 1 |
| 2126 | 95 | 23 | 2 | 180 | 59 | 6.822 | 0 |
| 2125 | 95 | 23 | 2 | 180 | 59 | 6.823 | 0 |
| 2124 | 95 | 23 | 2 | 180 | 59 | 6.824 | 0 |
| 2123 | 95 | 23 | 2 | 180 | 59 | 6.825 | 0 |
| 2122 | 95 | 23 | 2 | 180 | 59 | 6.826 | 1 |
| 2121 | 95 | 23 | 2 | 180 | 59 | 6.827 | 1 |
| 2120 | 95 | 23 | 2 | 180 | 59 | 6.828 | 1 |
| 2119 | 95 | 23 | 2 | 180 | 59 | 6.829 | 1 |
| 2763 | 95 | 23 | 2 | 180 | 59 | 6.83 | 0 |
| 3369 | 104 | 26 | 2 | 200 | 42 | 6.83 | 0 |
| 2762 | 95 | 23 | 2 | 180 | 59 | 6.831 | 0 |
| 2761 | 95 | 23 | 2 | 180 | 59 | 6.832 | 0 |
| 2760 | 95 | 23 | 2 | 180 | 59 | 6.833 | 0 |
| 2759 | 95 | 23 | 2 | 180 | 59 | 6.834 | 0 |
| 2758 | 95 | 23 | 2 | 180 | 59 | 6.835 | 0 |
| 2706 | 95 | 23 | 2 | 180 | 59 | 6.836 | 0 |
| 2705 | 95 | 23 | 2 | 180 | 59 | 6.837 | 0 |
| 2704 | 95 | 23 | 2 | 180 | 59 | 6.838 | 0 |
| 2703 | 95 | 23 | 2 | 180 | 59 | 6.839 | 1 |
| 2702 | 95 | 23 | 2 | 180 | 59 | 6.84 | 0 |
| 3368 | 104 | 26 | 2 | 200 | 42 | 6.84 | 0 |
| 2701 | 95 | 23 | 2 | 180 | 59 | 6.841 | 1 |
| 2594 | 95 | 23 | 2 | 180 | 59 | 6.842 | 1 |

| | | | | | | | |
|---|---|---|---|---|---|---|---|
| 2593 | 95 | 23 | 2 | 180 | 59 | 6.843 | 1 |
| 2586 | 95 | 23 | 2 | 180 | 59 | 6.844 | 0 |
| 2585 | 95 | 23 | 2 | 180 | 59 | 6.845 | 1 |
| 3054 | 95 | 23 | 2 | 180 | 59 | 6.846 | 0 |
| 3053 | 95 | 23 | 2 | 180 | 59 | 6.847 | 0 |
| 3052 | 95 | 23 | 2 | 180 | 59 | 6.848 | 0 |
| 3051 | 95 | 23 | 2 | 180 | 59 | 6.849 | 1 |
| 3050 | 95 | 23 | 2 | 180 | 59 | 6.85 | 0 |
| 3367 | 104 | 26 | 2 | 200 | 42 | 6.85 | 1 |
| 3049 | 95 | 23 | 2 | 180 | 59 | 6.851 | 0 |
| 2925 | 95 | 23 | 2 | 180 | 59 | 6.852 | 0 |
| 2924 | 95 | 23 | 2 | 180 | 59 | 6.853 | 0 |
| 2923 | 95 | 23 | 2 | 180 | 59 | 6.854 | 0 |
| 2922 | 95 | 23 | 2 | 180 | 59 | 6.855 | 0 |
| 2921 | 95 | 23 | 2 | 180 | 59 | 6.856 | 0 |
| 2920 | 95 | 23 | 2 | 180 | 59 | 6.857 | 0 |
| 2768 | 95 | 23 | 2 | 180 | 59 | 6.858 | 0 |
| 2725 | 95 | 23 | 2 | 180 | 59 | 6.859 | 0 |
| 2724 | 95 | 23 | 2 | 180 | 59 | 6.86 | 0 |
| 3366 | 104 | 26 | 2 | 200 | 42 | 6.86 | 1 |
| 2723 | 95 | 23 | 2 | 180 | 59 | 6.861 | 0 |
| 2837 | 95 | 23 | 2 | 180 | 58 | 6.862 | 0 |
| 2859 | 95 | 23 | 2 | 180 | 58 | 6.863 | 0 |
| 3197 | 95 | 23 | 2 | 180 | 58 | 6.864 | 0 |
| 3432 | 95 | 23 | 2 | 180 | 58 | 6.865 | 0 |
| 2011 | 95 | 23 | 2 | 180 | 58 | 6.866 | 0 |
| 2040 | 95 | 23 | 2 | 180 | 58 | 6.867 | 0 |
| 2081 | 95 | 23 | 2 | 180 | 58 | 6.868 | 0 |
| 2257 | 95 | 23 | 2 | 180 | 58 | 6.869 | 0 |
| 2465 | 95 | 23 | 2 | 180 | 58 | 6.87 | 0 |
| 3365 | 104 | 26 | 2 | 200 | 42 | 6.87 | 0 |
| 2508 | 95 | 23 | 2 | 180 | 58 | 6.871 | 0 |
| 2641 | 95 | 23 | 2 | 180 | 58 | 6.872 | 1 |
| 2735 | 95 | 23 | 2 | 180 | 58 | 6.873 | 1 |
| 2162 | 95 | 23 | 2 | 180 | 58 | 6.874 | 1 |
| 2161 | 95 | 23 | 2 | 180 | 58 | 6.875 | 1 |
| 2160 | 95 | 23 | 2 | 180 | 58 | 6.876 | 0 |
| 2159 | 95 | 23 | 2 | 180 | 58 | 6.877 | 0 |
| 2158 | 95 | 23 | 2 | 180 | 58 | 6.878 | 0 |
| 2157 | 95 | 23 | 2 | 180 | 58 | 6.879 | 0 |
| 2156 | 95 | 23 | 2 | 180 | 58 | 6.88 | 0 |
| 3364 | 104 | 26 | 2 | 200 | 42 | 6.88 | 0 |
| 2155 | 95 | 23 | 2 | 180 | 58 | 6.881 | 0 |
| 2154 | 95 | 23 | 2 | 180 | 58 | 6.882 | 0 |

| | | | | | | | |
|---|---|---|---|---|---|---|---|
| 2153 | 95 | 23 | 2 | 180 | 58 | 6.883 | 1 |
| 2152 | 95 | 23 | 2 | 180 | 58 | 6.884 | 1 |
| 2151 | 95 | 23 | 2 | 180 | 58 | 6.885 | 0 |
| 2150 | 95 | 23 | 2 | 180 | 58 | 6.886 | 0 |
| 3489 | 95 | 23 | 2 | 180 | 59 | 6.887 | 0 |
| 3512 | 95 | 23 | 2 | 180 | 59 | 6.888 | 0 |
| 3535 | 95 | 23 | 2 | 180 | 59 | 6.889 | 0 |
| 3546 | 95 | 23 | 2 | 180 | 59 | 6.89 | 0 |
| 3363 | 104 | 26 | 2 | 200 | 42 | 6.89 | 0 |
| 3545 | 95 | 23 | 2 | 180 | 59 | 6.891 | 0 |
| 3488 | 95 | 23 | 2 | 180 | 58 | 6.892 | 0 |
| 3511 | 95 | 23 | 2 | 180 | 58 | 6.893 | 0 |
| 3534 | 95 | 23 | 2 | 180 | 58 | 6.894 | 0 |
| 3554 | 95 | 23 | 2 | 180 | 58 | 6.895 | 0 |
| 3553 | 95 | 23 | 2 | 180 | 58 | 6.896 | 0 |
| 3552 | 95 | 23 | 2 | 180 | 58 | 6.897 | 0 |
| 3551 | 95 | 23 | 2 | 180 | 58 | 6.898 | 1 |
| 3550 | 95 | 23 | 2 | 180 | 58 | 6.899 | 1 |
| 3549 | 95 | 23 | 2 | 180 | 58 | 6.9 | 0 |
| 3362 | 104 | 26 | 2 | 200 | 42 | 6.9 | 1 |
| 3548 | 95 | 23 | 2 | 180 | 58 | 6.901 | 0 |
| 3547 | 95 | 23 | 2 | 180 | 58 | 6.902 | 0 |
| 3396 | 104 | 26 | 2 | 200 | 42 | 6.91 | 0 |
| 3395 | 104 | 26 | 2 | 200 | 42 | 6.92 | 0 |
| 2149 | 95 | 23 | 2 | 185 | 58 | 6.923 | 0 |
| 2148 | 95 | 23 | 2 | 185 | 58 | 6.924 | 0 |
| 2147 | 95 | 23 | 2 | 185 | 58 | 6.925 | 0 |
| 3022 | 95 | 23 | 2 | 185 | 58 | 6.926 | 0 |
| 3021 | 95 | 23 | 2 | 185 | 58 | 6.927 | 0 |
| 3020 | 95 | 23 | 2 | 185 | 58 | 6.928 | 0 |
| 2785 | 95 | 23 | 2 | 185 | 58 | 6.929 | 0 |
| 2784 | 95 | 23 | 2 | 185 | 58 | 6.93 | 1 |
| 3394 | 104 | 26 | 2 | 200 | 42 | 6.93 | 0 |
| 2783 | 95 | 23 | 2 | 185 | 58 | 6.931 | 0 |
| 2782 | 95 | 23 | 2 | 185 | 58 | 6.932 | 0 |
| 2781 | 95 | 23 | 2 | 185 | 58 | 6.933 | 1 |
| 2780 | 95 | 23 | 2 | 185 | 58 | 6.934 | 0 |
| 2778 | 95 | 23 | 2 | 185 | 58 | 6.936 | 0 |
| 2765 | 95 | 23 | 2 | 185 | 58 | 6.937 | 0 |
| 2764 | 95 | 23 | 2 | 185 | 58 | 6.938 | 0 |
| 3321 | 95 | 23 | 2 | 185 | 58 | 6.939 | 0 |
| 3320 | 95 | 23 | 2 | 185 | 58 | 6.94 | 0 |
| 3393 | 104 | 26 | 2 | 200 | 42 | 6.94 | 0 |
| 3319 | 95 | 23 | 2 | 185 | 58 | 6.941 | 1 |

| | | | | | | | |
|---|---|---|---|---|---|---|---|
| 3318 | 95 | 23 | 2 | 185 | 58 | 6.942 | 0 |
| 3317 | 95 | 23 | 2 | 185 | 58 | 6.943 | 1 |
| 3316 | 95 | 23 | 2 | 185 | 58 | 6.944 | 0 |
| 3064 | 95 | 23 | 2 | 185 | 58 | 6.945 | 0 |
| 3063 | 95 | 23 | 2 | 185 | 58 | 6.946 | 1 |
| 3062 | 95 | 23 | 2 | 185 | 58 | 6.947 | 1 |
| 3061 | 95 | 23 | 2 | 185 | 58 | 6.948 | 0 |
| 3060 | 95 | 23 | 2 | 185 | 58 | 6.949 | 0 |
| 3059 | 95 | 23 | 2 | 185 | 58 | 6.95 | 0 |
| 3392 | 104 | 26 | 2 | 200 | 42 | 6.95 | 0 |
| 3058 | 95 | 23 | 2 | 185 | 58 | 6.951 | 0 |
| 3057 | 95 | 23 | 2 | 185 | 58 | 6.952 | 0 |
| 3056 | 95 | 23 | 2 | 185 | 58 | 6.953 | 0 |
| 3055 | 95 | 23 | 2 | 185 | 58 | 6.954 | 0 |
| 2836 | 95 | 23 | 2 | 185 | 57 | 6.955 | 0 |
| 2858 | 95 | 23 | 2 | 185 | 57 | 6.956 | 0 |
| 3196 | 95 | 23 | 2 | 185 | 57 | 6.957 | 0 |
| 3431 | 95 | 23 | 2 | 185 | 57 | 6.958 | 0 |
| 2010 | 95 | 23 | 2 | 185 | 57 | 6.959 | 0 |
| 2039 | 95 | 23 | 2 | 185 | 57 | 6.96 | 0 |
| 3391 | 104 | 26 | 2 | 200 | 42 | 6.96 | 0 |
| 2080 | 95 | 23 | 2 | 185 | 57 | 6.961 | 1 |
| 2256 | 95 | 23 | 2 | 185 | 57 | 6.962 | 1 |
| 2464 | 95 | 23 | 2 | 185 | 57 | 6.963 | 0 |
| 2507 | 95 | 23 | 2 | 185 | 57 | 6.964 | 0 |
| 2582 | 95 | 23 | 2 | 185 | 57 | 6.965 | 0 |
| 2734 | 95 | 23 | 2 | 185 | 57 | 6.966 | 0 |
| 2530 | 94 | 23 | 2 | 185 | 62 | 6.967 | 1 |
| 2529 | 94 | 23 | 2 | 185 | 62 | 6.968 | 0 |
| 2528 | 94 | 23 | 2 | 185 | 62 | 6.969 | 0 |
| 2527 | 94 | 23 | 2 | 185 | 62 | 6.97 | 0 |
| 3390 | 104 | 26 | 2 | 200 | 42 | 6.97 | 0 |
| 2479 | 94 | 23 | 2 | 185 | 62 | 6.971 | 0 |
| 2478 | 94 | 23 | 2 | 185 | 62 | 6.972 | 1 |
| 2477 | 94 | 23 | 2 | 185 | 62 | 6.973 | 1 |
| 2476 | 94 | 23 | 2 | 185 | 62 | 6.974 | 0 |
| 2452 | 94 | 23 | 2 | 185 | 62 | 6.975 | 1 |
| 2426 | 94 | 23 | 2 | 185 | 62 | 6.976 | 1 |
| 2425 | 94 | 23 | 2 | 185 | 62 | 6.977 | 0 |
| 2418 | 94 | 23 | 2 | 185 | 62 | 6.978 | 0 |
| 2417 | 94 | 23 | 2 | 185 | 62 | 6.979 | 0 |
| 2416 | 94 | 23 | 2 | 185 | 62 | 6.98 | 0 |
| 3389 | 104 | 26 | 2 | 200 | 42 | 6.98 | 0 |
| 2415 | 94 | 23 | 2 | 185 | 62 | 6.981 | 0 |

| | | | | | | | |
|---|---|---|---|---|---|---|---|
| 2414 | 94 | 23 | 2 | 185 | 62 | 6.982 | 0 |
| 2840 | 94 | 23 | 2 | 185 | 61 | 6.983 | 0 |
| 2862 | 94 | 23 | 2 | 185 | 61 | 6.984 | 0 |
| 3200 | 94 | 23 | 2 | 185 | 61 | 6.985 | 0 |
| 3435 | 94 | 23 | 2 | 185 | 61 | 6.986 | 0 |
| 2014 | 94 | 23 | 2 | 185 | 61 | 6.987 | 0 |
| 2044 | 94 | 23 | 2 | 185 | 61 | 6.988 | 0 |
| 2228 | 94 | 23 | 2 | 185 | 61 | 6.989 | 0 |
| 2275 | 94 | 23 | 2 | 185 | 61 | 6.99 | 0 |
| 3388 | 104 | 26 | 2 | 200 | 42 | 6.99 | 0 |
| 2468 | 94 | 23 | 2 | 185 | 61 | 6.991 | 1 |
| 2563 | 94 | 23 | 2 | 185 | 61 | 6.992 | 1 |
| 2630 | 98 | 24 | 2 | 185 | 51 | 6.993 | 0 |
| 2629 | 98 | 24 | 2 | 185 | 51 | 6.994 | 0 |
| 2628 | 98 | 24 | 2 | 185 | 51 | 6.995 | 1 |
| 2627 | 98 | 24 | 2 | 185 | 51 | 6.996 | 0 |
| 2603 | 98 | 24 | 2 | 185 | 51 | 6.997 | 1 |
| 2589 | 98 | 24 | 2 | 185 | 51 | 6.998 | 1 |
| 2588 | 98 | 24 | 2 | 185 | 51 | 6.999 | 1 |
| 2587 | 98 | 24 | 2 | 185 | 51 | 7 | 1 |
| 3387 | 104 | 26 | 2 | 200 | 42 | 7 | 1 |
| 2526 | 98 | 24 | 2 | 185 | 51 | 7.001 | 0 |
| 1926 | 89 | 30 | 2 | 205 | 29 | 7.001 | 0 |
| 2028 | 91 | 31 | 2 | 205 | 70 | 7.0011 | 0 |
| 2525 | 98 | 24 | 2 | 185 | 51 | 7.002 | 0 |
| 2524 | 98 | 24 | 2 | 185 | 51 | 7.003 | 0 |
| 2390 | 98 | 24 | 2 | 185 | 51 | 7.004 | 1 |
| 2389 | 98 | 24 | 2 | 185 | 51 | 7.005 | 0 |
| 3189 | 98 | 24 | 2 | 185 | 50 | 7.006 | 0 |
| 3212 | 98 | 24 | 2 | 185 | 50 | 7.007 | 0 |
| 3447 | 98 | 24 | 2 | 185 | 50 | 7.008 | 1 |
| 1838 | 98 | 24 | 2 | 185 | 50 | 7.009 | 1 |
| 1927 | 89 | 30 | 2 | 205 | 29 | 7.009 | 0 |
| 2032 | 98 | 24 | 2 | 185 | 50 | 7.01 | 0 |
| 3386 | 104 | 26 | 2 | 200 | 42 | 7.01 | 1 |
| 2064 | 98 | 24 | 2 | 185 | 50 | 7.011 | 1 |
| 1840 | 89 | 30 | 2 | 205 | 30 | 7.0116 | 1 |
| 2249 | 98 | 24 | 2 | 185 | 50 | 7.012 | 1 |
| 2292 | 98 | 24 | 2 | 185 | 50 | 7.013 | 1 |
| 2500 | 98 | 24 | 2 | 185 | 50 | 7.014 | 1 |
| 2575 | 98 | 24 | 2 | 185 | 50 | 7.015 | 1 |
| 1859 | 89 | 30 | 2 | 205 | 30 | 7.01555 | 1 |
| 2698 | 98 | 24 | 2 | 185 | 50 | 7.016 | 1 |
| 2954 | 98 | 24 | 2 | 185 | 50 | 7.017 | 1 |

| | | | | | | | |
|---|---|---|---|---|---|---|---|
| 2953 | 98 | 24 | 2 | 185 | 50 | 7.018 | 1 |
| 2952 | 98 | 24 | 2 | 185 | 50 | 7.019 | 0 |
| 1940 | 89 | 30 | 2 | 205 | 30 | 7.0195 | 1 |
| 2951 | 98 | 24 | 2 | 185 | 50 | 7.02 | 0 |
| 3385 | 104 | 26 | 2 | 200 | 42 | 7.02 | 1 |
| 2950 | 98 | 24 | 2 | 185 | 50 | 7.021 | 1 |
| 2949 | 98 | 24 | 2 | 185 | 50 | 7.022 | 1 |
| 2948 | 98 | 24 | 2 | 185 | 50 | 7.023 | 0 |
| 3111 | 90 | 30 | 2 | 205 | 56 | 7.02345 | 1 |
| 2947 | 98 | 24 | 2 | 185 | 50 | 7.024 | 1 |
| 2946 | 98 | 24 | 2 | 185 | 50 | 7.025 | 1 |
| 2945 | 98 | 24 | 2 | 185 | 50 | 7.026 | 1 |
| 2944 | 98 | 24 | 2 | 185 | 50 | 7.027 | 1 |
| 3112 | 90 | 30 | 2 | 205 | 56 | 7.0274 | 0 |
| 2943 | 98 | 24 | 2 | 185 | 50 | 7.028 | 1 |
| 2942 | 98 | 24 | 2 | 185 | 50 | 7.029 | 0 |
| 2941 | 98 | 24 | 2 | 185 | 50 | 7.03 | 0 |
| 3384 | 104 | 26 | 2 | 200 | 42 | 7.03 | 0 |
| 2895 | 98 | 24 | 2 | 185 | 50 | 7.031 | 0 |
| 3113 | 90 | 30 | 2 | 205 | 56 | 7.03135 | 1 |
| 2894 | 98 | 24 | 2 | 185 | 50 | 7.032 | 1 |
| 2893 | 98 | 24 | 2 | 185 | 50 | 7.033 | 1 |
| 2892 | 98 | 24 | 2 | 185 | 50 | 7.034 | 0 |
| 2891 | 98 | 24 | 2 | 185 | 50 | 7.035 | 0 |
| 3114 | 90 | 30 | 2 | 205 | 56 | 7.0353 | 0 |
| 2890 | 98 | 24 | 2 | 185 | 50 | 7.036 | 0 |
| 2889 | 98 | 24 | 2 | 185 | 50 | 7.037 | 0 |
| 2303 | 97 | 24 | 2 | 185 | 55 | 7.038 | 0 |
| 2019 | 97 | 24 | 2 | 185 | 55 | 7.039 | 0 |
| 3115 | 90 | 30 | 2 | 205 | 56 | 7.03925 | 0 |
| 2015 | 97 | 24 | 2 | 185 | 55 | 7.04 | 1 |
| 3383 | 104 | 26 | 2 | 200 | 42 | 7.04 | 0 |
| 1974 | 97 | 24 | 2 | 185 | 55 | 7.041 | 1 |
| 1898 | 97 | 24 | 2 | 185 | 55 | 7.042 | 0 |
| 1857 | 97 | 24 | 2 | 185 | 55 | 7.043 | 1 |
| 3116 | 90 | 30 | 2 | 205 | 56 | 7.0432 | 1 |
| 1829 | 97 | 24 | 2 | 185 | 55 | 7.044 | 1 |
| 3487 | 95 | 23 | 2 | 185 | 57 | 7.045 | 1 |
| 3510 | 95 | 23 | 2 | 185 | 57 | 7.046 | 0 |
| 3533 | 95 | 23 | 2 | 185 | 57 | 7.047 | 1 |
| 3117 | 90 | 30 | 2 | 205 | 56 | 7.04715 | 1 |
| 3491 | 94 | 23 | 2 | 185 | 61 | 7.048 | 1 |
| 3514 | 94 | 23 | 2 | 185 | 61 | 7.049 | 0 |
| 3537 | 94 | 23 | 2 | 185 | 61 | 7.05 | 0 |

| | | | | | | | |
|---|---|---|---|---|---|---|---|
| 3382 | 104 | 26 | 2 | 200 | 42 | 7.05 | 0 |
| 3503 | 98 | 24 | 2 | 185 | 50 | 7.051 | 0 |
| 3118 | 90 | 30 | 2 | 205 | 56 | 7.0511 | 0 |
| 3526 | 98 | 24 | 2 | 185 | 50 | 7.052 | 1 |
| 3119 | 90 | 30 | 2 | 205 | 56 | 7.05505 | 1 |
| 3120 | 90 | 30 | 2 | 205 | 56 | 7.059 | 1 |
| 3381 | 104 | 26 | 2 | 200 | 42 | 7.06 | 1 |
| 3121 | 90 | 30 | 2 | 205 | 56 | 7.06295 | 1 |
| 3122 | 90 | 30 | 2 | 205 | 56 | 7.0669 | 0 |
| 3380 | 104 | 26 | 2 | 200 | 42 | 7.07 | 1 |
| 3123 | 90 | 30 | 2 | 205 | 56 | 7.07085 | 1 |
| 3124 | 90 | 30 | 2 | 205 | 56 | 7.0748 | 0 |
| 3125 | 90 | 30 | 2 | 205 | 56 | 7.07875 | 0 |
| 3379 | 104 | 26 | 2 | 200 | 42 | 7.08 | 0 |
| 3126 | 90 | 30 | 2 | 205 | 55 | 7.0827 | 0 |
| 3127 | 90 | 30 | 2 | 205 | 55 | 7.08665 | 0 |
| 3378 | 104 | 26 | 2 | 200 | 42 | 7.09 | 0 |
| 3128 | 90 | 30 | 2 | 205 | 55 | 7.0906 | 0 |
| 3356 | 97 | 24 | 2 | 190 | 55 | 7.093 | 0 |
| 3355 | 97 | 24 | 2 | 190 | 55 | 7.094 | 0 |
| 3129 | 90 | 30 | 2 | 205 | 55 | 7.09455 | 1 |
| 3354 | 97 | 24 | 2 | 190 | 55 | 7.095 | 0 |
| 3193 | 97 | 24 | 2 | 190 | 54 | 7.096 | 1 |
| 3428 | 97 | 24 | 2 | 190 | 54 | 7.097 | 0 |
| 1918 | 97 | 24 | 2 | 190 | 54 | 7.098 | 0 |
| 3130 | 90 | 30 | 2 | 205 | 55 | 7.0985 | 0 |
| 2036 | 97 | 24 | 2 | 190 | 54 | 7.099 | 0 |
| 2068 | 97 | 24 | 2 | 190 | 54 | 7.1 | 0 |
| 3377 | 104 | 26 | 2 | 200 | 42 | 7.1 | 1 |
| 2253 | 97 | 24 | 2 | 190 | 54 | 7.101 | 0 |
| 2430 | 97 | 24 | 2 | 190 | 54 | 7.102 | 0 |
| 3131 | 90 | 30 | 2 | 205 | 55 | 7.10245 | 0 |
| 2504 | 97 | 24 | 2 | 190 | 54 | 7.103 | 1 |
| 2579 | 97 | 24 | 2 | 190 | 54 | 7.104 | 1 |
| 2731 | 97 | 24 | 2 | 190 | 54 | 7.105 | 0 |
| 2612 | 97 | 24 | 2 | 190 | 54 | 7.106 | 0 |
| 3132 | 90 | 30 | 2 | 205 | 55 | 7.1064 | 0 |
| 2611 | 97 | 24 | 2 | 190 | 54 | 7.107 | 0 |
| 2610 | 97 | 24 | 2 | 190 | 54 | 7.108 | 0 |
| 2609 | 97 | 24 | 2 | 190 | 54 | 7.109 | 0 |
| 2608 | 97 | 24 | 2 | 190 | 54 | 7.11 | 0 |
| 3376 | 104 | 26 | 2 | 200 | 42 | 7.11 | 0 |
| 3133 | 90 | 30 | 2 | 205 | 55 | 7.11035 | 0 |
| 2607 | 97 | 24 | 2 | 190 | 54 | 7.111 | 0 |

| | | | | | | | |
|---|---|---|---|---|---|---|---|
| 2606 | 97 | 24 | 2 | 190 | 54 | 7.112 | 0 |
| 2605 | 97 | 24 | 2 | 190 | 54 | 7.113 | 0 |
| 2604 | 97 | 24 | 2 | 190 | 54 | 7.114 | 1 |
| 3134 | 90 | 30 | 2 | 205 | 55 | 7.1143 | 0 |
| 2424 | 97 | 24 | 2 | 190 | 54 | 7.115 | 1 |
| 2423 | 97 | 24 | 2 | 190 | 54 | 7.116 | 1 |
| 2422 | 97 | 24 | 2 | 190 | 54 | 7.117 | 1 |
| 2421 | 97 | 24 | 2 | 190 | 54 | 7.118 | 0 |
| 3135 | 90 | 30 | 2 | 205 | 55 | 7.11825 | 1 |
| 2420 | 97 | 24 | 2 | 190 | 54 | 7.119 | 1 |
| 2260 | 97 | 24 | 2 | 190 | 54 | 7.12 | 1 |
| 3375 | 104 | 26 | 2 | 200 | 42 | 7.12 | 1 |
| 2208 | 97 | 24 | 2 | 190 | 54 | 7.121 | 1 |
| 2199 | 97 | 24 | 2 | 190 | 54 | 7.122 | 1 |
| 3136 | 90 | 30 | 2 | 205 | 55 | 7.1222 | 1 |
| 2189 | 97 | 24 | 2 | 190 | 54 | 7.123 | 1 |
| 2188 | 97 | 24 | 2 | 190 | 54 | 7.124 | 1 |
| 2187 | 97 | 24 | 2 | 190 | 54 | 7.125 | 1 |
| 2166 | 97 | 24 | 2 | 190 | 54 | 7.126 | 1 |
| 3137 | 90 | 30 | 2 | 205 | 55 | 7.12615 | 1 |
| 2145 | 97 | 24 | 2 | 190 | 54 | 7.127 | 1 |
| 2144 | 97 | 24 | 2 | 190 | 54 | 7.128 | 1 |
| 2143 | 97 | 24 | 2 | 190 | 54 | 7.129 | 1 |
| 2135 | 97 | 24 | 2 | 190 | 54 | 7.13 | 1 |
| 3374 | 104 | 26 | 2 | 200 | 42 | 7.13 | 1 |
| 3138 | 90 | 30 | 2 | 205 | 55 | 7.1301 | 1 |
| 2041 | 97 | 24 | 2 | 190 | 54 | 7.131 | 0 |
| 2029 | 97 | 24 | 2 | 190 | 54 | 7.132 | 0 |
| 2027 | 97 | 24 | 2 | 190 | 54 | 7.133 | 0 |
| 3192 | 97 | 24 | 2 | 190 | 53 | 7.134 | 1 |
| 3139 | 90 | 30 | 2 | 205 | 55 | 7.13405 | 0 |
| 3427 | 97 | 24 | 2 | 190 | 53 | 7.135 | 0 |
| 1899 | 97 | 24 | 2 | 190 | 53 | 7.136 | 0 |
| 2035 | 97 | 24 | 2 | 190 | 53 | 7.137 | 1 |
| 3140 | 90 | 30 | 2 | 205 | 54 | 7.138 | 0 |
| 2067 | 97 | 24 | 2 | 190 | 53 | 7.138 | 0 |
| 2252 | 97 | 24 | 2 | 190 | 53 | 7.139 | 1 |
| 2347 | 97 | 24 | 2 | 190 | 53 | 7.14 | 1 |
| 3373 | 104 | 26 | 2 | 200 | 42 | 7.14 | 1 |
| 2503 | 97 | 24 | 2 | 190 | 53 | 7.141 | 1 |
| 3141 | 90 | 30 | 2 | 205 | 54 | 7.14195 | 0 |
| 2578 | 97 | 24 | 2 | 190 | 53 | 7.142 | 0 |
| 2730 | 97 | 24 | 2 | 190 | 53 | 7.143 | 0 |
| 2626 | 97 | 24 | 2 | 190 | 53 | 7.144 | 0 |

| | | | | | | | |
|---|---|---|---|---|---|---|---|
| 2625 | 97 | 24 | 2 | 190 | 53 | 7.145 | 0 |
| 3142 | 90 | 30 | 2 | 205 | 54 | 7.1459 | 0 |
| 2624 | 97 | 24 | 2 | 190 | 53 | 7.146 | 0 |
| 2623 | 97 | 24 | 2 | 190 | 53 | 7.147 | 0 |
| 2622 | 97 | 24 | 2 | 190 | 53 | 7.148 | 0 |
| 2621 | 97 | 24 | 2 | 190 | 53 | 7.149 | 0 |
| 3143 | 90 | 30 | 2 | 205 | 54 | 7.14985 | 0 |
| 2620 | 97 | 24 | 2 | 190 | 53 | 7.15 | 0 |
| 3372 | 104 | 26 | 2 | 200 | 42 | 7.15 | 0 |
| 2619 | 97 | 24 | 2 | 190 | 53 | 7.151 | 0 |
| 2618 | 97 | 24 | 2 | 190 | 53 | 7.152 | 0 |
| 2617 | 97 | 24 | 2 | 190 | 53 | 7.153 | 0 |
| 3144 | 90 | 30 | 2 | 205 | 54 | 7.1538 | 1 |
| 2616 | 97 | 24 | 2 | 190 | 53 | 7.154 | 1 |
| 2615 | 97 | 24 | 2 | 190 | 53 | 7.155 | 1 |
| 2614 | 97 | 24 | 2 | 190 | 53 | 7.156 | 1 |
| 2613 | 97 | 24 | 2 | 190 | 53 | 7.157 | 0 |
| 3145 | 90 | 30 | 2 | 205 | 54 | 7.15775 | 0 |
| 2336 | 97 | 24 | 2 | 190 | 53 | 7.158 | 0 |
| 2335 | 97 | 24 | 2 | 190 | 53 | 7.159 | 0 |
| 2334 | 97 | 24 | 2 | 190 | 53 | 7.16 | 0 |
| 3019 | 104 | 26 | 2 | 200 | 42 | 7.16 | 0 |
| 2333 | 97 | 24 | 2 | 190 | 53 | 7.161 | 0 |
| 3146 | 90 | 30 | 2 | 205 | 54 | 7.1617 | 0 |
| 2332 | 97 | 24 | 2 | 190 | 53 | 7.162 | 0 |
| 2309 | 97 | 24 | 2 | 190 | 53 | 7.163 | 0 |
| 2271 | 97 | 24 | 2 | 190 | 53 | 7.164 | 0 |
| 2270 | 97 | 24 | 2 | 190 | 53 | 7.165 | 0 |
| 3147 | 90 | 30 | 2 | 205 | 54 | 7.16565 | 0 |
| 2269 | 97 | 24 | 2 | 190 | 53 | 7.166 | 1 |
| 2268 | 97 | 24 | 2 | 190 | 53 | 7.167 | 1 |
| 2267 | 97 | 24 | 2 | 190 | 53 | 7.168 | 0 |
| 2266 | 97 | 24 | 2 | 190 | 53 | 7.169 | 0 |
| 3148 | 90 | 30 | 2 | 205 | 54 | 7.1696 | 0 |
| 2265 | 97 | 24 | 2 | 190 | 53 | 7.17 | 0 |
| 3018 | 104 | 26 | 2 | 200 | 42 | 7.17 | 0 |
| 2264 | 97 | 24 | 2 | 190 | 53 | 7.171 | 1 |
| 3191 | 97 | 24 | 2 | 190 | 52 | 7.172 | 0 |
| 3426 | 97 | 24 | 2 | 190 | 52 | 7.173 | 1 |
| 3149 | 90 | 30 | 2 | 205 | 54 | 7.17355 | 0 |
| 1860 | 97 | 24 | 2 | 190 | 52 | 7.174 | 0 |
| 2034 | 97 | 24 | 2 | 190 | 52 | 7.175 | 1 |
| 2066 | 97 | 24 | 2 | 190 | 52 | 7.176 | 0 |
| 2251 | 97 | 24 | 2 | 190 | 52 | 7.177 | 1 |

| | | | | | | | |
|---|---|---|---|---|---|---|---|
| 3150 | 90 | 30 | 2 | 205 | 54 | 7.1775 | 0 |
| 2183 | 96 | 24 | 2 | 190 | 57 | 7.178 | 0 |
| 2182 | 96 | 24 | 2 | 190 | 57 | 7.179 | 1 |
| 2181 | 96 | 24 | 2 | 190 | 57 | 7.18 | 0 |
| 3017 | 104 | 26 | 2 | 200 | 42 | 7.18 | 0 |
| 2180 | 96 | 24 | 2 | 190 | 57 | 7.181 | 1 |
| 3151 | 90 | 30 | 2 | 205 | 54 | 7.18145 | 0 |
| 2179 | 96 | 24 | 2 | 190 | 57 | 7.182 | 0 |
| 2178 | 96 | 24 | 2 | 190 | 57 | 7.183 | 0 |
| 2177 | 96 | 24 | 2 | 190 | 57 | 7.184 | 0 |
| 2176 | 96 | 24 | 2 | 190 | 57 | 7.185 | 0 |
| 3152 | 90 | 30 | 2 | 205 | 54 | 7.1854 | 0 |
| 2175 | 96 | 24 | 2 | 190 | 57 | 7.186 | 0 |
| 2174 | 96 | 24 | 2 | 190 | 57 | 7.187 | 0 |
| 2173 | 96 | 24 | 2 | 190 | 57 | 7.188 | 0 |
| 2172 | 96 | 24 | 2 | 190 | 57 | 7.189 | 0 |
| 3153 | 90 | 30 | 2 | 205 | 54 | 7.18935 | 0 |
| 2171 | 96 | 24 | 2 | 190 | 57 | 7.19 | 0 |
| 3016 | 104 | 26 | 2 | 200 | 42 | 7.19 | 0 |
| 2165 | 96 | 24 | 2 | 190 | 57 | 7.191 | 1 |
| 2164 | 96 | 24 | 2 | 190 | 57 | 7.192 | 1 |
| 2163 | 96 | 24 | 2 | 190 | 57 | 7.193 | 1 |
| 3154 | 90 | 30 | 2 | 205 | 53 | 7.1933 | 1 |
| 3337 | 96 | 24 | 2 | 190 | 57 | 7.194 | 1 |
| 3336 | 96 | 24 | 2 | 190 | 57 | 7.195 | 1 |
| 3335 | 96 | 24 | 2 | 190 | 57 | 7.196 | 1 |
| 3334 | 96 | 24 | 2 | 190 | 57 | 7.197 | 1 |
| 3155 | 90 | 30 | 2 | 205 | 53 | 7.19725 | 1 |
| 3333 | 96 | 24 | 2 | 190 | 57 | 7.198 | 1 |
| 3332 | 96 | 24 | 2 | 190 | 57 | 7.199 | 1 |
| 3331 | 96 | 24 | 2 | 190 | 57 | 7.2 | 1 |
| 3015 | 104 | 26 | 2 | 200 | 42 | 7.2 | 1 |
| 3330 | 96 | 24 | 2 | 190 | 57 | 7.201 | 1 |
| 3156 | 90 | 30 | 2 | 205 | 53 | 7.2012 | 1 |
| 3329 | 96 | 24 | 2 | 190 | 57 | 7.202 | 1 |
| 3328 | 96 | 24 | 2 | 190 | 57 | 7.203 | 1 |
| 3327 | 96 | 24 | 2 | 190 | 57 | 7.204 | 1 |
| 3326 | 96 | 24 | 2 | 190 | 57 | 7.205 | 0 |
| 3157 | 90 | 30 | 2 | 205 | 53 | 7.20515 | 0 |
| 3325 | 96 | 24 | 2 | 190 | 57 | 7.206 | 0 |
| 3324 | 96 | 24 | 2 | 190 | 57 | 7.207 | 1 |
| 3323 | 96 | 24 | 2 | 190 | 57 | 7.208 | 1 |
| 3322 | 96 | 24 | 2 | 190 | 57 | 7.209 | 1 |
| 2835 | 96 | 24 | 2 | 190 | 56 | 7.21 | 1 |

| | | | | | | | |
|---|---|---|---|---|---|---|---|
| 3014 | 104 | 26 | 2 | 200 | 42 | 7.21 | 1 |
| 2857 | 96 | 24 | 2 | 190 | 56 | 7.211 | 1 |
| 3430 | 96 | 24 | 2 | 190 | 56 | 7.213 | 1 |
| 3159 | 90 | 30 | 2 | 205 | 53 | 7.21305 | 1 |
| 1992 | 96 | 24 | 2 | 190 | 56 | 7.214 | 1 |
| 2038 | 96 | 24 | 2 | 190 | 56 | 7.215 | 1 |
| 2079 | 96 | 24 | 2 | 190 | 56 | 7.216 | 1 |
| 3160 | 90 | 30 | 2 | 205 | 53 | 7.217 | 1 |
| 2506 | 96 | 24 | 2 | 190 | 56 | 7.219 | 1 |
| 2581 | 96 | 24 | 2 | 190 | 56 | 7.22 | 1 |
| 3013 | 104 | 26 | 2 | 200 | 42 | 7.22 | 1 |
| 3161 | 90 | 30 | 2 | 205 | 53 | 7.22095 | 1 |
| 2733 | 96 | 24 | 2 | 190 | 56 | 7.221 | 1 |
| 2300 | 96 | 24 | 2 | 190 | 56 | 7.224 | 1 |
| 2219 | 96 | 24 | 2 | 190 | 56 | 7.226 | 1 |
| 2218 | 96 | 24 | 2 | 190 | 56 | 7.227 | 1 |
| 2217 | 96 | 24 | 2 | 190 | 56 | 7.228 | 1 |
| 3163 | 90 | 30 | 2 | 205 | 53 | 7.22885 | 1 |
| 2216 | 96 | 24 | 2 | 190 | 56 | 7.229 | 0 |
| 2211 | 96 | 24 | 2 | 190 | 56 | 7.23 | 0 |
| 2207 | 96 | 24 | 2 | 190 | 56 | 7.233 | 0 |
| 2204 | 96 | 24 | 2 | 190 | 56 | 7.236 | 0 |
| 3165 | 90 | 30 | 2 | 205 | 53 | 7.23675 | 0 |
| 2203 | 96 | 24 | 2 | 190 | 56 | 7.237 | 0 |
| 3353 | 96 | 24 | 2 | 190 | 56 | 7.238 | 0 |
| 3351 | 96 | 24 | 2 | 190 | 56 | 7.24 | 0 |
| 3011 | 104 | 26 | 2 | 200 | 42 | 7.24 | 0 |
| 3166 | 90 | 30 | 2 | 205 | 53 | 7.2407 | 0 |
| 3350 | 96 | 24 | 2 | 190 | 56 | 7.241 | 0 |
| 3348 | 96 | 24 | 2 | 190 | 56 | 7.243 | 0 |
| 3167 | 90 | 30 | 2 | 205 | 53 | 7.24465 | 0 |
| 3344 | 96 | 24 | 2 | 190 | 56 | 7.247 | 0 |
| 3343 | 96 | 24 | 2 | 190 | 56 | 7.248 | 0 |
| 3168 | 90 | 30 | 2 | 205 | 52 | 7.2486 | 0 |
| 3342 | 96 | 24 | 2 | 190 | 56 | 7.249 | 0 |
| 3339 | 96 | 24 | 2 | 190 | 56 | 7.252 | 0 |
| 3256 | 90 | 30 | 2 | 205 | 52 | 7.25255 | 0 |
| 3194 | 96 | 24 | 2 | 190 | 55 | 7.254 | 0 |
| 3429 | 96 | 24 | 2 | 190 | 55 | 7.255 | 0 |
| 1919 | 96 | 24 | 2 | 190 | 55 | 7.256 | 0 |
| 3448 | 90 | 30 | 2 | 205 | 52 | 7.2565 | 0 |
| 2037 | 96 | 24 | 2 | 190 | 55 | 7.257 | 0 |
| 2078 | 96 | 24 | 2 | 190 | 55 | 7.258 | 0 |
| 2668 | 101 | 25 | 2 | 190 | 41 | 7.259 | 0 |

| | | | | | | | |
|---|---|---|---|---|---|---|---|
| 3009 | 104 | 26 | 2 | 200 | 42 | 7.26 | 0 |
| 3449 | 90 | 30 | 2 | 205 | 52 | 7.26045 | 0 |
| 2666 | 101 | 25 | 2 | 190 | 41 | 7.261 | 0 |
| 3450 | 90 | 30 | 2 | 205 | 52 | 7.2644 | 0 |
| 2657 | 101 | 25 | 2 | 190 | 41 | 7.266 | 0 |
| 2656 | 101 | 25 | 2 | 190 | 41 | 7.267 | 0 |
| 2640 | 101 | 25 | 2 | 190 | 41 | 7.268 | 0 |
| 3008 | 104 | 26 | 2 | 200 | 42 | 7.27 | 0 |
| 2595 | 101 | 25 | 2 | 190 | 41 | 7.27 | 0 |
| 2461 | 101 | 25 | 2 | 190 | 41 | 7.271 | 0 |
| 2460 | 101 | 25 | 2 | 190 | 41 | 7.272 | 0 |
| 3452 | 90 | 30 | 2 | 205 | 52 | 7.2723 | 0 |
| 2451 | 101 | 25 | 2 | 190 | 41 | 7.273 | 0 |
| 2450 | 101 | 25 | 2 | 190 | 41 | 7.274 | 0 |
| 2449 | 101 | 25 | 2 | 190 | 41 | 7.275 | 0 |
| 2395 | 101 | 25 | 2 | 190 | 41 | 7.276 | 0 |
| 3453 | 90 | 30 | 2 | 205 | 52 | 7.27625 | 0 |
| 2394 | 101 | 25 | 2 | 190 | 41 | 7.277 | 0 |
| 2327 | 100 | 25 | 2 | 190 | 41 | 7.278 | 0 |
| 3007 | 104 | 26 | 2 | 200 | 42 | 7.28 | 0 |
| 2320 | 100 | 25 | 2 | 190 | 41 | 7.28 | 0 |
| 3454 | 90 | 30 | 2 | 205 | 52 | 7.2802 | 0 |
| 2319 | 100 | 25 | 2 | 190 | 41 | 7.281 | 0 |
| 2296 | 100 | 25 | 2 | 190 | 41 | 7.282 | 0 |
| 2294 | 100 | 25 | 2 | 190 | 41 | 7.283 | 0 |
| 2282 | 100 | 25 | 2 | 190 | 41 | 7.284 | 0 |
| 1850 | 87 | 29 | 2 | 205 | 34 | 7.28415 | 0 |
| 2245 | 100 | 25 | 2 | 190 | 41 | 7.285 | 0 |
| 1870 | 100 | 25 | 2 | 190 | 41 | 7.286 | 0 |
| 1869 | 100 | 25 | 2 | 190 | 41 | 7.287 | 0 |
| 1868 | 100 | 25 | 2 | 190 | 41 | 7.288 | 0 |
| 1924 | 87 | 29 | 2 | 205 | 34 | 7.2881 | 0 |
| 1866 | 100 | 25 | 2 | 190 | 41 | 7.289 | 0 |
| 1856 | 100 | 25 | 2 | 190 | 41 | 7.292 | 0 |
| 1821 | 88 | 29 | 2 | 205 | 31 | 7.29205 | 0 |
| 1855 | 100 | 25 | 2 | 190 | 41 | 7.293 | 0 |
| 1844 | 100 | 25 | 2 | 190 | 41 | 7.294 | 0 |
| 1835 | 100 | 25 | 2 | 190 | 41 | 7.295 | 0 |
| 1834 | 100 | 25 | 2 | 190 | 41 | 7.296 | 0 |
| 1822 | 88 | 29 | 2 | 205 | 31 | 7.296 | 0 |
| 1833 | 100 | 25 | 2 | 190 | 41 | 7.297 | 0 |
| 1832 | 100 | 25 | 2 | 190 | 41 | 7.298 | 0 |
| 3005 | 104 | 26 | 2 | 200 | 42 | 7.3 | 0 |
| 3419 | 100 | 25 | 2 | 190 | 41 | 7.3 | 0 |

| | | | | | | | |
|---|---|---|---|---|---|---|---|
| 3418 | 100 | 25 | 2 | 190 | 41 | 7.301 | 0 |
| 3417 | 100 | 25 | 2 | 190 | 41 | 7.302 | 0 |
| 3416 | 100 | 25 | 2 | 190 | 41 | 7.303 | 0 |
| 1839 | 88 | 29 | 2 | 205 | 31 | 7.3039 | 0 |
| 3415 | 100 | 25 | 2 | 190 | 41 | 7.304 | 0 |
| 3414 | 100 | 25 | 2 | 190 | 41 | 7.305 | 0 |
| 3413 | 100 | 25 | 2 | 190 | 41 | 7.306 | 0 |
| 2721 | 100 | 25 | 2 | 190 | 41 | 7.307 | 0 |
| 1851 | 88 | 29 | 2 | 205 | 31 | 7.30785 | 0 |
| 2681 | 100 | 25 | 2 | 190 | 41 | 7.308 | 0 |
| 2680 | 100 | 25 | 2 | 190 | 41 | 7.309 | 0 |
| 2379 | 100 | 25 | 2 | 190 | 41 | 7.311 | 0 |
| 1861 | 88 | 29 | 2 | 205 | 31 | 7.3118 | 0 |
| 2225 | 100 | 25 | 2 | 190 | 41 | 7.313 | 0 |
| 2224 | 100 | 25 | 2 | 190 | 41 | 7.314 | 0 |
| 2223 | 100 | 25 | 2 | 190 | 41 | 7.315 | 0 |
| 1925 | 88 | 29 | 2 | 205 | 31 | 7.31575 | 0 |
| 2222 | 100 | 25 | 2 | 190 | 41 | 7.316 | 0 |
| 2220 | 100 | 25 | 2 | 190 | 41 | 7.318 | 0 |
| 1939 | 88 | 29 | 2 | 205 | 31 | 7.3197 | 0 |
| 3003 | 104 | 26 | 2 | 200 | 42 | 7.32 | 0 |
| 2214 | 100 | 25 | 2 | 190 | 41 | 7.32 | 0 |
| 2777 | 99 | 25 | 2 | 190 | 50 | 7.321 | 0 |
| 2776 | 99 | 25 | 2 | 190 | 50 | 7.322 | 0 |
| 2775 | 99 | 25 | 2 | 190 | 50 | 7.323 | 0 |
| 2767 | 99 | 25 | 2 | 190 | 50 | 7.324 | 0 |
| 2766 | 99 | 25 | 2 | 190 | 50 | 7.325 | 0 |
| 2708 | 99 | 25 | 2 | 190 | 50 | 7.326 | 0 |
| 2707 | 99 | 25 | 2 | 190 | 50 | 7.327 | 0 |
| 1900 | 99 | 25 | 2 | 190 | 50 | 7.328 | 0 |
| 1842 | 99 | 25 | 2 | 190 | 50 | 7.329 | 0 |
| 3188 | 99 | 25 | 2 | 190 | 49 | 7.33 | 0 |
| 3002 | 104 | 26 | 2 | 200 | 42 | 7.33 | 0 |
| 3211 | 99 | 25 | 2 | 190 | 49 | 7.331 | 0 |
| 3446 | 99 | 25 | 2 | 190 | 49 | 7.332 | 0 |
| 1837 | 99 | 25 | 2 | 190 | 49 | 7.333 | 0 |
| 2031 | 99 | 25 | 2 | 190 | 49 | 7.334 | 0 |
| 2063 | 99 | 25 | 2 | 190 | 49 | 7.335 | 0 |
| 2248 | 99 | 25 | 2 | 190 | 49 | 7.336 | 0 |
| 2291 | 99 | 25 | 2 | 190 | 49 | 7.337 | 0 |
| 2499 | 99 | 25 | 2 | 190 | 49 | 7.338 | 0 |
| 3001 | 104 | 26 | 2 | 200 | 42 | 7.34 | 0 |
| 2968 | 99 | 25 | 2 | 190 | 49 | 7.341 | 0 |
| 2966 | 99 | 25 | 2 | 190 | 49 | 7.343 | 0 |

| | | | | | | | |
|---|---|---|---|---|---|---|---|
| 2965 | 99 | 25 | 2 | 190 | 49 | 7.344 | 0 |
| 2964 | 99 | 25 | 2 | 190 | 49 | 7.345 | 0 |
| 2963 | 99 | 25 | 2 | 190 | 49 | 7.346 | 0 |
| 2962 | 99 | 25 | 2 | 190 | 49 | 7.347 | 0 |
| 2960 | 99 | 25 | 2 | 190 | 49 | 7.349 | 0 |
| 2959 | 99 | 25 | 2 | 190 | 49 | 7.35 | 0 |
| 3000 | 104 | 26 | 2 | 200 | 42 | 7.35 | 0 |
| 2957 | 99 | 25 | 2 | 190 | 49 | 7.352 | 0 |
| 2956 | 99 | 25 | 2 | 190 | 49 | 7.353 | 0 |
| 2955 | 99 | 25 | 2 | 190 | 49 | 7.354 | 0 |
| 2909 | 99 | 25 | 2 | 190 | 49 | 7.355 | 0 |
| 2908 | 99 | 25 | 2 | 190 | 49 | 7.356 | 0 |
| 2907 | 99 | 25 | 2 | 190 | 49 | 7.357 | 0 |
| 2906 | 99 | 25 | 2 | 190 | 49 | 7.358 | 0 |
| 2905 | 99 | 25 | 2 | 190 | 49 | 7.359 | 0 |
| 2904 | 99 | 25 | 2 | 190 | 49 | 7.36 | 0 |
| 2999 | 104 | 26 | 2 | 200 | 42 | 7.36 | 0 |
| 2903 | 99 | 25 | 2 | 190 | 49 | 7.361 | 0 |
| 2902 | 99 | 25 | 2 | 190 | 49 | 7.362 | 0 |
| 2901 | 99 | 25 | 2 | 190 | 49 | 7.363 | 0 |
| 2898 | 99 | 25 | 2 | 190 | 49 | 7.366 | 0 |
| 2897 | 99 | 25 | 2 | 190 | 49 | 7.367 | 0 |
| 2896 | 99 | 25 | 2 | 190 | 49 | 7.368 | 0 |
| 2370 | 99 | 25 | 2 | 190 | 49 | 7.37 | 0 |
| 2998 | 104 | 26 | 2 | 200 | 42 | 7.37 | 0 |
| 2369 | 99 | 25 | 2 | 190 | 49 | 7.371 | 0 |
| 2316 | 99 | 25 | 2 | 190 | 49 | 7.372 | 0 |
| 2146 | 99 | 25 | 2 | 190 | 49 | 7.375 | 0 |
| 2140 | 99 | 25 | 2 | 190 | 49 | 7.377 | 0 |
| 2115 | 99 | 25 | 2 | 190 | 49 | 7.378 | 0 |
| 2114 | 99 | 25 | 2 | 190 | 49 | 7.379 | 0 |
| 2113 | 99 | 25 | 2 | 190 | 49 | 7.38 | 0 |
| 1987 | 99 | 25 | 2 | 190 | 49 | 7.381 | 0 |
| 1986 | 99 | 25 | 2 | 190 | 49 | 7.382 | 0 |
| 2008 | 99 | 25 | 2 | 190 | 43 | 7.383 | 0 |
| 2007 | 99 | 25 | 2 | 190 | 43 | 7.384 | 0 |
| 1980 | 99 | 25 | 2 | 190 | 43 | 7.385 | 0 |
| 1979 | 99 | 25 | 2 | 190 | 43 | 7.386 | 0 |
| 1975 | 99 | 25 | 2 | 190 | 43 | 7.387 | 0 |
| 3172 | 99 | 25 | 2 | 190 | 43 | 7.388 | 0 |
| 3170 | 99 | 25 | 2 | 190 | 43 | 7.39 | 0 |
| 2639 | 99 | 25 | 2 | 190 | 43 | 7.392 | 0 |
| 2988 | 99 | 25 | 2 | 190 | 43 | 7.398 | 0 |
| 2987 | 99 | 25 | 2 | 190 | 43 | 7.399 | 0 |

| | | | | | | | |
|---|---|---|---|---|---|---|---|
| 2986 | 99 | 25 | 2 | 190 | 43 | 7.4 | 0 |
| 2995 | 104 | 26 | 2 | 200 | 42 | 7.4 | 0 |
| 2985 | 99 | 25 | 2 | 190 | 43 | 7.401 | 0 |
| 2983 | 99 | 25 | 2 | 190 | 43 | 7.403 | 0 |
| 2982 | 99 | 25 | 2 | 190 | 43 | 7.404 | 0 |
| 2980 | 99 | 25 | 2 | 190 | 43 | 7.406 | 0 |
| 2978 | 99 | 25 | 2 | 190 | 43 | 7.408 | 0 |
| 3035 | 99 | 25 | 2 | 190 | 43 | 7.418 | 0 |
| 3031 | 99 | 25 | 2 | 190 | 43 | 7.422 | 0 |
| 3025 | 99 | 25 | 2 | 190 | 43 | 7.428 | 0 |
| 3024 | 99 | 25 | 2 | 190 | 43 | 7.429 | 0 |
| 3023 | 99 | 25 | 2 | 190 | 43 | 7.43 | 0 |
| 2992 | 104 | 26 | 2 | 200 | 42 | 7.43 | 0 |
| 2914 | 99 | 25 | 2 | 190 | 43 | 7.433 | 0 |
| 2913 | 99 | 25 | 2 | 190 | 43 | 7.434 | 0 |
| 2912 | 99 | 25 | 2 | 190 | 43 | 7.435 | 0 |
| 2911 | 99 | 25 | 2 | 190 | 43 | 7.436 | 0 |
| 2910 | 99 | 25 | 2 | 190 | 43 | 7.437 | 0 |
| 2549 | 99 | 25 | 2 | 190 | 43 | 7.438 | 0 |
| 2493 | 99 | 25 | 2 | 190 | 43 | 7.439 | 0 |
| 2991 | 104 | 26 | 2 | 200 | 42 | 7.44 | 0 |
| 2491 | 99 | 25 | 2 | 190 | 43 | 7.441 | 0 |
| 2487 | 99 | 25 | 2 | 190 | 43 | 7.445 | 0 |
| 2577 | 98 | 25 | 2 | 190 | 52 | 7.448 | 0 |
| 2729 | 98 | 25 | 2 | 190 | 52 | 7.449 | 0 |
| 2926 | 98 | 25 | 2 | 190 | 52 | 7.45 | 0 |
| 3361 | 103 | 26 | 2 | 200 | 42 | 7.45 | 0 |
| 2700 | 98 | 25 | 2 | 190 | 52 | 7.453 | 0 |
| 2677 | 98 | 25 | 2 | 190 | 52 | 7.455 | 0 |
| 2654 | 98 | 25 | 2 | 190 | 52 | 7.457 | 0 |
| 2385 | 98 | 25 | 2 | 190 | 52 | 7.467 | 0 |
| 3359 | 103 | 26 | 2 | 200 | 42 | 7.47 | 0 |
| 2355 | 98 | 25 | 2 | 190 | 52 | 7.471 | 0 |
| 2354 | 98 | 25 | 2 | 190 | 52 | 7.472 | 0 |
| 2353 | 98 | 25 | 2 | 190 | 52 | 7.473 | 0 |
| 2352 | 98 | 25 | 2 | 190 | 52 | 7.474 | 0 |
| 2339 | 98 | 25 | 2 | 190 | 52 | 7.475 | 0 |
| 2338 | 98 | 25 | 2 | 190 | 52 | 7.476 | 0 |
| 3484 | 97 | 24 | 2 | 190 | 54 | 7.477 | 0 |
| 3507 | 97 | 24 | 2 | 190 | 54 | 7.478 | 0 |
| 3530 | 97 | 24 | 2 | 190 | 54 | 7.479 | 0 |
| 3483 | 97 | 24 | 2 | 190 | 53 | 7.48 | 0 |
| 3358 | 103 | 26 | 2 | 200 | 42 | 7.48 | 0 |
| 3506 | 97 | 24 | 2 | 190 | 53 | 7.481 | 0 |

| | | | | | | | |
|---|---|---|---|---|---|---|---|
| 3529 | 97 | 24 | 2 | 190 | 53 | 7.482 | 0 |
| 3482 | 97 | 24 | 2 | 190 | 52 | 7.483 | 0 |
| 3505 | 97 | 24 | 2 | 190 | 52 | 7.484 | 0 |
| 3528 | 97 | 24 | 2 | 190 | 52 | 7.485 | 0 |
| 3509 | 96 | 24 | 2 | 190 | 56 | 7.487 | 0 |
| 3532 | 96 | 24 | 2 | 190 | 56 | 7.488 | 0 |
| 3485 | 96 | 24 | 2 | 190 | 55 | 7.489 | 0 |
| 3508 | 96 | 24 | 2 | 190 | 55 | 7.49 | 0 |
| 3357 | 103 | 26 | 2 | 200 | 42 | 7.49 | 0 |
| 3531 | 96 | 24 | 2 | 190 | 59 | 7.491 | 0 |
| 3502 | 99 | 25 | 2 | 190 | 59 | 7.492 | 0 |
| 3525 | 99 | 25 | 2 | 190 | 59 | 7.493 | 0 |
| 3065 | 103 | 26 | 2 | 200 | 59 | 7.51 | 1 |
| 2825 | 103 | 26 | 2 | 200 | 60 | 7.55 | 0 |
| 2720 | 103 | 26 | 2 | 200 | 60 | 7.56 | 0 |
| 2719 | 103 | 26 | 2 | 200 | 60 | 7.57 | 0 |
| 2718 | 103 | 26 | 2 | 200 | 60 | 7.58 | 0 |
| 2715 | 103 | 26 | 2 | 200 | 60 | 7.61 | 0 |
| 2714 | 103 | 26 | 2 | 200 | 60 | 7.62 | 0 |
| 2687 | 103 | 26 | 2 | 200 | 60 | 7.63 | 0 |
| 2686 | 103 | 26 | 2 | 200 | 60 | 7.64 | 0 |
| 2685 | 103 | 26 | 2 | 200 | 60 | 7.65 | 1 |
| 2684 | 103 | 26 | 2 | 200 | 60 | 7.66 | 1 |
| 2631 | 103 | 26 | 2 | 200 | 60 | 7.67 | 0 |
| 2592 | 103 | 26 | 2 | 200 | 60 | 7.68 | 1 |
| 2591 | 103 | 26 | 2 | 200 | 60 | 7.69 | 0 |
| 2562 | 103 | 26 | 2 | 200 | 60 | 7.7 | 0 |
| 2561 | 103 | 26 | 2 | 200 | 60 | 7.71 | 0 |
| 2559 | 103 | 26 | 2 | 200 | 60 | 7.73 | 0 |
| 2558 | 103 | 26 | 2 | 200 | 60 | 7.74 | 0 |
| 2556 | 103 | 26 | 2 | 200 | 60 | 7.76 | 0 |
| 2337 | 98 | 25 | 2 | 200 | 60 | 7.77 | 0 |
| 3190 | 98 | 25 | 2 | 200 | 60 | 7.78 | 1 |
| 2554 | 103 | 26 | 2 | 200 | 60 | 7.78 | 0 |
| 3213 | 98 | 25 | 2 | 200 | 60 | 7.79 | 0 |
| 1858 | 98 | 25 | 2 | 200 | 61 | 7.8 | 1 |
| 2552 | 103 | 26 | 2 | 200 | 61 | 7.8 | 0 |
| 2033 | 98 | 25 | 2 | 200 | 61 | 7.81 | 0 |
| 2551 | 103 | 26 | 2 | 200 | 61 | 7.81 | 0 |
| 2065 | 98 | 25 | 2 | 200 | 61 | 7.82 | 0 |
| 2550 | 103 | 26 | 2 | 200 | 61 | 7.82 | 0 |
| 2250 | 98 | 25 | 2 | 200 | 61 | 7.83 | 1 |
| 2005 | 103 | 26 | 2 | 200 | 61 | 7.83 | 0 |
| 2293 | 98 | 25 | 2 | 200 | 61 | 7.84 | 0 |

| | | | | | | | |
|---|---|---|---|---|---|---|---|
| 2002 | 103 | 26 | 2 | 200 | 61 | 7.84 | 0 |
| 2501 | 98 | 25 | 2 | 200 | 61 | 7.85 | 0 |
| 1985 | 103 | 26 | 2 | 200 | 61 | 7.85 | 0 |
| 2576 | 98 | 25 | 2 | 200 | 61 | 7.86 | 0 |
| 1984 | 103 | 26 | 2 | 200 | 61 | 7.86 | 0 |
| 2699 | 98 | 25 | 2 | 200 | 61 | 7.87 | 1 |
| 1983 | 103 | 26 | 2 | 200 | 61 | 7.87 | 0 |
| 2940 | 98 | 25 | 2 | 200 | 61 | 7.88 | 0 |
| 1982 | 103 | 26 | 2 | 200 | 61 | 7.88 | 0 |
| 2939 | 98 | 25 | 2 | 200 | 61 | 7.89 | 0 |
| 2938 | 98 | 25 | 2 | 200 | 61 | 7.9 | 1 |
| 1921 | 103 | 26 | 2 | 200 | 61 | 7.9 | 0 |
| 2937 | 98 | 25 | 2 | 200 | 61 | 7.91 | 1 |
| 1920 | 103 | 26 | 2 | 200 | 61 | 7.91 | 1 |
| 2936 | 98 | 25 | 2 | 200 | 61 | 7.92 | 0 |
| 2935 | 98 | 25 | 2 | 200 | 61 | 7.93 | 1 |
| 2934 | 98 | 25 | 2 | 200 | 61 | 7.94 | 1 |
| 1897 | 103 | 26 | 2 | 200 | 61 | 7.94 | 1 |
| 2933 | 98 | 25 | 2 | 200 | 61 | 7.95 | 1 |
| 1896 | 103 | 26 | 2 | 200 | 61 | 7.95 | 1 |
| 2932 | 98 | 25 | 2 | 200 | 61 | 7.96 | 1 |
| 1894 | 103 | 26 | 2 | 200 | 61 | 7.97 | 0 |
| 3481 | 98 | 25 | 2 | 200 | 61 | 7.99 | 0 |
| 2929 | 98 | 25 | 2 | 200 | 61 | 7.99 | 0 |
| 3504 | 98 | 25 | 2 | 200 | 61 | 8 | 0 |
| 2928 | 98 | 25 | 2 | 200 | 61 | 8 | 0 |
| 3527 | 98 | 25 | 2 | 200 | 61 | 8.01 | 0 |
| 2927 | 98 | 25 | 2 | 200 | 61 | 8.01 | 0 |
| 3473 | 90 | 30 | 2 | 205 | 61 | 8.9497 | 0 |
| 3469 | 90 | 30 | 2 | 205 | 61 | 8.9501 | 0 |
| 3466 | 90 | 30 | 2 | 205 | 61 | 8.9504 | 0 |
| 3465 | 90 | 30 | 2 | 205 | 61 | 8.9505 | 0 |
| 3464 | 90 | 30 | 2 | 205 | 61 | 8.9506 | 0 |
| 3463 | 90 | 30 | 2 | 205 | 61 | 8.9507 | 0 |
| 3461 | 90 | 30 | 2 | 205 | 61 | 8.9509 | 0 |
| 3460 | 90 | 30 | 2 | 205 | 61 | 8.951 | 0 |
| 3459 | 90 | 30 | 2 | 205 | 61 | 8.9511 | 0 |
| 3458 | 90 | 30 | 2 | 205 | 61 | 8.9512 | 0 |
| 3457 | 90 | 30 | 2 | 205 | 61 | 8.9513 | 0 |
| 3455 | 90 | 30 | 2 | 205 | 61 | 8.9515 | 0 |
| 3412 | 103 | 26 | 2 | 210 | 63 | 8.9724 | 0 |
| 3411 | 103 | 26 | 2 | 210 | 63 | 8.9725 | 0 |
| 3407 | 103 | 26 | 2 | 210 | 63 | 8.9729 | 0 |
| 3406 | 103 | 26 | 2 | 210 | 63 | 8.973 | 0 |

| | | | | | | | |
|---|---|---|---|---|---|---|---|
| 3404 | 103 | 26 | 2 | 210 | 63 | 8.9732 | 0 |
| 3397 | 103 | 26 | 2 | 210 | 63 | 8.9739 | 0 |
| 1882 | 102 | 26 | 2 | 210 | 64 | 8.974 | 0 |
| 1841 | 102 | 26 | 2 | 210 | 64 | 8.9741 | 0 |
| 2834 | 102 | 26 | 2 | 210 | 64 | 8.9742 | 0 |
| 2833 | 102 | 26 | 2 | 210 | 64 | 8.9743 | 0 |
| 2832 | 102 | 26 | 2 | 210 | 64 | 8.9744 | 0 |
| 2831 | 102 | 26 | 2 | 210 | 64 | 8.9745 | 0 |
| 2829 | 101 | 26 | 2 | 210 | 64 | 8.9747 | 0 |
| 2828 | 101 | 26 | 2 | 210 | 64 | 8.9748 | 0 |
| 2827 | 101 | 26 | 2 | 210 | 64 | 8.9749 | 0 |
| 2808 | 101 | 26 | 2 | 210 | 64 | 8.9751 | 0 |
| 2805 | 101 | 26 | 2 | 210 | 64 | 8.9754 | 0 |
| 2791 | 101 | 26 | 2 | 210 | 64 | 8.9768 | 0 |
| 2787 | 101 | 26 | 2 | 210 | 64 | 8.9772 | 0 |
| 2771 | 101 | 26 | 2 | 210 | 64 | 8.9775 | 0 |
| 2242 | 82 | 27 | 2 | 210 | 64 | 8.9781 | 0 |
| 2076 | 82 | 27 | 2 | 210 | 64 | 8.9783 | 0 |
| 2075 | 82 | 27 | 2 | 210 | 64 | 8.9784 | 0 |
| 2074 | 82 | 27 | 2 | 210 | 64 | 8.9785 | 0 |
| 2073 | 82 | 27 | 2 | 210 | 64 | 8.9786 | 0 |
| 2072 | 82 | 27 | 2 | 210 | 64 | 8.9787 | 0 |
| 2057 | 82 | 27 | 2 | 210 | 64 | 8.9791 | 0 |
| 2053 | 82 | 27 | 2 | 210 | 64 | 8.9792 | 0 |
| 2052 | 82 | 27 | 2 | 210 | 64 | 8.9793 | 0 |
| 2049 | 81 | 27 | 2 | 210 | 64 | 8.9794 | 0 |
| 2048 | 81 | 27 | 2 | 210 | 64 | 8.9795 | 0 |
| 2047 | 81 | 27 | 2 | 210 | 64 | 8.9796 | 0 |
| 2046 | 81 | 27 | 2 | 210 | 64 | 8.9797 | 0 |
| 2045 | 81 | 27 | 2 | 210 | 64 | 8.9798 | 0 |
| 2018 | 81 | 27 | 2 | 210 | 64 | 8.9799 | 0 |
| 2016 | 81 | 27 | 2 | 210 | 64 | 8.98 | 0 |
| 3423 | 81 | 27 | 2 | 210 | 64 | 8.9803 | 0 |
| 1828 | 80 | 27 | 2 | 210 | 64 | 8.9808 | 0 |
| 2486 | 84 | 28 | 2 | 210 | 64 | 8.9812 | 0 |
| 2485 | 84 | 28 | 2 | 210 | 64 | 8.9813 | 0 |
| 2484 | 84 | 28 | 2 | 210 | 64 | 8.9814 | 0 |
| 2483 | 84 | 28 | 2 | 210 | 64 | 8.9815 | 0 |
| 2482 | 84 | 28 | 2 | 210 | 64 | 8.9816 | 0 |
| 2481 | 84 | 28 | 2 | 210 | 64 | 8.9817 | 0 |
| 2375 | 84 | 28 | 2 | 210 | 64 | 8.982 | 0 |
| 2372 | 84 | 28 | 2 | 210 | 64 | 8.9823 | 0 |
| 2003 | 84 | 28 | 2 | 210 | 64 | 8.9825 | 0 |
| 1946 | 89 | 29 | 2 | 210 | 65 | 8.9838 | 0 |

| | | | | | | | |
|---|---|---|---|---|---|---|---|
| 1944 | 89 | 29 | 2 | 210 | 65 | 8.984 | 0 |
| 1937 | 89 | 29 | 2 | 210 | 65 | 8.9844 | 0 |
| 1934 | 89 | 29 | 2 | 210 | 65 | 8.9847 | 0 |
| 1933 | 89 | 29 | 2 | 210 | 65 | 8.9848 | 0 |
| 1932 | 89 | 29 | 2 | 210 | 65 | 8.9849 | 0 |
| 1931 | 89 | 29 | 2 | 210 | 65 | 8.985 | 0 |
| 1930 | 89 | 29 | 2 | 210 | 65 | 8.9851 | 0 |
| 1929 | 89 | 29 | 2 | 210 | 65 | 8.9852 | 0 |
| 1928 | 89 | 29 | 2 | 210 | 65 | 8.9853 | 0 |
| 1917 | 89 | 29 | 2 | 210 | 65 | 8.9854 | 0 |
| 1916 | 89 | 29 | 2 | 210 | 65 | 8.9855 | 0 |
| 1915 | 89 | 29 | 2 | 210 | 65 | 8.9856 | 0 |
| 1891 | 89 | 29 | 2 | 210 | 65 | 8.9857 | 0 |
| 1879 | 89 | 29 | 2 | 210 | 65 | 8.9858 | 0 |
| 1863 | 89 | 29 | 2 | 210 | 65 | 8.9859 | 0 |
| 1854 | 89 | 29 | 2 | 210 | 65 | 8.986 | 0 |
| 1853 | 89 | 29 | 2 | 210 | 65 | 8.9861 | 0 |
| 1852 | 89 | 29 | 2 | 210 | 65 | 8.9862 | 0 |
| 1847 | 89 | 29 | 2 | 210 | 65 | 8.9863 | 0 |
| 1846 | 89 | 29 | 2 | 210 | 65 | 8.9864 | 0 |
| 1843 | 89 | 29 | 2 | 210 | 65 | 8.9865 | 0 |
| 1819 | 89 | 29 | 2 | 210 | 65 | 8.9866 | 0 |
| 1817 | 89 | 29 | 2 | 210 | 65 | 8.9867 | 0 |
| 1815 | 89 | 29 | 2 | 210 | 65 | 8.9869 | 0 |
| 1814 | 89 | 29 | 2 | 210 | 65 | 8.987 | 0 |
| 3480 | 81 | 27 | 2 | 210 | 65 | 8.9871 | 0 |
| 3479 | 81 | 27 | 2 | 210 | 65 | 8.9872 | 0 |
| 3478 | 81 | 27 | 2 | 210 | 65 | 8.9873 | 0 |
| 3477 | 81 | 27 | 2 | 210 | 65 | 8.9874 | 0 |
| 3476 | 81 | 27 | 2 | 210 | 65 | 8.9875 | 0 |
| 3475 | 81 | 27 | 2 | 210 | 65 | 8.9876 | 0 |
| 3474 | 81 | 27 | 2 | 210 | 65 | 8.9877 | 0 |
| 1506 | 99 | 25 | 1 | 190 | 49 | 7.25 | 0 |
| 1465 | 99 | 25 | 1 | 190 | 42 | 7.29 | 0 |
| 1647 | 103 | 26 | 1 | 200 | 42 | 7.3276 | 0 |
| 1459 | 99 | 25 | 1 | 190 | 42 | 7.35 | 0 |
| 1662 | 103 | 26 | 1 | 200 | 42 | 7.3513 | 0 |
| 1663 | 103 | 26 | 1 | 200 | 42 | 7.35525 | 0 |
| 1674 | 103 | 26 | 1 | 200 | 42 | 7.375 | 0 |
| 1740 | 99 | 25 | 1 | 190 | 43 | 7.39 | 0 |
| 1680 | 103 | 26 | 1 | 200 | 42 | 7.39475 | 0 |
| 1684 | 103 | 26 | 1 | 200 | 42 | 7.4066 | 0 |
| 1441 | 99 | 25 | 1 | 190 | 42 | 7.41 | 0 |
| 1688 | 103 | 26 | 1 | 200 | 42 | 7.41055 | 0 |

| | | | | | | | |
|---|---|---|---|---|---|---|---|
| 1689 | 103 | 26 | 1 | 200 | 42 | 7.4145 | 0 |
| 1690 | 103 | 26 | 1 | 200 | 42 | 7.41845 | 0 |
| 1692 | 103 | 26 | 1 | 200 | 42 | 7.4224 | 0 |
| 1718 | 103 | 26 | 1 | 200 | 42 | 7.4303 | 0 |
| 1637 | 99 | 25 | 1 | 190 | 43 | 7.44 | 0 |
| 1721 | 103 | 26 | 1 | 200 | 42 | 7.44215 | 0 |
| 1722 | 103 | 26 | 1 | 200 | 42 | 7.4461 | 0 |
| 1738 | 103 | 26 | 1 | 200 | 42 | 7.454 | 0 |
| 1014 | 104 | 26 | 1 | 200 | 51 | 7.45795 | 0 |
| 1622 | 99 | 25 | 1 | 190 | 43 | 7.46 | 0 |
| 1019 | 104 | 26 | 1 | 200 | 51 | 7.4619 | 0 |
| 1036 | 104 | 26 | 1 | 200 | 51 | 7.4698 | 0 |
| 1607 | 99 | 25 | 1 | 190 | 43 | 7.47 | 0 |
| 1160 | 104 | 26 | 1 | 200 | 59 | 7.4935 | 0 |
| 1791 | 97 | 24 | 1 | 190 | 59 | 7.495 | 0 |
| 1142 | 97 | 24 | 1 | 190 | 59 | 7.502 | 0 |
| 1140 | 97 | 24 | 1 | 190 | 59 | 7.503 | 0 |
| 1284 | 97 | 24 | 1 | 190 | 59 | 7.513 | 0 |
| 1184 | 97 | 24 | 1 | 190 | 59 | 7.515 | 1 |
| 1180 | 97 | 24 | 1 | 190 | 60 | 7.516 | 0 |
| 1171 | 97 | 24 | 1 | 190 | 60 | 7.517 | 0 |
| 1169 | 97 | 24 | 1 | 190 | 60 | 7.519 | 0 |
| 1587 | 99 | 25 | 1 | 190 | 60 | 7.52 | 0 |
| 1165 | 97 | 24 | 1 | 190 | 60 | 7.52 | 0 |
| 1164 | 97 | 24 | 1 | 190 | 60 | 7.521 | 0 |
| 1163 | 97 | 24 | 1 | 190 | 60 | 7.522 | 0 |
| 1162 | 97 | 24 | 1 | 190 | 60 | 7.523 | 0 |
| 1161 | 97 | 24 | 1 | 190 | 60 | 7.524 | 0 |
| 1156 | 97 | 24 | 1 | 190 | 60 | 7.525 | 0 |
| 1154 | 97 | 24 | 1 | 190 | 60 | 7.526 | 0 |
| 1147 | 97 | 24 | 1 | 190 | 60 | 7.527 | 0 |
| 1487 | 97 | 24 | 1 | 190 | 60 | 7.528 | 0 |
| 1486 | 97 | 24 | 1 | 190 | 60 | 7.529 | 1 |
| 1584 | 99 | 25 | 1 | 190 | 60 | 7.53 | 1 |
| 1483 | 97 | 24 | 1 | 190 | 60 | 7.53 | 0 |
| 1482 | 97 | 24 | 1 | 190 | 60 | 7.531 | 0 |
| 1481 | 97 | 24 | 1 | 190 | 60 | 7.532 | 0 |
| 1480 | 97 | 24 | 1 | 190 | 60 | 7.533 | 0 |
| 1479 | 97 | 24 | 1 | 190 | 60 | 7.534 | 0 |
| 1380 | 97 | 24 | 1 | 190 | 60 | 7.538 | 0 |
| 1259 | 97 | 24 | 1 | 190 | 60 | 7.543 | 0 |
| 1258 | 97 | 24 | 1 | 190 | 60 | 7.544 | 0 |
| 1257 | 97 | 24 | 1 | 190 | 60 | 7.545 | 0 |
| 1237 | 97 | 24 | 1 | 190 | 60 | 7.546 | 0 |

| | | | | | | | |
|---|---|---|---|---|---|---|---|
| 1236 | 97 | 24 | 1 | 190 | 60 | 7.547 | 0 |
| 1547 | 99 | 25 | 1 | 190 | 60 | 7.6 | 0 |
| 1531 | 99 | 25 | 1 | 190 | 60 | 7.61 | 0 |
| 1320 | 98 | 25 | 1 | 190 | 60 | 7.68 | 0 |
| 1319 | 98 | 25 | 1 | 190 | 60 | 7.69 | 0 |
| 1274 | 98 | 25 | 1 | 190 | 60 | 7.73 | 0 |
| 1260 | 98 | 25 | 1 | 190 | 60 | 7.76 | 0 |
| 1772 | 89 | 29 | 1 | 205 | 61 | 8.9518 | 0 |
| 1756 | 89 | 29 | 1 | 205 | 61 | 8.9519 | 0 |
| 1713 | 89 | 29 | 1 | 205 | 61 | 8.9524 | 0 |
| 1709 | 89 | 29 | 1 | 205 | 61 | 8.9525 | 0 |
| 1705 | 89 | 29 | 1 | 205 | 61 | 8.9526 | 0 |
| 1704 | 89 | 29 | 1 | 205 | 61 | 8.9527 | 0 |
| 1642 | 89 | 29 | 1 | 205 | 61 | 8.953 | 0 |
| 1523 | 89 | 29 | 1 | 205 | 61 | 8.9539 | 0 |
| 1452 | 89 | 29 | 1 | 205 | 61 | 8.9543 | 0 |
| 1450 | 89 | 29 | 1 | 205 | 61 | 8.9544 | 0 |
| 1446 | 89 | 29 | 1 | 205 | 61 | 8.9545 | 0 |
| 1436 | 89 | 29 | 1 | 205 | 61 | 8.9547 | 0 |
| 1409 | 89 | 29 | 1 | 205 | 62 | 8.9551 | 0 |
| 1403 | 89 | 29 | 1 | 205 | 62 | 8.9552 | 0 |
| 1425 | 88 | 29 | 1 | 205 | 62 | 8.9566 | 0 |
| 1811 | 87 | 29 | 1 | 205 | 62 | 8.9582 | 0 |
| 1807 | 87 | 29 | 1 | 205 | 62 | 8.9583 | 0 |
| 1806 | 87 | 29 | 1 | 205 | 62 | 8.9584 | 0 |
| 1707 | 87 | 29 | 1 | 205 | 62 | 8.9585 | 0 |
| 1144 | 90 | 30 | 1 | 205 | 62 | 8.9595 | 0 |
| 1125 | 90 | 30 | 1 | 205 | 62 | 8.9596 | 0 |
| 1362 | 89 | 30 | 1 | 205 | 62 | 8.9612 | 0 |
| 1358 | 89 | 30 | 1 | 205 | 62 | 8.9613 | 0 |
| 1334 | 89 | 30 | 1 | 205 | 62 | 8.9616 | 0 |
| 1302 | 89 | 30 | 1 | 205 | 62 | 8.9622 | 0 |
| 1291 | 89 | 30 | 1 | 205 | 62 | 8.9624 | 0 |
| 1015 | 89 | 30 | 1 | 205 | 62 | 8.9637 | 0 |
| 1810 | 89 | 30 | 1 | 205 | 63 | 8.9653 | 0 |
| 1617 | 89 | 30 | 1 | 205 | 63 | 8.9657 | 0 |
| 1356 | 89 | 30 | 1 | 205 | 63 | 8.9659 | 0 |
| 1288 | 89 | 30 | 1 | 205 | 63 | 8.9667 | 0 |
| 1286 | 89 | 30 | 1 | 205 | 63 | 8.9669 | 0 |
| 1245 | 89 | 30 | 1 | 205 | 63 | 8.9672 | 0 |
| 1630 | 86 | 30 | 1 | 205 | 63 | 8.9673 | 0 |
| 1149 | 91 | 31 | 1 | 205 | 63 | 8.9678 | 0 |
| 1343 | 90 | 31 | 1 | 205 | 63 | 8.9691 | 0 |
| 1342 | 90 | 31 | 1 | 205 | 63 | 8.9692 | 0 |

| | | | | | | | |
|---|---|---|---|---|---|---|---|
| 1327 | 90 | 31 | 1 | 205 | 63 | 8.9697 | 0 |
| 1298 | 90 | 31 | 1 | 205 | 63 | 8.9704 | 0 |
| 1297 | 90 | 31 | 1 | 205 | 63 | 8.9705 | 0 |
| 1293 | 90 | 31 | 1 | 205 | 63 | 8.9707 | 0 |
| 1290 | 90 | 31 | 1 | 205 | 63 | 8.9708 | 0 |
| 1255 | 90 | 31 | 1 | 205 | 63 | 8.9713 | 0 |
| 1641 | 103 | 26 | 1 | 210 | 65 | 8.9879 | 0 |
| 1639 | 103 | 26 | 1 | 210 | 65 | 8.9881 | 0 |
| 1770 | 102 | 26 | 1 | 210 | 65 | 8.9887 | 0 |
| 1708 | 102 | 26 | 1 | 210 | 65 | 8.9892 | 0 |
| 1685 | 102 | 26 | 1 | 210 | 65 | 8.9893 | 0 |
| 1371 | 102 | 26 | 1 | 210 | 65 | 8.9907 | 0 |
| 1365 | 102 | 26 | 1 | 210 | 65 | 8.9909 | 0 |
| 1329 | 102 | 26 | 1 | 210 | 65 | 8.991 | 0 |
| 1295 | 102 | 26 | 1 | 210 | 65 | 8.9911 | 0 |
| 1282 | 102 | 26 | 1 | 210 | 66 | 8.9913 | 0 |
| 1263 | 102 | 26 | 1 | 210 | 66 | 8.9914 | 0 |
| 1262 | 102 | 26 | 1 | 210 | 66 | 8.9915 | 0 |
| 1229 | 102 | 26 | 1 | 210 | 66 | 8.9916 | 0 |
| 1226 | 102 | 26 | 1 | 210 | 66 | 8.9917 | 0 |
| 1199 | 102 | 26 | 1 | 210 | 66 | 8.9918 | 0 |
| 1196 | 102 | 26 | 1 | 210 | 66 | 8.9919 | 0 |
| 1190 | 102 | 26 | 1 | 210 | 66 | 8.992 | 0 |
| 1168 | 102 | 26 | 1 | 210 | 66 | 8.9921 | 0 |
| 1159 | 102 | 26 | 1 | 210 | 66 | 8.9922 | 0 |
| 1157 | 102 | 26 | 1 | 210 | 66 | 8.9923 | 0 |
| 1100 | 102 | 26 | 1 | 210 | 66 | 8.9924 | 0 |
| 1098 | 102 | 26 | 1 | 210 | 66 | 8.9925 | 0 |
| 1087 | 102 | 26 | 1 | 210 | 66 | 8.9926 | 0 |
| 1082 | 102 | 26 | 1 | 210 | 66 | 8.9927 | 0 |
| 1046 | 102 | 26 | 1 | 210 | 67 | 8.9928 | 0 |
| 1011 | 102 | 26 | 1 | 210 | 67 | 8.9929 | 0 |
| 1798 | 80 | 27 | 1 | 210 | 67 | 8.993 | 0 |
| 1795 | 80 | 27 | 1 | 210 | 67 | 8.9931 | 0 |
| 1794 | 80 | 27 | 1 | 210 | 67 | 8.9932 | 0 |
| 1788 | 80 | 27 | 1 | 210 | 67 | 8.9936 | 0 |
| 1778 | 80 | 27 | 1 | 210 | 67 | 8.994 | 0 |
| 1775 | 80 | 27 | 1 | 210 | 67 | 8.9943 | 0 |
| 1774 | 80 | 27 | 1 | 210 | 68 | 8.9944 | 0 |
| 1768 | 80 | 27 | 1 | 210 | 68 | 8.9945 | 0 |
| 1763 | 80 | 27 | 1 | 210 | 68 | 8.9946 | 0 |
| 1759 | 80 | 27 | 1 | 210 | 68 | 8.9947 | 0 |
| 1438 | 86 | 28 | 1 | 210 | 69 | 8.9965 | 0 |
| 1415 | 86 | 28 | 1 | 210 | 69 | 8.9966 | 0 |

| | | | | | | | |
|---|---|---|---|---|---|---|---|
| 1373 | 86 | 28 | 1 | 210 | 69 | 8.9967 | 0 |
| 1364 | 86 | 28 | 1 | 210 | 69 | 8.9968 | 0 |
| 1153 | 83 | 28 | 1 | 210 | 70 | 8.9983 | 0 |
| 1148 | 83 | 28 | 1 | 210 | 70 | 8.9984 | 0 |
| 1214 | 89 | 29 | 1 | 210 | 70 | 8.9991 | 0 |
| 1212 | 89 | 29 | 1 | 210 | 71 | 8.9993 | 0 |
| 1175 | 89 | 29 | 1 | 210 | 71 | 8.9996 | 0 |
| 1008 | 89 | 29 | 1 | 210 | 71 | 8.9998 | 0 |
| 1696 | 89 | 29 | 1 | 210 | 71 | 9.0001 | 0 |
| 1360 | 89 | 29 | 1 | 210 | 71 | 9.0005 | 0 |
| 2852 | 91 | 20 | 2 | 140 | 50 | 6.232 | 1 |
| 1953 | 92 | 20 | 2 | 140 | 50 | 6.29 | 1 |
| 1905 | 92 | 20 | 2 | 140 | 49 | 6.298 | 1 |
| 2779 | 95 | 23 | 2 | 185 | 58 | 6.935 | 1 |
| 3158 | 90 | 30 | 2 | 205 | 53 | 7.2091 | 1 |
| 3195 | 96 | 24 | 2 | 190 | 56 | 7.212 | 1 |
| 2255 | 96 | 24 | 2 | 190 | 56 | 7.217 | 1 |
| 2463 | 96 | 24 | 2 | 190 | 56 | 7.218 | 1 |
| 2302 | 96 | 24 | 2 | 190 | 56 | 7.222 | 1 |
| 2301 | 96 | 24 | 2 | 190 | 56 | 7.223 | 1 |
| 3162 | 90 | 30 | 2 | 205 | 53 | 7.2249 | 1 |
| 2299 | 96 | 24 | 2 | 190 | 56 | 7.225 | 1 |
| 3012 | 104 | 26 | 2 | 200 | 42 | 7.23 | 0 |
| 2210 | 96 | 24 | 2 | 190 | 56 | 7.231 | 0 |
| 2209 | 96 | 24 | 2 | 190 | 56 | 7.232 | 0 |
| 3164 | 90 | 30 | 2 | 205 | 53 | 7.2328 | 0 |
| 2206 | 96 | 24 | 2 | 190 | 56 | 7.234 | 0 |
| 2205 | 96 | 24 | 2 | 190 | 56 | 7.235 | 0 |
| 3352 | 96 | 24 | 2 | 190 | 56 | 7.239 | 0 |
| 3349 | 96 | 24 | 2 | 190 | 56 | 7.242 | 0 |
| 3347 | 96 | 24 | 2 | 190 | 56 | 7.244 | 0 |
| 3346 | 96 | 24 | 2 | 190 | 56 | 7.245 | 0 |
| 3345 | 96 | 24 | 2 | 190 | 56 | 7.246 | 0 |
| 3341 | 96 | 24 | 2 | 190 | 56 | 7.25 | 0 |
| 3010 | 104 | 26 | 2 | 200 | 42 | 7.25 | 0 |
| 3340 | 96 | 24 | 2 | 190 | 56 | 7.251 | 0 |
| 3338 | 96 | 24 | 2 | 190 | 56 | 7.253 | 0 |
| 2667 | 101 | 25 | 2 | 190 | 41 | 7.26 | 0 |
| 2665 | 101 | 25 | 2 | 190 | 41 | 7.262 | 0 |
| 2664 | 101 | 25 | 2 | 190 | 41 | 7.263 | 0 |
| 2659 | 101 | 25 | 2 | 190 | 41 | 7.264 | 0 |
| 2658 | 101 | 25 | 2 | 190 | 41 | 7.265 | 0 |
| 3451 | 90 | 30 | 2 | 205 | 52 | 7.26835 | 0 |
| 2596 | 101 | 25 | 2 | 190 | 41 | 7.269 | 0 |

| | | | | | | | |
|---|---|---|---|---|---|---|---|
| 2326 | 100 | 25 | 2 | 190 | 41 | 7.279 | 0 |
| 3006 | 104 | 26 | 2 | 200 | 42 | 7.29 | 0 |
| 1864 | 100 | 25 | 2 | 190 | 41 | 7.29 | 0 |
| 1862 | 100 | 25 | 2 | 190 | 41 | 7.291 | 0 |
| 1831 | 100 | 25 | 2 | 190 | 41 | 7.299 | 0 |
| 1823 | 88 | 29 | 2 | 205 | 31 | 7.29995 | 0 |
| 3004 | 104 | 26 | 2 | 200 | 42 | 7.31 | 0 |
| 2380 | 100 | 25 | 2 | 190 | 41 | 7.31 | 0 |
| 2378 | 100 | 25 | 2 | 190 | 41 | 7.312 | 0 |
| 2221 | 100 | 25 | 2 | 190 | 41 | 7.317 | 0 |
| 2215 | 100 | 25 | 2 | 190 | 41 | 7.319 | 0 |
| 2574 | 99 | 25 | 2 | 190 | 49 | 7.339 | 0 |
| 2697 | 99 | 25 | 2 | 190 | 49 | 7.34 | 0 |
| 2967 | 99 | 25 | 2 | 190 | 49 | 7.342 | 0 |
| 2961 | 99 | 25 | 2 | 190 | 49 | 7.348 | 0 |
| 2958 | 99 | 25 | 2 | 190 | 49 | 7.351 | 0 |
| 2900 | 99 | 25 | 2 | 190 | 49 | 7.364 | 0 |
| 2899 | 99 | 25 | 2 | 190 | 49 | 7.365 | 0 |
| 2371 | 99 | 25 | 2 | 190 | 49 | 7.369 | 0 |
| 2202 | 99 | 25 | 2 | 190 | 49 | 7.373 | 0 |
| 2170 | 99 | 25 | 2 | 190 | 49 | 7.374 | 0 |
| 2141 | 99 | 25 | 2 | 190 | 49 | 7.376 | 0 |
| 2997 | 104 | 26 | 2 | 200 | 42 | 7.38 | 0 |
| 3171 | 99 | 25 | 2 | 190 | 43 | 7.389 | 0 |
| 2996 | 104 | 26 | 2 | 200 | 42 | 7.39 | 0 |
| 3169 | 99 | 25 | 2 | 190 | 43 | 7.391 | 0 |
| 2638 | 99 | 25 | 2 | 190 | 43 | 7.393 | 0 |
| 2286 | 99 | 25 | 2 | 190 | 43 | 7.394 | 0 |
| 2285 | 99 | 25 | 2 | 190 | 43 | 7.395 | 0 |
| 2241 | 99 | 25 | 2 | 190 | 43 | 7.396 | 0 |
| 2240 | 99 | 25 | 2 | 190 | 43 | 7.397 | 0 |
| 2984 | 99 | 25 | 2 | 190 | 43 | 7.402 | 0 |
| 2981 | 99 | 25 | 2 | 190 | 43 | 7.405 | 0 |
| 2979 | 99 | 25 | 2 | 190 | 43 | 7.407 | 0 |
| 2977 | 99 | 25 | 2 | 190 | 43 | 7.409 | 0 |
| 2976 | 99 | 25 | 2 | 190 | 43 | 7.41 | 0 |
| 2994 | 104 | 26 | 2 | 200 | 42 | 7.41 | 0 |
| 2975 | 99 | 25 | 2 | 190 | 43 | 7.411 | 0 |
| 2974 | 99 | 25 | 2 | 190 | 43 | 7.412 | 0 |
| 2973 | 99 | 25 | 2 | 190 | 43 | 7.413 | 0 |
| 2972 | 99 | 25 | 2 | 190 | 43 | 7.414 | 0 |
| 2971 | 99 | 25 | 2 | 190 | 43 | 7.415 | 0 |
| 2970 | 99 | 25 | 2 | 190 | 43 | 7.416 | 0 |
| 2969 | 99 | 25 | 2 | 190 | 43 | 7.417 | 0 |

| | | | | | | | |
|---|---|---|---|---|---|---|---|
| 3034 | 99 | 25 | 2 | 190 | 43 | 7.419 | 0 |
| 3033 | 99 | 25 | 2 | 190 | 43 | 7.42 | 0 |
| 2993 | 104 | 26 | 2 | 200 | 42 | 7.42 | 0 |
| 3032 | 99 | 25 | 2 | 190 | 43 | 7.421 | 0 |
| 3030 | 99 | 25 | 2 | 190 | 43 | 7.423 | 0 |
| 3029 | 99 | 25 | 2 | 190 | 43 | 7.424 | 0 |
| 3028 | 99 | 25 | 2 | 190 | 43 | 7.425 | 0 |
| 3027 | 99 | 25 | 2 | 190 | 43 | 7.426 | 0 |
| 3026 | 99 | 25 | 2 | 190 | 43 | 7.427 | 0 |
| 2916 | 99 | 25 | 2 | 190 | 43 | 7.431 | 0 |
| 2915 | 99 | 25 | 2 | 190 | 43 | 7.432 | 0 |
| 2492 | 99 | 25 | 2 | 190 | 43 | 7.44 | 0 |
| 2490 | 99 | 25 | 2 | 190 | 43 | 7.442 | 0 |
| 2489 | 99 | 25 | 2 | 190 | 43 | 7.443 | 0 |
| 2488 | 99 | 25 | 2 | 190 | 43 | 7.444 | 0 |
| 2295 | 98 | 25 | 2 | 190 | 52 | 7.446 | 0 |
| 2502 | 98 | 25 | 2 | 190 | 52 | 7.447 | 0 |
| 2888 | 98 | 25 | 2 | 190 | 52 | 7.451 | 0 |
| 2887 | 98 | 25 | 2 | 190 | 52 | 7.452 | 0 |
| 2678 | 98 | 25 | 2 | 190 | 52 | 7.454 | 0 |
| 2655 | 98 | 25 | 2 | 190 | 52 | 7.456 | 0 |
| 2653 | 98 | 25 | 2 | 190 | 52 | 7.458 | 0 |
| 2652 | 98 | 25 | 2 | 190 | 52 | 7.459 | 0 |
| 2651 | 98 | 25 | 2 | 190 | 52 | 7.46 | 0 |
| 3360 | 103 | 26 | 2 | 200 | 42 | 7.46 | 0 |
| 2650 | 98 | 25 | 2 | 190 | 52 | 7.461 | 0 |
| 2649 | 98 | 25 | 2 | 190 | 52 | 7.462 | 0 |
| 2648 | 98 | 25 | 2 | 190 | 52 | 7.463 | 0 |
| 2388 | 98 | 25 | 2 | 190 | 52 | 7.464 | 0 |
| 2387 | 98 | 25 | 2 | 190 | 52 | 7.465 | 0 |
| 2386 | 98 | 25 | 2 | 190 | 52 | 7.466 | 0 |
| 2384 | 98 | 25 | 2 | 190 | 52 | 7.468 | 0 |
| 2377 | 98 | 25 | 2 | 190 | 52 | 7.469 | 0 |
| 2356 | 98 | 25 | 2 | 190 | 52 | 7.47 | 0 |
| 3486 | 96 | 24 | 2 | 190 | 56 | 7.486 | 0 |
| 3066 | 103 | 26 | 2 | 200 | 59 | 7.5 | 0 |
| 2919 | 103 | 26 | 2 | 200 | 60 | 7.52 | 0 |
| 2918 | 103 | 26 | 2 | 200 | 60 | 7.53 | 1 |
| 2917 | 103 | 26 | 2 | 200 | 60 | 7.54 | 1 |
| 2717 | 103 | 26 | 2 | 200 | 60 | 7.59 | 0 |
| 2716 | 103 | 26 | 2 | 200 | 60 | 7.6 | 0 |
| 2560 | 103 | 26 | 2 | 200 | 60 | 7.72 | 0 |
| 2557 | 103 | 26 | 2 | 200 | 60 | 7.75 | 0 |
| 2555 | 103 | 26 | 2 | 200 | 60 | 7.77 | 0 |

| | | | | | | | |
|---|---|---|---|---|---|---|---|
| 2553 | 103 | 26 | 2 | 200 | 60 | 7.79 | 0 |
| 1978 | 103 | 26 | 2 | 200 | 61 | 7.89 | 0 |
| 1907 | 103 | 26 | 2 | 200 | 61 | 7.92 | 0 |
| 1902 | 103 | 26 | 2 | 200 | 61 | 7.93 | 0 |
| 1895 | 103 | 26 | 2 | 200 | 61 | 7.96 | 0 |
| 2931 | 98 | 25 | 2 | 200 | 61 | 7.97 | 1 |
| 2930 | 98 | 25 | 2 | 200 | 61 | 7.98 | 0 |
| 1887 | 103 | 26 | 2 | 200 | 61 | 7.98 | 0 |
| 2061 | 91 | 31 | 2 | 205 | 61 | 8.9496 | 0 |
| 3472 | 90 | 30 | 2 | 205 | 61 | 8.9498 | 0 |
| 3471 | 90 | 30 | 2 | 205 | 61 | 8.9499 | 0 |
| 3470 | 90 | 30 | 2 | 205 | 61 | 8.95 | 0 |
| 3468 | 90 | 30 | 2 | 205 | 61 | 8.9502 | 0 |
| 3467 | 90 | 30 | 2 | 205 | 61 | 8.9503 | 0 |
| 3462 | 90 | 30 | 2 | 205 | 61 | 8.9508 | 0 |
| 3456 | 90 | 30 | 2 | 205 | 61 | 8.9514 | 0 |
| 3523 | 91 | 31 | 2 | 205 | 61 | 8.9516 | 0 |
| 3410 | 103 | 26 | 2 | 210 | 63 | 8.9726 | 0 |
| 3409 | 103 | 26 | 2 | 210 | 63 | 8.9727 | 0 |
| 3408 | 103 | 26 | 2 | 210 | 63 | 8.9728 | 0 |
| 3405 | 103 | 26 | 2 | 210 | 63 | 8.9731 | 0 |
| 3403 | 103 | 26 | 2 | 210 | 63 | 8.9733 | 0 |
| 3402 | 103 | 26 | 2 | 210 | 63 | 8.9734 | 0 |
| 3401 | 103 | 26 | 2 | 210 | 63 | 8.9735 | 0 |
| 3400 | 103 | 26 | 2 | 210 | 63 | 8.9736 | 0 |
| 3399 | 103 | 26 | 2 | 210 | 63 | 8.9737 | 0 |
| 3398 | 103 | 26 | 2 | 210 | 63 | 8.9738 | 0 |
| 2830 | 101 | 26 | 2 | 210 | 64 | 8.9746 | 0 |
| 2826 | 101 | 26 | 2 | 210 | 64 | 8.975 | 0 |
| 2807 | 101 | 26 | 2 | 210 | 64 | 8.9752 | 0 |
| 2806 | 101 | 26 | 2 | 210 | 64 | 8.9753 | 0 |
| 2804 | 101 | 26 | 2 | 210 | 64 | 8.9755 | 0 |
| 2803 | 101 | 26 | 2 | 210 | 64 | 8.9756 | 0 |
| 2802 | 101 | 26 | 2 | 210 | 64 | 8.9757 | 0 |
| 2801 | 101 | 26 | 2 | 210 | 64 | 8.9758 | 0 |
| 2800 | 101 | 26 | 2 | 210 | 64 | 8.9759 | 0 |
| 2799 | 101 | 26 | 2 | 210 | 64 | 8.976 | 0 |
| 2798 | 101 | 26 | 2 | 210 | 64 | 8.9761 | 0 |
| 2797 | 101 | 26 | 2 | 210 | 64 | 8.9762 | 0 |
| 2796 | 101 | 26 | 2 | 210 | 64 | 8.9763 | 0 |
| 2795 | 101 | 26 | 2 | 210 | 64 | 8.9764 | 0 |
| 2794 | 101 | 26 | 2 | 210 | 64 | 8.9765 | 0 |
| 2793 | 101 | 26 | 2 | 210 | 64 | 8.9766 | 0 |
| 2792 | 101 | 26 | 2 | 210 | 64 | 8.9767 | 0 |

| | | | | | | | |
|---|---|---|---|---|---|---|---|
| 2790 | 101 | 26 | 2 | 210 | 64 | 8.9769 | 0 |
| 2789 | 101 | 26 | 2 | 210 | 64 | 8.977 | 0 |
| 2788 | 101 | 26 | 2 | 210 | 64 | 8.9771 | 0 |
| 2786 | 101 | 26 | 2 | 210 | 64 | 8.9773 | 0 |
| 2772 | 101 | 26 | 2 | 210 | 64 | 8.9774 | 0 |
| 2770 | 101 | 26 | 2 | 210 | 64 | 8.9776 | 0 |
| 2769 | 101 | 26 | 2 | 210 | 64 | 8.9777 | 0 |
| 2671 | 101 | 26 | 2 | 210 | 64 | 8.9778 | 0 |
| 2670 | 101 | 26 | 2 | 210 | 64 | 8.9779 | 0 |
| 2669 | 101 | 26 | 2 | 210 | 64 | 8.978 | 0 |
| 2077 | 82 | 27 | 2 | 210 | 64 | 8.9782 | 0 |
| 2071 | 82 | 27 | 2 | 210 | 64 | 8.9788 | 0 |
| 2070 | 82 | 27 | 2 | 210 | 64 | 8.9789 | 0 |
| 2069 | 82 | 27 | 2 | 210 | 64 | 8.979 | 0 |
| 3425 | 81 | 27 | 2 | 210 | 64 | 8.9801 | 0 |
| 3424 | 81 | 27 | 2 | 210 | 64 | 8.9802 | 0 |
| 3422 | 81 | 27 | 2 | 210 | 64 | 8.9804 | 0 |
| 3421 | 81 | 27 | 2 | 210 | 64 | 8.9805 | 0 |
| 3420 | 81 | 27 | 2 | 210 | 64 | 8.9806 | 0 |
| 3173 | 81 | 27 | 2 | 210 | 64 | 8.9807 | 0 |
| 1827 | 80 | 27 | 2 | 210 | 64 | 8.9809 | 0 |
| 1824 | 80 | 27 | 2 | 210 | 64 | 8.981 | 0 |
| 1922 | 86 | 28 | 2 | 210 | 64 | 8.9811 | 0 |
| 2480 | 84 | 28 | 2 | 210 | 64 | 8.9818 | 0 |
| 2393 | 84 | 28 | 2 | 210 | 64 | 8.9819 | 0 |
| 2374 | 84 | 28 | 2 | 210 | 64 | 8.9821 | 0 |
| 2373 | 84 | 28 | 2 | 210 | 64 | 8.9822 | 0 |
| 2004 | 84 | 28 | 2 | 210 | 64 | 8.9824 | 0 |
| 1981 | 84 | 28 | 2 | 210 | 64 | 8.9826 | 0 |
| 1977 | 84 | 28 | 2 | 210 | 64 | 8.9827 | 0 |
| 1976 | 84 | 28 | 2 | 210 | 64 | 8.9828 | 0 |
| 1909 | 84 | 28 | 2 | 210 | 64 | 8.9829 | 0 |
| 1890 | 84 | 28 | 2 | 210 | 64 | 8.983 | 0 |
| 1889 | 84 | 28 | 2 | 210 | 64 | 8.9831 | 0 |
| 1888 | 84 | 28 | 2 | 210 | 64 | 8.9832 | 0 |
| 1881 | 83 | 28 | 2 | 210 | 64 | 8.9833 | 0 |
| 1865 | 83 | 28 | 2 | 210 | 64 | 8.9834 | 0 |
| 1826 | 83 | 28 | 2 | 210 | 64 | 8.9835 | 0 |
| 1941 | 89 | 29 | 2 | 210 | 65 | 8.9836 | 0 |
| 1947 | 89 | 29 | 2 | 210 | 65 | 8.9837 | 0 |
| 1945 | 89 | 29 | 2 | 210 | 65 | 8.9839 | 0 |
| 1943 | 89 | 29 | 2 | 210 | 65 | 8.9841 | 0 |
| 1942 | 89 | 29 | 2 | 210 | 65 | 8.9842 | 0 |
| 1938 | 89 | 29 | 2 | 210 | 65 | 8.9843 | 0 |

| | | | | | | | |
|---|---|---|---|---|---|---|---|
| 1936 | 89 | 29 | 2 | 210 | 65 | 8.9845 | 0 |
| 1935 | 89 | 29 | 2 | 210 | 65 | 8.9846 | 0 |
| 1816 | 89 | 29 | 2 | 210 | 65 | 8.9868 | 0 |

| MARS | TCL | DURATION | HYPERTEN | CHRONICC | EPISODES | SEX | HEMOGA1C |
|---|---|---|---|---|---|---|---|
| 10 | 180 | 6.32 | 0 | 0 | 1 | 1 | 0 |
| 10 | 180 | 5.54 | 0 | 0 | 0 | 1 | 0 |
| 10 | 180 | 6.33 | 0 | 0 | 0 | 1 | 0 |
| 10 | 180 | 5.55 | 0 | 0 | 0 | 1 | 0 |
| 10 | 180 | 6.34 | 0 | 0 | 0 | 1 | 0 |
| 10 | 180 | 5.56 | 0 | 0 | 0 | 1 | 0 |
| 10 | 180 | 6.35 | 0 | 0 | 0 | 1 | 0 |
| 10 | 180 | 5.57 | 0 | 0 | 0 | 1 | 0 |
| 10 | 180 | 6.36 | 0 | 0 | 0 | 1 | 0 |
| 10 | 180 | 5.58 | 0 | 0 | 0 | 1 | 0 |
| 10 | 180 | 6.37 | 0 | 0 | 0 | 1 | 0 |
| 10 | 180 | 5.59 | 0 | 0 | 0 | 1 | 0 |
| 9 | 180 | 7.15 | 0 | 0 | 0 | 1 | 0 |
| 9 | 180 | 7.16 | 0 | 0 | 1 | 1 | 0 |
| 9 | 180 | 6.38 | 0 | 0 | 1 | 1 | 0 |
| 9 | 180 | 5.6 | 0 | 0 | 0 | 1 | 0 |
| 9 | 180 | 7.17 | 0 | 0 | 0 | 1 | 0 |
| 9 | 180 | 7.18 | 0 | 0 | 1 | 1 | 0 |
| 9 | 180 | 7.19 | 0 | 0 | 0 | 1 | 0 |
| 9 | 180 | 7.2 | 0 | 0 | 0 | 1 | 0 |
| 9 | 180 | 7.21 | 0 | 0 | 0 | 1 | 0 |
| 9 | 180 | 17.8 | 0 | 1 | 0 | 1 | 0 |
| 9 | 180 | 17.81 | 0 | 1 | 1 | 1 | 0 |
| 9 | 180 | 17.82 | 0 | 1 | 0 | 1 | 0 |
| 9 | 180 | 17.83 | 0 | 1 | 0 | 1 | 0 |
| 9 | 180 | 17.84 | 0 | 1 | 0 | 1 | 0 |
| 9 | 180 | 17.85 | 0 | 1 | 0 | 1 | 0 |
| 9 | 180 | 17.86 | 0 | 1 | 0 | 1 | 0 |
| 9 | 180 | 17.87 | 0 | 1 | 0 | 1 | 0 |
| 9 | 180 | 24.01231 | 0 | 1 | 1 | 0 | 0 |
| 9 | 180 | 7.22 | 0 | 0 | 0 | 1 | 0 |
| 9 | 180 | 6.39 | 0 | 0 | 0 | 1 | 0 |
| 9 | 180 | 5.61 | 0 | 0 | 0 | 1 | 0 |
| 9 | 180 | 8.97 | 0 | 0 | 1 | 1 | 0 |
| 9 | 180 | 8.98 | 0 | 0 | 0 | 1 | 0 |
| 9 | 180 | 8.99 | 0 | 0 | 0 | 1 | 0 |
| 9 | 180 | 9 | 0 | 0 | 1 | 1 | 0 |
| 9 | 180 | 9.01 | 0 | 0 | 1 | 1 | 0 |
| 9 | 180 | 9.02 | 0 | 0 | 0 | 1 | 0 |
| 9 | 180 | 24.01232 | 0 | 1 | 0 | 0 | 0 |
| 9 | 195 | 24.01233 | 0 | 1 | 0 | 0 | 0 |
| 9 | 195 | 24.01206 | 0 | 1 | 0 | 0 | 0 |
| 9 | 195 | 7.23 | 0 | 0 | 0 | 1 | 0 |

| | | | | | | | |
|---|---|---|---|---|---|---|---|
| 9 | 195 | 7.24 | 0 | 0 | 0 | 1 | 0 |
| 9 | 195 | 7.25 | 0 | 0 | 1 | 1 | 0 |
| 9 | 195 | 7.26 | 0 | 0 | 0 | 1 | 0 |
| 9 | 195 | 7.27 | 0 | 0 | 0 | 1 | 0 |
| 9 | 195 | 6.4 | 0 | 0 | 0 | 1 | 0 |
| 9 | 195 | 5.23 | 0 | 0 | 0 | 1 | 0 |
| 9 | 195 | 7.28 | 0 | 0 | 0 | 1 | 0 |
| 9 | 195 | 7.29 | 0 | 0 | 0 | 1 | 0 |
| 9 | 195 | 6.16 | 0 | 0 | 1 | 1 | 0 |
| 9 | 195 | 10.78 | 0 | 1 | 0 | 0 | 0 |
| 9 | 195 | 10.79 | 0 | 1 | 0 | 0 | 0 |
| 9 | 195 | 10.8 | 0 | 1 | 0 | 0 | 0 |
| 9 | 195 | 6.41 | 0 | 0 | 0 | 1 | 0 |
| 9 | 195 | 5.24 | 0 | 0 | 0 | 1 | 0 |
| 9 | 195 | 10.81 | 0 | 1 | 0 | 0 | 0 |
| 9 | 195 | 15.3 | 0 | 0 | 1 | 0 | 0 |
| 9 | 195 | 15.31 | 0 | 0 | 0 | 0 | 0 |
| 9 | 195 | 15.32 | 0 | 0 | 0 | 0 | 0 |
| 9 | 195 | 15.33 | 0 | 0 | 0 | 0 | 0 |
| 9 | 195 | 15.34 | 0 | 0 | 0 | 0 | 0 |
| 9 | 195 | 15.35 | 0 | 0 | 0 | 0 | 0 |
| 9 | 195 | 15.36 | 0 | 0 | 0 | 0 | 0 |
| 9 | 195 | 15.37 | 0 | 0 | 0 | 0 | 0 |
| 9 | 195 | 15.38 | 0 | 0 | 0 | 0 | 0 |
| 9 | 195 | 15.39 | 0 | 0 | 0 | 0 | 0 |
| 9 | 195 | 10.82 | 0 | 1 | 0 | 0 | 0 |
| 9 | 195 | 15.4 | 0 | 0 | 0 | 0 | 0 |
| 9 | 195 | 15.41 | 0 | 0 | 0 | 0 | 0 |
| 9 | 195 | 15.42 | 0 | 1 | 0 | 0 | 0 |
| 9 | 195 | 15.43 | 0 | 1 | 0 | 0 | 0 |
| 9 | 195 | 24.012 | 0 | 1 | 0 | 0 | 0 |
| 9 | 195 | 24.01201 | 0 | 1 | 0 | 0 | 0 |
| 9 | 195 | 18.99 | 0 | 1 | 0 | 0 | 0 |
| 9 | 195 | 18.83 | 0 | 1 | 1 | 0 | 0 |
| 9 | 195 | 18.67 | 0 | 1 | 0 | 0 | 0 |
| 9 | 195 | 18.25 | 0 | 1 | 0 | 0 | 0 |
| 9 | 195 | 10.83 | 0 | 1 | 0 | 0 | 0 |
| 9 | 195 | 18.26 | 0 | 1 | 0 | 0 | 0 |
| 9 | 195 | 18.27 | 0 | 1 | 0 | 0 | 0 |
| 9 | 195 | 18.28 | 0 | 1 | 1 | 0 | 0 |
| 9 | 195 | 18.29 | 0 | 1 | 0 | 0 | 0 |
| 9 | 195 | 18.3 | 0 | 1 | 0 | 0 | 0 |
| 9 | 195 | 18.31 | 0 | 1 | 0 | 0 | 0 |
| 9 | 195 | 18.32 | 0 | 1 | 1 | 0 | 0 |

| | | | | | | | |
|---|---|---|---|---|---|---|---|
| 9 | 195 | 18.33 | 0 | 1 | 0 | 0 | 0 |
| 9 | 195 | 18.34 | 0 | 1 | 0 | 0 | 0 |
| 9 | 195 | 18.35 | 0 | 1 | 0 | 0 | 0 |
| 9 | 195 | 10.84 | 0 | 1 | 0 | 0 | 0 |
| 9 | 195 | 18.36 | 0 | 1 | 0 | 0 | 0 |
| 9 | 195 | 18.37 | 0 | 1 | 1 | 0 | 0 |
| 9 | 195 | 18.38 | 0 | 1 | 0 | 0 | 0 |
| 9 | 195 | 18.39 | 0 | 1 | 0 | 0 | 0 |
| 9 | 195 | 10.85 | 0 | 1 | 0 | 0 | 0 |
| 9 | 195 | 5.25 | 0 | 0 | 0 | 1 | 0 |
| 9 | 195 | 6.42 | 0 | 0 | 0 | 1 | 0 |
| 9 | 195 | 10.86 | 0 | 1 | 0 | 0 | 0 |
| 9 | 195 | 10.87 | 0 | 1 | 0 | 0 | 0 |
| 9 | 195 | 10.88 | 0 | 1 | 0 | 0 | 0 |
| 9 | 195 | 10.89 | 0 | 1 | 0 | 0 | 0 |
| 9 | 195 | 10.9 | 0 | 1 | 0 | 0 | 0 |
| 9 | 225 | 10.91 | 0 | 1 | 0 | 0 | 0 |
| 9 | 225 | 6.43 | 0 | 0 | 1 | 1 | 0 |
| 9 | 225 | 5.26 | 0 | 0 | 1 | 1 | 0 |
| 9 | 225 | 10.92 | 0 | 1 | 0 | 0 | 0 |
| 9 | 225 | 10.93 | 0 | 0 | 0 | 0 | 0 |
| 9 | 225 | 13.21 | 0 | 0 | 1 | 0 | 0 |
| 9 | 225 | 10.94 | 0 | 0 | 0 | 0 | 0 |
| 9 | 225 | 10.95 | 0 | 0 | 0 | 0 | 0 |
| 8 | 195 | 10.96 | 0 | 0 | 1 | 0 | 0 |
| 8 | 195 | 6.44 | 0 | 0 | 0 | 1 | 0 |
| 8 | 195 | 5.27 | 0 | 0 | 0 | 1 | 0 |
| 8 | 195 | 10.97 | 0 | 0 | 0 | 0 | 0 |
| 8 | 195 | 10.98 | 0 | 0 | 0 | 0 | 0 |
| 8 | 195 | 10.99 | 0 | 0 | 0 | 0 | 0 |
| 8 | 190 | 11 | 0 | 0 | 0 | 0 | 0 |
| 8 | 190 | 11.01 | 0 | 0 | 1 | 0 | 0 |
| 8 | 190 | 17.55 | 0 | 1 | 1 | 0 | 0 |
| 8 | 190 | 17.56 | 0 | 1 | 0 | 0 | 0 |
| 8 | 190 | 17.57 | 0 | 1 | 0 | 0 | 0 |
| 8 | 190 | 17.58 | 0 | 1 | 1 | 0 | 0 |
| 8 | 190 | 17.59 | 0 | 1 | 0 | 0 | 0 |
| 8 | 180 | 17.6 | 0 | 1 | 0 | 0 | 0 |
| 8 | 180 | 6.45 | 0 | 0 | 0 | 1 | 0 |
| 8 | 180 | 11.02 | 0 | 0 | 0 | 0 | 0 |
| 8 | 180 | 5.28 | 0 | 0 | 1 | 1 | 0 |
| 8 | 180 | 17.61 | 0 | 1 | 0 | 0 | 0 |
| 8 | 180 | 17.62 | 0 | 1 | 0 | 0 | 0 |
| 8 | 180 | 17.63 | 0 | 1 | 0 | 0 | 0 |

| | | | | | | | |
|---|---|---|---|---|---|---|---|
| 8 | 180 | 17.64 | 0 | 1 | 0 | 0 | 0 |
| 8 | 180 | 17.56 | 0 | 1 | 1 | 0 | 0 |
| 8 | 180 | 17.57 | 0 | 1 | 0 | 0 | 0 |
| 8 | 180 | 17.58 | 0 | 1 | 0 | 0 | 0 |
| 8 | 180 | 17.59 | 0 | 1 | 0 | 0 | 0 |
| 8 | 180 | 17.6 | 0 | 1 | 0 | 0 | 0 |
| 8 | 180 | 11.03 | 0 | 0 | 0 | 0 | 0 |
| 8 | 180 | 17.61 | 0 | 1 | 0 | 0 | 0 |
| 8 | 180 | 9.6 | 0 | 0 | 0 | 0 | 0 |
| 8 | 180 | 9.61 | 0 | 0 | 0 | 0 | 0 |
| 8 | 230 | 9.62 | 0 | 0 | 0 | 0 | 0 |
| 8 | 230 | 9.63 | 0 | 0 | 0 | 0 | 0 |
| 8 | 230 | 5.47 | 0 | 0 | 0 | 1 | 0 |
| 8 | 230 | 5.29 | 0 | 0 | 0 | 1 | 0 |
| 8 | 230 | 9.64 | 0 | 0 | 0 | 0 | 0 |
| 8 | 230 | 9.65 | 0 | 0 | 1 | 0 | 0 |
| 8 | 230 | 9.66 | 0 | 0 | 0 | 0 | 0 |
| 8 | 230 | 9.67 | 0 | 0 | 0 | 0 | 0 |
| 8 | 230 | 9.68 | 0 | 0 | 0 | 0 | 0 |
| 8 | 230 | 9.69 | 0 | 0 | 0 | 0 | 0 |
| 8 | 230 | 5.48 | 0 | 0 | 0 | 1 | 0 |
| 8 | 230 | 5.3 | 0 | 0 | 0 | 1 | 0 |
| 8 | 230 | 17.72 | 0 | 1 | 0 | 0 | 0 |
| 8 | 230 | 17.73 | 0 | 1 | 0 | 0 | 0 |
| 8 | 230 | 17.74 | 0 | 1 | 1 | 0 | 0 |
| 8 | 230 | 17.75 | 0 | 1 | 0 | 0 | 0 |
| 8 | 230 | 16.35 | 0 | 1 | 0 | 0 | 0 |
| 8 | 230 | 16.36 | 0 | 1 | 0 | 0 | 0 |
| 8 | 230 | 16.37 | 0 | 1 | 0 | 0 | 0 |
| 8 | 230 | 16.38 | 0 | 1 | 0 | 0 | 0 |
| 8 | 230 | 16.39 | 0 | 1 | 0 | 0 | 0 |
| 8 | 230 | 9.7 | 0 | 0 | 0 | 0 | 0 |
| 8 | 230 | 16.4 | 0 | 1 | 0 | 0 | 0 |
| 8 | 230 | 16.41 | 0 | 1 | 0 | 0 | 0 |
| 8 | 230 | 16.42 | 0 | 1 | 1 | 0 | 0 |
| 8 | 230 | 16.43 | 0 | 1 | 0 | 0 | 0 |
| 8 | 230 | 16.44 | 0 | 1 | 1 | 0 | 0 |
| 8 | 230 | 16.45 | 0 | 1 | 0 | 0 | 0 |
| 8 | 230 | 16.46 | 0 | 1 | 0 | 0 | 0 |
| 8 | 230 | 16.47 | 0 | 1 | 0 | 0 | 0 |
| 8 | 230 | 16.48 | 0 | 1 | 1 | 0 | 0 |
| 8 | 230 | 16.49 | 0 | 1 | 0 | 0 | 0 |
| 8 | 230 | 9.71 | 0 | 0 | 0 | 0 | 0 |
| 8 | 230 | 16.5 | 0 | 1 | 0 | 0 | 0 |

| | | | | | | | |
|---|---|---|---|---|---|---|---|
| 8 | 230 | 16.51 | 0 | 1 | 0 | 0 | 0 |
| 8 | 230 | 14.61 | 0 | 0 | 1 | 0 | 0 |
| 8 | 230 | 8.33 | 0 | 0 | 0 | 0 | 0 |
| 8 | 230 | 8.34 | 0 | 0 | 0 | 0 | 0 |
| 8 | 230 | 8.35 | 0 | 0 | 0 | 0 | 0 |
| 8 | 230 | 8.36 | 0 | 0 | 0 | 0 | 0 |
| 8 | 230 | 9.72 | 0 | 0 | 0 | 0 | 0 |
| 8 | 230 | 9.73 | 0 | 0 | 0 | 0 | 0 |
| 8 | 230 | 9.74 | 0 | 0 | 0 | 0 | 0 |
| 8 | 230 | 5.49 | 0 | 0 | 0 | 1 | 0 |
| 8 | 230 | 5.31 | 0 | 0 | 0 | 1 | 0 |
| 8 | 230 | 9.75 | 0 | 0 | 1 | 0 | 0 |
| 8 | 230 | 9.76 | 0 | 0 | 0 | 0 | 0 |
| 8 | 230 | 9.77 | 0 | 0 | 0 | 0 | 0 |
| 8 | 230 | 9.78 | 0 | 0 | 0 | 0 | 0 |
| 8 | 230 | 9.79 | 0 | 0 | 0 | 0 | 0 |
| 8 | 230 | 9.8 | 0 | 0 | 0 | 0 | 0 |
| 8 | 230 | 5.5 | 0 | 0 | 0 | 1 | 0 |
| 8 | 230 | 5.32 | 0 | 0 | 1 | 1 | 0 |
| 8 | 230 | 9.81 | 0 | 0 | 0 | 0 | 0 |
| 8 | 230 | 9.82 | 0 | 0 | 0 | 0 | 0 |
| 8 | 230 | 9.83 | 0 | 0 | 0 | 0 | 0 |
| 7 | 230 | 9.84 | 0 | 0 | 0 | 0 | 0 |
| 7 | 230 | 5.51 | 0 | 0 | 0 | 1 | 0 |
| 7 | 230 | 5.33 | 0 | 0 | 0 | 1 | 0 |
| 7 | 230 | 8.5 | 0 | 1 | 1 | 0 | 0 |
| 7 | 230 | 8.51 | 0 | 1 | 0 | 0 | 0 |
| 7 | 230 | 8.52 | 0 | 0 | 0 | 0 | 0 |
| 7 | 230 | 8.53 | 0 | 0 | 0 | 0 | 0 |
| 7 | 230 | 8.54 | 0 | 0 | 0 | 0 | 0 |
| 7 | 230 | 8.55 | 0 | 0 | 0 | 0 | 0 |
| 7 | 230 | 8.56 | 0 | 0 | 0 | 0 | 0 |
| 7 | 230 | 9.85 | 0 | 0 | 0 | 0 | 0 |
| 7 | 230 | 8.57 | 0 | 0 | 0 | 0 | 0 |
| 7 | 230 | 8.58 | 0 | 0 | 0 | 0 | 0 |
| 7 | 230 | 8.59 | 0 | 0 | 0 | 0 | 0 |
| 7 | 230 | 12.95 | 0 | 0 | 0 | 0 | 0 |
| 7 | 230 | 11.56 | 0 | 0 | 0 | 0 | 0 |
| 7 | 230 | 11.57 | 0 | 0 | 0 | 0 | 0 |
| 7 | 230 | 11.58 | 0 | 0 | 0 | 0 | 0 |
| 7 | 230 | 11.59 | 0 | 0 | 0 | 0 | 0 |
| 7 | 230 | 11.6 | 0 | 0 | 0 | 0 | 0 |
| 7 | 230 | 11.61 | 0 | 0 | 0 | 0 | 0 |
| 7 | 230 | 9.86 | 0 | 0 | 0 | 0 | 0 |

| | | | | | | | |
|---|---|---|---|---|---|---|---|
| 7 | 230 | 11.62 | 0 | 0 | 0 | 0 | 0 |
| 7 | 230 | 11.63 | 0 | 0 | 0 | 0 | 0 |
| 7 | 230 | 11.64 | 0 | 0 | 0 | 0 | 0 |
| 7 | 230 | 9.87 | 0 | 0 | 0 | 0 | 0 |
| 7 | 230 | 9.88 | 0 | 0 | 0 | 0 | 0 |
| 7 | 230 | 9.89 | 0 | 0 | 0 | 0 | 0 |
| 7 | 230 | 9.9 | 0 | 0 | 0 | 0 | 0 |
| 7 | 230 | 9.91 | 0 | 0 | 0 | 0 | 0 |
| 7 | 230 | 9.92 | 0 | 0 | 0 | 0 | 0 |
| 7 | 230 | 8.97 | 0 | 0 | 0 | 0 | 0 |
| 7 | 230 | 2.03 | 0 | 0 | 0 | 1 | 0 |
| 7 | 230 | 5.52 | 0 | 0 | 1 | 1 | 0 |
| 7 | 230 | 5.34 | 0 | 0 | 0 | 1 | 0 |
| 7 | 230 | 2.04 | 0 | 0 | 0 | 1 | 1 |
| 7 | 230 | 5.53 | 0 | 0 | 0 | 1 | 1 |
| 7 | 230 | 5.35 | 0 | 0 | 0 | 1 | 1 |
| 7 | 230 | 2.05 | 0 | 0 | 1 | 1 | 1 |
| 7 | 230 | 2.06 | 0 | 0 | 0 | 1 | 1 |
| 7 | 230 | 2.07 | 0 | 0 | 0 | 1 | 1 |
| 7 | 230 | 2.08 | 0 | 0 | 0 | 1 | 1 |
| 7 | 230 | 11.97 | 0 | 0 | 0 | 0 | 1 |
| 7 | 230 | 11.98 | 0 | 0 | 0 | 0 | 1 |
| 7 | 230 | 11.99 | 0 | 0 | 0 | 0 | 1 |
| 7 | 230 | 10.39 | 0 | 0 | 0 | 0 | 1 |
| 7 | 230 | 10.4 | 0 | 0 | 0 | 0 | 1 |
| 7 | 230 | 10.41 | 0 | 0 | 1 | 0 | 1 |
| 7 | 235 | 10.42 | 0 | 0 | 0 | 0 | 1 |
| 7 | 235 | 10.43 | 0 | 0 | 0 | 0 | 1 |
| 7 | 235 | 2.09 | 0 | 0 | 0 | 1 | 1 |
| 7 | 235 | 10.44 | 0 | 0 | 1 | 0 | 1 |
| 7 | 235 | 10.45 | 0 | 0 | 0 | 0 | 1 |
| 7 | 235 | 14.87 | 0 | 0 | 0 | 0 | 1 |
| 7 | 235 | 5.05 | 0 | 0 | 0 | 1 | 1 |
| 7 | 235 | 5.06 | 0 | 0 | 0 | 1 | 1 |
| 7 | 235 | 5.07 | 0 | 0 | 0 | 1 | 1 |
| 7 | 235 | 5.08 | 0 | 0 | 1 | 1 | 1 |
| 7 | 235 | 5.09 | 0 | 0 | 0 | 1 | 1 |
| 7 | 235 | 5.1 | 0 | 0 | 1 | 1 | 1 |
| 7 | 235 | 5.11 | 0 | 0 | 0 | 1 | 1 |
| 7 | 235 | 2.1 | 0 | 0 | 0 | 1 | 1 |
| 7 | 235 | 5.12 | 0 | 0 | 0 | 1 | 1 |
| 7 | 235 | 5.13 | 0 | 0 | 0 | 1 | 1 |
| 7 | 235 | 5.14 | 0 | 0 | 0 | 1 | 1 |
| 7 | 235 | 5.15 | 0 | 0 | 0 | 1 | 1 |

| | | | | | | | |
|---|---|---|---|---|---|---|---|
| 7 | 235 | 5.16 | 0 | 0 | 0 | 1 | 1 |
| 7 | 235 | 5.17 | 0 | 0 | 1 | 1 | 1 |
| 7 | 235 | 5.18 | 0 | 0 | 0 | 1 | 1 |
| 7 | 235 | 4.42 | 0 | 0 | 0 | 1 | 1 |
| 7 | 235 | 4.43 | 0 | 0 | 0 | 1 | 1 |
| 7 | 235 | 4.44 | 0 | 0 | 0 | 1 | 1 |
| 7 | 235 | 2.11 | 0 | 0 | 0 | 1 | 1 |
| 7 | 235 | 4.45 | 0 | 0 | 0 | 1 | 1 |
| 7 | 235 | 4.46 | 0 | 0 | 0 | 1 | 1 |
| 7 | 235 | 4.47 | 0 | 0 | 0 | 1 | 1 |
| 7 | 235 | 4.48 | 0 | 0 | 0 | 1 | 1 |
| 7 | 235 | 4.49 | 0 | 0 | 1 | 1 | 1 |
| 7 | 235 | 4.5 | 0 | 0 | 1 | 1 | 1 |
| 7 | 235 | 4.51 | 0 | 0 | 0 | 1 | 1 |
| 7 | 235 | 4.52 | 0 | 0 | 0 | 1 | 1 |
| 7 | 235 | 4.53 | 0 | 0 | 0 | 1 | 1 |
| 7 | 235 | 4.54 | 0 | 0 | 0 | 1 | 1 |
| 7 | 235 | 2.12 | 0 | 0 | 0 | 1 | 1 |
| 7 | 235 | 4.55 | 0 | 0 | 0 | 1 | 1 |
| 7 | 235 | 4.56 | 0 | 0 | 0 | 1 | 1 |
| 7 | 235 | 2.13 | 0 | 0 | 0 | 1 | 1 |
| 7 | 240 | 4.66 | 0 | 0 | 1 | 1 | 1 |
| 7 | 240 | 4.67 | 0 | 0 | 0 | 1 | 1 |
| 7 | 240 | 4.68 | 0 | 0 | 1 | 1 | 1 |
| 7 | 240 | 4.69 | 0 | 0 | 1 | 1 | 1 |
| 7 | 245 | 4.7 | 0 | 0 | 0 | 1 | 1 |
| 7 | 245 | 4.71 | 0 | 0 | 0 | 1 | 1 |
| 7 | 245 | 4.72 | 0 | 0 | 0 | 1 | 1 |
| 7 | 245 | 4.73 | 0 | 0 | 0 | 1 | 1 |
| 7 | 245 | 4.74 | 0 | 0 | 0 | 1 | 1 |
| 7 | 245 | 4.75 | 0 | 0 | 1 | 1 | 1 |
| 5 | 245 | 4.76 | 0 | 0 | 0 | 1 | 1 |
| 5 | 245 | 4.77 | 0 | 0 | 0 | 1 | 1 |
| 5 | 245 | 3.88 | 0 | 0 | 0 | 1 | 1 |
| 6 | 245 | 3.89 | 0 | 0 | 0 | 1 | 1 |
| 6 | 245 | 3.91 | 0 | 0 | 1 | 1 | 1 |
| 6 | 250 | 3.92 | 0 | 0 | 0 | 1 | 1 |
| 6 | 250 | 3.93 | 0 | 0 | 0 | 1 | 1 |
| 6 | 250 | 3.95 | 0 | 0 | 0 | 1 | 1 |
| 6 | 250 | 3.96 | 0 | 0 | 1 | 1 | 1 |
| 6 | 250 | 3.97 | 0 | 0 | 0 | 1 | 1 |
| 6 | 250 | 5.83 | 0 | 0 | 0 | 1 | 1 |
| 6 | 250 | 3.98 | 0 | 0 | 0 | 1 | 1 |
| 6 | 250 | 5.81 | 0 | 0 | 0 | 1 | 1 |

| | | | | | | | |
|---|---|---|---|---|---|---|---|
| 6 | 250 | 5.8 | 0 | 0 | 0 | 1 | 1 |
| 6 | 250 | 5.79 | 0 | 0 | 0 | 1 | 1 |
| 6 | 250 | 3.99 | 0 | 0 | 0 | 1 | 1 |
| 6 | 250 | 5.78 | 0 | 0 | 0 | 1 | 1 |
| 6 | 254 | 5.77 | 0 | 0 | 0 | 1 | 1 |
| 6 | 254 | 5.74 | 0 | 0 | 0 | 1 | 1 |
| 6 | 254 | 4.01 | 0 | 0 | 1 | 1 | 1 |
| 6 | 254 | 5.73 | 0 | 0 | 0 | 1 | 1 |
| 6 | 254 | 5.72 | 0 | 0 | 0 | 1 | 1 |
| 6 | 254 | 4.02 | 0 | 0 | 0 | 1 | 1 |
| 6 | 254 | 5.71 | 0 | 0 | 0 | 1 | 1 |
| 6 | 254 | 5.69 | 0 | 0 | 0 | 1 | 1 |
| 6 | 254 | 8.37 | 0 | 0 | 0 | 1 | 1 |
| 6 | 254 | 5.68 | 0 | 0 | 0 | 1 | 1 |
| 6 | 254 | 5.67 | 0 | 0 | 0 | 1 | 1 |
| 6 | 254 | 5.66 | 0 | 0 | 1 | 1 | 1 |
| 6 | 254 | 5.64 | 0 | 0 | 0 | 1 | 1 |
| 6 | 254 | 8.39 | 0 | 0 | 0 | 1 | 1 |
| 6 | 254 | 5.63 | 0 | 0 | 0 | 1 | 1 |
| 6 | 254 | 8.41 | 0 | 0 | 0 | 1 | 1 |
| 6 | 254 | 5.57 | 0 | 0 | 0 | 1 | 1 |
| 6 | 254 | 8.42 | 0 | 0 | 0 | 1 | 1 |
| 6 | 254 | 5.55 | 0 | 0 | 1 | 1 | 1 |
| 6 | 254 | 5.54 | 0 | 0 | 1 | 1 | 1 |
| 6 | 254 | 8.44 | 0 | 0 | 0 | 1 | 1 |
| 6 | 254 | 5.51 | 0 | 0 | 0 | 1 | 1 |
| 6 | 254 | 5.47 | 0 | 0 | 0 | 1 | 1 |
| 6 | 254 | 5.45 | 0 | 0 | 0 | 1 | 1 |
| 6 | 254 | 5.44 | 0 | 0 | 0 | 1 | 1 |
| 6 | 254 | 8.47 | 0 | 0 | 0 | 1 | 1 |
| 6 | 254 | 5.43 | 0 | 0 | 0 | 1 | 1 |
| 6 | 254 | 5.42 | 0 | 0 | 0 | 1 | 1 |
| 6 | 254 | 5.41 | 0 | 0 | 1 | 1 | 1 |
| 6 | 254 | 8.48 | 0 | 1 | 1 | 1 | 1 |
| 6 | 254 | 8.78 | 0 | 0 | 1 | 0 | 1 |
| 6 | 254 | 8.8 | 0 | 0 | 0 | 0 | 1 |
| 6 | 254 | 8.81 | 0 | 0 | 0 | 0 | 1 |
| 6 | 254 | 5.39 | 0 | 0 | 0 | 1 | 1 |
| 6 | 254 | 8.82 | 0 | 0 | 0 | 0 | 1 |
| 6 | 254 | 8.83 | 0 | 0 | 0 | 0 | 1 |
| 6 | 254 | 8.84 | 0 | 0 | 0 | 0 | 1 |
| 6 | 254 | 8.49 | 0 | 1 | 0 | 1 | 1 |
| 6 | 254 | 8.85 | 0 | 0 | 0 | 0 | 1 |
| 6 | 254 | 5.38 | 0 | 0 | 1 | 1 | 1 |

| | | | | | | | |
|---|---|---|---|---|---|---|---|
| 6 | 254 | 8.88 | 0 | 0 | 0 | 0 | 1 |
| 6 | 254 | 8.89 | 0 | 0 | 0 | 0 | 1 |
| 6 | 254 | 5.37 | 0 | 0 | 0 | 1 | 1 |
| 6 | 254 | 8.9 | 0 | 0 | 0 | 0 | 1 |
| 6 | 254 | 8.91 | 0 | 0 | 0 | 0 | 1 |
| 6 | 254 | 8.92 | 0 | 0 | 0 | 0 | 1 |
| 6 | 254 | 8.93 | 0 | 0 | 1 | 0 | 1 |
| 6 | 254 | 5.36 | 0 | 0 | 0 | 1 | 1 |
| 6 | 254 | 8.94 | 0 | 0 | 1 | 0 | 1 |
| 6 | 254 | 8.5 | 0 | 1 | 0 | 1 | 1 |
| 6 | 254 | 8.95 | 0 | 0 | 0 | 0 | 1 |
| 6 | 254 | 8.96 | 0 | 0 | 1 | 0 | 1 |
| 6 | 254 | 7.86 | 0 | 0 | 0 | 0 | 1 |
| 6 | 254 | 7.9 | 0 | 0 | 0 | 0 | 1 |
| 6 | 254 | 8.07 | 0 | 0 | 0 | 1 | 1 |
| 6 | 254 | 8.08 | 0 | 0 | 0 | 1 | 1 |
| 6 | 254 | 8.09 | 0 | 0 | 0 | 1 | 1 |
| 6 | 254 | 8.11 | 0 | 0 | 0 | 1 | 1 |
| 6 | 254 | 8.53 | 0 | 0 | 1 | 1 | 1 |
| 6 | 254 | 8.12 | 0 | 0 | 0 | 1 | 1 |
| 6 | 254 | 8.13 | 0 | 0 | 0 | 1 | 1 |
| 6 | 254 | 7.01 | 0 | 0 | 1 | 1 | 1 |
| 6 | 254 | 7.07 | 0 | 0 | 1 | 1 | 1 |
| 6 | 254 | 7.08 | 0 | 0 | 0 | 1 | 1 |
| 6 | 254 | 8.54 | 0 | 0 | 1 | 1 | 1 |
| 6 | 254 | 7.09 | 0 | 0 | 1 | 1 | 1 |
| 6 | 254 | 7.1 | 0 | 0 | 1 | 1 | 1 |
| 6 | 254 | 7.11 | 0 | 0 | 1 | 1 | 1 |
| 6 | 254 | 7.12 | 0 | 0 | 0 | 1 | 1 |
| 6 | 254 | 7.13 | 0 | 0 | 1 | 1 | 1 |
| 6 | 254 | 7.14 | 0 | 0 | 0 | 1 | 1 |
| 6 | 254 | 8.55 | 0 | 0 | 0 | 1 | 1 |
| 6 | 254 | 8.56 | 0 | 0 | 1 | 1 | 1 |
| 6 | 254 | 8.57 | 0 | 0 | 0 | 1 | 1 |
| 6 | 254 | 8.58 | 0 | 0 | 0 | 1 | 1 |
| 6 | 254 | 8.61 | 0 | 0 | 1 | 1 | 1 |
| 6 | 254 | 6.17 | 0 | 0 | 0 | 1 | 1 |
| 6 | 254 | 6.18 | 0 | 0 | 1 | 1 | 1 |
| 6 | 254 | 6.19 | 0 | 0 | 1 | 1 | 1 |
| 6 | 254 | 6.2 | 0 | 0 | 0 | 1 | 1 |
| 6 | 254 | 6.21 | 0 | 0 | 1 | 1 | 1 |
| 6 | 254 | 6.24 | 0 | 0 | 1 | 1 | 1 |
| 6 | 254 | 6.25 | 0 | 0 | 1 | 1 | 1 |
| 6 | 254 | 6.26 | 0 | 0 | 0 | 1 | 1 |

| | | | | | | | |
|---|---|---|---|---|---|---|---|
| 6 | 254 | 6.28 | 0 | 0 | 0 | 1 | 1 |
| 6 | 254 | 6.29 | 0 | 0 | 0 | 1 | 1 |
| 6 | 254 | 5.84 | 0 | 0 | 0 | 1 | 1 |
| 6 | 254 | 5.87 | 0 | 0 | 0 | 1 | 1 |
| 6 | 254 | 5.88 | 0 | 0 | 1 | 1 | 1 |
| 6 | 254 | 5.89 | 0 | 0 | 1 | 1 | 1 |
| 6 | 254 | 5.9 | 0 | 0 | 0 | 1 | 1 |
| 6 | 254 | 5.95 | 0 | 0 | 0 | 1 | 1 |
| 6 | 254 | 5.96 | 0 | 0 | 1 | 1 | 1 |
| 6 | 256 | 5.98 | 0 | 0 | 1 | 1 | 1 |
| 6 | 256 | 5.99 | 0 | 0 | 1 | 1 | 1 |
| 6 | 256 | 6 | 0 | 0 | 1 | 1 | 1 |
| 6 | 256 | 6.01 | 0 | 0 | 1 | 1 | 1 |
| 6 | 256 | 6.02 | 0 | 0 | 0 | 1 | 1 |
| 6 | 256 | 6.03 | 0 | 0 | 1 | 1 | 1 |
| 6 | 256 | 6.04 | 0 | 0 | 1 | 1 | 1 |
| 6 | 256 | 6.05 | 0 | 0 | 1 | 1 | 1 |
| 6 | 256 | 6.07 | 0 | 0 | 1 | 1 | 1 |
| 6 | 256 | 6.08 | 0 | 0 | 1 | 1 | 1 |
| 6 | 256 | 6.09 | 0 | 0 | 0 | 1 | 1 |
| 6 | 256 | 6.13 | 0 | 0 | 0 | 1 | 1 |
| 6 | 256 | 14.33 | 0 | 0 | 0 | 1 | 1 |
| 6 | 256 | 14.34 | 0 | 0 | 1 | 1 | 1 |
| 6 | 256 | 14.35 | 0 | 0 | 1 | 1 | 1 |
| 6 | 256 | 0.77 | 0 | 0 | 0 | 1 | 1 |
| 6 | 256 | 0.76 | 0 | 0 | 1 | 1 | 1 |
| 6 | 256 | 0.75 | 0 | 0 | 0 | 1 | 1 |
| 6 | 256 | 0.74 | 0 | 0 | 0 | 1 | 1 |
| 6 | 256 | 0.73 | 0 | 0 | 1 | 1 | 1 |
| 6 | 256 | 14.08 | 0 | 0 | 0 | 1 | 1 |
| 6 | 256 | 14.07 | 0 | 0 | 0 | 1 | 1 |
| 6 | 256 | 14.06 | 0 | 0 | 1 | 1 | 1 |
| 6 | 256 | 14.05 | 0 | 0 | 1 | 1 | 1 |
| 6 | 256 | 14.04 | 0 | 0 | 0 | 1 | 1 |
| 6 | 256 | 14.03 | 0 | 0 | 1 | 1 | 1 |
| 6 | 256 | 14.02 | 0 | 0 | 0 | 1 | 1 |
| 6 | 256 | 14.01 | 0 | 0 | 0 | 1 | 1 |
| 6 | 256 | 13.99 | 0 | 0 | 1 | 1 | 1 |
| 6 | 256 | 13.98 | 0 | 0 | 1 | 1 | 1 |
| 6 | 256 | 0.62 | 0 | 0 | 1 | 1 | 1 |
| 6 | 256 | 0.61 | 0 | 0 | 1 | 1 | 1 |
| 6 | 256 | 0.6 | 0 | 0 | 1 | 1 | 1 |
| 6 | 256 | 0.59 | 0 | 0 | 1 | 1 | 1 |
| 6 | 256 | 0.58 | 0 | 0 | 1 | 1 | 1 |

| | | | | | | | |
|---|---|---|---|---|---|---|---|
| 6 | 256 | 0.57 | 0 | 0 | 0 | 1 | 1 |
| 6 | 256 | 0.56 | 0 | 0 | 1 | 1 | 1 |
| 6 | 256 | 0.55 | 0 | 0 | 0 | 1 | 1 |
| 6 | 256 | 0.54 | 0 | 0 | 1 | 1 | 1 |
| 6 | 256 | 0.53 | 0 | 0 | 1 | 1 | 1 |
| 6 | 256 | 0.52 | 0 | 0 | 1 | 1 | 1 |
| 6 | 256 | 0.51 | 0 | 0 | 0 | 1 | 1 |
| 6 | 256 | 0.5 | 0 | 0 | 0 | 1 | 1 |
| 6 | 256 | 0.68 | 0 | 0 | 1 | 1 | 1 |
| 6 | 256 | 0.67 | 0 | 0 | 1 | 1 | 1 |
| 6 | 256 | 0.66 | 0 | 0 | 1 | 1 | 1 |
| 6 | 256 | 0.65 | 0 | 0 | 0 | 1 | 1 |
| 6 | 256 | 0.64 | 0 | 0 | 0 | 1 | 1 |
| 6 | 256 | 14.14 | 0 | 0 | 0 | 1 | 1 |
| 6 | 256 | 14.15 | 0 | 0 | 0 | 1 | 1 |
| 6 | 256 | 14.16 | 0 | 0 | 0 | 1 | 1 |
| 6 | 256 | 14.17 | 0 | 0 | 0 | 1 | 1 |
| 6 | 256 | 14.2 | 0 | 0 | 0 | 1 | 1 |
| 6 | 256 | 14.21 | 0 | 0 | 0 | 1 | 1 |
| 6 | 256 | 14.22 | 0 | 0 | 0 | 1 | 1 |
| 6 | 256 | 14.23 | 0 | 0 | 0 | 1 | 1 |
| 6 | 256 | 14.24 | 0 | 0 | 0 | 1 | 1 |
| 6 | 256 | 14.25 | 0 | 0 | 1 | 1 | 1 |
| 6 | 256 | 14.26 | 0 | 0 | 0 | 1 | 1 |
| 6 | 256 | 14.27 | 0 | 0 | 0 | 1 | 1 |
| 6 | 256 | 14.28 | 0 | 0 | 0 | 1 | 1 |
| 6 | 256 | 14.29 | 0 | 0 | 0 | 1 | 1 |
| 6 | 256 | 14.3 | 0 | 0 | 0 | 1 | 1 |
| 6 | 256 | 14.31 | 0 | 0 | 0 | 1 | 1 |
| 6 | 256 | 14.32 | 0 | 0 | 1 | 1 | 1 |
| 6 | 256 | 13.97 | 0 | 0 | 1 | 1 | 1 |
| 6 | 256 | 13.96 | 0 | 0 | 1 | 1 | 1 |
| 6 | 256 | 13.93 | 0 | 0 | 1 | 1 | 1 |
| 6 | 256 | 13.92 | 0 | 0 | 1 | 1 | 1 |
| 6 | 256 | 1.01 | 0 | 0 | 1 | 1 | 1 |
| 6 | 256 | 1 | 0 | 0 | 1 | 1 | 1 |
| 6 | 256 | 0.99 | 0 | 0 | 1 | 1 | 1 |
| 6 | 256 | 0.98 | 0 | 0 | 0 | 1 | 1 |
| 6 | 256 | 0.97 | 0 | 0 | 0 | 1 | 1 |
| 6 | 256 | 0.95 | 0 | 0 | 0 | 1 | 1 |
| 6 | 256 | 0.93 | 0 | 0 | 1 | 1 | 1 |
| 6 | 256 | 0.92 | 0 | 0 | 0 | 1 | 1 |
| 6 | 256 | 0.91 | 0 | 0 | 1 | 1 | 1 |
| 6 | 256 | 0.9 | 0 | 0 | 0 | 1 | 1 |

| | | | | | | | |
|---|---|---|---|---|---|---|---|
| 6 | 256 | 0.89 | 0 | 0 | 0 | 1 | 1 |
| 6 | 256 | 0.88 | 0 | 0 | 0 | 1 | 1 |
| 6 | 256 | 0.87 | 0 | 0 | 0 | 1 | 1 |
| 6 | 256 | 0.86 | 0 | 0 | 0 | 1 | 1 |
| 6 | 256 | 0.85 | 0 | 0 | 1 | 1 | 1 |
| 6 | 256 | 0.84 | 0 | 0 | 1 | 1 | 1 |
| 6 | 256 | 0.83 | 0 | 0 | 0 | 1 | 1 |
| 6 | 256 | 0.82 | 0 | 0 | 0 | 1 | 1 |
| 6 | 256 | 0.8 | 0 | 0 | 0 | 1 | 1 |
| 6 | 256 | 14.13 | 0 | 0 | 0 | 1 | 1 |
| 6 | 256 | 14.12 | 0 | 0 | 1 | 1 | 1 |
| 6 | 256 | 14.11 | 0 | 0 | 1 | 1 | 1 |
| 6 | 256 | 14.1 | 0 | 0 | 1 | 1 | 1 |
| 6 | 256 | 14.09 | 0 | 0 | 1 | 1 | 1 |
| 6 | 256 | 14.85 | 0 | 0 | 0 | 1 | 1 |
| 6 | 256 | 14.84 | 0 | 0 | 1 | 1 | 1 |
| 6 | 256 | 14.83 | 0 | 0 | 1 | 1 | 1 |
| 6 | 256 | 14.82 | 0 | 0 | 0 | 1 | 1 |
| 6 | 256 | 14.81 | 0 | 0 | 0 | 1 | 1 |
| 6 | 256 | 14.8 | 0 | 0 | 1 | 1 | 1 |
| 6 | 256 | 14.79 | 0 | 0 | 1 | 1 | 1 |
| 6 | 256 | 14.78 | 0 | 0 | 1 | 1 | 1 |
| 6 | 256 | 15.06 | 0 | 0 | 0 | 1 | 1 |
| 6 | 256 | 15.04 | 0 | 0 | 0 | 1 | 1 |
| 6 | 256 | 15.03 | 0 | 0 | 0 | 1 | 1 |
| 6 | 256 | 15.02 | 0 | 0 | 0 | 1 | 1 |
| 6 | 256 | 15 | 0 | 0 | 0 | 1 | 1 |
| 6 | 256 | 14.98 | 0 | 0 | 0 | 1 | 1 |
| 6 | 256 | 14.97 | 0 | 0 | 0 | 1 | 1 |
| 6 | 256 | 14.96 | 0 | 0 | 0 | 1 | 1 |
| 6 | 256 | 14.95 | 0 | 0 | 1 | 1 | 1 |
| 6 | 256 | 14.94 | 0 | 0 | 0 | 1 | 1 |
| 6 | 256 | 14.93 | 0 | 0 | 1 | 1 | 1 |
| 6 | 256 | 14.92 | 0 | 0 | 0 | 1 | 1 |
| 6 | 256 | 14.9 | 0 | 0 | 0 | 1 | 1 |
| 6 | 256 | 14.88 | 0 | 0 | 0 | 1 | 1 |
| 6 | 256 | 14.87 | 0 | 0 | 0 | 1 | 1 |
| 6 | 256 | 15.08 | 0 | 0 | 0 | 1 | 1 |
| 6 | 256 | 15.09 | 0 | 0 | 0 | 1 | 1 |
| 6 | 256 | 15.1 | 0 | 0 | 0 | 1 | 1 |
| 6 | 256 | 15.11 | 0 | 0 | 0 | 1 | 1 |
| 6 | 256 | 16.53 | 0 | 1 | 0 | 0 | 1 |
| 6 | 256 | 15.16 | 0 | 0 | 0 | 1 | 1 |
| 6 | 256 | 15.17 | 0 | 0 | 0 | 1 | 1 |

| | | | | | | | |
|---|---|---|---|---|---|---|---|
| 6 | 256 | 15.18 | 0 | 0 | 0 | 1 | 1 |
| 6 | 256 | 15.19 | 0 | 0 | 0 | 1 | 1 |
| 6 | 256 | 15.2 | 0 | 0 | 1 | 1 | 1 |
| 6 | 256 | 15.21 | 0 | 0 | 0 | 1 | 1 |
| 6 | 256 | 15.22 | 0 | 0 | 0 | 1 | 1 |
| 6 | 256 | 15.23 | 0 | 0 | 0 | 1 | 1 |
| 6 | 256 | 15.24 | 0 | 0 | 0 | 1 | 1 |
| 6 | 256 | 15.25 | 0 | 0 | 0 | 1 | 1 |
| 6 | 256 | 15.26 | 0 | 0 | 0 | 1 | 1 |
| 6 | 256 | 15.29 | 0 | 0 | 1 | 1 | 1 |
| 6 | 256 | 15.3 | 0 | 0 | 1 | 1 | 1 |
| 6 | 256 | 15.31 | 0 | 0 | 0 | 1 | 1 |
| 6 | 256 | 15.32 | 0 | 0 | 1 | 1 | 1 |
| 6 | 256 | 15.34 | 0 | 0 | 0 | 1 | 1 |
| 6 | 256 | 15.35 | 0 | 0 | 1 | 1 | 1 |
| 6 | 256 | 15.52 | 0 | 0 | 1 | 1 | 1 |
| 6 | 256 | 15.53 | 0 | 0 | 1 | 1 | 1 |
| 6 | 256 | 15.54 | 0 | 0 | 1 | 1 | 1 |
| 6 | 256 | 15.55 | 0 | 0 | 0 | 1 | 1 |
| 6 | 256 | 15.58 | 0 | 0 | 1 | 1 | 1 |
| 6 | 256 | 15.61 | 0 | 0 | 0 | 1 | 1 |
| 6 | 256 | 15.62 | 0 | 0 | 0 | 1 | 1 |
| 6 | 256 | 15.63 | 0 | 0 | 0 | 1 | 1 |
| 6 | 256 | 15.64 | 0 | 0 | 0 | 1 | 1 |
| 6 | 256 | 15.66 | 0 | 0 | 0 | 1 | 1 |
| 6 | 256 | 15.67 | 0 | 0 | 0 | 1 | 1 |
| 6 | 256 | 15.68 | 0 | 0 | 0 | 1 | 1 |
| 6 | 256 | 15.69 | 0 | 0 | 1 | 1 | 1 |
| 6 | 256 | 15.7 | 0 | 0 | 0 | 1 | 1 |
| 6 | 256 | 15.71 | 0 | 0 | 0 | 1 | 1 |
| 6 | 256 | 15.12 | 0 | 0 | 0 | 1 | 1 |
| 6 | 256 | 15.13 | 0 | 0 | 1 | 1 | 1 |
| 6 | 256 | 15.14 | 0 | 0 | 1 | 1 | 1 |
| 6 | 256 | 15.15 | 0 | 0 | 0 | 1 | 1 |
| 5 | 256 | 18.47 | 0 | 0 | 0 | 1 | 1 |
| 5 | 256 | 18.49 | 0 | 0 | 0 | 1 | 1 |
| 5 | 256 | 17.94 | 0 | 0 | 0 | 1 | 1 |
| 5 | 256 | 17.95 | 0 | 0 | 0 | 1 | 1 |
| 5 | 256 | 17.96 | 0 | 0 | 1 | 1 | 1 |
| 5 | 256 | 17.97 | 0 | 0 | 0 | 1 | 1 |
| 5 | 256 | 17.98 | 0 | 0 | 0 | 1 | 1 |
| 5 | 256 | 18 | 0 | 0 | 0 | 1 | 1 |
| 5 | 256 | 18.01 | 0 | 0 | 0 | 1 | 1 |
| 5 | 256 | 18.02 | 0 | 0 | 0 | 1 | 1 |

| | | | | | | | |
|---|---|---|---|---|---|---|---|
| 5 | 256 | 18.03 | 0 | 0 | 1 | 1 | 1 |
| 5 | 256 | 18.06 | 0 | 0 | 0 | 1 | 1 |
| 5 | 256 | 18.07 | 0 | 0 | 1 | 1 | 1 |
| 5 | 256 | 18.08 | 0 | 0 | 1 | 1 | 1 |
| 5 | 256 | 18.09 | 0 | 0 | 1 | 1 | 1 |
| 5 | 256 | 18.1 | 0 | 0 | 0 | 1 | 1 |
| 5 | 256 | 18.11 | 0 | 0 | 1 | 1 | 1 |
| 5 | 256 | 18.12 | 0 | 0 | 1 | 1 | 1 |
| 5 | 256 | 18.13 | 0 | 0 | 0 | 1 | 1 |
| 5 | 256 | 18.14 | 0 | 0 | 1 | 1 | 1 |
| 5 | 256 | 18.15 | 0 | 0 | 0 | 1 | 1 |
| 5 | 256 | 18.16 | 0 | 0 | 0 | 1 | 1 |
| 5 | 256 | 18.17 | 0 | 0 | 0 | 1 | 1 |
| 5 | 256 | 18.18 | 0 | 0 | 1 | 1 | 1 |
| 5 | 256 | 18.2 | 0 | 0 | 0 | 1 | 1 |
| 5 | 256 | 18.51 | 0 | 0 | 0 | 1 | 1 |
| 5 | 256 | 18.72 | 0 | 0 | 1 | 1 | 1 |
| 5 | 256 | 18.73 | 0 | 0 | 0 | 1 | 1 |
| 5 | 256 | 18.74 | 0 | 0 | 0 | 1 | 1 |
| 5 | 256 | 18.76 | 0 | 1 | 0 | 1 | 1 |
| 5 | 256 | 18.77 | 0 | 1 | 0 | 1 | 1 |
| 5 | 256 | 18.78 | 0 | 0 | 1 | 1 | 1 |
| 5 | 256 | 18.8 | 0 | 0 | 0 | 1 | 1 |
| 5 | 256 | 18.81 | 0 | 0 | 0 | 1 | 1 |
| 5 | 256 | 18.88 | 0 | 0 | 1 | 1 | 1 |
| 5 | 256 | 18.89 | 0 | 0 | 0 | 1 | 1 |
| 5 | 256 | 18.9 | 0 | 0 | 1 | 1 | 1 |
| 5 | 256 | 18.91 | 0 | 0 | 0 | 1 | 1 |
| 5 | 256 | 18.92 | 0 | 0 | 0 | 1 | 1 |
| 5 | 256 | 18.93 | 0 | 0 | 0 | 1 | 1 |
| 5 | 256 | 18.94 | 0 | 0 | 1 | 1 | 1 |
| 5 | 256 | 18.95 | 0 | 0 | 1 | 1 | 1 |
| 5 | 256 | 18.96 | 0 | 0 | 0 | 1 | 1 |
| 5 | 256 | 18.97 | 0 | 0 | 1 | 1 | 1 |
| 5 | 256 | 18.98 | 0 | 0 | 0 | 1 | 1 |
| 5 | 256 | 18.83 | 0 | 0 | 0 | 1 | 1 |
| 5 | 256 | 24.01 | 0 | 0 | 0 | 1 | 1 |
| 5 | 256 | 24.01 | 0 | 0 | 0 | 1 | 1 |
| 5 | 256 | 24.01 | 0 | 0 | 0 | 1 | 1 |
| 5 | 256 | 24.01 | 0 | 0 | 0 | 1 | 1 |
| 5 | 256 | 24.01 | 0 | 0 | 0 | 1 | 1 |
| 5 | 256 | 18.99 | 0 | 0 | 0 | 1 | 1 |
| 5 | 256 | 24.01 | 0 | 0 | 0 | 1 | 1 |
| 5 | 256 | 24.01 | 0 | 0 | 0 | 1 | 1 |

| | | | | | | | |
|---|---|---|---|---|---|---|---|
| 5 | 256 | 24.01 | 0 | 0 | 0 | 1 | 1 |
| 5 | 256 | 24.01 | 0 | 1 | 0 | 1 | 1 |
| 5 | 256 | 24.01 | 0 | 0 | 1 | 1 | 1 |
| 5 | 256 | 24.01 | 0 | 0 | 1 | 1 | 1 |
| 5 | 256 | 24.01 | 0 | 0 | 1 | 1 | 1 |
| 5 | 256 | 24.01 | 0 | 0 | 1 | 1 | 1 |
| 5 | 256 | 24.01 | 0 | 0 | 1 | 1 | 1 |
| 5 | 256 | 24.01 | 0 | 0 | 0 | 1 | 1 |
| 5 | 256 | 24.01 | 0 | 0 | 0 | 1 | 1 |
| 5 | 256 | 24.01 | 0 | 0 | 0 | 1 | 1 |
| 5 | 256 | 24.01 | 0 | 0 | 0 | 1 | 1 |
| 5 | 256 | 24.01 | 0 | 0 | 0 | 1 | 1 |
| 5 | 256 | 24.01 | 0 | 0 | 1 | 1 | 1 |
| 5 | 256 | 24.01 | 0 | 0 | 0 | 1 | 1 |
| 5 | 256 | 24.01 | 0 | 0 | 0 | 1 | 1 |
| 5 | 256 | 24.01 | 0 | 0 | 0 | 1 | 1 |
| 5 | 256 | 24.01 | 0 | 0 | 1 | 1 | 1 |
| 5 | 256 | 24.01 | 0 | 0 | 0 | 1 | 1 |
| 5 | 256 | 24.01 | 0 | 0 | 1 | 1 | 1 |
| 5 | 256 | 24.01 | 0 | 0 | 0 | 1 | 1 |
| 5 | 256 | 24.01 | 0 | 0 | 0 | 1 | 1 |
| 5 | 256 | 24.01 | 0 | 0 | 1 | 1 | 1 |
| 5 | 256 | 24.01 | 0 | 0 | 1 | 1 | 1 |
| 5 | 256 | 24.01 | 0 | 0 | 0 | 1 | 1 |
| 5 | 256 | 24.01 | 0 | 0 | 0 | 1 | 1 |
| 5 | 256 | 24.01 | 0 | 0 | 0 | 1 | 1 |
| 5 | 256 | 24.01 | 0 | 0 | 1 | 1 | 1 |
| 5 | 256 | 24.01 | 0 | 0 | 1 | 1 | 1 |
| 5 | 256 | 24.01 | 0 | 0 | 0 | 1 | 1 |
| 5 | 256 | 24.01 | 0 | 0 | 0 | 1 | 1 |
| 5 | 256 | 24.01 | 0 | 0 | 1 | 1 | 1 |
| 5 | 256 | 24.01 | 0 | 0 | 1 | 1 | 1 |
| 5 | 256 | 24.01 | 0 | 0 | 1 | 1 | 1 |
| 5 | 256 | 24.01 | 0 | 0 | 1 | 1 | 1 |
| 5 | 256 | 24.01 | 0 | 0 | 1 | 1 | 1 |
| 5 | 256 | 24.01 | 0 | 0 | 0 | 1 | 1 |
| 5 | 256 | 24.01 | 0 | 0 | 0 | 1 | 1 |
| 5 | 256 | 24.01 | 0 | 0 | 0 | 1 | 1 |
| 5 | 256 | 24.01 | 0 | 0 | 0 | 1 | 1 |
| 5 | 256 | 24.01 | 0 | 0 | 0 | 1 | 1 |
| 10 | 180 | 5.43 | 0 | 0 | 1 | 1 | 0 |
| 10 | 180 | 16.8 | 0 | 1 | 0 | 0 | 0 |
| 10 | 180 | 16.81 | 0 | 1 | 1 | 0 | 0 |
| 10 | 180 | 9.12 | 0 | 0 | 0 | 1 | 0 |

| | | | | | | | |
|---|---|---|---|---|---|---|---|
| 10 | 180 | 10.08 | 0 | 0 | 0 | 1 | 0 |
| 10 | 180 | 10.09 | 0 | 0 | 0 | 1 | 0 |
| 10 | 180 | 10.1 | 0 | 0 | 0 | 1 | 0 |
| 10 | 180 | 10.11 | 0 | 0 | 0 | 1 | 0 |
| 10 | 180 | 10.12 | 0 | 0 | 0 | 1 | 0 |
| 10 | 180 | 10.13 | 0 | 0 | 0 | 1 | 0 |
| 10 | 180 | 10.14 | 0 | 0 | 0 | 1 | 0 |
| 10 | 180 | 3.48 | 0 | 0 | 0 | 1 | 0 |
| 10 | 180 | 10.15 | 0 | 0 | 0 | 1 | 0 |
| 10 | 180 | 10.16 | 0 | 0 | 0 | 1 | 0 |
| 10 | 180 | 10.17 | 0 | 0 | 0 | 1 | 0 |
| 10 | 180 | 10.18 | 0 | 0 | 0 | 1 | 0 |
| 10 | 180 | 10.19 | 0 | 0 | 0 | 1 | 0 |
| 10 | 180 | 11.04 | 0 | 0 | 0 | 1 | 0 |
| 10 | 180 | 11.05 | 0 | 0 | 0 | 1 | 0 |
| 10 | 180 | 11.06 | 0 | 0 | 0 | 1 | 0 |
| 10 | 180 | 11.07 | 0 | 0 | 0 | 1 | 0 |
| 10 | 180 | 11.08 | 0 | 0 | 0 | 1 | 0 |
| 10 | 180 | 3.49 | 0 | 0 | 0 | 1 | 0 |
| 10 | 180 | 11.09 | 0 | 0 | 0 | 1 | 0 |
| 10 | 180 | 11.1 | 0 | 0 | 0 | 1 | 0 |
| 10 | 180 | 11.11 | 0 | 0 | 0 | 1 | 0 |
| 10 | 180 | 11.12 | 0 | 0 | 0 | 1 | 0 |
| 10 | 180 | 11.13 | 0 | 0 | 0 | 1 | 0 |
| 10 | 180 | 12 | 0 | 0 | 0 | 1 | 0 |
| 10 | 180 | 12.01 | 0 | 1 | 0 | 1 | 0 |
| 10 | 180 | 12.02 | 0 | 1 | 0 | 1 | 0 |
| 10 | 180 | 12.03 | 0 | 0 | 0 | 1 | 0 |
| 10 | 180 | 12.04 | 0 | 0 | 0 | 1 | 0 |
| 10 | 180 | 3.5 | 0 | 0 | 0 | 1 | 0 |
| 10 | 180 | 12.05 | 0 | 0 | 0 | 1 | 0 |
| 10 | 180 | 12.06 | 0 | 0 | 0 | 1 | 0 |
| 10 | 180 | 12.07 | 0 | 0 | 0 | 1 | 0 |
| 10 | 180 | 12.08 | 0 | 0 | 0 | 1 | 0 |
| 10 | 180 | 12.09 | 0 | 0 | 0 | 1 | 0 |
| 10 | 180 | 12.1 | 0 | 0 | 0 | 1 | 0 |
| 10 | 180 | 12.11 | 0 | 0 | 1 | 1 | 0 |
| 10 | 180 | 12.12 | 0 | 0 | 0 | 1 | 0 |
| 10 | 180 | 12.13 | 0 | 0 | 1 | 1 | 0 |
| 10 | 180 | 12.14 | 0 | 0 | 0 | 1 | 0 |
| 10 | 180 | 3.51 | 0 | 0 | 0 | 1 | 0 |
| 10 | 180 | 12.15 | 0 | 0 | 0 | 1 | 0 |
| 10 | 180 | 12.96 | 0 | 0 | 0 | 1 | 0 |
| 10 | 180 | 12.97 | 0 | 0 | 0 | 1 | 0 |

| | | | | | | | |
|---|---|---|---|---|---|---|---|
| 10 | 180 | 12.98 | 0 | 0 | 0 | 1 | 0 |
| 10 | 180 | 12.99 | 0 | 0 | 0 | 1 | 0 |
| 10 | 180 | 13 | 0 | 0 | 0 | 1 | 0 |
| 10 | 180 | 13.01 | 0 | 0 | 0 | 1 | 0 |
| 10 | 180 | 13.02 | 0 | 0 | 0 | 1 | 0 |
| 10 | 180 | 13.03 | 0 | 0 | 0 | 1 | 0 |
| 10 | 180 | 13.04 | 0 | 0 | 1 | 1 | 0 |
| 10 | 180 | 3.52 | 0 | 0 | 0 | 1 | 0 |
| 10 | 180 | 13.05 | 0 | 0 | 0 | 1 | 0 |
| 10 | 180 | 13.06 | 0 | 0 | 0 | 1 | 0 |
| 10 | 180 | 13.07 | 0 | 0 | 0 | 1 | 0 |
| 10 | 180 | 13.08 | 0 | 0 | 1 | 1 | 0 |
| 10 | 180 | 13.09 | 0 | 0 | 0 | 1 | 0 |
| 10 | 180 | 13.1 | 0 | 0 | 0 | 1 | 0 |
| 10 | 180 | 13.11 | 0 | 0 | 0 | 1 | 0 |
| 10 | 180 | 13.92 | 0 | 0 | 0 | 1 | 0 |
| 10 | 180 | 13.93 | 0 | 0 | 0 | 1 | 0 |
| 10 | 180 | 13.94 | 0 | 0 | 0 | 1 | 0 |
| 10 | 180 | 3.53 | 0 | 0 | 0 | 1 | 0 |
| 10 | 180 | 13.95 | 0 | 0 | 0 | 1 | 0 |
| 10 | 180 | 13.96 | 0 | 0 | 0 | 1 | 0 |
| 10 | 180 | 13.97 | 0 | 0 | 1 | 1 | 0 |
| 10 | 180 | 13.98 | 0 | 0 | 1 | 1 | 0 |
| 10 | 180 | 13.99 | 0 | 0 | 0 | 1 | 0 |
| 10 | 180 | 14 | 0 | 0 | 0 | 1 | 0 |
| 10 | 180 | 14.01 | 0 | 0 | 1 | 1 | 0 |
| 10 | 180 | 14.02 | 0 | 0 | 1 | 1 | 0 |
| 10 | 180 | 14.03 | 0 | 0 | 1 | 1 | 0 |
| 10 | 180 | 14.04 | 0 | 0 | 0 | 1 | 0 |
| 10 | 180 | 3.54 | 0 | 0 | 0 | 1 | 0 |
| 10 | 180 | 14.05 | 0 | 0 | 0 | 1 | 0 |
| 10 | 180 | 14.06 | 0 | 0 | 0 | 1 | 0 |
| 10 | 180 | 14.07 | 0 | 0 | 0 | 1 | 0 |
| 10 | 180 | 14.88 | 0 | 0 | 1 | 1 | 0 |
| 10 | 180 | 14.89 | 0 | 0 | 0 | 1 | 0 |
| 10 | 180 | 14.9 | 0 | 0 | 1 | 1 | 0 |
| 10 | 180 | 14.91 | 0 | 0 | 0 | 1 | 0 |
| 10 | 180 | 14.92 | 0 | 0 | 0 | 1 | 0 |
| 10 | 180 | 14.93 | 0 | 0 | 1 | 1 | 0 |
| 10 | 180 | 14.94 | 0 | 0 | 1 | 1 | 0 |
| 10 | 180 | 3.55 | 0 | 0 | 0 | 1 | 0 |
| 10 | 180 | 14.95 | 0 | 0 | 1 | 1 | 0 |
| 10 | 180 | 14.96 | 0 | 0 | 0 | 1 | 0 |
| 10 | 180 | 14.97 | 0 | 0 | 0 | 1 | 0 |

| | | | | | | | |
|---|---|---|---|---|---|---|---|
| 10 | 180 | 14.98 | 0 | 0 | 1 | 1 | 0 |
| 10 | 180 | 14.99 | 0 | 0 | 1 | 1 | 0 |
| 10 | 180 | 15 | 0 | 0 | 0 | 1 | 0 |
| 10 | 180 | 15.01 | 0 | 0 | 0 | 1 | 0 |
| 10 | 180 | 15.02 | 0 | 0 | 1 | 1 | 0 |
| 10 | 180 | 15.03 | 0 | 0 | 0 | 1 | 0 |
| 10 | 180 | 15.84 | 0 | 1 | 0 | 1 | 0 |
| 10 | 180 | 3.56 | 0 | 0 | 0 | 1 | 0 |
| 10 | 180 | 15.85 | 0 | 1 | 0 | 1 | 0 |
| 10 | 180 | 15.86 | 0 | 1 | 0 | 1 | 0 |
| 10 | 180 | 15.87 | 0 | 1 | 0 | 1 | 0 |
| 10 | 180 | 15.88 | 0 | 1 | 0 | 1 | 0 |
| 10 | 180 | 15.89 | 0 | 1 | 1 | 1 | 0 |
| 10 | 180 | 15.9 | 0 | 1 | 0 | 1 | 0 |
| 10 | 180 | 15.91 | 0 | 1 | 0 | 1 | 0 |
| 10 | 180 | 15.92 | 0 | 1 | 0 | 1 | 0 |
| 10 | 180 | 15.93 | 0 | 1 | 0 | 1 | 0 |
| 10 | 180 | 15.94 | 0 | 1 | 0 | 1 | 0 |
| 10 | 180 | 3.57 | 0 | 0 | 0 | 1 | 0 |
| 10 | 180 | 15.95 | 0 | 1 | 1 | 1 | 0 |
| 10 | 180 | 15.96 | 0 | 1 | 0 | 1 | 0 |
| 10 | 180 | 15.97 | 0 | 1 | 0 | 1 | 0 |
| 10 | 180 | 10.2 | 0 | 0 | 0 | 1 | 0 |
| 10 | 180 | 10.21 | 0 | 0 | 0 | 1 | 0 |
| 10 | 180 | 10.22 | 0 | 0 | 0 | 1 | 0 |
| 10 | 180 | 10.23 | 0 | 0 | 0 | 1 | 0 |
| 10 | 180 | 11.14 | 0 | 0 | 0 | 1 | 0 |
| 10 | 180 | 11.15 | 0 | 0 | 0 | 1 | 0 |
| 10 | 180 | 11.16 | 0 | 0 | 0 | 1 | 0 |
| 10 | 180 | 3.58 | 0 | 0 | 0 | 1 | 0 |
| 10 | 180 | 11.17 | 0 | 0 | 0 | 1 | 0 |
| 10 | 180 | 11.18 | 0 | 0 | 0 | 1 | 0 |
| 10 | 180 | 11.19 | 0 | 0 | 0 | 1 | 0 |
| 10 | 180 | 0.63 | 0 | 0 | 1 | 1 | 0 |
| 10 | 180 | 3.42 | 0 | 0 | 0 | 1 | 0 |
| 10 | 180 | 3.43 | 0 | 0 | 0 | 1 | 0 |
| 10 | 180 | 16.82 | 0 | 1 | 0 | 0 | 0 |
| 10 | 180 | 16.83 | 0 | 1 | 0 | 0 | 0 |
| 10 | 180 | 16.84 | 0 | 1 | 0 | 0 | 0 |
| 10 | 180 | 16.85 | 0 | 1 | 0 | 0 | 0 |
| 10 | 180 | 3.59 | 0 | 0 | 1 | 1 | 0 |
| 10 | 180 | 16.86 | 0 | 1 | 0 | 0 | 0 |
| 10 | 180 | 16.87 | 0 | 1 | 0 | 0 | 0 |
| 10 | 180 | 16.88 | 0 | 1 | 0 | 0 | 0 |

| | | | | | | | |
|---|---|---|---|---|---|---|---|
| 10 | 180 | 0.37 | 0 | 0 | 0 | 1 | 0 |
| 10 | 180 | 3.44 | 0 | 0 | 0 | 1 | 0 |
| 10 | 180 | 3.45 | 0 | 0 | 0 | 1 | 0 |
| 10 | 180 | 3.46 | 0 | 0 | 1 | 1 | 0 |
| 10 | 180 | 3.47 | 0 | 0 | 0 | 1 | 0 |
| 10 | 180 | 16.89 | 0 | 1 | 1 | 0 | 0 |
| 10 | 180 | 16.9 | 0 | 1 | 0 | 0 | 0 |
| 10 | 180 | 3.6 | 0 | 0 | 1 | 1 | 0 |
| 10 | 180 | 16.91 | 0 | 1 | 0 | 0 | 0 |
| 10 | 180 | 15.98 | 0 | 1 | 0 | 1 | 0 |
| 10 | 180 | 15.99 | 0 | 1 | 0 | 1 | 0 |
| 10 | 180 | 16.92 | 0 | 1 | 0 | 1 | 0 |
| 10 | 180 | 16.93 | 0 | 1 | 1 | 1 | 0 |
| 10 | 180 | 16.94 | 0 | 1 | 0 | 1 | 0 |
| 10 | 180 | 16.95 | 0 | 1 | 1 | 1 | 0 |
| 10 | 180 | 16.96 | 0 | 1 | 0 | 1 | 0 |
| 10 | 180 | 16.97 | 0 | 1 | 0 | 1 | 0 |
| 10 | 180 | 16.98 | 0 | 1 | 0 | 1 | 0 |
| 10 | 180 | 3.61 | 0 | 0 | 0 | 1 | 0 |
| 10 | 180 | 16.99 | 0 | 1 | 0 | 1 | 0 |
| 10 | 180 | 17 | 0 | 1 | 0 | 1 | 0 |
| 10 | 180 | 17.01 | 0 | 1 | 0 | 1 | 0 |
| 10 | 180 | 17.02 | 0 | 1 | 0 | 1 | 0 |
| 10 | 180 | 17.03 | 0 | 1 | 0 | 1 | 0 |
| 10 | 180 | 17.04 | 0 | 1 | 0 | 1 | 0 |
| 10 | 180 | 17.05 | 0 | 1 | 0 | 1 | 0 |
| 10 | 180 | 17.06 | 0 | 1 | 0 | 1 | 0 |
| 10 | 180 | 17.07 | 0 | 1 | 1 | 1 | 0 |
| 10 | 180 | 17.76 | 0 | 1 | 0 | 1 | 0 |
| 10 | 180 | 3.62 | 0 | 0 | 1 | 1 | 0 |
| 10 | 180 | 17.77 | 0 | 1 | 0 | 1 | 0 |
| 10 | 180 | 17.78 | 0 | 1 | 0 | 1 | 0 |
| 10 | 180 | 17.79 | 0 | 1 | 0 | 1 | 0 |
| 10 | 180 | 24.01244 | 0 | 1 | 0 | 0 | 0 |
| 10 | 180 | 24.01245 | 0 | 1 | 0 | 0 | 0 |
| 10 | 180 | 24.01242 | 0 | 1 | 0 | 0 | 0 |
| 10 | 180 | 24.01243 | 0 | 1 | 0 | 0 | 0 |
| 10 | 180 | 24.0124 | 0 | 1 | 0 | 0 | 0 |
| 10 | 180 | 24.01241 | 0 | 1 | 0 | 0 | 0 |
| 10 | 180 | 24.01238 | 0 | 1 | 1 | 0 | 0 |
| 10 | 180 | 3.63 | 0 | 0 | 0 | 1 | 0 |
| 10 | 180 | 24.01239 | 0 | 1 | 1 | 0 | 0 |
| 10 | 180 | 24.01237 | 0 | 1 | 0 | 0 | 0 |
| 9 | 180 | 24.01234 | 0 | 1 | 0 | 0 | 0 |

| | | | | | | | |
|---|---|---|---|---|---|---|---|
| 9 | 180 | 24.01235 | 0 | 1 | 0 | 0 | 0 |
| 9 | 180 | 24.01218 | 0 | 1 | 0 | 0 | 0 |
| 9 | 180 | 24.01219 | 0 | 1 | 1 | 0 | 0 |
| 9 | 180 | 24.0122 | 0 | 1 | 0 | 0 | 0 |
| 9 | 180 | 24.01221 | 0 | 1 | 0 | 0 | 0 |
| 9 | 180 | 24.01222 | 0 | 1 | 0 | 0 | 0 |
| 9 | 180 | 3.64 | 0 | 0 | 0 | 1 | 0 |
| 9 | 180 | 24.01223 | 0 | 1 | 0 | 0 | 0 |
| 9 | 180 | 24.01224 | 0 | 1 | 0 | 0 | 0 |
| 9 | 180 | 24.01225 | 0 | 1 | 0 | 0 | 0 |
| 9 | 180 | 24.01226 | 0 | 1 | 0 | 0 | 0 |
| 9 | 180 | 24.01227 | 0 | 1 | 0 | 0 | 0 |
| 9 | 180 | 24.01228 | 0 | 1 | 0 | 0 | 0 |
| 9 | 180 | 24.01229 | 0 | 1 | 0 | 0 | 0 |
| 9 | 180 | 24.0123 | 0 | 1 | 1 | 0 | 0 |
| 9 | 180 | 24.01202 | 0 | 1 | 1 | 0 | 0 |
| 9 | 180 | 24.01203 | 0 | 1 | 0 | 0 | 0 |
| 9 | 180 | 3.65 | 0 | 0 | 0 | 1 | 0 |
| 9 | 180 | 24.01204 | 0 | 1 | 1 | 0 | 0 |
| 9 | 180 | 24.01205 | 0 | 1 | 0 | 0 | 0 |
| 9 | 180 | 15.04 | 0 | 0 | 1 | 0 | 0 |
| 9 | 180 | 15.05 | 0 | 0 | 1 | 0 | 0 |
| 9 | 180 | 15.06 | 0 | 0 | 1 | 0 | 0 |
| 9 | 180 | 15.07 | 0 | 0 | 0 | 0 | 0 |
| 9 | 180 | 15.08 | 0 | 0 | 1 | 0 | 0 |
| 9 | 180 | 15.09 | 0 | 0 | 0 | 0 | 0 |
| 9 | 180 | 15.1 | 0 | 0 | 1 | 0 | 0 |
| 9 | 180 | 15.11 | 0 | 0 | 0 | 0 | 0 |
| 9 | 180 | 3.66 | 0 | 0 | 0 | 1 | 0 |
| 9 | 180 | 15.12 | 0 | 0 | 0 | 0 | 0 |
| 9 | 180 | 15.13 | 0 | 0 | 0 | 0 | 0 |
| 9 | 180 | 15.14 | 0 | 0 | 1 | 0 | 0 |
| 9 | 180 | 15.15 | 0 | 0 | 0 | 0 | 0 |
| 9 | 180 | 15.16 | 0 | 0 | 0 | 0 | 0 |
| 9 | 180 | 15.17 | 0 | 0 | 0 | 0 | 0 |
| 9 | 180 | 15.18 | 0 | 0 | 0 | 0 | 0 |
| 9 | 180 | 15.19 | 0 | 0 | 0 | 0 | 0 |
| 9 | 180 | 15.2 | 0 | 0 | 1 | 0 | 0 |
| 9 | 180 | 15.21 | 0 | 0 | 0 | 0 | 0 |
| 9 | 180 | 3.67 | 0 | 0 | 1 | 1 | 0 |
| 9 | 180 | 17.08 | 0 | 1 | 1 | 0 | 0 |
| 9 | 180 | 17.09 | 0 | 1 | 1 | 0 | 0 |
| 9 | 180 | 17.1 | 0 | 1 | 1 | 0 | 0 |
| 9 | 180 | 17.11 | 0 | 1 | 0 | 0 | 0 |

| | | | | | | | |
|---|---|---|---|---|---|---|---|
| 9 | 180 | 17.12 | 0 | 1 | 0 | 0 | 0 |
| 9 | 180 | 17.13 | 0 | 1 | 0 | 0 | 0 |
| 9 | 180 | 17.14 | 0 | 1 | 0 | 0 | 0 |
| 9 | 180 | 17.15 | 0 | 1 | 0 | 0 | 0 |
| 9 | 180 | 17.16 | 0 | 1 | 0 | 0 | 0 |
| 9 | 180 | 17.17 | 0 | 1 | 0 | 0 | 0 |
| 9 | 180 | 3.68 | 0 | 0 | 0 | 1 | 0 |
| 9 | 180 | 17.18 | 0 | 1 | 0 | 0 | 0 |
| 9 | 180 | 17.19 | 0 | 1 | 0 | 0 | 0 |
| 9 | 180 | 17.2 | 0 | 1 | 0 | 0 | 0 |
| 9 | 180 | 17.21 | 0 | 1 | 0 | 0 | 0 |
| 9 | 180 | 17.22 | 0 | 1 | 1 | 0 | 0 |
| 9 | 180 | 17.23 | 0 | 1 | 0 | 0 | 0 |
| 9 | 180 | 17.24 | 0 | 1 | 0 | 0 | 0 |
| 9 | 180 | 17.25 | 0 | 1 | 0 | 0 | 0 |
| 9 | 180 | 17.26 | 0 | 1 | 0 | 0 | 0 |
| 9 | 180 | 3.69 | 0 | 0 | 0 | 1 | 0 |
| 9 | 180 | 17.28 | 0 | 1 | 0 | 0 | 0 |
| 9 | 180 | 17.29 | 0 | 1 | 1 | 0 | 0 |
| 9 | 180 | 17.3 | 0 | 1 | 0 | 0 | 0 |
| 9 | 180 | 17.31 | 0 | 1 | 0 | 0 | 0 |
| 9 | 180 | 17.32 | 0 | 1 | 0 | 0 | 0 |
| 9 | 180 | 17.33 | 0 | 1 | 0 | 0 | 0 |
| 9 | 180 | 17.34 | 0 | 1 | 0 | 0 | 0 |
| 9 | 180 | 8.94 | 0 | 0 | 0 | 1 | 0 |
| 9 | 180 | 8.95 | 0 | 0 | 0 | 1 | 0 |
| 9 | 180 | 3.7 | 0 | 0 | 0 | 1 | 0 |
| 9 | 180 | 8.96 | 0 | 0 | 0 | 1 | 0 |
| 9 | 180 | 3.71 | 0 | 0 | 0 | 1 | 0 |
| 9 | 195 | 15.22 | 0 | 0 | 0 | 0 | 0 |
| 9 | 195 | 3.72 | 0 | 0 | 0 | 1 | 0 |
| 9 | 195 | 15.23 | 0 | 0 | 1 | 0 | 0 |
| 9 | 195 | 15.24 | 0 | 0 | 0 | 0 | 0 |
| 9 | 195 | 15.25 | 0 | 0 | 1 | 0 | 0 |
| 9 | 195 | 15.26 | 0 | 0 | 0 | 0 | 0 |
| 9 | 195 | 15.27 | 0 | 0 | 0 | 0 | 0 |
| 9 | 195 | 15.28 | 0 | 0 | 0 | 0 | 0 |
| 9 | 195 | 15.29 | 0 | 0 | 0 | 0 | 0 |
| 9 | 195 | 24.01207 | 0 | 1 | 0 | 0 | 0 |
| 9 | 195 | 24.01208 | 0 | 1 | 0 | 0 | 0 |
| 9 | 195 | 24.01209 | 0 | 1 | 0 | 0 | 0 |
| 9 | 195 | 3.73 | 0 | 0 | 1 | 1 | 0 |
| 9 | 195 | 24.0121 | 0 | 1 | 1 | 0 | 0 |
| 9 | 195 | 24.01211 | 0 | 1 | 0 | 0 | 0 |

| | | | | | | | |
|---|---|---|---|---|---|---|---|
| 9 | 195 | 24.01212 | 0 | 1 | 0 | 0 | 0 |
| 9 | 195 | 19 | 0 | 1 | 0 | 0 | 0 |
| 9 | 195 | 19.01 | 0 | 1 | 0 | 0 | 0 |
| 9 | 195 | 24.01188 | 0 | 1 | 0 | 0 | 0 |
| 9 | 195 | 24.01189 | 0 | 1 | 0 | 0 | 0 |
| 9 | 195 | 24.0119 | 0 | 1 | 0 | 0 | 0 |
| 9 | 195 | 24.01191 | 0 | 1 | 0 | 0 | 0 |
| 9 | 195 | 24.01192 | 0 | 1 | 0 | 0 | 0 |
| 9 | 195 | 3.74 | 0 | 0 | 0 | 1 | 0 |
| 9 | 195 | 24.01193 | 0 | 1 | 0 | 0 | 0 |
| 9 | 195 | 24.01194 | 0 | 1 | 0 | 0 | 0 |
| 9 | 195 | 24.01195 | 0 | 1 | 0 | 0 | 0 |
| 9 | 195 | 24.01196 | 0 | 1 | 0 | 0 | 0 |
| 9 | 195 | 24.01197 | 0 | 1 | 0 | 0 | 0 |
| 9 | 195 | 18.84 | 0 | 1 | 0 | 0 | 0 |
| 9 | 195 | 18.85 | 0 | 1 | 1 | 0 | 0 |
| 9 | 195 | 18.86 | 0 | 1 | 0 | 0 | 0 |
| 9 | 195 | 18.87 | 0 | 1 | 0 | 0 | 0 |
| 9 | 195 | 18.88 | 0 | 1 | 0 | 0 | 0 |
| 9 | 195 | 3.75 | 0 | 0 | 0 | 1 | 0 |
| 9 | 195 | 18.89 | 0 | 1 | 0 | 0 | 0 |
| 9 | 195 | 18.9 | 0 | 1 | 0 | 0 | 0 |
| 9 | 195 | 18.91 | 0 | 1 | 0 | 0 | 0 |
| 9 | 195 | 18.92 | 0 | 1 | 0 | 0 | 0 |
| 9 | 195 | 18.93 | 0 | 1 | 1 | 0 | 0 |
| 9 | 195 | 18.94 | 0 | 1 | 1 | 0 | 0 |
| 9 | 195 | 18.95 | 0 | 1 | 0 | 0 | 0 |
| 9 | 195 | 18.68 | 0 | 1 | 0 | 0 | 0 |
| 9 | 195 | 18.69 | 0 | 1 | 0 | 0 | 0 |
| 9 | 195 | 18.7 | 0 | 1 | 1 | 0 | 0 |
| 9 | 195 | 3.76 | 0 | 0 | 0 | 1 | 0 |
| 9 | 195 | 18.71 | 0 | 1 | 0 | 0 | 0 |
| 9 | 195 | 18.72 | 0 | 1 | 0 | 0 | 0 |
| 9 | 195 | 18.73 | 0 | 1 | 0 | 0 | 0 |
| 9 | 195 | 18.74 | 0 | 1 | 0 | 0 | 0 |
| 9 | 195 | 18.75 | 0 | 1 | 0 | 0 | 0 |
| 9 | 195 | 18.76 | 0 | 1 | 0 | 0 | 0 |
| 9 | 195 | 18.77 | 0 | 1 | 0 | 0 | 0 |
| 9 | 195 | 18.78 | 0 | 1 | 0 | 0 | 0 |
| 9 | 195 | 18.79 | 0 | 1 | 0 | 0 | 0 |
| 9 | 195 | 3.77 | 0 | 0 | 0 | 1 | 0 |
| 9 | 195 | 18.52 | 0 | 1 | 0 | 0 | 0 |
| 9 | 195 | 18.53 | 0 | 1 | 0 | 0 | 0 |
| 9 | 195 | 18.54 | 0 | 1 | 0 | 0 | 0 |

| | | | | | | | |
|---|---|---|---|---|---|---|---|
| 9 | 195 | 18.55 | 0 | 1 | 0 | 0 | 0 |
| 9 | 195 | 18.56 | 0 | 1 | 0 | 0 | 0 |
| 9 | 195 | 18.57 | 0 | 1 | 0 | 0 | 0 |
| 9 | 195 | 18.58 | 0 | 1 | 0 | 0 | 0 |
| 9 | 195 | 18.59 | 0 | 1 | 0 | 0 | 0 |
| 9 | 195 | 18.6 | 0 | 1 | 0 | 0 | 0 |
| 9 | 195 | 18.61 | 0 | 1 | 0 | 0 | 0 |
| 9 | 195 | 18.62 | 0 | 1 | 0 | 0 | 0 |
| 9 | 195 | 3.78 | 0 | 0 | 0 | 1 | 0 |
| 9 | 195 | 18.63 | 0 | 1 | 0 | 0 | 0 |
| 9 | 195 | 17.88 | 0 | 1 | 0 | 0 | 0 |
| 9 | 195 | 17.89 | 0 | 1 | 0 | 0 | 0 |
| 9 | 195 | 17.9 | 0 | 1 | 0 | 0 | 0 |
| 9 | 195 | 17.91 | 0 | 1 | 0 | 0 | 0 |
| 9 | 195 | 17.92 | 0 | 1 | 0 | 0 | 0 |
| 9 | 195 | 17.93 | 0 | 1 | 0 | 0 | 0 |
| 9 | 195 | 17.94 | 0 | 1 | 0 | 0 | 0 |
| 9 | 195 | 17.95 | 0 | 1 | 0 | 0 | 0 |
| 9 | 195 | 17.96 | 0 | 1 | 0 | 0 | 0 |
| 9 | 195 | 3.79 | 0 | 0 | 0 | 1 | 0 |
| 9 | 195 | 17.97 | 0 | 1 | 0 | 0 | 0 |
| 9 | 195 | 17.98 | 0 | 1 | 0 | 0 | 0 |
| 9 | 195 | 17.99 | 0 | 1 | 1 | 0 | 0 |
| 9 | 195 | 18 | 0 | 1 | 1 | 0 | 0 |
| 9 | 195 | 18.01 | 0 | 1 | 1 | 0 | 0 |
| 9 | 195 | 18.02 | 0 | 1 | 1 | 0 | 0 |
| 9 | 195 | 18.03 | 0 | 1 | 0 | 0 | 0 |
| 9 | 195 | 18.04 | 0 | 1 | 0 | 0 | 0 |
| 9 | 195 | 18.05 | 0 | 1 | 0 | 0 | 0 |
| 9 | 195 | 18.06 | 0 | 1 | 0 | 0 | 0 |
| 9 | 195 | 3.8 | 0 | 0 | 0 | 1 | 0 |
| 9 | 195 | 18.07 | 0 | 1 | 0 | 0 | 0 |
| 9 | 195 | 18.08 | 0 | 1 | 0 | 0 | 0 |
| 9 | 195 | 18.09 | 0 | 1 | 0 | 0 | 0 |
| 9 | 195 | 18.1 | 0 | 1 | 0 | 0 | 0 |
| 9 | 195 | 18.11 | 0 | 1 | 1 | 0 | 0 |
| 9 | 195 | 18.12 | 0 | 1 | 0 | 0 | 0 |
| 9 | 195 | 18.13 | 0 | 1 | 0 | 0 | 0 |
| 9 | 195 | 18.14 | 0 | 1 | 0 | 0 | 0 |
| 9 | 195 | 18.15 | 0 | 1 | 0 | 0 | 0 |
| 9 | 195 | 18.16 | 0 | 1 | 0 | 0 | 0 |
| 9 | 195 | 3.81 | 0 | 0 | 0 | 1 | 0 |
| 9 | 195 | 18.17 | 0 | 1 | 0 | 0 | 0 |
| 9 | 195 | 18.18 | 0 | 1 | 0 | 0 | 0 |

| | | | | | | | |
|---|---|---|---|---|---|---|---|
| 9 | 195 | 18.19 | 0 | 1 | 0 | 0 | 0 |
| 9 | 195 | 18.2 | 0 | 1 | 0 | 0 | 0 |
| 9 | 195 | 18.21 | 0 | 1 | 0 | 0 | 0 |
| 9 | 195 | 18.22 | 0 | 1 | 0 | 0 | 0 |
| 9 | 195 | 18.23 | 0 | 1 | 0 | 0 | 0 |
| 9 | 195 | 24.01198 | 0 | 1 | 0 | 0 | 0 |
| 9 | 195 | 24.01199 | 0 | 1 | 0 | 0 | 0 |
| 9 | 195 | 18.96 | 0 | 1 | 0 | 0 | 0 |
| 9 | 195 | 3.82 | 0 | 0 | 0 | 1 | 0 |
| 9 | 195 | 18.97 | 0 | 1 | 0 | 0 | 0 |
| 9 | 195 | 18.98 | 0 | 1 | 1 | 0 | 0 |
| 9 | 195 | 18.8 | 0 | 1 | 1 | 0 | 0 |
| 9 | 195 | 18.81 | 0 | 1 | 1 | 0 | 0 |
| 9 | 195 | 18.82 | 0 | 1 | 1 | 0 | 0 |
| 9 | 195 | 18.64 | 0 | 1 | 0 | 0 | 0 |
| 9 | 195 | 18.65 | 0 | 1 | 0 | 0 | 0 |
| 9 | 195 | 18.66 | 0 | 1 | 0 | 0 | 0 |
| 9 | 195 | 18.24 | 0 | 1 | 0 | 0 | 0 |
| 9 | 195 | 3.83 | 0 | 0 | 1 | 1 | 0 |
| 9 | 195 | 3.84 | 0 | 0 | 0 | 1 | 0 |
| 9 | 195 | 3.85 | 0 | 0 | 0 | 1 | 0 |
| 9 | 195 | 3.86 | 0 | 0 | 1 | 1 | 0 |
| 9 | 195 | 18.4 | 0 | 1 | 1 | 0 | 0 |
| 9 | 195 | 18.41 | 0 | 1 | 0 | 0 | 0 |
| 9 | 195 | 18.42 | 0 | 1 | 0 | 0 | 0 |
| 9 | 195 | 18.43 | 0 | 1 | 0 | 0 | 0 |
| 9 | 195 | 18.44 | 0 | 1 | 0 | 0 | 0 |
| 9 | 195 | 18.45 | 0 | 1 | 0 | 0 | 0 |
| 9 | 195 | 18.46 | 0 | 1 | 0 | 0 | 0 |
| 9 | 195 | 3.87 | 0 | 0 | 0 | 1 | 0 |
| 9 | 195 | 18.47 | 0 | 1 | 0 | 0 | 0 |
| 9 | 195 | 18.48 | 0 | 1 | 0 | 0 | 0 |
| 9 | 195 | 18.49 | 0 | 1 | 0 | 0 | 0 |
| 9 | 195 | 18.5 | 0 | 1 | 0 | 0 | 0 |
| 9 | 195 | 18.51 | 0 | 1 | 0 | 0 | 0 |
| 9 | 195 | 17.36 | 0 | 1 | 0 | 0 | 0 |
| 9 | 195 | 17.37 | 0 | 1 | 0 | 0 | 0 |
| 9 | 195 | 17.38 | 0 | 1 | 0 | 0 | 0 |
| 9 | 195 | 17.39 | 0 | 1 | 1 | 0 | 0 |
| 9 | 195 | 14.08 | 0 | 0 | 0 | 0 | 0 |
| 9 | 195 | 3.88 | 0 | 0 | 0 | 1 | 0 |
| 9 | 195 | 14.09 | 0 | 0 | 0 | 0 | 0 |
| 9 | 195 | 14.1 | 0 | 0 | 0 | 0 | 0 |
| 9 | 195 | 14.11 | 0 | 0 | 1 | 0 | 0 |

| | | | | | | | |
|---|---|---|---|---|---|---|---|
| 9 | 195 | 14.12 | 0 | 0 | 0 | 0 | 0 |
| 9 | 195 | 14.13 | 0 | 0 | 0 | 0 | 0 |
| 9 | 195 | 14.14 | 0 | 0 | 0 | 0 | 0 |
| 9 | 195 | 14.15 | 0 | 0 | 0 | 0 | 0 |
| 9 | 195 | 14.16 | 0 | 0 | 0 | 0 | 0 |
| 9 | 195 | 14.17 | 0 | 0 | 0 | 0 | 0 |
| 9 | 195 | 14.18 | 0 | 0 | 0 | 0 | 0 |
| 9 | 195 | 3.89 | 0 | 0 | 0 | 1 | 0 |
| 9 | 195 | 14.19 | 0 | 1 | 0 | 0 | 0 |
| 9 | 195 | 14.2 | 0 | 1 | 0 | 0 | 0 |
| 9 | 195 | 14.21 | 0 | 1 | 0 | 0 | 0 |
| 9 | 195 | 14.22 | 0 | 1 | 0 | 0 | 0 |
| 9 | 195 | 14.23 | 0 | 1 | 0 | 0 | 0 |
| 9 | 195 | 14.24 | 0 | 1 | 0 | 0 | 0 |
| 9 | 195 | 14.25 | 0 | 1 | 0 | 0 | 0 |
| 9 | 195 | 14.26 | 0 | 0 | 0 | 0 | 0 |
| 9 | 195 | 14.27 | 0 | 0 | 0 | 0 | 0 |
| 9 | 195 | 14.28 | 0 | 0 | 0 | 0 | 0 |
| 9 | 195 | 3.9 | 0 | 0 | 0 | 1 | 0 |
| 9 | 195 | 14.29 | 0 | 0 | 0 | 0 | 0 |
| 9 | 195 | 14.3 | 0 | 0 | 1 | 0 | 0 |
| 9 | 195 | 14.31 | 0 | 0 | 0 | 0 | 0 |
| 9 | 195 | 14.32 | 0 | 0 | 0 | 0 | 0 |
| 9 | 195 | 14.33 | 0 | 0 | 1 | 0 | 0 |
| 9 | 195 | 14.34 | 0 | 0 | 0 | 0 | 0 |
| 9 | 195 | 14.35 | 0 | 0 | 0 | 0 | 0 |
| 9 | 195 | 14.36 | 0 | 0 | 0 | 0 | 0 |
| 9 | 195 | 14.37 | 0 | 0 | 0 | 0 | 0 |
| 9 | 195 | 14.38 | 0 | 0 | 0 | 0 | 0 |
| 9 | 195 | 3.91 | 0 | 0 | 1 | 1 | 0 |
| 9 | 195 | 14.39 | 0 | 0 | 0 | 0 | 0 |
| 9 | 195 | 14.4 | 0 | 0 | 0 | 0 | 0 |
| 9 | 195 | 14.41 | 0 | 0 | 0 | 0 | 0 |
| 9 | 195 | 14.42 | 0 | 0 | 0 | 0 | 0 |
| 9 | 195 | 14.43 | 0 | 0 | 0 | 0 | 0 |
| 9 | 195 | 17.4 | 0 | 1 | 0 | 0 | 0 |
| 9 | 195 | 14.44 | 0 | 0 | 1 | 0 | 0 |
| 9 | 195 | 14.45 | 0 | 0 | 0 | 0 | 0 |
| 9 | 195 | 14.46 | 0 | 0 | 0 | 0 | 0 |
| 9 | 195 | 14.47 | 0 | 0 | 0 | 0 | 0 |
| 9 | 195 | 3.92 | 0 | 0 | 0 | 1 | 0 |
| 9 | 195 | 14.48 | 0 | 0 | 0 | 0 | 0 |
| 9 | 195 | 14.49 | 0 | 0 | 0 | 0 | 0 |
| 9 | 195 | 14.5 | 0 | 0 | 0 | 0 | 0 |

| | | | | | | | |
|---|---|---|---|---|---|---|---|
| 9 | 195 | 14.51 | 0 | 0 | 0 | 0 | 0 |
| 9 | 225 | 14.52 | 0 | 0 | 0 | 0 | 0 |
| 9 | 225 | 14.53 | 0 | 0 | 0 | 0 | 0 |
| 9 | 225 | 14.54 | 0 | 0 | 0 | 0 | 0 |
| 9 | 225 | 14.55 | 0 | 0 | 0 | 0 | 0 |
| 9 | 225 | 14.56 | 0 | 0 | 0 | 0 | 0 |
| 9 | 225 | 14.57 | 0 | 0 | 0 | 0 | 0 |
| 9 | 225 | 3.93 | 0 | 0 | 0 | 1 | 0 |
| 9 | 225 | 14.58 | 0 | 0 | 0 | 0 | 0 |
| 9 | 225 | 14.59 | 0 | 0 | 1 | 0 | 0 |
| 9 | 225 | 14.6 | 0 | 0 | 0 | 0 | 0 |
| 9 | 225 | 14.61 | 0 | 0 | 0 | 0 | 0 |
| 9 | 225 | 14.62 | 0 | 0 | 0 | 0 | 0 |
| 9 | 225 | 14.63 | 0 | 0 | 0 | 0 | 0 |
| 9 | 225 | 14.64 | 0 | 0 | 0 | 0 | 0 |
| 9 | 225 | 14.65 | 0 | 0 | 0 | 0 | 0 |
| 9 | 225 | 14.66 | 0 | 0 | 1 | 0 | 0 |
| 9 | 225 | 14.67 | 0 | 0 | 0 | 0 | 0 |
| 9 | 225 | 3.94 | 0 | 0 | 0 | 1 | 0 |
| 9 | 225 | 14.68 | 0 | 0 | 0 | 0 | 0 |
| 9 | 225 | 14.69 | 0 | 0 | 0 | 0 | 0 |
| 9 | 225 | 14.7 | 0 | 0 | 0 | 0 | 0 |
| 9 | 225 | 14.71 | 0 | 0 | 0 | 0 | 0 |
| 9 | 225 | 14.72 | 0 | 0 | 0 | 0 | 0 |
| 9 | 225 | 14.73 | 0 | 0 | 1 | 0 | 0 |
| 9 | 225 | 13.12 | 0 | 1 | 1 | 0 | 0 |
| 9 | 225 | 13.13 | 0 | 1 | 1 | 0 | 0 |
| 9 | 225 | 13.14 | 0 | 0 | 0 | 0 | 0 |
| 9 | 225 | 13.15 | 0 | 0 | 1 | 0 | 0 |
| 9 | 225 | 3.95 | 0 | 0 | 0 | 1 | 0 |
| 9 | 225 | 13.16 | 0 | 0 | 0 | 0 | 0 |
| 9 | 225 | 13.17 | 0 | 0 | 0 | 0 | 0 |
| 9 | 225 | 13.18 | 0 | 0 | 0 | 0 | 0 |
| 9 | 225 | 13.19 | 0 | 0 | 1 | 0 | 0 |
| 9 | 225 | 13.2 | 0 | 0 | 1 | 0 | 0 |
| 9 | 225 | 13.22 | 0 | 0 | 0 | 0 | 0 |
| 9 | 225 | 13.23 | 0 | 0 | 0 | 0 | 0 |
| 9 | 225 | 13.24 | 0 | 0 | 0 | 0 | 0 |
| 9 | 225 | 13.25 | 0 | 0 | 0 | 0 | 0 |
| 9 | 225 | 3.96 | 0 | 0 | 0 | 1 | 0 |
| 9 | 225 | 13.26 | 0 | 0 | 0 | 0 | 0 |
| 9 | 225 | 13.27 | 0 | 0 | 0 | 0 | 0 |
| 9 | 225 | 13.28 | 0 | 0 | 0 | 0 | 0 |
| 9 | 225 | 13.29 | 0 | 0 | 1 | 0 | 0 |

| | | | | | | | |
|---|---|---|---|---|---|---|---|
| 9 | 225 | 13.3 | 0 | 0 | 0 | 0 | 0 |
| 9 | 225 | 13.31 | 0 | 0 | 0 | 0 | 0 |
| 9 | 225 | 13.32 | 0 | 0 | 0 | 0 | 0 |
| 9 | 225 | 13.33 | 0 | 0 | 0 | 0 | 0 |
| 9 | 225 | 13.34 | 0 | 0 | 0 | 0 | 0 |
| 8 | 225 | 3.97 | 0 | 0 | 0 | 1 | 0 |
| 9 | 225 | 13.35 | 0 | 0 | 0 | 0 | 0 |
| 8 | 225 | 13.36 | 0 | 0 | 0 | 0 | 0 |
| 8 | 225 | 13.37 | 0 | 0 | 0 | 0 | 0 |
| 8 | 225 | 13.38 | 0 | 0 | 0 | 0 | 0 |
| 8 | 225 | 13.39 | 0 | 0 | 0 | 0 | 0 |
| 8 | 225 | 13.4 | 0 | 0 | 0 | 0 | 0 |
| 8 | 225 | 13.41 | 0 | 0 | 0 | 0 | 0 |
| 8 | 225 | 13.42 | 0 | 0 | 0 | 0 | 0 |
| 8 | 225 | 13.43 | 0 | 0 | 0 | 0 | 0 |
| 8 | 225 | 13.44 | 0 | 0 | 0 | 0 | 0 |
| 8 | 225 | 3.98 | 0 | 0 | 0 | 1 | 0 |
| 8 | 195 | 13.45 | 0 | 0 | 0 | 0 | 0 |
| 8 | 195 | 13.46 | 0 | 0 | 0 | 0 | 0 |
| 8 | 195 | 13.47 | 0 | 0 | 0 | 0 | 0 |
| 8 | 195 | 13.48 | 0 | 0 | 0 | 0 | 0 |
| 8 | 195 | 13.49 | 0 | 0 | 0 | 0 | 0 |
| 8 | 195 | 13.5 | 0 | 0 | 0 | 0 | 0 |
| 8 | 195 | 13.51 | 0 | 0 | 0 | 0 | 0 |
| 8 | 195 | 13.52 | 0 | 0 | 0 | 0 | 0 |
| 8 | 195 | 13.53 | 0 | 0 | 0 | 0 | 0 |
| 8 | 195 | 13.54 | 0 | 0 | 0 | 0 | 0 |
| 8 | 195 | 13.55 | 0 | 0 | 0 | 0 | 0 |
| 8 | 195 | 3.99 | 0 | 0 | 0 | 1 | 0 |
| 8 | 195 | 13.56 | 0 | 0 | 0 | 0 | 0 |
| 8 | 195 | 13.57 | 0 | 0 | 0 | 0 | 0 |
| 8 | 195 | 13.58 | 0 | 0 | 0 | 0 | 0 |
| 8 | 195 | 13.59 | 0 | 0 | 0 | 0 | 0 |
| 8 | 195 | 13.6 | 0 | 0 | 0 | 0 | 0 |
| 8 | 195 | 13.61 | 0 | 0 | 0 | 0 | 0 |
| 8 | 195 | 13.62 | 0 | 0 | 0 | 0 | 0 |
| 8 | 195 | 13.63 | 0 | 0 | 0 | 0 | 0 |
| 8 | 195 | 13.64 | 0 | 0 | 1 | 0 | 0 |
| 8 | 195 | 13.65 | 0 | 0 | 0 | 0 | 0 |
| 8 | 195 | 4 | 0 | 0 | 1 | 1 | 0 |
| 8 | 195 | 13.66 | 0 | 0 | 0 | 0 | 0 |
| 8 | 195 | 13.67 | 0 | 0 | 0 | 0 | 0 |
| 8 | 195 | 13.68 | 0 | 0 | 0 | 0 | 0 |
| 8 | 195 | 13.69 | 0 | 0 | 0 | 0 | 0 |

| | | | | | | | |
|---|---|---|---|---|---|---|---|
| 8 | 195 | 13.7 | 0 | 0 | 1 | 0 | 0 |
| 8 | 195 | 13.71 | 0 | 0 | 1 | 0 | 0 |
| 8 | 195 | 13.72 | 0 | 0 | 0 | 0 | 0 |
| 8 | 195 | 13.73 | 0 | 0 | 0 | 0 | 0 |
| 8 | 195 | 13.74 | 0 | 0 | 0 | 0 | 0 |
| 8 | 195 | 13.75 | 0 | 0 | 0 | 0 | 0 |
| 8 | 195 | 4.01 | 0 | 0 | 0 | 1 | 0 |
| 8 | 190 | 13.76 | 0 | 0 | 0 | 0 | 0 |
| 8 | 190 | 13.77 | 0 | 0 | 0 | 0 | 0 |
| 8 | 190 | 13.78 | 0 | 0 | 0 | 0 | 0 |
| 8 | 190 | 13.79 | 0 | 0 | 0 | 0 | 0 |
| 8 | 190 | 13.8 | 0 | 0 | 0 | 0 | 0 |
| 8 | 190 | 13.81 | 0 | 0 | 0 | 0 | 0 |
| 8 | 190 | 13.82 | 0 | 0 | 0 | 0 | 0 |
| 8 | 190 | 13.83 | 0 | 0 | 0 | 0 | 0 |
| 8 | 190 | 13.84 | 0 | 0 | 0 | 0 | 0 |
| 8 | 190 | 13.85 | 0 | 0 | 0 | 0 | 0 |
| 8 | 190 | 4.02 | 0 | 0 | 0 | 1 | 0 |
| 8 | 190 | 13.86 | 0 | 0 | 0 | 0 | 0 |
| 8 | 190 | 13.87 | 0 | 0 | 0 | 0 | 0 |
| 8 | 190 | 13.88 | 0 | 0 | 0 | 0 | 0 |
| 8 | 190 | 13.89 | 0 | 0 | 0 | 0 | 0 |
| 8 | 190 | 13.9 | 0 | 0 | 0 | 0 | 0 |
| 8 | 190 | 13.91 | 0 | 0 | 0 | 0 | 0 |
| 8 | 190 | 12.16 | 0 | 0 | 0 | 0 | 0 |
| 8 | 190 | 12.17 | 0 | 0 | 0 | 0 | 0 |
| 8 | 190 | 12.18 | 0 | 0 | 0 | 0 | 0 |
| 8 | 190 | 17.5 | 0 | 1 | 0 | 0 | 0 |
| 8 | 190 | 4.03 | 0 | 0 | 0 | 1 | 0 |
| 8 | 190 | 17.51 | 0 | 1 | 0 | 0 | 0 |
| 8 | 190 | 17.52 | 0 | 1 | 0 | 0 | 0 |
| 8 | 190 | 17.53 | 0 | 1 | 0 | 0 | 0 |
| 8 | 190 | 17.54 | 0 | 1 | 1 | 0 | 0 |
| 8 | 180 | 4.04 | 0 | 0 | 0 | 1 | 0 |
| 8 | 180 | 4.05 | 0 | 0 | 0 | 1 | 0 |
| 8 | 180 | 17.65 | 0 | 1 | 1 | 0 | 0 |
| 8 | 180 | 17.66 | 0 | 1 | 0 | 0 | 0 |
| 8 | 180 | 17.67 | 0 | 1 | 0 | 0 | 0 |
| 8 | 180 | 17.68 | 0 | 1 | 0 | 0 | 0 |
| 8 | 180 | 17.69 | 0 | 1 | 1 | 0 | 0 |
| 8 | 180 | 17.7 | 0 | 1 | 0 | 0 | 0 |
| 8 | 180 | 17.71 | 0 | 1 | 0 | 0 | 0 |
| 8 | 180 | 17.72 | 0 | 1 | 0 | 0 | 0 |
| 8 | 180 | 17.73 | 0 | 1 | 0 | 0 | 0 |

| | | | | | | | |
|---|---|---|---|---|---|---|---|
| 8 | 180 | 17.74 | 0 | 1 | 0 | 0 | 0 |
| 8 | 180 | 4.06 | 0 | 0 | 0 | 1 | 0 |
| 8 | 180 | 15.72 | 0 | 1 | 0 | 0 | 0 |
| 8 | 180 | 15.73 | 0 | 1 | 0 | 0 | 0 |
| 8 | 180 | 15.74 | 0 | 1 | 0 | 0 | 0 |
| 8 | 180 | 15.75 | 0 | 1 | 0 | 0 | 0 |
| 8 | 180 | 15.76 | 0 | 1 | 0 | 0 | 0 |
| 8 | 180 | 15.77 | 0 | 1 | 0 | 0 | 0 |
| 8 | 180 | 15.78 | 0 | 1 | 0 | 0 | 0 |
| 8 | 180 | 15.79 | 0 | 1 | 0 | 0 | 0 |
| 8 | 180 | 15.8 | 0 | 1 | 0 | 0 | 0 |
| 8 | 180 | 15.81 | 0 | 1 | 0 | 0 | 0 |
| 8 | 180 | 4.07 | 0 | 0 | 0 | 1 | 0 |
| 8 | 180 | 15.82 | 0 | 1 | 1 | 0 | 0 |
| 8 | 180 | 15.83 | 0 | 1 | 0 | 0 | 0 |
| 8 | 180 | 15.84 | 0 | 1 | 0 | 0 | 0 |
| 8 | 180 | 15.85 | 0 | 1 | 0 | 0 | 0 |
| 8 | 180 | 15.86 | 0 | 1 | 0 | 0 | 0 |
| 8 | 180 | 15.87 | 0 | 1 | 0 | 0 | 0 |
| 8 | 180 | 15.88 | 0 | 1 | 0 | 0 | 0 |
| 8 | 180 | 15.89 | 0 | 1 | 0 | 0 | 0 |
| 8 | 180 | 15.9 | 0 | 1 | 0 | 0 | 0 |
| 8 | 230 | 15.91 | 0 | 1 | 1 | 0 | 0 |
| 8 | 180 | 4.08 | 0 | 0 | 0 | 1 | 0 |
| 8 | 230 | 15.92 | 0 | 1 | 0 | 0 | 0 |
| 8 | 230 | 15.93 | 0 | 1 | 0 | 0 | 0 |
| 8 | 230 | 15.94 | 0 | 1 | 0 | 0 | 0 |
| 8 | 230 | 15.95 | 0 | 1 | 1 | 0 | 0 |
| 8 | 230 | 15.96 | 0 | 1 | 0 | 0 | 0 |
| 8 | 230 | 15.97 | 0 | 1 | 0 | 0 | 0 |
| 8 | 230 | 15.98 | 0 | 1 | 0 | 0 | 0 |
| 8 | 230 | 15.99 | 0 | 1 | 0 | 0 | 0 |
| 8 | 230 | 16 | 0 | 1 | 0 | 0 | 0 |
| 8 | 230 | 16.01 | 0 | 1 | 0 | 0 | 0 |
| 8 | 230 | 4.09 | 0 | 0 | 0 | 1 | 0 |
| 8 | 230 | 16.02 | 0 | 1 | 0 | 0 | 0 |
| 8 | 230 | 16.03 | 0 | 1 | 0 | 0 | 0 |
| 8 | 230 | 16.04 | 0 | 1 | 0 | 0 | 0 |
| 8 | 230 | 16.05 | 0 | 1 | 0 | 0 | 0 |
| 8 | 230 | 16.06 | 0 | 1 | 1 | 0 | 0 |
| 8 | 230 | 16.07 | 0 | 1 | 0 | 0 | 0 |
| 8 | 230 | 16.08 | 0 | 1 | 0 | 0 | 0 |
| 8 | 230 | 16.09 | 0 | 1 | 0 | 0 | 0 |
| 8 | 230 | 16.1 | 0 | 1 | 0 | 0 | 0 |

| | | | | | | | |
|---|---|---|---|---|---|---|---|
| 8 | 230 | 16.11 | 0 | 1 | 0 | 0 | 0 |
| 8 | 230 | 4.1 | 0 | 0 | 0 | 1 | 0 |
| 8 | 230 | 16.12 | 0 | 1 | 0 | 0 | 0 |
| 8 | 230 | 16.13 | 0 | 1 | 0 | 0 | 0 |
| 8 | 230 | 16.14 | 0 | 1 | 0 | 0 | 0 |
| 8 | 230 | 16.15 | 0 | 1 | 0 | 0 | 0 |
| 8 | 230 | 16.16 | 0 | 1 | 1 | 0 | 0 |
| 8 | 230 | 16.17 | 0 | 1 | 1 | 0 | 0 |
| 8 | 230 | 16.18 | 0 | 1 | 1 | 0 | 0 |
| 8 | 230 | 16.19 | 0 | 1 | 1 | 0 | 0 |
| 8 | 230 | 16.2 | 0 | 1 | 0 | 0 | 0 |
| 8 | 230 | 16.21 | 0 | 1 | 1 | 0 | 0 |
| 8 | 230 | 4.11 | 0 | 0 | 0 | 1 | 0 |
| 8 | 230 | 16.22 | 0 | 1 | 1 | 0 | 0 |
| 8 | 230 | 16.23 | 0 | 1 | 0 | 0 | 0 |
| 8 | 230 | 16.24 | 0 | 1 | 0 | 0 | 0 |
| 8 | 230 | 16.25 | 0 | 1 | 1 | 0 | 0 |
| 8 | 230 | 16.26 | 0 | 1 | 0 | 0 | 0 |
| 8 | 230 | 16.27 | 0 | 1 | 0 | 0 | 0 |
| 8 | 230 | 16.28 | 0 | 1 | 0 | 0 | 0 |
| 8 | 230 | 16.29 | 0 | 1 | 0 | 0 | 0 |
| 8 | 230 | 16.3 | 0 | 1 | 0 | 0 | 0 |
| 8 | 230 | 16.31 | 0 | 1 | 1 | 0 | 0 |
| 8 | 230 | 4.12 | 0 | 0 | 1 | 1 | 0 |
| 8 | 230 | 14.38 | 0 | 1 | 0 | 0 | 0 |
| 8 | 230 | 14.39 | 0 | 0 | 0 | 0 | 0 |
| 8 | 230 | 14.4 | 0 | 0 | 0 | 0 | 0 |
| 8 | 230 | 14.41 | 0 | 0 | 0 | 0 | 0 |
| 8 | 230 | 14.42 | 0 | 0 | 0 | 0 | 0 |
| 8 | 230 | 14.43 | 0 | 0 | 0 | 0 | 0 |
| 8 | 230 | 14.44 | 0 | 0 | 0 | 0 | 0 |
| 8 | 230 | 14.45 | 0 | 0 | 0 | 0 | 0 |
| 8 | 230 | 14.46 | 0 | 0 | 0 | 0 | 0 |
| 8 | 230 | 14.47 | 0 | 0 | 0 | 0 | 0 |
| 8 | 230 | 4.13 | 0 | 0 | 0 | 1 | 0 |
| 8 | 230 | 14.48 | 0 | 0 | 1 | 0 | 0 |
| 8 | 230 | 14.49 | 0 | 0 | 0 | 0 | 0 |
| 8 | 230 | 14.5 | 0 | 0 | 0 | 0 | 0 |
| 8 | 230 | 14.51 | 0 | 0 | 0 | 0 | 0 |
| 8 | 230 | 14.52 | 0 | 0 | 0 | 0 | 0 |
| 8 | 230 | 14.53 | 0 | 0 | 0 | 0 | 0 |
| 8 | 230 | 14.54 | 0 | 0 | 0 | 0 | 0 |
| 8 | 230 | 14.55 | 0 | 0 | 0 | 0 | 0 |
| 8 | 230 | 14.56 | 0 | 0 | 0 | 0 | 0 |

| | | | | | | | |
|---|---|---|---|---|---|---|---|
| 8 | 230 | 14.57 | 0 | 0 | 0 | 0 | 0 |
| 8 | 230 | 4.14 | 0 | 0 | 0 | 1 | 0 |
| 8 | 230 | 8.28 | 0 | 0 | 0 | 0 | 0 |
| 8 | 230 | 8.29 | 0 | 0 | 1 | 0 | 0 |
| 8 | 230 | 8.3 | 0 | 0 | 0 | 0 | 0 |
| 8 | 230 | 8.31 | 0 | 0 | 0 | 0 | 0 |
| 8 | 230 | 8.32 | 0 | 0 | 1 | 0 | 0 |
| 8 | 230 | 16.32 | 0 | 1 | 1 | 0 | 0 |
| 8 | 230 | 16.33 | 0 | 1 | 0 | 0 | 0 |
| 8 | 230 | 16.34 | 0 | 1 | 0 | 0 | 0 |
| 8 | 230 | 14.58 | 0 | 0 | 0 | 0 | 0 |
| 8 | 230 | 14.59 | 0 | 0 | 0 | 0 | 0 |
| 8 | 230 | 4.15 | 0 | 0 | 0 | 1 | 0 |
| 8 | 230 | 14.6 | 0 | 0 | 0 | 0 | 0 |
| 8 | 230 | 4.16 | 0 | 0 | 0 | 1 | 0 |
| 8 | 230 | 4.17 | 0 | 0 | 0 | 1 | 0 |
| 8 | 230 | 8.37 | 0 | 0 | 0 | 0 | 0 |
| 8 | 230 | 8.38 | 0 | 0 | 0 | 0 | 0 |
| 8 | 230 | 8.39 | 0 | 0 | 0 | 0 | 0 |
| 8 | 230 | 4.18 | 0 | 0 | 0 | 1 | 0 |
| 8 | 230 | 8.4 | 0 | 0 | 0 | 0 | 0 |
| 8 | 230 | 8.41 | 0 | 0 | 0 | 0 | 0 |
| 8 | 230 | 8.42 | 0 | 0 | 0 | 0 | 0 |
| 8 | 230 | 8.43 | 0 | 0 | 0 | 0 | 0 |
| 8 | 230 | 8.44 | 0 | 0 | 0 | 0 | 0 |
| 8 | 230 | 8.45 | 0 | 0 | 0 | 0 | 0 |
| 8 | 230 | 8.46 | 0 | 0 | 0 | 0 | 0 |
| 8 | 230 | 8.47 | 0 | 0 | 0 | 0 | 0 |
| 8 | 230 | 8.48 | 0 | 1 | 1 | 0 | 0 |
| 8 | 230 | 8.49 | 0 | 1 | 0 | 0 | 0 |
| 8 | 230 | 4.19 | 0 | 0 | 0 | 1 | 0 |
| 8 | 230 | 12.19 | 0 | 0 | 0 | 0 | 0 |
| 8 | 230 | 12.2 | 0 | 0 | 0 | 0 | 0 |
| 8 | 230 | 12.21 | 0 | 0 | 0 | 0 | 0 |
| 8 | 230 | 12.22 | 0 | 0 | 0 | 0 | 0 |
| 8 | 230 | 12.23 | 0 | 0 | 0 | 0 | 0 |
| 8 | 230 | 12.24 | 0 | 0 | 0 | 0 | 0 |
| 8 | 230 | 12.25 | 0 | 0 | 0 | 0 | 0 |
| 8 | 230 | 12.26 | 0 | 0 | 0 | 0 | 0 |
| 8 | 230 | 12.27 | 0 | 0 | 0 | 0 | 0 |
| 8 | 230 | 12.28 | 0 | 0 | 0 | 0 | 0 |
| 8 | 230 | 4.2 | 0 | 0 | 0 | 1 | 0 |
| 8 | 230 | 12.29 | 0 | 0 | 0 | 0 | 0 |
| 8 | 230 | 12.3 | 0 | 0 | 0 | 0 | 0 |

| | | | | | | | |
|---|---|---|---|---|---|---|---|
| 8 | 230 | 12.31 | 0 | 0 | 1 | 0 | 0 |
| 8 | 230 | 12.32 | 0 | 1 | 0 | 0 | 0 |
| 8 | 230 | 12.33 | 0 | 1 | 0 | 0 | 0 |
| 8 | 230 | 12.34 | 0 | 1 | 0 | 0 | 0 |
| 8 | 230 | 12.35 | 0 | 0 | 1 | 0 | 0 |
| 8 | 230 | 12.36 | 0 | 0 | 0 | 0 | 0 |
| 8 | 230 | 12.37 | 0 | 0 | 0 | 0 | 0 |
| 8 | 230 | 12.38 | 0 | 0 | 0 | 0 | 0 |
| 8 | 230 | 4.21 | 0 | 0 | 1 | 1 | 0 |
| 8 | 230 | 12.39 | 0 | 0 | 1 | 0 | 0 |
| 8 | 230 | 12.4 | 0 | 0 | 0 | 0 | 0 |
| 8 | 230 | 12.41 | 0 | 0 | 1 | 0 | 0 |
| 8 | 230 | 12.42 | 0 | 0 | 0 | 0 | 0 |
| 8 | 230 | 12.43 | 0 | 0 | 0 | 0 | 0 |
| 8 | 230 | 12.44 | 0 | 0 | 0 | 0 | 0 |
| 8 | 230 | 12.45 | 0 | 0 | 0 | 0 | 0 |
| 8 | 230 | 12.46 | 0 | 0 | 0 | 0 | 0 |
| 8 | 230 | 12.47 | 0 | 0 | 0 | 0 | 0 |
| 8 | 230 | 12.48 | 0 | 0 | 0 | 0 | 0 |
| 8 | 230 | 4.22 | 0 | 0 | 0 | 1 | 0 |
| 8 | 230 | 12.49 | 0 | 0 | 0 | 0 | 0 |
| 8 | 230 | 12.5 | 0 | 0 | 0 | 0 | 0 |
| 8 | 230 | 12.51 | 0 | 0 | 0 | 0 | 0 |
| 8 | 230 | 12.52 | 0 | 0 | 0 | 0 | 0 |
| 8 | 230 | 12.53 | 0 | 0 | 0 | 0 | 0 |
| 8 | 230 | 12.54 | 0 | 0 | 0 | 0 | 0 |
| 8 | 230 | 12.55 | 0 | 0 | 0 | 0 | 0 |
| 8 | 230 | 12.56 | 0 | 0 | 1 | 0 | 0 |
| 8 | 230 | 12.57 | 0 | 0 | 0 | 0 | 0 |
| 8 | 230 | 12.58 | 0 | 0 | 0 | 0 | 0 |
| 8 | 230 | 4.23 | 0 | 0 | 0 | 1 | 0 |
| 8 | 230 | 12.59 | 0 | 0 | 0 | 0 | 0 |
| 8 | 230 | 12.6 | 0 | 0 | 0 | 0 | 0 |
| 8 | 230 | 12.61 | 0 | 0 | 1 | 0 | 0 |
| 8 | 230 | 12.62 | 0 | 0 | 1 | 0 | 0 |
| 8 | 230 | 12.63 | 0 | 0 | 0 | 0 | 0 |
| 8 | 230 | 12.64 | 0 | 0 | 1 | 0 | 0 |
| 8 | 230 | 12.65 | 0 | 0 | 0 | 0 | 0 |
| 8 | 230 | 12.66 | 0 | 0 | 0 | 0 | 0 |
| 8 | 230 | 12.67 | 0 | 0 | 0 | 0 | 0 |
| 8 | 230 | 12.68 | 0 | 0 | 0 | 0 | 0 |
| 8 | 230 | 4.24 | 0 | 0 | 0 | 1 | 0 |
| 8 | 230 | 12.69 | 0 | 0 | 0 | 0 | 0 |
| 8 | 230 | 12.7 | 0 | 0 | 0 | 0 | 0 |

| | | | | | | | |
|---|---|---|---|---|---|---|---|
| 8 | 230 | 12.71 | 0 | 0 | 0 | 0 | 0 |
| 8 | 230 | 12.72 | 0 | 0 | 0 | 0 | 0 |
| 8 | 230 | 12.73 | 0 | 0 | 0 | 0 | 0 |
| 8 | 230 | 12.74 | 0 | 0 | 0 | 0 | 0 |
| 8 | 230 | 12.75 | 0 | 0 | 0 | 0 | 0 |
| 8 | 230 | 12.76 | 0 | 0 | 0 | 0 | 0 |
| 8 | 230 | 12.77 | 0 | 0 | 0 | 0 | 0 |
| 8 | 230 | 12.78 | 0 | 0 | 0 | 0 | 0 |
| 8 | 230 | 4.25 | 0 | 0 | 0 | 1 | 0 |
| 8 | 230 | 12.79 | 0 | 0 | 0 | 0 | 0 |
| 8 | 230 | 12.8 | 0 | 0 | 0 | 0 | 0 |
| 8 | 230 | 12.81 | 0 | 0 | 0 | 0 | 0 |
| 8 | 230 | 12.82 | 0 | 0 | 0 | 0 | 0 |
| 8 | 230 | 12.83 | 0 | 0 | 0 | 0 | 0 |
| 8 | 230 | 12.84 | 0 | 0 | 0 | 0 | 0 |
| 8 | 230 | 12.85 | 0 | 0 | 0 | 0 | 0 |
| 8 | 230 | 12.86 | 0 | 0 | 0 | 0 | 0 |
| 8 | 230 | 12.87 | 0 | 0 | 0 | 0 | 0 |
| 8 | 230 | 12.88 | 0 | 0 | 0 | 0 | 0 |
| 8 | 230 | 4.26 | 0 | 0 | 0 | 1 | 0 |
| 8 | 230 | 12.89 | 0 | 0 | 0 | 0 | 0 |
| 8 | 230 | 11.2 | 0 | 0 | 0 | 0 | 0 |
| 8 | 230 | 11.21 | 0 | 0 | 0 | 0 | 0 |
| 8 | 230 | 11.22 | 0 | 0 | 1 | 0 | 0 |
| 8 | 230 | 11.23 | 0 | 0 | 1 | 0 | 0 |
| 8 | 230 | 11.24 | 0 | 0 | 1 | 0 | 0 |
| 8 | 230 | 11.25 | 0 | 0 | 0 | 0 | 0 |
| 8 | 230 | 11.26 | 0 | 0 | 1 | 0 | 0 |
| 8 | 230 | 11.27 | 0 | 0 | 0 | 0 | 0 |
| 8 | 230 | 11.28 | 0 | 0 | 0 | 0 | 0 |
| 8 | 230 | 4.27 | 0 | 0 | 0 | 1 | 0 |
| 8 | 230 | 11.29 | 0 | 0 | 0 | 0 | 0 |
| 8 | 230 | 11.3 | 0 | 0 | 0 | 0 | 0 |
| 8 | 230 | 11.31 | 0 | 0 | 0 | 0 | 0 |
| 8 | 230 | 11.32 | 0 | 0 | 0 | 0 | 0 |
| 8 | 230 | 11.33 | 0 | 0 | 0 | 0 | 0 |
| 8 | 230 | 11.34 | 0 | 0 | 0 | 0 | 0 |
| 8 | 230 | 11.35 | 0 | 0 | 0 | 0 | 0 |
| 8 | 230 | 11.36 | 0 | 0 | 0 | 0 | 0 |
| 8 | 230 | 11.37 | 0 | 0 | 0 | 0 | 0 |
| 8 | 230 | 11.38 | 0 | 0 | 1 | 0 | 0 |
| 8 | 230 | 4.28 | 0 | 0 | 0 | 1 | 0 |
| 8 | 230 | 11.39 | 0 | 0 | 0 | 0 | 0 |
| 8 | 230 | 11.4 | 0 | 0 | 0 | 0 | 0 |

| | | | | | | | |
|---|---|---|---|---|---|---|---|
| 8 | 230 | 11.41 | 0 | 0 | 0 | 0 | 0 |
| 8 | 230 | 11.42 | 0 | 0 | 0 | 0 | 0 |
| 8 | 230 | 11.43 | 0 | 0 | 0 | 0 | 0 |
| 8 | 230 | 11.44 | 0 | 0 | 1 | 0 | 0 |
| 8 | 230 | 12.9 | 0 | 0 | 1 | 0 | 0 |
| 8 | 230 | 12.91 | 0 | 0 | 0 | 0 | 0 |
| 8 | 230 | 12.92 | 0 | 0 | 0 | 0 | 0 |
| 8 | 230 | 12.93 | 0 | 0 | 0 | 0 | 0 |
| 8 | 230 | 4.29 | 0 | 0 | 0 | 1 | 0 |
| 8 | 230 | 12.94 | 0 | 0 | 0 | 0 | 0 |
| 8 | 230 | 11.45 | 0 | 0 | 0 | 0 | 0 |
| 8 | 230 | 11.46 | 0 | 0 | 0 | 0 | 0 |
| 8 | 230 | 11.47 | 0 | 0 | 0 | 0 | 0 |
| 8 | 230 | 11.48 | 0 | 0 | 0 | 0 | 0 |
| 8 | 230 | 11.49 | 0 | 0 | 0 | 0 | 0 |
| 8 | 230 | 11.5 | 0 | 0 | 0 | 0 | 0 |
| 8 | 230 | 11.51 | 0 | 0 | 0 | 0 | 0 |
| 7 | 230 | 11.52 | 0 | 0 | 0 | 0 | 0 |
| 7 | 230 | 11.53 | 0 | 0 | 0 | 0 | 0 |
| 7 | 230 | 4.3 | 0 | 0 | 0 | 1 | 0 |
| 7 | 230 | 11.54 | 0 | 0 | 0 | 0 | 0 |
| 7 | 230 | 11.55 | 0 | 0 | 0 | 0 | 0 |
| 7 | 230 | 4.31 | 0 | 0 | 1 | 1 | 0 |
| 7 | 230 | 4.32 | 0 | 0 | 0 | 1 | 0 |
| 7 | 230 | 11.65 | 0 | 0 | 0 | 0 | 0 |
| 7 | 230 | 11.66 | 0 | 0 | 0 | 0 | 0 |
| 7 | 230 | 11.67 | 0 | 0 | 0 | 0 | 0 |
| 7 | 230 | 11.68 | 0 | 0 | 0 | 0 | 0 |
| 7 | 230 | 11.69 | 0 | 0 | 0 | 0 | 0 |
| 7 | 230 | 11.7 | 0 | 1 | 0 | 0 | 0 |
| 7 | 230 | 11.71 | 0 | 0 | 0 | 0 | 0 |
| 7 | 230 | 11.72 | 0 | 0 | 0 | 0 | 0 |
| 7 | 230 | 4.33 | 0 | 0 | 0 | 1 | 0 |
| 7 | 230 | 11.73 | 0 | 0 | 0 | 0 | 0 |
| 7 | 230 | 11.74 | 0 | 0 | 0 | 0 | 0 |
| 7 | 230 | 11.75 | 0 | 0 | 1 | 0 | 0 |
| 7 | 230 | 11.76 | 0 | 0 | 0 | 0 | 0 |
| 7 | 230 | 11.78 | 0 | 0 | 0 | 0 | 0 |
| 7 | 230 | 11.79 | 0 | 0 | 0 | 0 | 0 |
| 7 | 230 | 11.8 | 0 | 0 | 0 | 0 | 0 |
| 7 | 230 | 11.81 | 0 | 0 | 0 | 0 | 0 |
| 7 | 230 | 11.82 | 0 | 0 | 0 | 0 | 0 |
| 7 | 230 | 4.34 | 0 | 0 | 0 | 1 | 0 |
| 7 | 230 | 11.83 | 0 | 0 | 0 | 0 | 0 |

| | | | | | | | |
|---|---|---|---|---|---|---|---|
| 7 | 230 | 11.84 | 0 | 0 | 0 | 0 | 0 |
| 7 | 230 | 11.85 | 0 | 0 | 0 | 0 | 0 |
| 7 | 230 | 11.86 | 0 | 0 | 1 | 0 | 0 |
| 7 | 230 | 11.87 | 0 | 0 | 1 | 0 | 0 |
| 7 | 230 | 11.88 | 0 | 0 | 1 | 0 | 0 |
| 7 | 230 | 11.89 | 0 | 0 | 0 | 0 | 0 |
| 7 | 230 | 11.9 | 0 | 0 | 0 | 0 | 0 |
| 7 | 230 | 11.91 | 0 | 0 | 0 | 0 | 0 |
| 7 | 230 | 11.92 | 0 | 0 | 0 | 0 | 0 |
| 7 | 230 | 4.35 | 0 | 0 | 0 | 1 | 0 |
| 7 | 230 | 11.93 | 0 | 0 | 0 | 0 | 0 |
| 7 | 230 | 11.94 | 0 | 0 | 0 | 0 | 0 |
| 7 | 230 | 11.95 | 0 | 0 | 0 | 0 | 0 |
| 7 | 230 | 11.96 | 0 | 0 | 0 | 0 | 0 |
| 7 | 230 | 10.24 | 0 | 0 | 0 | 0 | 0 |
| 7 | 230 | 10.25 | 0 | 0 | 0 | 0 | 0 |
| 7 | 230 | 10.26 | 0 | 0 | 0 | 0 | 0 |
| 7 | 230 | 10.27 | 0 | 0 | 0 | 0 | 0 |
| 7 | 230 | 10.28 | 0 | 0 | 0 | 0 | 0 |
| 7 | 230 | 10.29 | 0 | 0 | 0 | 0 | 0 |
| 7 | 230 | 4.36 | 0 | 0 | 1 | 1 | 0 |
| 7 | 230 | 10.3 | 0 | 0 | 0 | 0 | 0 |
| 7 | 230 | 10.31 | 0 | 0 | 0 | 0 | 0 |
| 7 | 230 | 10.32 | 0 | 0 | 0 | 0 | 0 |
| 7 | 230 | 10.33 | 0 | 0 | 0 | 0 | 0 |
| 7 | 230 | 10.34 | 0 | 0 | 0 | 0 | 0 |
| 7 | 230 | 10.35 | 0 | 0 | 1 | 0 | 0 |
| 7 | 230 | 14.62 | 0 | 0 | 0 | 0 | 0 |
| 7 | 230 | 14.63 | 0 | 0 | 1 | 0 | 0 |
| 7 | 230 | 14.64 | 0 | 1 | 0 | 0 | 0 |
| 7 | 230 | 14.65 | 0 | 1 | 0 | 0 | 0 |
| 7 | 230 | 4.37 | 0 | 0 | 0 | 1 | 0 |
| 7 | 230 | 14.66 | 0 | 0 | 0 | 0 | 0 |
| 7 | 230 | 14.67 | 0 | 0 | 0 | 0 | 0 |
| 7 | 230 | 14.68 | 0 | 0 | 0 | 0 | 0 |
| 7 | 230 | 14.69 | 0 | 1 | 0 | 0 | 0 |
| 7 | 230 | 14.7 | 0 | 1 | 0 | 0 | 0 |
| 7 | 230 | 14.71 | 0 | 1 | 0 | 0 | 0 |
| 7 | 230 | 14.72 | 0 | 1 | 0 | 0 | 0 |
| 7 | 230 | 14.73 | 0 | 1 | 0 | 0 | 0 |
| 7 | 230 | 14.74 | 0 | 1 | 0 | 0 | 0 |
| 7 | 230 | 14.75 | 0 | 1 | 0 | 0 | 0 |
| 7 | 230 | 4.38 | 0 | 0 | 0 | 1 | 0 |
| 7 | 230 | 14.76 | 0 | 1 | 0 | 0 | 0 |

| | | | | | | | |
|---|---|---|---|---|---|---|---|
| 7 | 230 | 14.77 | 0 | 1 | 0 | 0 | 0 |
| 7 | 230 | 14.74 | 0 | 0 | 0 | 0 | 0 |
| 7 | 230 | 14.75 | 0 | 0 | 0 | 0 | 0 |
| 7 | 230 | 14.76 | 0 | 0 | 0 | 0 | 0 |
| 7 | 230 | 14.77 | 0 | 0 | 0 | 0 | 0 |
| 7 | 230 | 14.78 | 0 | 0 | 0 | 0 | 0 |
| 7 | 230 | 14.79 | 0 | 0 | 0 | 0 | 0 |
| 7 | 230 | 14.8 | 0 | 0 | 0 | 0 | 0 |
| 7 | 230 | 14.81 | 0 | 0 | 0 | 0 | 0 |
| 7 | 230 | 4.39 | 0 | 0 | 0 | 1 | 0 |
| 7 | 230 | 14.82 | 0 | 0 | 0 | 0 | 0 |
| 7 | 230 | 14.83 | 0 | 0 | 0 | 0 | 0 |
| 7 | 230 | 4.92 | 0 | 0 | 0 | 1 | 0 |
| 7 | 230 | 4.93 | 0 | 0 | 0 | 1 | 0 |
| 7 | 230 | 4.94 | 0 | 0 | 0 | 1 | 0 |
| 7 | 230 | 4.95 | 0 | 0 | 0 | 1 | 0 |
| 7 | 230 | 4.96 | 0 | 0 | 0 | 1 | 0 |
| 7 | 230 | 4.97 | 0 | 0 | 0 | 1 | 0 |
| 7 | 230 | 4.98 | 0 | 0 | 0 | 1 | 0 |
| 7 | 230 | 4.99 | 0 | 0 | 0 | 1 | 0 |
| 7 | 230 | 4.4 | 0 | 0 | 1 | 1 | 0 |
| 7 | 230 | 5 | 0 | 0 | 0 | 1 | 1 |
| 7 | 230 | 1.74 | 0 | 0 | 0 | 1 | 1 |
| 7 | 230 | 24.01 | 0 | 1 | 0 | 0 | 1 |
| 7 | 230 | 5.01 | 0 | 0 | 0 | 1 | 1 |
| 7 | 230 | 5.02 | 0 | 0 | 0 | 1 | 1 |
| 7 | 230 | 5.03 | 0 | 0 | 0 | 1 | 1 |
| 7 | 230 | 5.04 | 0 | 0 | 0 | 1 | 1 |
| 7 | 230 | 4.08 | 0 | 0 | 0 | 1 | 1 |
| 7 | 230 | 4.09 | 0 | 0 | 0 | 1 | 1 |
| 7 | 230 | 4.1 | 0 | 0 | 0 | 1 | 1 |
| 7 | 230 | 4.11 | 0 | 0 | 0 | 1 | 1 |
| 7 | 230 | 1.75 | 0 | 0 | 1 | 1 | 1 |
| 7 | 230 | 4.12 | 0 | 0 | 0 | 1 | 1 |
| 7 | 230 | 4.41 | 0 | 0 | 1 | 1 | 1 |
| 7 | 230 | 4.13 | 0 | 0 | 0 | 1 | 1 |
| 7 | 230 | 1.41 | 0 | 0 | 0 | 1 | 1 |
| 7 | 230 | 4.14 | 0 | 0 | 0 | 1 | 1 |
| 7 | 230 | 4.15 | 0 | 0 | 0 | 1 | 1 |
| 7 | 230 | 4.16 | 0 | 0 | 0 | 1 | 1 |
| 7 | 230 | 4.17 | 0 | 0 | 0 | 1 | 1 |
| 7 | 230 | 1.42 | 0 | 0 | 0 | 1 | 1 |
| 7 | 230 | 4.18 | 0 | 0 | 0 | 1 | 1 |
| 7 | 230 | 4.19 | 0 | 0 | 0 | 1 | 1 |

| | | | | | | | |
|---|---|---|---|---|---|---|---|
| 7 | 230 | 4.2 | 0 | 0 | 1 | 1 | 1 |
| 7 | 230 | 4.21 | 0 | 0 | 1 | 1 | 1 |
| 7 | 230 | 1.43 | 0 | 0 | 0 | 1 | 1 |
| 7 | 230 | 4.22 | 0 | 0 | 0 | 1 | 1 |
| 7 | 230 | 4.42 | 0 | 0 | 1 | 1 | 1 |
| 7 | 230 | 4.23 | 0 | 0 | 1 | 1 | 1 |
| 7 | 230 | 4.24 | 0 | 0 | 0 | 1 | 1 |
| 7 | 230 | 4.25 | 0 | 0 | 0 | 1 | 1 |
| 7 | 230 | 10.07 | 0 | 0 | 0 | 1 | 1 |
| 7 | 230 | 4.26 | 0 | 0 | 0 | 1 | 1 |
| 7 | 230 | 4.27 | 0 | 0 | 0 | 1 | 1 |
| 7 | 230 | 4.28 | 0 | 0 | 1 | 1 | 1 |
| 7 | 230 | 4.29 | 0 | 0 | 0 | 1 | 1 |
| 7 | 230 | 10.06 | 0 | 0 | 0 | 1 | 1 |
| 7 | 230 | 4.3 | 0 | 0 | 0 | 1 | 1 |
| 7 | 230 | 4.31 | 0 | 0 | 1 | 1 | 1 |
| 7 | 230 | 4.32 | 0 | 0 | 0 | 1 | 1 |
| 7 | 230 | 4.43 | 0 | 0 | 1 | 1 | 1 |
| 7 | 230 | 4.33 | 0 | 0 | 1 | 1 | 1 |
| 7 | 230 | 10.05 | 0 | 0 | 0 | 1 | 1 |
| 7 | 230 | 4.34 | 0 | 0 | 1 | 1 | 1 |
| 7 | 230 | 4.35 | 0 | 0 | 0 | 1 | 1 |
| 7 | 230 | 4.36 | 0 | 0 | 0 | 1 | 1 |
| 7 | 230 | 4.37 | 0 | 0 | 1 | 1 | 1 |
| 7 | 230 | 10.04 | 0 | 0 | 1 | 1 | 1 |
| 7 | 230 | 4.38 | 0 | 0 | 0 | 1 | 1 |
| 7 | 230 | 4.39 | 0 | 0 | 0 | 1 | 1 |
| 7 | 230 | 8.6 | 0 | 0 | 1 | 0 | 1 |
| 7 | 230 | 8.61 | 0 | 0 | 0 | 0 | 1 |
| 7 | 230 | 10.03 | 0 | 0 | 0 | 1 | 1 |
| 7 | 230 | 8.62 | 0 | 0 | 0 | 0 | 1 |
| 7 | 230 | 4.44 | 0 | 0 | 0 | 1 | 1 |
| 7 | 230 | 8.63 | 0 | 0 | 0 | 0 | 1 |
| 7 | 230 | 8.64 | 0 | 0 | 0 | 0 | 1 |
| 7 | 230 | 8.65 | 0 | 0 | 1 | 0 | 1 |
| 7 | 230 | 10.02 | 0 | 0 | 0 | 1 | 1 |
| 7 | 230 | 8.66 | 0 | 0 | 0 | 0 | 1 |
| 7 | 230 | 10.36 | 0 | 0 | 0 | 0 | 1 |
| 7 | 230 | 10.37 | 0 | 0 | 0 | 0 | 1 |
| 7 | 230 | 10.38 | 0 | 0 | 0 | 0 | 1 |
| 7 | 230 | 10.01 | 0 | 0 | 0 | 1 | 1 |
| 7 | 230 | 14.84 | 0 | 0 | 1 | 0 | 1 |
| 7 | 230 | 14.85 | 0 | 0 | 0 | 0 | 1 |
| 7 | 230 | 14.86 | 0 | 0 | 0 | 0 | 1 |

| | | | | | | | |
|---|---|---|---|---|---|---|---|
| 7 | 230 | 4.45 | 0 | 0 | 0 | 1 | 1 |
| 7 | 230 | 4.4 | 0 | 0 | 0 | 1 | 1 |
| 7 | 230 | 10 | 0 | 0 | 0 | 1 | 1 |
| 7 | 230 | 4.41 | 0 | 0 | 0 | 1 | 1 |
| 7 | 230 | 9.99 | 0 | 0 | 0 | 1 | 1 |
| 7 | 230 | 9.98 | 0 | 0 | 0 | 1 | 1 |
| 7 | 235 | 4.46 | 0 | 0 | 0 | 1 | 1 |
| 7 | 235 | 9.97 | 0 | 0 | 0 | 1 | 1 |
| 7 | 235 | 9.96 | 0 | 0 | 0 | 1 | 1 |
| 7 | 235 | 4.47 | 0 | 0 | 1 | 1 | 1 |
| 7 | 235 | 9.95 | 0 | 0 | 0 | 1 | 1 |
| 7 | 235 | 9.94 | 0 | 0 | 1 | 1 | 1 |
| 7 | 235 | 9.93 | 0 | 0 | 0 | 1 | 1 |
| 7 | 235 | 4.48 | 0 | 0 | 0 | 1 | 1 |
| 7 | 235 | 9.11 | 0 | 0 | 0 | 1 | 1 |
| 7 | 235 | 9.1 | 0 | 0 | 0 | 1 | 1 |
| 7 | 235 | 4.49 | 0 | 0 | 0 | 1 | 1 |
| 7 | 235 | 9.09 | 0 | 0 | 0 | 1 | 1 |
| 7 | 235 | 8.67 | 0 | 0 | 0 | 0 | 1 |
| 7 | 235 | 8.68 | 0 | 0 | 1 | 0 | 1 |
| 7 | 235 | 9.08 | 0 | 0 | 1 | 1 | 1 |
| 7 | 235 | 8.69 | 0 | 0 | 1 | 0 | 1 |
| 7 | 235 | 7.44 | 0 | 0 | 0 | 0 | 1 |
| 7 | 235 | 7.45 | 0 | 0 | 0 | 0 | 1 |
| 7 | 235 | 7.46 | 0 | 0 | 0 | 0 | 1 |
| 7 | 235 | 9.07 | 0 | 0 | 0 | 1 | 1 |
| 7 | 235 | 7.47 | 0 | 0 | 1 | 0 | 1 |
| 7 | 235 | 7.48 | 0 | 0 | 0 | 0 | 1 |
| 7 | 235 | 4.5 | 0 | 0 | 0 | 1 | 1 |
| 7 | 235 | 7.49 | 0 | 0 | 0 | 0 | 1 |
| 7 | 235 | 7.5 | 0 | 0 | 0 | 0 | 1 |
| 7 | 235 | 9.06 | 0 | 0 | 0 | 1 | 1 |
| 7 | 240 | 7.51 | 0 | 0 | 0 | 0 | 1 |
| 7 | 240 | 7.52 | 0 | 0 | 0 | 0 | 1 |
| 7 | 240 | 7.53 | 0 | 0 | 0 | 0 | 1 |
| 7 | 240 | 7.54 | 0 | 0 | 0 | 0 | 1 |
| 7 | 240 | 9.05 | 0 | 0 | 0 | 1 | 1 |
| 7 | 240 | 7.55 | 0 | 0 | 0 | 0 | 1 |
| 7 | 240 | 7.56 | 0 | 1 | 1 | 1 | 1 |
| 7 | 240 | 7.57 | 0 | 1 | 1 | 1 | 1 |
| 7 | 240 | 7.58 | 0 | 0 | 0 | 1 | 1 |
| 7 | 240 | 4.51 | 0 | 0 | 0 | 1 | 1 |
| 7 | 240 | 9.04 | 0 | 0 | 1 | 1 | 1 |
| 7 | 240 | 7.59 | 0 | 0 | 0 | 1 | 1 |

| | | | | | | | |
|---|---|---|---|---|---|---|---|
| 7 | 240 | 7.6 | 0 | 0 | 1 | 1 | 1 |
| 7 | 240 | 7.61 | 0 | 0 | 1 | 1 | 1 |
| 7 | 240 | 7.62 | 0 | 0 | 1 | 1 | 1 |
| 7 | 240 | 9.03 | 0 | 0 | 1 | 1 | 1 |
| 7 | 240 | 7.63 | 0 | 0 | 0 | 1 | 1 |
| 7 | 240 | 7.64 | 0 | 0 | 0 | 1 | 1 |
| 7 | 240 | 7.65 | 0 | 0 | 1 | 1 | 1 |
| 7 | 240 | 7.66 | 0 | 0 | 0 | 1 | 1 |
| 7 | 240 | 9.02 | 0 | 0 | 1 | 1 | 1 |
| 7 | 240 | 7.67 | 0 | 0 | 0 | 1 | 1 |
| 7 | 240 | 7.68 | 0 | 0 | 0 | 1 | 1 |
| 7 | 240 | 4.52 | 0 | 0 | 0 | 1 | 1 |
| 7 | 240 | 7.69 | 0 | 0 | 0 | 1 | 1 |
| 7 | 240 | 7.7 | 0 | 0 | 1 | 1 | 1 |
| 7 | 240 | 9.01 | 0 | 0 | 0 | 1 | 1 |
| 7 | 240 | 7.71 | 0 | 0 | 0 | 1 | 1 |
| 7 | 240 | 7.72 | 0 | 0 | 0 | 1 | 1 |
| 7 | 240 | 7.73 | 0 | 0 | 0 | 1 | 1 |
| 7 | 240 | 7.74 | 0 | 1 | 0 | 1 | 1 |
| 7 | 240 | 9 | 0 | 0 | 1 | 1 | 1 |
| 7 | 240 | 7.75 | 0 | 0 | 0 | 1 | 1 |
| 7 | 240 | 7.76 | 0 | 0 | 1 | 1 | 1 |
| 7 | 240 | 7.77 | 0 | 0 | 1 | 1 | 1 |
| 7 | 240 | 7.78 | 0 | 0 | 1 | 1 | 1 |
| 7 | 240 | 4.53 | 0 | 0 | 1 | 1 | 1 |
| 7 | 240 | 8.99 | 0 | 0 | 0 | 1 | 1 |
| 7 | 240 | 7.79 | 0 | 0 | 0 | 1 | 1 |
| 7 | 240 | 7.8 | 0 | 0 | 0 | 1 | 1 |
| 7 | 240 | 7.81 | 0 | 0 | 0 | 1 | 1 |
| 7 | 240 | 6.6 | 0 | 0 | 0 | 1 | 1 |
| 7 | 240 | 8.98 | 0 | 0 | 0 | 1 | 1 |
| 7 | 240 | 6.61 | 0 | 0 | 0 | 1 | 1 |
| 7 | 240 | 6.62 | 0 | 0 | 0 | 1 | 1 |
| 7 | 240 | 6.63 | 0 | 0 | 0 | 1 | 1 |
| 7 | 240 | 8.27 | 0 | 0 | 0 | 1 | 1 |
| 7 | 240 | 6.64 | 0 | 0 | 0 | 1 | 1 |
| 7 | 240 | 6.65 | 0 | 0 | 0 | 1 | 1 |
| 7 | 240 | 6.66 | 0 | 0 | 0 | 1 | 1 |
| 7 | 240 | 4.54 | 0 | 0 | 0 | 1 | 1 |
| 7 | 240 | 6.67 | 0 | 0 | 0 | 1 | 1 |
| 7 | 240 | 8.26 | 0 | 0 | 0 | 1 | 1 |
| 7 | 240 | 6.68 | 0 | 0 | 0 | 1 | 1 |
| 7 | 240 | 6.69 | 0 | 0 | 0 | 1 | 1 |
| 7 | 245 | 6.7 | 0 | 0 | 0 | 1 | 1 |

| | | | | | | | |
|---|---|---|---|---|---|---|---|
| 7 | 245 | 6.71 | 0 | 0 | 0 | 1 | 1 |
| 7 | 245 | 8.25 | 0 | 0 | 0 | 1 | 1 |
| 7 | 245 | 6.72 | 0 | 0 | 1 | 1 | 1 |
| 7 | 245 | 6.73 | 0 | 0 | 0 | 1 | 1 |
| 7 | 245 | 6.74 | 0 | 0 | 0 | 1 | 1 |
| 7 | 245 | 6.75 | 0 | 0 | 0 | 1 | 1 |
| 7 | 245 | 8.24 | 0 | 0 | 0 | 1 | 1 |
| 7 | 245 | 6.76 | 0 | 0 | 0 | 1 | 1 |
| 7 | 245 | 4.55 | 0 | 0 | 0 | 1 | 1 |
| 7 | 245 | 6.77 | 0 | 0 | 0 | 1 | 1 |
| 7 | 245 | 6.78 | 0 | 0 | 0 | 1 | 1 |
| 7 | 245 | 6.79 | 0 | 0 | 0 | 1 | 1 |
| 7 | 245 | 8.23 | 0 | 0 | 0 | 1 | 1 |
| 7 | 245 | 6.8 | 0 | 0 | 0 | 1 | 1 |
| 7 | 245 | 6.81 | 0 | 0 | 1 | 1 | 1 |
| 7 | 245 | 6.82 | 0 | 0 | 0 | 1 | 1 |
| 7 | 245 | 6.83 | 0 | 0 | 0 | 1 | 1 |
| 7 | 245 | 8.22 | 0 | 0 | 0 | 1 | 1 |
| 7 | 245 | 6.84 | 0 | 0 | 0 | 1 | 1 |
| 7 | 245 | 6.85 | 0 | 0 | 0 | 1 | 1 |
| 7 | 245 | 6.86 | 0 | 0 | 0 | 1 | 1 |
| 7 | 245 | 4.56 | 0 | 0 | 0 | 1 | 1 |
| 7 | 245 | 6.87 | 0 | 0 | 0 | 1 | 1 |
| 7 | 245 | 8.21 | 0 | 0 | 0 | 1 | 1 |
| 7 | 245 | 6.88 | 0 | 0 | 1 | 1 | 1 |
| 7 | 245 | 6.89 | 0 | 0 | 1 | 1 | 1 |
| 7 | 245 | 6.9 | 0 | 0 | 0 | 1 | 1 |
| 7 | 245 | 6.91 | 0 | 0 | 0 | 1 | 1 |
| 7 | 245 | 8.2 | 0 | 0 | 1 | 1 | 1 |
| 7 | 245 | 6.92 | 0 | 0 | 1 | 1 | 1 |
| 7 | 245 | 6.93 | 0 | 0 | 0 | 1 | 1 |
| 7 | 245 | 6.94 | 0 | 0 | 0 | 1 | 1 |
| 7 | 245 | 6.95 | 0 | 0 | 0 | 1 | 1 |
| 7 | 245 | 8.19 | 0 | 0 | 0 | 1 | 1 |
| 7 | 245 | 6.96 | 0 | 0 | 0 | 1 | 1 |
| 7 | 245 | 4.57 | 0 | 0 | 0 | 1 | 1 |
| 7 | 245 | 6.97 | 0 | 0 | 1 | 1 | 1 |
| 7 | 245 | 5.76 | 0 | 0 | 0 | 1 | 1 |
| 7 | 245 | 5.77 | 0 | 0 | 1 | 1 | 1 |
| 7 | 245 | 8.18 | 0 | 0 | 0 | 1 | 1 |
| 7 | 245 | 5.78 | 0 | 0 | 0 | 1 | 1 |
| 7 | 245 | 5.79 | 0 | 0 | 0 | 1 | 1 |
| 7 | 245 | 5.8 | 0 | 0 | 0 | 1 | 1 |
| 7 | 245 | 5.81 | 0 | 0 | 0 | 1 | 1 |

| | | | | | | | |
|---|---|---|---|---|---|---|---|
| 7 | 245 | 8.17 | 0 | 0 | 0 | 1 | 1 |
| 7 | 245 | 10.46 | 0 | 0 | 0 | 0 | 1 |
| 7 | 245 | 10.47 | 0 | 0 | 1 | 0 | 1 |
| 7 | 245 | 10.48 | 0 | 0 | 1 | 0 | 1 |
| 7 | 245 | 4.58 | 0 | 0 | 0 | 1 | 1 |
| 7 | 245 | 10.49 | 0 | 0 | 0 | 0 | 1 |
| 7 | 245 | 8.16 | 0 | 0 | 0 | 1 | 1 |
| 7 | 245 | 10.5 | 0 | 0 | 0 | 0 | 1 |
| 7 | 245 | 10.51 | 0 | 0 | 0 | 0 | 1 |
| 7 | 245 | 10.52 | 0 | 0 | 0 | 0 | 1 |
| 7 | 245 | 10.53 | 0 | 0 | 0 | 0 | 1 |
| 7 | 245 | 8.15 | 0 | 0 | 0 | 1 | 1 |
| 7 | 245 | 10.54 | 0 | 0 | 1 | 0 | 1 |
| 7 | 245 | 10.55 | 0 | 0 | 0 | 0 | 1 |
| 7 | 245 | 10.56 | 0 | 0 | 0 | 0 | 1 |
| 7 | 245 | 10.57 | 0 | 0 | 0 | 0 | 1 |
| 7 | 245 | 8.14 | 0 | 0 | 0 | 1 | 1 |
| 7 | 245 | 10.58 | 0 | 0 | 0 | 0 | 1 |
| 7 | 245 | 4.59 | 0 | 0 | 0 | 1 | 1 |
| 7 | 245 | 10.59 | 0 | 0 | 0 | 0 | 1 |
| 7 | 245 | 10.6 | 0 | 0 | 1 | 0 | 1 |
| 7 | 245 | 10.61 | 0 | 0 | 0 | 0 | 1 |
| 7 | 245 | 7.43 | 0 | 0 | 0 | 1 | 1 |
| 7 | 245 | 10.62 | 0 | 0 | 0 | 0 | 1 |
| 7 | 245 | 10.63 | 0 | 0 | 0 | 0 | 1 |
| 7 | 245 | 10.64 | 0 | 0 | 0 | 0 | 1 |
| 7 | 245 | 10.65 | 0 | 0 | 0 | 0 | 1 |
| 7 | 245 | 7.42 | 0 | 0 | 0 | 1 | 1 |
| 7 | 245 | 10.66 | 0 | 0 | 0 | 0 | 1 |
| 7 | 245 | 10.67 | 0 | 0 | 0 | 0 | 1 |
| 7 | 245 | 10.68 | 0 | 0 | 1 | 0 | 1 |
| 7 | 245 | 4.6 | 0 | 0 | 0 | 1 | 1 |
| 7 | 245 | 10.69 | 0 | 0 | 0 | 0 | 1 |
| 7 | 245 | 7.41 | 0 | 0 | 0 | 1 | 1 |
| 7 | 245 | 10.7 | 0 | 0 | 0 | 0 | 1 |
| 7 | 245 | 10.71 | 0 | 0 | 0 | 0 | 1 |
| 7 | 245 | 10.72 | 0 | 0 | 1 | 0 | 1 |
| 7 | 245 | 10.73 | 0 | 1 | 0 | 0 | 1 |
| 7 | 245 | 7.4 | 0 | 0 | 0 | 1 | 1 |
| 7 | 245 | 10.74 | 0 | 1 | 0 | 0 | 1 |
| 5 | 245 | 10.75 | 0 | 1 | 0 | 0 | 1 |
| 5 | 245 | 10.76 | 0 | 1 | 0 | 0 | 1 |
| 5 | 245 | 10.77 | 0 | 1 | 0 | 0 | 1 |
| 5 | 245 | 9.13 | 0 | 0 | 0 | 0 | 1 |

| | | | | | | | |
|---|---|---|---|---|---|---|---|
| 5 | 245 | 4.61 | 0 | 0 | 0 | 1 | 1 |
| 5 | 245 | 9.14 | 0 | 0 | 0 | 0 | 1 |
| 5 | 245 | 9.16 | 0 | 0 | 0 | 0 | 1 |
| 5 | 245 | 7.38 | 0 | 0 | 0 | 1 | 1 |
| 5 | 245 | 9.17 | 0 | 0 | 0 | 0 | 1 |
| 5 | 245 | 9.18 | 0 | 0 | 0 | 0 | 1 |
| 5 | 245 | 9.19 | 0 | 0 | 0 | 0 | 1 |
| 5 | 245 | 7.37 | 0 | 0 | 0 | 1 | 1 |
| 5 | 245 | 9.22 | 0 | 0 | 0 | 0 | 1 |
| 5 | 245 | 9.23 | 0 | 0 | 0 | 0 | 1 |
| 5 | 245 | 4.62 | 0 | 0 | 0 | 1 | 1 |
| 5 | 245 | 7.36 | 0 | 0 | 0 | 1 | 1 |
| 5 | 245 | 9.24 | 0 | 0 | 0 | 0 | 1 |
| 5 | 245 | 9.27 | 0 | 0 | 0 | 0 | 1 |
| 5 | 245 | 9.29 | 0 | 0 | 0 | 0 | 1 |
| 5 | 245 | 9.3 | 0 | 0 | 0 | 0 | 1 |
| 5 | 245 | 9.31 | 0 | 0 | 0 | 0 | 1 |
| 5 | 245 | 7.34 | 0 | 0 | 0 | 1 | 1 |
| 5 | 245 | 9.32 | 0 | 0 | 1 | 0 | 1 |
| 5 | 245 | 9.33 | 0 | 0 | 0 | 0 | 1 |
| 6 | 245 | 9.36 | 0 | 0 | 1 | 0 | 1 |
| 6 | 245 | 9.39 | 0 | 0 | 0 | 0 | 1 |
| 6 | 245 | 7.32 | 0 | 1 | 0 | 1 | 1 |
| 6 | 245 | 9.4 | 0 | 0 | 0 | 0 | 1 |
| 6 | 245 | 9.41 | 0 | 0 | 0 | 0 | 1 |
| 6 | 245 | 9.43 | 0 | 0 | 0 | 0 | 1 |
| 6 | 245 | 4.64 | 0 | 0 | 0 | 1 | 1 |
| 6 | 245 | 7.31 | 0 | 1 | 0 | 1 | 1 |
| 6 | 245 | 9.44 | 0 | 0 | 1 | 0 | 1 |
| 6 | 245 | 9.46 | 0 | 0 | 0 | 0 | 1 |
| 6 | 245 | 7.3 | 0 | 0 | 0 | 1 | 1 |
| 6 | 245 | 9.5 | 0 | 1 | 0 | 0 | 1 |
| 6 | 245 | 9.51 | 0 | 0 | 0 | 0 | 1 |
| 6 | 245 | 6.54 | 0 | 0 | 0 | 1 | 1 |
| 6 | 245 | 9.52 | 0 | 0 | 0 | 0 | 1 |
| 6 | 245 | 9.55 | 0 | 0 | 0 | 0 | 1 |
| 6 | 245 | 6.53 | 0 | 0 | 0 | 1 | 1 |
| 6 | 245 | 8.7 | 0 | 0 | 0 | 0 | 1 |
| 6 | 245 | 8.71 | 0 | 0 | 0 | 0 | 1 |
| 6 | 245 | 8.72 | 0 | 0 | 0 | 0 | 1 |
| 6 | 245 | 6.52 | 0 | 0 | 0 | 1 | 1 |
| 6 | 245 | 8.73 | 0 | 0 | 0 | 0 | 1 |
| 6 | 245 | 8.74 | 0 | 0 | 1 | 0 | 1 |
| 6 | 245 | 0.36 | 0 | 0 | 1 | 1 | 1 |

| | | | | | | | |
|---|---|---|---|---|---|---|---|
| 6 | 245 | 4.66 | 0 | 0 | 0 | 1 | 1 |
| 6 | 245 | 6.51 | 0 | 0 | 0 | 1 | 1 |
| 6 | 245 | 0.34 | 0 | 0 | 0 | 1 | 1 |
| 6 | 250 | 6.5 | 0 | 0 | 0 | 1 | 1 |
| 6 | 250 | 0.29 | 0 | 0 | 0 | 1 | 1 |
| 6 | 250 | 0.28 | 0 | 0 | 0 | 1 | 1 |
| 6 | 250 | 0.27 | 0 | 0 | 0 | 1 | 1 |
| 6 | 250 | 4.67 | 0 | 0 | 0 | 1 | 1 |
| 6 | 250 | 0.25 | 0 | 0 | 0 | 1 | 1 |
| 6 | 250 | 0.24 | 0 | 0 | 0 | 1 | 1 |
| 6 | 250 | 0.23 | 0 | 0 | 0 | 1 | 1 |
| 6 | 250 | 6.48 | 0 | 0 | 0 | 1 | 1 |
| 6 | 250 | 0.22 | 0 | 0 | 0 | 1 | 1 |
| 6 | 250 | 0.21 | 0 | 0 | 0 | 1 | 1 |
| 6 | 250 | 0.2 | 0 | 0 | 0 | 1 | 1 |
| 6 | 250 | 0.19 | 0 | 0 | 0 | 1 | 1 |
| 6 | 250 | 6.47 | 0 | 0 | 0 | 1 | 1 |
| 6 | 250 | 0.18 | 0 | 0 | 0 | 1 | 1 |
| 6 | 250 | 0.17 | 0 | 0 | 1 | 1 | 1 |
| 6 | 250 | 4.68 | 0 | 0 | 0 | 1 | 1 |
| 6 | 250 | 0.15 | 0 | 0 | 0 | 1 | 1 |
| 6 | 250 | 6.46 | 0 | 0 | 0 | 1 | 1 |
| 6 | 250 | 0.14 | 0 | 0 | 0 | 1 | 1 |
| 6 | 250 | 0.13 | 0 | 0 | 1 | 1 | 1 |
| 6 | 250 | 0.12 | 0 | 0 | 0 | 1 | 1 |
| 6 | 250 | 1.76 | 0 | 0 | 0 | 1 | 1 |
| 6 | 250 | 0.78 | 0 | 0 | 0 | 1 | 1 |
| 6 | 250 | 1.77 | 0 | 0 | 1 | 1 | 1 |
| 6 | 250 | 1.78 | 0 | 0 | 0 | 1 | 1 |
| 6 | 250 | 1.79 | 0 | 0 | 0 | 1 | 1 |
| 6 | 250 | 1.8 | 0 | 0 | 0 | 1 | 1 |
| 6 | 250 | 0.79 | 0 | 0 | 1 | 1 | 1 |
| 6 | 250 | 1.81 | 0 | 0 | 0 | 1 | 1 |
| 6 | 250 | 1.84 | 0 | 0 | 1 | 1 | 1 |
| 6 | 250 | 1.2 | 0 | 0 | 0 | 1 | 1 |
| 6 | 250 | 1.85 | 0 | 0 | 0 | 1 | 1 |
| 6 | 250 | 1.86 | 0 | 0 | 0 | 1 | 1 |
| 6 | 250 | 1.87 | 0 | 0 | 0 | 1 | 1 |
| 6 | 250 | 1.88 | 0 | 0 | 0 | 1 | 1 |
| 6 | 250 | 1.21 | 0 | 0 | 0 | 1 | 1 |
| 6 | 250 | 1.89 | 0 | 0 | 0 | 1 | 1 |
| 6 | 250 | 1.9 | 0 | 0 | 0 | 1 | 1 |
| 6 | 250 | 4.7 | 0 | 0 | 0 | 1 | 1 |
| 6 | 250 | 1.92 | 0 | 0 | 0 | 1 | 1 |

| | | | | | | | |
|---|---|---|---|---|---|---|---|
| 6 | 250 | 1.93 | 0 | 0 | 0 | 1 | 1 |
| 6 | 250 | 1.94 | 0 | 0 | 0 | 1 | 1 |
| 6 | 250 | 1.95 | 0 | 0 | 0 | 1 | 1 |
| 6 | 250 | 1.23 | 0 | 0 | 0 | 1 | 1 |
| 6 | 250 | 1.96 | 0 | 0 | 0 | 1 | 1 |
| 6 | 250 | 1.97 | 0 | 0 | 1 | 1 | 1 |
| 6 | 250 | 1.98 | 0 | 0 | 0 | 1 | 1 |
| 6 | 250 | 1.99 | 0 | 0 | 0 | 1 | 1 |
| 6 | 250 | 1.24 | 0 | 0 | 0 | 1 | 1 |
| 6 | 250 | 2 | 0 | 0 | 0 | 1 | 1 |
| 6 | 250 | 2.01 | 0 | 0 | 0 | 1 | 1 |
| 6 | 250 | 1.93 | 0 | 0 | 0 | 1 | 1 |
| 6 | 250 | 1.25 | 0 | 0 | 0 | 1 | 1 |
| 6 | 250 | 1.95 | 0 | 0 | 1 | 1 | 1 |
| 6 | 250 | 1.96 | 0 | 0 | 0 | 1 | 1 |
| 6 | 250 | 1.97 | 0 | 0 | 0 | 1 | 1 |
| 6 | 250 | 1.26 | 0 | 0 | 0 | 1 | 1 |
| 6 | 250 | 1.98 | 0 | 0 | 0 | 1 | 1 |
| 6 | 250 | 2 | 0 | 0 | 0 | 1 | 1 |
| 6 | 250 | 1.27 | 0 | 0 | 1 | 1 | 1 |
| 6 | 250 | 4.72 | 0 | 0 | 1 | 1 | 1 |
| 6 | 250 | 2.02 | 0 | 0 | 1 | 1 | 1 |
| 6 | 250 | 4.57 | 0 | 0 | 0 | 1 | 1 |
| 6 | 250 | 4.58 | 0 | 0 | 0 | 1 | 1 |
| 6 | 250 | 4.59 | 0 | 0 | 0 | 1 | 1 |
| 6 | 250 | 4.6 | 0 | 0 | 0 | 1 | 1 |
| 6 | 250 | 4.61 | 0 | 0 | 0 | 1 | 1 |
| 6 | 250 | 4.62 | 0 | 0 | 0 | 1 | 1 |
| 6 | 250 | 4.63 | 0 | 0 | 0 | 1 | 1 |
| 6 | 250 | 4.64 | 0 | 0 | 0 | 1 | 1 |
| 6 | 250 | 4.65 | 0 | 0 | 0 | 1 | 1 |
| 6 | 250 | 9.03 | 0 | 0 | 0 | 1 | 1 |
| 6 | 250 | 4.73 | 0 | 0 | 0 | 1 | 1 |
| 6 | 250 | 9.04 | 0 | 0 | 1 | 1 | 1 |
| 6 | 250 | 9.05 | 0 | 0 | 0 | 1 | 1 |
| 6 | 250 | 9.06 | 0 | 0 | 1 | 1 | 1 |
| 6 | 250 | 9.07 | 0 | 0 | 0 | 1 | 1 |
| 6 | 250 | 9.08 | 0 | 0 | 0 | 1 | 1 |
| 6 | 250 | 8.69 | 0 | 0 | 0 | 1 | 1 |
| 6 | 250 | 8.7 | 0 | 0 | 0 | 1 | 1 |
| 6 | 250 | 8.71 | 0 | 0 | 0 | 1 | 1 |
| 6 | 250 | 4.74 | 0 | 0 | 1 | 1 | 1 |
| 6 | 250 | 8.74 | 0 | 0 | 0 | 1 | 1 |
| 6 | 250 | 8.76 | 0 | 0 | 1 | 1 | 1 |

| | | | | | | | |
|---|---|---|---|---|---|---|---|
| 6 | 250 | 8.77 | 0 | 0 | 0 | 1 | 1 |
| 6 | 250 | 8.78 | 0 | 0 | 1 | 1 | 1 |
| 6 | 254 | 8.79 | 0 | 0 | 0 | 1 | 1 |
| 6 | 254 | 8.8 | 0 | 0 | 0 | 1 | 1 |
| 6 | 254 | 8.82 | 0 | 0 | 1 | 1 | 1 |
| 6 | 254 | 8.83 | 0 | 0 | 0 | 1 | 1 |
| 6 | 254 | 4.75 | 0 | 0 | 0 | 1 | 1 |
| 6 | 254 | 8.85 | 0 | 0 | 0 | 1 | 1 |
| 6 | 254 | 8.86 | 0 | 0 | 0 | 1 | 1 |
| 6 | 254 | 8.87 | 0 | 0 | 0 | 1 | 1 |
| 6 | 254 | 8.88 | 0 | 0 | 1 | 1 | 1 |
| 6 | 254 | 8.89 | 0 | 0 | 1 | 1 | 1 |
| 6 | 254 | 8.9 | 0 | 0 | 1 | 1 | 1 |
| 6 | 254 | 8.91 | 0 | 0 | 0 | 1 | 1 |
| 6 | 254 | 8.92 | 0 | 0 | 0 | 1 | 1 |
| 6 | 254 | 8.93 | 0 | 0 | 1 | 1 | 1 |
| 6 | 254 | 4.76 | 0 | 0 | 1 | 1 | 1 |
| 6 | 254 | 8.94 | 0 | 0 | 1 | 1 | 1 |
| 6 | 254 | 8.95 | 0 | 0 | 0 | 1 | 1 |
| 6 | 254 | 8.96 | 0 | 0 | 0 | 1 | 1 |
| 6 | 254 | 3.69 | 0 | 0 | 1 | 1 | 1 |
| 6 | 254 | 3.7 | 0 | 0 | 0 | 1 | 1 |
| 6 | 254 | 3.71 | 0 | 0 | 1 | 1 | 1 |
| 6 | 254 | 3.73 | 0 | 0 | 0 | 1 | 1 |
| 6 | 254 | 4.77 | 0 | 0 | 0 | 1 | 1 |
| 6 | 254 | 3.74 | 0 | 0 | 0 | 1 | 1 |
| 6 | 254 | 3.75 | 0 | 0 | 0 | 1 | 1 |
| 6 | 254 | 3.78 | 0 | 0 | 0 | 1 | 1 |
| 6 | 254 | 3.8 | 0 | 0 | 1 | 1 | 1 |
| 6 | 254 | 3.81 | 0 | 0 | 0 | 1 | 1 |
| 6 | 254 | 3.82 | 0 | 0 | 1 | 1 | 1 |
| 6 | 254 | 3.83 | 0 | 0 | 0 | 1 | 1 |
| 6 | 254 | 3.84 | 0 | 0 | 0 | 1 | 1 |
| 6 | 254 | 3.85 | 0 | 0 | 1 | 1 | 1 |
| 6 | 254 | 7.74 | 0 | 0 | 0 | 1 | 1 |
| 6 | 254 | 7.75 | 0 | 1 | 0 | 1 | 1 |
| 6 | 254 | 7.76 | 0 | 0 | 0 | 1 | 1 |
| 6 | 254 | 7.77 | 0 | 0 | 0 | 1 | 1 |
| 6 | 254 | 7.78 | 0 | 0 | 0 | 1 | 1 |
| 6 | 254 | 7.79 | 0 | 0 | 1 | 1 | 1 |
| 6 | 254 | 7.81 | 0 | 0 | 0 | 1 | 1 |
| 6 | 254 | 7.83 | 0 | 0 | 0 | 1 | 1 |
| 6 | 254 | 7.89 | 0 | 0 | 0 | 1 | 1 |
| 6 | 254 | 7.9 | 0 | 0 | 0 | 1 | 1 |

| | | | | | | | |
|---|---|---|---|---|---|---|---|
| 6 | 254 | 7.91 | 0 | 0 | 0 | 1 | 1 |
| 6 | 254 | 4.8 | 0 | 0 | 0 | 1 | 1 |
| 6 | 254 | 7.92 | 0 | 0 | 0 | 1 | 1 |
| 6 | 254 | 7.94 | 0 | 0 | 1 | 1 | 1 |
| 6 | 254 | 7.95 | 0 | 0 | 0 | 1 | 1 |
| 6 | 254 | 7.97 | 0 | 0 | 0 | 1 | 1 |
| 6 | 254 | 7.99 | 0 | 0 | 1 | 1 | 1 |
| 6 | 254 | 8.09 | 0 | 0 | 0 | 1 | 1 |
| 6 | 254 | 8.13 | 0 | 0 | 1 | 1 | 1 |
| 6 | 254 | 8.19 | 0 | 0 | 0 | 1 | 1 |
| 6 | 254 | 8.2 | 0 | 0 | 0 | 1 | 1 |
| 6 | 254 | 8.21 | 0 | 0 | 1 | 1 | 1 |
| 6 | 254 | 4.83 | 0 | 0 | 0 | 1 | 1 |
| 6 | 254 | 8.24 | 0 | 0 | 1 | 1 | 1 |
| 6 | 254 | 8.25 | 0 | 0 | 0 | 1 | 1 |
| 6 | 254 | 8.26 | 0 | 0 | 0 | 1 | 1 |
| 6 | 254 | 8.27 | 0 | 0 | 0 | 1 | 1 |
| 6 | 254 | 8.28 | 0 | 0 | 1 | 1 | 1 |
| 6 | 254 | 8.29 | 0 | 0 | 1 | 1 | 1 |
| 6 | 254 | 8.3 | 0 | 0 | 0 | 1 | 1 |
| 6 | 254 | 4.84 | 0 | 0 | 1 | 1 | 1 |
| 6 | 254 | 8.32 | 0 | 0 | 1 | 1 | 1 |
| 6 | 254 | 8.36 | 0 | 0 | 1 | 1 | 1 |
| 6 | 254 | 5.84 | 0 | 0 | 1 | 1 | 1 |
| 6 | 254 | 5.85 | 0 | 0 | 1 | 1 | 1 |
| 6 | 254 | 5.86 | 0 | 0 | 1 | 1 | 1 |
| 6 | 254 | 4.85 | 0 | 0 | 1 | 1 | 1 |
| 6 | 254 | 5.89 | 0 | 0 | 0 | 1 | 1 |
| 6 | 254 | 5.91 | 0 | 0 | 0 | 1 | 1 |
| 6 | 254 | 5.93 | 0 | 0 | 0 | 1 | 1 |
| 6 | 254 | 6.03 | 0 | 0 | 1 | 1 | 1 |
| 6 | 254 | 4.87 | 0 | 0 | 1 | 1 | 1 |
| 6 | 254 | 6.07 | 0 | 0 | 0 | 1 | 1 |
| 6 | 254 | 6.08 | 0 | 0 | 0 | 1 | 1 |
| 6 | 254 | 6.09 | 0 | 0 | 1 | 1 | 1 |
| 6 | 254 | 6.1 | 0 | 0 | 0 | 1 | 1 |
| 6 | 254 | 6.11 | 0 | 0 | 0 | 1 | 1 |
| 6 | 254 | 6.12 | 0 | 0 | 0 | 1 | 1 |
| 6 | 254 | 7.82 | 0 | 0 | 0 | 0 | 1 |
| 6 | 254 | 7.83 | 0 | 0 | 0 | 0 | 1 |
| 6 | 254 | 7.84 | 0 | 0 | 1 | 0 | 1 |
| 6 | 254 | 6.98 | 0 | 0 | 0 | 1 | 1 |
| 6 | 254 | 4.88 | 0 | 0 | 0 | 1 | 1 |
| 6 | 254 | 6.99 | 0 | 0 | 0 | 1 | 1 |

| | | | | | | | |
|---|---|---|---|---|---|---|---|
| 6 | 254 | 7 | 0 | 0 | 0 | 1 | 1 |
| 6 | 254 | 6.13 | 0 | 0 | 0 | 1 | 1 |
| 6 | 254 | 6.14 | 0 | 0 | 0 | 1 | 1 |
| 6 | 254 | 6.15 | 0 | 0 | 1 | 1 | 1 |
| 6 | 254 | 9.58 | 0 | 0 | 0 | 0 | 1 |
| 6 | 254 | 9.59 | 0 | 0 | 1 | 0 | 1 |
| 6 | 254 | 8.75 | 0 | 0 | 0 | 0 | 1 |
| 6 | 254 | 8.76 | 0 | 0 | 1 | 0 | 1 |
| 6 | 254 | 4.89 | 0 | 0 | 1 | 1 | 1 |
| 6 | 254 | 8.77 | 0 | 0 | 1 | 0 | 1 |
| 6 | 254 | 3.86 | 0 | 0 | 1 | 1 | 1 |
| 6 | 254 | 3.87 | 0 | 0 | 0 | 1 | 1 |
| 6 | 254 | 4.91 | 0 | 0 | 1 | 1 | 1 |
| 6 | 254 | 4.95 | 0 | 0 | 1 | 1 | 1 |
| 6 | 254 | 4.96 | 0 | 0 | 0 | 1 | 1 |
| 6 | 254 | 4.97 | 0 | 0 | 0 | 1 | 1 |
| 6 | 254 | 4.98 | 0 | 0 | 0 | 1 | 1 |
| 6 | 254 | 5.01 | 0 | 0 | 1 | 1 | 1 |
| 6 | 254 | 5.02 | 0 | 0 | 0 | 1 | 1 |
| 6 | 254 | 5.03 | 0 | 0 | 1 | 1 | 1 |
| 6 | 254 | 5.04 | 0 | 0 | 1 | 1 | 1 |
| 6 | 254 | 5.05 | 0 | 0 | 0 | 1 | 1 |
| 6 | 254 | 5.06 | 0 | 0 | 0 | 1 | 1 |
| 6 | 254 | 5.07 | 0 | 0 | 1 | 1 | 1 |
| 6 | 254 | 5.08 | 0 | 0 | 1 | 1 | 1 |
| 6 | 254 | 5.09 | 0 | 0 | 0 | 1 | 1 |
| 6 | 254 | 5.1 | 0 | 0 | 0 | 1 | 1 |
| 6 | 254 | 5.11 | 0 | 0 | 0 | 1 | 1 |
| 6 | 254 | 5.13 | 0 | 0 | 1 | 1 | 1 |
| 6 | 254 | 5.14 | 0 | 0 | 0 | 1 | 1 |
| 6 | 254 | 5.16 | 0 | 0 | 0 | 1 | 1 |
| 6 | 254 | 6.31 | 0 | 0 | 0 | 1 | 1 |
| 6 | 254 | 5.19 | 0 | 0 | 0 | 1 | 1 |
| 6 | 254 | 5.18 | 0 | 0 | 1 | 1 | 1 |
| 6 | 254 | 5.2 | 0 | 0 | 0 | 1 | 1 |
| 6 | 254 | 5.21 | 0 | 0 | 0 | 1 | 1 |
| 6 | 254 | 5.2 | 0 | 0 | 1 | 1 | 1 |
| 6 | 254 | 5.22 | 0 | 0 | 0 | 1 | 1 |
| 6 | 254 | 5.21 | 0 | 0 | 1 | 1 | 1 |
| 6 | 254 | 5.23 | 0 | 0 | 0 | 1 | 1 |
| 6 | 254 | 5.22 | 0 | 0 | 1 | 1 | 1 |
| 6 | 254 | 5.24 | 0 | 0 | 0 | 1 | 1 |
| 6 | 254 | 2.14 | 0 | 0 | 0 | 1 | 1 |
| 6 | 254 | 5.25 | 0 | 0 | 0 | 1 | 1 |

| | | | | | | | |
|---|---|---|---|---|---|---|---|
| 6 | 254 | 2.15 | 0 | 0 | 0 | 1 | 1 |
| 6 | 254 | 5.26 | 0 | 0 | 0 | 1 | 1 |
| 6 | 254 | 2.16 | 0 | 0 | 1 | 1 | 1 |
| 6 | 254 | 5.27 | 0 | 0 | 1 | 1 | 1 |
| 6 | 254 | 2.17 | 0 | 0 | 0 | 1 | 1 |
| 6 | 254 | 5.28 | 0 | 0 | 1 | 1 | 1 |
| 6 | 254 | 2.18 | 0 | 0 | 1 | 1 | 1 |
| 6 | 254 | 5.29 | 0 | 0 | 1 | 1 | 1 |
| 6 | 254 | 2.19 | 0 | 0 | 1 | 1 | 1 |
| 6 | 254 | 5.3 | 0 | 0 | 0 | 1 | 1 |
| 6 | 254 | 5.31 | 0 | 0 | 0 | 1 | 1 |
| 6 | 254 | 2.21 | 0 | 0 | 1 | 1 | 1 |
| 6 | 254 | 5.32 | 0 | 0 | 1 | 1 | 1 |
| 6 | 254 | 2.22 | 0 | 0 | 0 | 1 | 1 |
| 6 | 254 | 5.33 | 0 | 0 | 0 | 1 | 1 |
| 6 | 254 | 5.34 | 0 | 0 | 0 | 1 | 1 |
| 6 | 254 | 5.35 | 0 | 0 | 1 | 1 | 1 |
| 6 | 254 | 2.25 | 0 | 0 | 1 | 1 | 1 |
| 6 | 254 | 5.36 | 0 | 0 | 1 | 1 | 1 |
| 6 | 254 | 2.26 | 0 | 0 | 1 | 1 | 1 |
| 6 | 254 | 5.37 | 0 | 0 | 0 | 1 | 1 |
| 6 | 254 | 2.28 | 0 | 0 | 0 | 1 | 1 |
| 6 | 254 | 5.44 | 0 | 0 | 1 | 1 | 1 |
| 6 | 254 | 5.4 | 0 | 0 | 0 | 1 | 1 |
| 6 | 254 | 5.45 | 0 | 0 | 1 | 1 | 1 |
| 6 | 254 | 5.41 | 0 | 0 | 0 | 1 | 1 |
| 6 | 254 | 5.46 | 0 | 0 | 1 | 1 | 1 |
| 6 | 254 | 5.42 | 0 | 0 | 0 | 1 | 1 |
| 6 | 254 | 5.62 | 0 | 0 | 1 | 1 | 1 |
| 6 | 254 | 5.66 | 0 | 0 | 0 | 1 | 1 |
| 6 | 254 | 5.69 | 0 | 0 | 1 | 1 | 1 |
| 6 | 254 | 5.7 | 0 | 0 | 1 | 1 | 1 |
| 6 | 254 | 5.71 | 0 | 0 | 1 | 1 | 1 |
| 6 | 254 | 5.72 | 0 | 0 | 1 | 1 | 1 |
| 6 | 254 | 5.74 | 0 | 0 | 0 | 1 | 1 |
| 6 | 254 | 5.75 | 0 | 0 | 0 | 1 | 1 |
| 6 | 254 | 6.55 | 0 | 0 | 1 | 1 | 1 |
| 6 | 254 | 6.56 | 0 | 0 | 1 | 1 | 1 |
| 6 | 254 | 6.57 | 0 | 0 | 1 | 1 | 1 |
| 6 | 254 | 6.59 | 0 | 0 | 1 | 1 | 1 |
| 6 | 256 | 15.36 | 0 | 0 | 0 | 1 | 1 |
| 6 | 256 | 15.37 | 0 | 0 | 0 | 1 | 1 |
| 6 | 256 | 15.41 | 0 | 0 | 0 | 1 | 1 |
| 6 | 256 | 15.42 | 0 | 0 | 1 | 1 | 1 |

| | | | | | | | |
|---|---|---|---|---|---|---|---|
| 6 | 256 | 15.44 | 0 | 0 | 1 | 1 | 1 |
| 6 | 256 | 15.51 | 0 | 0 | 1 | 1 | 1 |
| 6 | 256 | 16.55 | 0 | 0 | 1 | 1 | 1 |
| 6 | 256 | 16.56 | 0 | 0 | 0 | 1 | 1 |
| 6 | 256 | 16.57 | 0 | 0 | 1 | 1 | 1 |
| 6 | 256 | 16.58 | 0 | 0 | 0 | 1 | 1 |
| 6 | 256 | 16.59 | 0 | 0 | 1 | 1 | 1 |
| 6 | 256 | 16.6 | 0 | 0 | 0 | 1 | 1 |
| 6 | 256 | 16.62 | 0 | 0 | 1 | 1 | 1 |
| 6 | 256 | 16.63 | 0 | 0 | 0 | 1 | 1 |
| 6 | 256 | 16.64 | 0 | 0 | 0 | 1 | 1 |
| 6 | 256 | 16.66 | 0 | 0 | 1 | 1 | 1 |
| 6 | 256 | 16.69 | 0 | 0 | 0 | 1 | 1 |
| 6 | 256 | 16.83 | 0 | 0 | 1 | 1 | 1 |
| 6 | 256 | 16.87 | 0 | 0 | 0 | 1 | 1 |
| 6 | 256 | 16.9 | 0 | 0 | 0 | 1 | 1 |
| 6 | 256 | 16.96 | 0 | 0 | 0 | 1 | 1 |
| 6 | 256 | 16.98 | 0 | 0 | 0 | 1 | 1 |
| 6 | 256 | 16.99 | 0 | 0 | 0 | 1 | 1 |
| 6 | 256 | 17 | 0 | 0 | 0 | 1 | 1 |
| 6 | 256 | 17.01 | 0 | 0 | 0 | 1 | 1 |
| 6 | 256 | 17.02 | 0 | 0 | 0 | 1 | 1 |
| 5 | 256 | 17.06 | 0 | 0 | 0 | 1 | 1 |
| 5 | 256 | 17.07 | 0 | 0 | 1 | 1 | 1 |
| 5 | 256 | 17.08 | 0 | 0 | 1 | 1 | 1 |
| 5 | 256 | 17.09 | 0 | 0 | 0 | 1 | 1 |
| 5 | 256 | 17.1 | 0 | 0 | 0 | 1 | 1 |
| 5 | 256 | 17.11 | 0 | 0 | 1 | 1 | 1 |
| 5 | 256 | 17.12 | 0 | 0 | 0 | 1 | 1 |
| 5 | 256 | 17.13 | 0 | 0 | 1 | 1 | 1 |
| 5 | 256 | 17.14 | 0 | 0 | 1 | 1 | 1 |
| 5 | 256 | 17.15 | 0 | 0 | 0 | 1 | 1 |
| 5 | 256 | 17.18 | 0 | 0 | 0 | 1 | 1 |
| 5 | 256 | 17.23 | 0 | 0 | 0 | 1 | 1 |
| 5 | 256 | 17.38 | 0 | 0 | 1 | 1 | 1 |
| 5 | 256 | 17.39 | 0 | 0 | 1 | 1 | 1 |
| 5 | 256 | 17.4 | 0 | 0 | 1 | 1 | 1 |
| 5 | 256 | 17.41 | 0 | 0 | 0 | 1 | 1 |
| 5 | 256 | 17.42 | 0 | 0 | 0 | 1 | 1 |
| 5 | 256 | 17.43 | 0 | 0 | 1 | 1 | 1 |
| 5 | 256 | 17.46 | 0 | 0 | 0 | 1 | 1 |
| 5 | 256 | 17.49 | 0 | 0 | 0 | 1 | 1 |
| 5 | 256 | 17.27 | 0 | 0 | 0 | 1 | 1 |
| 5 | 256 | 17.76 | 0 | 0 | 0 | 1 | 1 |

| | | | | | | | |
|---|---|---|---|---|---|---|---|
| 5 | 256 | 17.78 | 0 | 0 | 0 | 1 | 1 |
| 5 | 256 | 17.83 | 0 | 0 | 0 | 1 | 1 |
| 5 | 256 | 17.86 | 0 | 0 | 1 | 1 | 1 |
| 5 | 256 | 17.87 | 0 | 0 | 1 | 1 | 1 |
| 5 | 256 | 17.88 | 0 | 0 | 0 | 1 | 1 |
| 5 | 256 | 17.89 | 0 | 0 | 0 | 1 | 1 |
| 5 | 256 | 17.9 | 0 | 0 | 0 | 1 | 1 |
| 5 | 256 | 17.91 | 0 | 0 | 0 | 1 | 1 |
| 5 | 256 | 17.92 | 0 | 0 | 1 | 1 | 1 |
| 5 | 256 | 17.93 | 0 | 0 | 0 | 1 | 1 |
| 5 | 256 | 18.24 | 0 | 0 | 1 | 1 | 1 |
| 5 | 256 | 18.25 | 0 | 0 | 1 | 1 | 1 |
| 5 | 256 | 18.26 | 0 | 0 | 0 | 1 | 1 |
| 5 | 256 | 18.27 | 0 | 0 | 0 | 1 | 1 |
| 5 | 256 | 18.28 | 0 | 0 | 1 | 1 | 1 |
| 5 | 256 | 18.29 | 0 | 0 | 0 | 1 | 1 |
| 5 | 256 | 18.3 | 0 | 0 | 0 | 1 | 1 |
| 5 | 256 | 18.31 | 0 | 0 | 1 | 1 | 1 |
| 5 | 256 | 18.32 | 0 | 0 | 1 | 1 | 1 |
| 5 | 256 | 18.33 | 0 | 0 | 0 | 1 | 1 |
| 5 | 256 | 18.34 | 0 | 0 | 0 | 1 | 1 |
| 5 | 256 | 18.35 | 0 | 0 | 0 | 1 | 1 |
| 5 | 256 | 18.36 | 0 | 0 | 1 | 1 | 1 |
| 5 | 256 | 18.38 | 0 | 0 | 1 | 1 | 1 |
| 5 | 256 | 18.39 | 0 | 0 | 1 | 1 | 1 |
| 5 | 256 | 18.4 | 0 | 0 | 1 | 1 | 1 |
| 5 | 256 | 18.41 | 0 | 0 | 1 | 1 | 1 |
| 5 | 256 | 18.42 | 0 | 0 | 0 | 1 | 1 |
| 5 | 256 | 18.43 | 0 | 0 | 0 | 1 | 1 |
| 5 | 256 | 18.44 | 0 | 0 | 1 | 1 | 1 |
| 5 | 256 | 18.45 | 0 | 0 | 0 | 1 | 1 |
| 5 | 256 | 18.46 | 0 | 0 | 1 | 1 | 1 |
| 6 | 245 | 3.9 | 1 | 0 | 0 | 1 | 1 |
| 6 | 250 | 3.94 | 1 | 0 | 0 | 1 | 1 |
| 6 | 250 | 5.82 | 1 | 0 | 0 | 1 | 1 |
| 6 | 254 | 4 | 1 | 0 | 1 | 1 | 1 |
| 6 | 254 | 5.76 | 1 | 0 | 0 | 1 | 1 |
| 6 | 254 | 5.75 | 1 | 0 | 1 | 1 | 1 |
| 6 | 254 | 5.7 | 1 | 0 | 0 | 1 | 1 |
| 6 | 254 | 8.38 | 1 | 0 | 0 | 1 | 1 |
| 6 | 254 | 5.65 | 1 | 0 | 0 | 1 | 1 |
| 6 | 254 | 5.62 | 1 | 0 | 0 | 1 | 1 |
| 6 | 254 | 8.4 | 1 | 0 | 1 | 1 | 1 |
| 6 | 254 | 5.61 | 1 | 0 | 1 | 1 | 1 |

| | | | | | | | |
|---|---|---|---|---|---|---|---|
| 6 | 254 | 5.6 | 1 | 0 | 0 | 1 | 1 |
| 6 | 254 | 5.59 | 1 | 0 | 0 | 1 | 1 |
| 6 | 254 | 5.58 | 1 | 0 | 0 | 1 | 1 |
| 6 | 254 | 5.56 | 1 | 0 | 0 | 1 | 1 |
| 6 | 254 | 8.43 | 1 | 0 | 0 | 1 | 1 |
| 6 | 254 | 5.53 | 1 | 0 | 0 | 1 | 1 |
| 6 | 254 | 5.52 | 1 | 0 | 1 | 1 | 1 |
| 6 | 254 | 5.5 | 1 | 0 | 0 | 1 | 1 |
| 6 | 254 | 5.49 | 1 | 0 | 0 | 1 | 1 |
| 6 | 254 | 8.45 | 1 | 0 | 1 | 1 | 1 |
| 6 | 254 | 5.48 | 1 | 0 | 1 | 1 | 1 |
| 6 | 254 | 5.46 | 1 | 0 | 0 | 1 | 1 |
| 6 | 254 | 8.46 | 1 | 0 | 0 | 1 | 1 |
| 6 | 254 | 5.4 | 1 | 0 | 0 | 1 | 1 |
| 6 | 254 | 8.79 | 1 | 0 | 1 | 0 | 1 |
| 6 | 254 | 8.86 | 1 | 0 | 1 | 0 | 1 |
| 6 | 254 | 8.87 | 1 | 0 | 0 | 0 | 1 |
| 6 | 254 | 7.85 | 1 | 0 | 0 | 0 | 1 |
| 6 | 254 | 7.87 | 1 | 0 | 1 | 0 | 1 |
| 6 | 254 | 7.88 | 1 | 0 | 1 | 0 | 1 |
| 6 | 254 | 7.89 | 1 | 0 | 0 | 0 | 1 |
| 6 | 254 | 7.91 | 1 | 0 | 0 | 0 | 1 |
| 6 | 254 | 8.51 | 1 | 1 | 0 | 1 | 1 |
| 6 | 254 | 7.92 | 1 | 0 | 0 | 0 | 1 |
| 6 | 254 | 7.93 | 1 | 0 | 0 | 0 | 1 |
| 6 | 254 | 7.94 | 1 | 0 | 0 | 0 | 1 |
| 6 | 254 | 7.95 | 1 | 0 | 0 | 0 | 1 |
| 6 | 254 | 7.96 | 1 | 1 | 0 | 0 | 1 |
| 6 | 254 | 7.97 | 1 | 0 | 1 | 0 | 1 |
| 6 | 254 | 7.98 | 1 | 0 | 1 | 0 | 1 |
| 6 | 254 | 7.99 | 1 | 0 | 0 | 0 | 1 |
| 6 | 254 | 8 | 1 | 0 | 0 | 1 | 1 |
| 6 | 254 | 8.01 | 1 | 0 | 0 | 1 | 1 |
| 6 | 254 | 8.52 | 1 | 1 | 0 | 1 | 1 |
| 6 | 254 | 8.02 | 1 | 0 | 1 | 1 | 1 |
| 6 | 254 | 8.03 | 1 | 0 | 0 | 1 | 1 |
| 6 | 254 | 8.04 | 1 | 0 | 0 | 1 | 1 |
| 6 | 254 | 8.05 | 1 | 1 | 0 | 1 | 1 |
| 6 | 254 | 8.06 | 1 | 0 | 0 | 1 | 1 |
| 6 | 254 | 8.1 | 1 | 0 | 0 | 1 | 1 |
| 6 | 254 | 7.02 | 1 | 0 | 1 | 1 | 1 |
| 6 | 254 | 7.03 | 1 | 0 | 0 | 1 | 1 |
| 6 | 254 | 7.04 | 1 | 0 | 1 | 1 | 1 |
| 6 | 254 | 7.05 | 1 | 0 | 1 | 1 | 1 |

| | | | | | | | |
|---|---|---|---|---|---|---|---|
| 6 | 254 | 7.06 | 1 | 0 | 1 | 1 | 1 |
| 6 | 254 | 8.59 | 1 | 0 | 0 | 1 | 1 |
| 6 | 254 | 8.6 | 1 | 0 | 1 | 1 | 1 |
| 6 | 254 | 6.22 | 1 | 0 | 0 | 1 | 1 |
| 6 | 254 | 6.23 | 1 | 0 | 1 | 1 | 1 |
| 6 | 254 | 6.27 | 1 | 0 | 0 | 1 | 1 |
| 6 | 254 | 6.3 | 1 | 0 | 0 | 1 | 1 |
| 6 | 254 | 5.85 | 1 | 0 | 0 | 1 | 1 |
| 6 | 254 | 5.86 | 1 | 0 | 1 | 1 | 1 |
| 6 | 254 | 5.91 | 1 | 0 | 1 | 1 | 1 |
| 6 | 254 | 5.92 | 1 | 0 | 0 | 1 | 1 |
| 6 | 254 | 5.93 | 1 | 0 | 0 | 1 | 1 |
| 6 | 254 | 5.94 | 1 | 0 | 0 | 1 | 1 |
| 6 | 256 | 5.97 | 1 | 0 | 0 | 1 | 1 |
| 6 | 256 | 6.06 | 1 | 0 | 0 | 1 | 1 |
| 6 | 256 | 6.1 | 1 | 0 | 0 | 1 | 1 |
| 6 | 256 | 6.11 | 1 | 0 | 0 | 1 | 1 |
| 6 | 256 | 6.12 | 1 | 0 | 0 | 1 | 1 |
| 6 | 256 | 6.14 | 1 | 0 | 0 | 1 | 1 |
| 6 | 256 | 14.36 | 1 | 0 | 0 | 1 | 1 |
| 6 | 256 | 14.37 | 1 | 0 | 0 | 1 | 1 |
| 6 | 256 | 14 | 1 | 0 | 1 | 1 | 1 |
| 6 | 256 | 0.72 | 1 | 0 | 0 | 1 | 1 |
| 6 | 256 | 0.71 | 1 | 0 | 0 | 1 | 1 |
| 6 | 256 | 0.7 | 1 | 0 | 0 | 1 | 1 |
| 6 | 256 | 0.69 | 1 | 0 | 1 | 1 | 1 |
| 6 | 256 | 14.18 | 1 | 0 | 0 | 1 | 1 |
| 6 | 256 | 14.19 | 1 | 0 | 0 | 1 | 1 |
| 6 | 256 | 13.95 | 1 | 0 | 1 | 1 | 1 |
| 6 | 256 | 13.94 | 1 | 0 | 0 | 1 | 1 |
| 6 | 256 | 13.91 | 1 | 0 | 0 | 1 | 1 |
| 6 | 256 | 0.96 | 1 | 0 | 0 | 1 | 1 |
| 6 | 256 | 0.94 | 1 | 0 | 0 | 1 | 1 |
| 6 | 256 | 0.81 | 1 | 0 | 0 | 1 | 1 |
| 6 | 256 | 15.05 | 1 | 0 | 0 | 1 | 1 |
| 6 | 256 | 15.01 | 1 | 0 | 1 | 1 | 1 |
| 6 | 256 | 14.99 | 1 | 0 | 1 | 1 | 1 |
| 6 | 256 | 14.91 | 1 | 0 | 1 | 1 | 1 |
| 6 | 256 | 14.89 | 1 | 0 | 0 | 1 | 1 |
| 6 | 256 | 14.86 | 1 | 0 | 1 | 1 | 1 |
| 6 | 256 | 15.07 | 1 | 0 | 1 | 1 | 1 |
| 6 | 256 | 16.52 | 1 | 1 | 1 | 0 | 1 |
| 6 | 256 | 15.27 | 1 | 0 | 1 | 1 | 1 |
| 6 | 256 | 15.28 | 1 | 0 | 1 | 1 | 1 |

| | | | | | | | |
|---|---|---|---|---|---|---|---|
| 6 | 256 | 15.33 | 1 | 0 | 1 | 1 | 1 |
| 6 | 256 | 15.56 | 1 | 0 | 1 | 1 | 1 |
| 6 | 256 | 15.57 | 1 | 0 | 1 | 1 | 1 |
| 6 | 256 | 15.59 | 1 | 0 | 0 | 1 | 1 |
| 6 | 256 | 15.6 | 1 | 0 | 0 | 1 | 1 |
| 6 | 256 | 15.65 | 1 | 0 | 1 | 1 | 1 |
| 5 | 256 | 18.48 | 1 | 0 | 0 | 1 | 1 |
| 5 | 256 | 18.5 | 1 | 0 | 0 | 1 | 1 |
| 5 | 256 | 17.99 | 1 | 0 | 0 | 1 | 1 |
| 5 | 256 | 18.04 | 1 | 0 | 1 | 1 | 1 |
| 5 | 256 | 18.05 | 1 | 0 | 0 | 1 | 1 |
| 5 | 256 | 18.19 | 1 | 0 | 0 | 1 | 1 |
| 5 | 256 | 18.21 | 1 | 0 | 0 | 1 | 1 |
| 5 | 256 | 18.22 | 1 | 0 | 1 | 1 | 1 |
| 5 | 256 | 18.23 | 1 | 0 | 0 | 1 | 1 |
| 5 | 256 | 18.52 | 1 | 0 | 0 | 1 | 1 |
| 5 | 256 | 18.53 | 1 | 0 | 1 | 1 | 1 |
| 5 | 256 | 18.54 | 1 | 0 | 0 | 1 | 1 |
| 5 | 256 | 18.55 | 1 | 0 | 0 | 1 | 1 |
| 5 | 256 | 18.56 | 1 | 0 | 0 | 1 | 1 |
| 5 | 256 | 18.57 | 1 | 0 | 0 | 1 | 1 |
| 5 | 256 | 18.58 | 1 | 0 | 0 | 1 | 1 |
| 5 | 256 | 18.59 | 1 | 0 | 1 | 1 | 1 |
| 5 | 256 | 18.6 | 1 | 0 | 1 | 1 | 1 |
| 5 | 256 | 18.61 | 1 | 0 | 1 | 1 | 1 |
| 5 | 256 | 18.62 | 1 | 0 | 0 | 1 | 1 |
| 5 | 256 | 18.63 | 1 | 0 | 0 | 1 | 1 |
| 5 | 256 | 18.64 | 1 | 0 | 0 | 1 | 1 |
| 5 | 256 | 18.65 | 1 | 0 | 0 | 1 | 1 |
| 5 | 256 | 18.66 | 1 | 0 | 1 | 1 | 1 |
| 5 | 256 | 18.67 | 1 | 0 | 1 | 1 | 1 |
| 5 | 256 | 18.68 | 1 | 0 | 0 | 1 | 1 |
| 5 | 256 | 18.69 | 1 | 0 | 0 | 1 | 1 |
| 5 | 256 | 18.7 | 1 | 0 | 0 | 1 | 1 |
| 5 | 256 | 18.71 | 1 | 0 | 0 | 1 | 1 |
| 5 | 256 | 18.75 | 1 | 1 | 0 | 1 | 1 |
| 5 | 256 | 18.79 | 1 | 0 | 0 | 1 | 1 |
| 5 | 256 | 18.82 | 1 | 0 | 0 | 1 | 1 |
| 5 | 256 | 18.84 | 1 | 0 | 1 | 1 | 1 |
| 5 | 256 | 18.85 | 1 | 0 | 0 | 1 | 1 |
| 5 | 256 | 18.86 | 1 | 0 | 1 | 1 | 1 |
| 5 | 256 | 18.87 | 1 | 0 | 0 | 1 | 1 |
| 5 | 256 | 24.01 | 1 | 0 | 0 | 1 | 1 |
| 5 | 256 | 24.01 | 1 | 0 | 0 | 1 | 1 |

| | | | | | | | |
|---|---|---|---|---|---|---|---|
| 5 | 256 | 19.01 | 1 | 0 | 0 | 1 | 1 |
| 5 | 256 | 19 | 1 | 0 | 0 | 1 | 1 |
| 5 | 256 | 24.01 | 1 | 0 | 0 | 1 | 1 |
| 5 | 256 | 24.01 | 1 | 0 | 0 | 1 | 1 |
| 5 | 256 | 24.01 | 1 | 0 | 0 | 1 | 1 |
| 5 | 256 | 24.01 | 1 | 0 | 0 | 1 | 1 |
| 5 | 256 | 24.01 | 1 | 0 | 0 | 1 | 1 |
| 5 | 256 | 24.01 | 1 | 0 | 0 | 1 | 1 |
| 5 | 256 | 24.01 | 1 | 0 | 0 | 1 | 1 |
| 5 | 256 | 24.01 | 1 | 0 | 0 | 1 | 1 |
| 10 | 180 | 24.01236 | 1 | 1 | 1 | 0 | 0 |
| 9 | 180 | 17.27 | 1 | 1 | 0 | 0 | 0 |
| 9 | 180 | 17.35 | 1 | 1 | 0 | 0 | 0 |
| 7 | 230 | 11.77 | 1 | 0 | 1 | 0 | 0 |
| 5 | 245 | 7.39 | 1 | 1 | 0 | 1 | 1 |
| 5 | 245 | 9.15 | 1 | 0 | 0 | 0 | 1 |
| 5 | 245 | 9.2 | 1 | 0 | 0 | 0 | 1 |
| 5 | 245 | 9.21 | 1 | 0 | 0 | 0 | 1 |
| 5 | 245 | 9.25 | 1 | 0 | 0 | 0 | 1 |
| 5 | 245 | 9.26 | 1 | 0 | 0 | 0 | 1 |
| 5 | 245 | 7.35 | 1 | 0 | 0 | 1 | 1 |
| 5 | 245 | 9.28 | 1 | 0 | 0 | 0 | 1 |
| 6 | 245 | 4.63 | 1 | 0 | 0 | 1 | 1 |
| 6 | 245 | 9.34 | 1 | 0 | 0 | 0 | 1 |
| 6 | 245 | 9.35 | 1 | 0 | 1 | 0 | 1 |
| 6 | 245 | 7.33 | 1 | 1 | 1 | 1 | 1 |
| 6 | 245 | 9.37 | 1 | 0 | 1 | 0 | 1 |
| 6 | 245 | 9.38 | 1 | 0 | 0 | 0 | 1 |
| 6 | 245 | 9.42 | 1 | 0 | 0 | 0 | 1 |
| 6 | 245 | 9.45 | 1 | 0 | 0 | 0 | 1 |
| 6 | 245 | 9.47 | 1 | 1 | 0 | 0 | 1 |
| 6 | 245 | 9.48 | 1 | 1 | 0 | 0 | 1 |
| 6 | 245 | 9.49 | 1 | 1 | 0 | 0 | 1 |
| 6 | 245 | 9.53 | 1 | 0 | 0 | 0 | 1 |
| 6 | 245 | 4.65 | 1 | 0 | 0 | 1 | 1 |
| 6 | 245 | 9.54 | 1 | 0 | 0 | 0 | 1 |
| 6 | 245 | 9.56 | 1 | 0 | 0 | 0 | 1 |
| 6 | 245 | 0.35 | 1 | 0 | 1 | 1 | 1 |
| 6 | 245 | 0.33 | 1 | 0 | 0 | 1 | 1 |
| 6 | 245 | 0.32 | 1 | 0 | 1 | 1 | 1 |
| 6 | 245 | 0.31 | 1 | 0 | 1 | 1 | 1 |
| 6 | 250 | 0.3 | 1 | 0 | 0 | 1 | 1 |
| 6 | 250 | 6.49 | 1 | 0 | 0 | 1 | 1 |
| 6 | 250 | 0.26 | 1 | 0 | 0 | 1 | 1 |

| | | | | | | | |
|---|---|---|---|---|---|---|---|
| 6 | 250 | 0.16 | 1 | 0 | 0 | 1 | 1 |
| 6 | 250 | 4.69 | 1 | 0 | 0 | 1 | 1 |
| 6 | 250 | 1.82 | 1 | 0 | 1 | 1 | 1 |
| 6 | 250 | 1.83 | 1 | 0 | 0 | 1 | 1 |
| 6 | 250 | 1.91 | 1 | 0 | 0 | 1 | 1 |
| 6 | 250 | 1.22 | 1 | 0 | 0 | 1 | 1 |
| 6 | 250 | 4.71 | 1 | 0 | 0 | 1 | 1 |
| 6 | 250 | 1.92 | 1 | 0 | 0 | 1 | 1 |
| 6 | 250 | 1.94 | 1 | 0 | 0 | 1 | 1 |
| 6 | 250 | 1.99 | 1 | 0 | 0 | 1 | 1 |
| 6 | 250 | 2.01 | 1 | 0 | 1 | 1 | 1 |
| 6 | 250 | 8.72 | 1 | 0 | 0 | 1 | 1 |
| 6 | 250 | 8.73 | 1 | 0 | 0 | 1 | 1 |
| 6 | 250 | 8.75 | 1 | 0 | 0 | 1 | 1 |
| 6 | 254 | 8.81 | 1 | 0 | 1 | 1 | 1 |
| 6 | 254 | 8.84 | 1 | 0 | 0 | 1 | 1 |
| 6 | 254 | 8.97 | 1 | 0 | 0 | 1 | 1 |
| 6 | 254 | 8.98 | 1 | 0 | 0 | 1 | 1 |
| 6 | 254 | 3.72 | 1 | 0 | 0 | 1 | 1 |
| 6 | 254 | 3.76 | 1 | 0 | 0 | 1 | 1 |
| 6 | 254 | 3.77 | 1 | 0 | 0 | 1 | 1 |
| 6 | 254 | 3.79 | 1 | 0 | 0 | 1 | 1 |
| 6 | 254 | 4.78 | 1 | 0 | 1 | 1 | 1 |
| 6 | 254 | 7.8 | 1 | 0 | 0 | 1 | 1 |
| 6 | 254 | 4.79 | 1 | 0 | 1 | 1 | 1 |
| 6 | 254 | 7.82 | 1 | 0 | 0 | 1 | 1 |
| 6 | 254 | 7.84 | 1 | 0 | 0 | 1 | 1 |
| 6 | 254 | 7.85 | 1 | 0 | 1 | 1 | 1 |
| 6 | 254 | 7.86 | 1 | 0 | 0 | 1 | 1 |
| 6 | 254 | 7.87 | 1 | 1 | 0 | 1 | 1 |
| 6 | 254 | 7.88 | 1 | 0 | 0 | 1 | 1 |
| 6 | 254 | 7.93 | 1 | 0 | 1 | 1 | 1 |
| 6 | 254 | 7.96 | 1 | 0 | 0 | 1 | 1 |
| 6 | 254 | 7.98 | 1 | 0 | 0 | 1 | 1 |
| 6 | 254 | 8 | 1 | 0 | 1 | 1 | 1 |
| 6 | 254 | 8.01 | 1 | 0 | 1 | 1 | 1 |
| 6 | 254 | 4.81 | 1 | 0 | 1 | 1 | 1 |
| 6 | 254 | 8.02 | 1 | 0 | 1 | 1 | 1 |
| 6 | 254 | 8.03 | 1 | 0 | 0 | 1 | 1 |
| 6 | 254 | 8.04 | 1 | 0 | 0 | 1 | 1 |
| 6 | 254 | 8.05 | 1 | 1 | 0 | 1 | 1 |
| 6 | 254 | 8.06 | 1 | 0 | 1 | 1 | 1 |
| 6 | 254 | 8.07 | 1 | 0 | 0 | 1 | 1 |
| 6 | 254 | 8.08 | 1 | 0 | 0 | 1 | 1 |

| | | | | | | | |
|---|---|---|---|---|---|---|---|
| 6 | 254 | 8.1 | 1 | 0 | 0 | 1 | 1 |
| 6 | 254 | 8.11 | 1 | 0 | 0 | 1 | 1 |
| 6 | 254 | 4.82 | 1 | 0 | 1 | 1 | 1 |
| 6 | 254 | 8.12 | 1 | 0 | 1 | 1 | 1 |
| 6 | 254 | 8.14 | 1 | 0 | 1 | 1 | 1 |
| 6 | 254 | 8.15 | 1 | 0 | 1 | 1 | 1 |
| 6 | 254 | 8.16 | 1 | 0 | 1 | 1 | 1 |
| 6 | 254 | 8.17 | 1 | 0 | 0 | 1 | 1 |
| 6 | 254 | 8.18 | 1 | 0 | 0 | 1 | 1 |
| 6 | 254 | 8.22 | 1 | 0 | 0 | 1 | 1 |
| 6 | 254 | 8.23 | 1 | 0 | 0 | 1 | 1 |
| 6 | 254 | 8.31 | 1 | 1 | 0 | 1 | 1 |
| 6 | 254 | 8.33 | 1 | 0 | 0 | 1 | 1 |
| 6 | 254 | 8.34 | 1 | 0 | 1 | 1 | 1 |
| 6 | 254 | 8.35 | 1 | 0 | 1 | 1 | 1 |
| 6 | 254 | 5.82 | 1 | 0 | 1 | 1 | 1 |
| 6 | 254 | 5.83 | 1 | 0 | 0 | 1 | 1 |
| 6 | 254 | 5.87 | 1 | 0 | 0 | 1 | 1 |
| 6 | 254 | 5.88 | 1 | 0 | 0 | 1 | 1 |
| 6 | 254 | 5.9 | 1 | 0 | 1 | 1 | 1 |
| 6 | 254 | 5.92 | 1 | 0 | 0 | 1 | 1 |
| 6 | 254 | 5.94 | 1 | 0 | 0 | 1 | 1 |
| 6 | 254 | 5.95 | 1 | 0 | 0 | 1 | 1 |
| 6 | 254 | 5.96 | 1 | 0 | 0 | 1 | 1 |
| 6 | 254 | 4.86 | 1 | 0 | 0 | 1 | 1 |
| 6 | 254 | 5.97 | 1 | 0 | 1 | 1 | 1 |
| 6 | 254 | 5.98 | 1 | 0 | 1 | 1 | 1 |
| 6 | 254 | 5.99 | 1 | 0 | 0 | 1 | 1 |
| 6 | 254 | 6 | 1 | 0 | 0 | 1 | 1 |
| 6 | 254 | 6.01 | 1 | 0 | 1 | 1 | 1 |
| 6 | 254 | 6.02 | 1 | 0 | 0 | 1 | 1 |
| 6 | 254 | 6.04 | 1 | 0 | 0 | 1 | 1 |
| 6 | 254 | 6.05 | 1 | 0 | 0 | 1 | 1 |
| 6 | 254 | 6.06 | 1 | 0 | 0 | 1 | 1 |
| 6 | 254 | 9.57 | 1 | 0 | 0 | 0 | 1 |
| 6 | 254 | 4.9 | 1 | 0 | 0 | 1 | 1 |
| 6 | 254 | 4.92 | 1 | 0 | 0 | 1 | 1 |
| 6 | 254 | 4.93 | 1 | 0 | 0 | 1 | 1 |
| 6 | 254 | 4.94 | 1 | 0 | 0 | 1 | 1 |
| 6 | 254 | 4.99 | 1 | 0 | 0 | 1 | 1 |
| 6 | 254 | 5 | 1 | 0 | 0 | 1 | 1 |
| 6 | 254 | 5.12 | 1 | 0 | 0 | 1 | 1 |
| 6 | 254 | 5.15 | 1 | 0 | 1 | 1 | 1 |
| 6 | 254 | 5.17 | 1 | 0 | 0 | 1 | 1 |

| | | | | | | | |
|---|---|---|---|---|---|---|---|
| 6 | 254 | 5.19 | 1 | 0 | 0 | 1 | 1 |
| 6 | 254 | 2.2 | 1 | 0 | 1 | 1 | 1 |
| 6 | 254 | 2.23 | 1 | 0 | 0 | 1 | 1 |
| 6 | 254 | 2.24 | 1 | 0 | 1 | 1 | 1 |
| 6 | 254 | 2.27 | 1 | 0 | 0 | 1 | 1 |
| 6 | 254 | 5.38 | 1 | 0 | 0 | 1 | 1 |
| 6 | 254 | 5.39 | 1 | 0 | 1 | 1 | 1 |
| 6 | 254 | 2.29 | 1 | 0 | 1 | 1 | 1 |
| 6 | 254 | 24.01214 | 1 | 1 | 0 | 0 | 1 |
| 6 | 254 | 5.63 | 1 | 0 | 1 | 1 | 1 |
| 6 | 254 | 5.64 | 1 | 0 | 1 | 1 | 1 |
| 6 | 254 | 5.65 | 1 | 0 | 1 | 1 | 1 |
| 6 | 254 | 5.67 | 1 | 0 | 0 | 1 | 1 |
| 6 | 254 | 5.68 | 1 | 0 | 0 | 1 | 1 |
| 6 | 254 | 5.73 | 1 | 0 | 0 | 1 | 1 |
| 6 | 254 | 6.58 | 1 | 0 | 0 | 1 | 1 |
| 6 | 254 | 24.01215 | 1 | 1 | 0 | 0 | 1 |
| 6 | 256 | 15.38 | 1 | 0 | 1 | 1 | 1 |
| 6 | 256 | 15.39 | 1 | 0 | 1 | 1 | 1 |
| 6 | 256 | 15.4 | 1 | 0 | 0 | 1 | 1 |
| 6 | 256 | 15.43 | 1 | 0 | 1 | 1 | 1 |
| 6 | 256 | 15.45 | 1 | 0 | 0 | 1 | 1 |
| 6 | 256 | 15.46 | 1 | 0 | 0 | 1 | 1 |
| 6 | 256 | 15.47 | 1 | 0 | 0 | 1 | 1 |
| 6 | 256 | 15.48 | 1 | 0 | 1 | 1 | 1 |
| 6 | 256 | 15.49 | 1 | 0 | 1 | 1 | 1 |
| 6 | 256 | 15.5 | 1 | 0 | 1 | 1 | 1 |
| 6 | 256 | 16.61 | 1 | 0 | 1 | 1 | 1 |
| 6 | 256 | 16.65 | 1 | 0 | 0 | 1 | 1 |
| 6 | 256 | 16.67 | 1 | 0 | 0 | 1 | 1 |
| 6 | 256 | 16.68 | 1 | 0 | 0 | 1 | 1 |
| 6 | 256 | 16.7 | 1 | 0 | 0 | 1 | 1 |
| 6 | 256 | 16.71 | 1 | 0 | 0 | 1 | 1 |
| 6 | 256 | 16.72 | 1 | 0 | 0 | 1 | 1 |
| 6 | 256 | 16.73 | 1 | 0 | 0 | 1 | 1 |
| 6 | 256 | 16.74 | 1 | 0 | 0 | 1 | 1 |
| 6 | 256 | 16.75 | 1 | 0 | 1 | 1 | 1 |
| 6 | 256 | 16.76 | 1 | 0 | 1 | 1 | 1 |
| 6 | 256 | 16.77 | 1 | 0 | 1 | 1 | 1 |
| 6 | 256 | 16.78 | 1 | 0 | 1 | 1 | 1 |
| 6 | 256 | 16.79 | 1 | 0 | 1 | 1 | 1 |
| 6 | 256 | 16.8 | 1 | 0 | 1 | 1 | 1 |
| 6 | 256 | 16.81 | 1 | 0 | 1 | 1 | 1 |
| 6 | 256 | 16.82 | 1 | 0 | 1 | 1 | 1 |

| | | | | | | | |
|---|---|---|---|---|---|---|---|
| 6 | 256 | 16.84 | 1 | 0 | 1 | 1 | 1 |
| 6 | 256 | 16.85 | 1 | 0 | 1 | 1 | 1 |
| 6 | 256 | 16.86 | 1 | 0 | 1 | 1 | 1 |
| 6 | 256 | 16.88 | 1 | 0 | 0 | 1 | 1 |
| 6 | 256 | 16.89 | 1 | 0 | 0 | 1 | 1 |
| 6 | 256 | 16.91 | 1 | 0 | 0 | 1 | 1 |
| 6 | 256 | 16.92 | 1 | 0 | 1 | 1 | 1 |
| 6 | 256 | 16.93 | 1 | 0 | 0 | 1 | 1 |
| 6 | 256 | 16.94 | 1 | 0 | 0 | 1 | 1 |
| 6 | 256 | 16.95 | 1 | 0 | 0 | 1 | 1 |
| 6 | 256 | 16.97 | 1 | 0 | 1 | 1 | 1 |
| 5 | 256 | 17.03 | 1 | 0 | 0 | 1 | 1 |
| 5 | 256 | 17.04 | 1 | 0 | 0 | 1 | 1 |
| 5 | 256 | 17.05 | 1 | 0 | 0 | 1 | 1 |
| 5 | 256 | 17.16 | 1 | 0 | 0 | 1 | 1 |
| 5 | 256 | 17.17 | 1 | 0 | 0 | 1 | 1 |
| 5 | 256 | 17.19 | 1 | 0 | 1 | 1 | 1 |
| 5 | 256 | 17.2 | 1 | 0 | 1 | 1 | 1 |
| 5 | 256 | 17.21 | 1 | 0 | 0 | 1 | 1 |
| 5 | 256 | 17.22 | 1 | 0 | 0 | 1 | 1 |
| 5 | 256 | 17.24 | 1 | 0 | 0 | 1 | 1 |
| 5 | 256 | 17.25 | 1 | 0 | 0 | 1 | 1 |
| 5 | 256 | 16.54 | 1 | 0 | 0 | 1 | 1 |
| 5 | 256 | 17.44 | 1 | 0 | 0 | 1 | 1 |
| 5 | 256 | 17.45 | 1 | 0 | 0 | 1 | 1 |
| 5 | 256 | 17.47 | 1 | 0 | 0 | 1 | 1 |
| 5 | 256 | 17.48 | 1 | 0 | 0 | 1 | 1 |
| 5 | 256 | 17.26 | 1 | 0 | 1 | 1 | 1 |
| 5 | 256 | 17.28 | 1 | 0 | 1 | 1 | 1 |
| 5 | 256 | 17.29 | 1 | 0 | 0 | 1 | 1 |
| 5 | 256 | 17.3 | 1 | 0 | 1 | 1 | 1 |
| 5 | 256 | 17.31 | 1 | 0 | 0 | 1 | 1 |
| 5 | 256 | 17.32 | 1 | 0 | 1 | 1 | 1 |
| 5 | 256 | 17.33 | 1 | 0 | 1 | 1 | 1 |
| 5 | 256 | 17.34 | 1 | 0 | 0 | 1 | 1 |
| 5 | 256 | 17.35 | 1 | 0 | 0 | 1 | 1 |
| 5 | 256 | 17.36 | 1 | 0 | 0 | 1 | 1 |
| 5 | 256 | 17.37 | 1 | 0 | 0 | 1 | 1 |
| 5 | 256 | 17.79 | 1 | 0 | 0 | 1 | 1 |
| 5 | 256 | 17.75 | 1 | 0 | 0 | 1 | 1 |
| 5 | 256 | 17.77 | 1 | 0 | 0 | 1 | 1 |
| 5 | 256 | 17.8 | 1 | 0 | 0 | 1 | 1 |
| 5 | 256 | 17.81 | 1 | 0 | 0 | 1 | 1 |
| 5 | 256 | 17.82 | 1 | 0 | 0 | 1 | 1 |

| | | | | | | | |
|---|---|---|---|---|---|---|---|
| 5 | 256 | 17.84 | 1 | 0 | 0 | 1 | 1 |
| 5 | 256 | 17.85 | 1 | 0 | 0 | 1 | 1 |
| 5 | 256 | 18.37 | 1 | 0 | 1 | 1 | 1 |
